# Supplementary material for: MVsim is a toolset for quantifying and designing multivalent interactions
Source: Nat Commun. 2022 Sep 6;13:5029. doi: 10.1038/s41467-022-32496-6 (PMC9448752; doi:10.1038/s41467-022-32496-6)
Supplement: Supplementary file 1 — Supplementary Information [file 41467_2022_32496_MOESM1_ESM.pdf]

Supplementary Information for:

MVsim is a toolset for quantifying and designing multivalent interactions

Bence Bruncsics[a,*], Wesley J. Errington[b,*], and Casim A. Sarkar[b,1]

[a] Department of Measurement and Information Systems, Budapest University of Technology and Economics, Budapest H-1111, Hungary

[b] Department of Biomedical Engineering, University of Minnesota, Minneapolis, MN 55455-0215, USA

[*] Contributed equally

[1] To whom correspondence may be addressed: csarkar@umn.edu

Supplementary Information

1. Extended Experimental Methods

2. Extended Computational Methods

3. Supplementary Figures and Tables

Supplementary Fig. 1: Expanded *MVsim* input features

Supplementary Fig. 2: Expanded *MVsim* output features

Supplementary Fig. 3: *MVsim* parameter variation identifies binding response sensitivities

Supplementary Fig. 4: Mechanistic insights into multispecific and multi-ligand interactions through *MVsim* microstate analysis

Supplementary Fig. 5. *MVsim* input parameters used in Fig. 3

Supplementary Fig. 6: Design-build-test optimization of three-input AND gates

Supplementary Fig. 7: *MVsim* illustrates the use of multivalent interactions in the temporal coding of sequential ligand interactions

Supplementary Fig. 8: Parameterization of *MVsim* to simulate the conformational dynamics, ACE2-RBD binding, and therapeutic neutralization of the SARS-CoV-2 S protein

Supplementary Fig. 9: Conformational rates of RBD switching are diagnostically captured in the multiphasic SPR binding responses

Supplementary Fig. 10: Simulating the effects of altered rate constants of RBD-switching on the S protein conformational ensemble and comparative binding to a neutralizing therapeutic or ACE2

Supplementary Table 1: Protein sequences of multivalent receptors and ligands used in this study

4. *MVsim* User Tutorial

5. Supplementary References

1. Extended Experimental Methods

Multivalent protein design and surface plasmon resonance (SPR) experiments

1.1 Multivalent protein design

The receptor protein-protein interaction domains that were used in our experimental binding studies (Fig. 3) derive from the C-terminal SH3 domain of the human adaptor protein Gads (1) and the synthetic DARPin Pdar (2). As the cognate ligands to these two receptor binding domains, we used the Gads SH3-binding peptide sequence (SPAPSIDRSTKPPL) derived from the Gads ligand, SLP-76 (1), and the designed ligand for the Pdar DARPin, Prb (2). DNA sequences of the receptors and ligands were synthesized as gBlocks (Integrated DNA Technologies). SH3 and DARPin receptors were introduced into His$_6$-tag-encoding pET28a (Novagen) and SLP-76 peptide and Prb ligands into maltose binding protein (MBP)-tag-encoding pMal-c5x (New England Biolabs (NEB)) expression vectors with standard DNA cloning methods. Multiple cloning sites in the expression vectors were used to create multivalent proteins with binding domains connected by peptide linkers (described below).

Molecular weight differences between the multivalent ligand analytes would cause measurable differences in signal amplitude (i.e., due to SPR being a molecular mass-based detection method), and possible differences in molecular diffusion and transport. In addition to the preparation of all multivalent peptide ligands as MBP fusions, lower valency ligands (i.e., bivalent, monovalent, and non-binding controls) were engineered from the trivalent ligand through incorporation of two alanine substitutions into either one, two, or all three of the SLP-76 peptide elements (i.e., SPAPSID**A**ST**A**PPL).

The interdomain polypeptide linkers used for both receptors and ligands were designed as either "short" or "long", and "flexible" (i.e., random coil) or "rigid" (i.e., alpha-helical). The following sequences were used:

short flexible: GSTSGDNSNSGGSGNSGGSGGN

long flexible: GSTGGDNSNSGGSGNSGGSGGNSGSTSGDNSNSGGSGNSGGASGN

short rigid: SPAEAAAKEAAAKEAAAKEAAAKAPS

long rigid: SPAEAAAKEAAAKEAAAKEAAAKEAAAKEAAAKEAAAKEAAAKAPSG

Recombinant expression of the mulitvalent receptors and ligands were performed in *Escherichia coli* BL21 cells transformed with the receptor and ligand expression vectors described above. For the purposes of immobilization for SPR, pET28a-receptor plasmids include the insertion of an N-terminal biotinylation tag (Avidity AviTag). AviTagged receptors were biotinylated by co-transformation of BL21 with GST-BirA. Protein expression was induced with 0.5 mM IPTG followed by overnight shaking and incubation at 17°C. Proteins were purified by Talon affinity (Clontech) for His$_6$-tagged receptors or amylose resin (NEB) for MBP-tagged ligands, followed by size exclusion chromatography (Hiload Superdex S200; GE Life Sciences) and buffer exchange by dialysis or desalting column (ThermoFisher).

1.2 Surface plasmon resonance (SPR)

1.2.1 SPR instrumentation

SPR experiments were performed at 25°C on a Biacore S200 instrument (GE Life Sciences). Running buffer HBS-EP+ (10 mM HEPES, 150 mM NaCl, 3 mM EDTA, 0.05% Tween-20, pH 7.4) was used for all immobilizations and binding experiments.

1.2.2 Immobilization of biotinylated receptors

Biacore CM5 sensor chip flow cells were preconditioned as follows: two 12 s injections of each of 50 mM NaOH, 20 mM HCl, and 0.1% w/v SDS. NeutrAvidin immobilized surfaces were generated on all flow cells as follows: 100 µg/ml NeutrAvidin (ThermoFisher) was prepared in 10 mM sodium acetate, pH 4.5; the amine coupling kit (GE Life Sciences) was used to activate the CM5 chip surfaces with the immobilization software protocol set to immobilize 2500 RU NeutrAvidin on each of the four flow cells; following NeutrAvidin immobilization, flow cells were quenched with 100 mM ethanolamine in 150 mM borate, pH 8.5.

1.2.3 Kinetic assays

Kinetic assays were performed in HBS-EP+ running buffer. Biotinylated receptors were prepared at concentrations ranging from 50-500 ng/mL in running buffer and delivered to the NeutrAvidin surface at 10 µl/min to achieve approximately 30 RUs of immobilized receptor density. To reduce experimental variation, monovalent, bivalent, and trivalent receptors were generally immobilized in series to adjacent flow cells (FC), FC2, FC3, and FC4, respectively. NeutrAvidin-coated FC1 was used as the reference flow cell and experiments were conducted with buffer blanks run at least in triplicate for double referencing. Ligands were prepared in 10 mM stock solutions and serially diluted across desired concentration ranges in HBS-EP+. Concentration series duplicates were performed to assess cycle-to-cycle variation. To minimize the occurrence of injection spikes and mass transfer phenomena (described further below) all experiments were performed at high flow rates of 75 µl/min.

1.2.4 Treatment of mass-transfer phenomena

To minimize complicating our engineered valency-driven avidity enhancements with mass-transfer phenomena (3, 4), several steps were taken. First, interacting protein pairs were chosen with modest association rate constants (k_{on}) and molecular weights. Further, all SPR experiments were intentionally performed with low immobilized surface density (e.g., less than 30 RUs of immobilized receptors with molecular weights of ~50 kDa). Additionally, diagnostic assessments of possible mass-transfer phenomena were performed by varying flow rates (20-100 µl/min); minimal changes to the kinetic traces were observed, but we conservatively still used high flow rates (75 µl/min). Finally, it was ensured that good fits were achieved with the “rapid mixing” 1:1 Langmuir model in the Biacore software for monovalent interactions. This indicated to us that the measured kinetic rate constants in Fig. 3a, which were used to parameterize the simulations in Fig. 3c,e,h, were not significantly affected by mass-transfer limitations. Further as stated above (section 1.1), the multivalent protein design approach sought to avoid increases in molecular weight with increasing valency.

These experimental measures were taken to ensure that the primary source of valency-driven alterations to binding avidity, affinity, and kinetics derived from the reduction in the apparent k_{off} due to the intra-complex statistical rebinding phenomena that is implicit to both multivalency and the network model of multivalency around which *MVsim* is constructed.

1.2.5 SPR data analysis

Experimental data for the monovalent receptor-ligand interaction were fit with a 1:1 Langmuir model for kinetic fits, and the equilibrium analysis model for RU_{max} and equilibrium dissociation constant fits.

2. Extended Computational Methods

The basic framework of *MVsim* is based on state-space representation with gray-box modeling where the input and output of the model are measures proportional to the concentration of certain receptor-ligand conformations. *MVsim* generates the model structure based on rules determined by the experimental setup then estimates the model parameters based on simple experimental measurements and binding probability estimation.

Model structure

In a state-space representation of multivalent receptor (R)-ligand (L) binding (5), the state vector x(t) represents the concentrations of the different receptor-ligand conformations, and the state (or system) matrix **A**(t) contains the free ligand concentrations and rate constants. The output vector y(t) is a derived measure of the state concentrations. In surface plasmon resonance (SPR) experiments, y(t) is the product of matrix **C**, containing an appropriate SPR scaling constant times the molecular weights of the relevant states, and state vector x(t). Due to the inability to directly influence the state concentrations, there is no input vector in the model. This representation is equivalent to a system of kinetic reaction equations in vector-matrix form:

$$[R] \leftrightarrow -kon * [R] * [L] + koff * [RL]$$
$$[RL] \leftrightarrow +kon * [R] * [L] - koff * [RL]$$
$$\frac{d\begin{bmatrix} R \\ RL \end{bmatrix}}{dt} = \begin{bmatrix} -kon * L & +koff \\ +kon * L & -koff \end{bmatrix} \begin{bmatrix} R \\ RL \end{bmatrix}$$
$$\frac{dx(t)}{dt} = A(t) * x(t)$$
$$y(t) = C * x(t)$$

The corresponding MATLAB code representation of the kinetic equations generated based on the experimental setup using unique rate constant identifiers is as follows:

```
dydt = zeros(2,1);

dydt(1)=+Koffs.Koff_Receptor1_pos_1_Ligand1_pos_1*y(2)-
L1*Kons.Kon_Receptor1_pos_1_Ligand1_pos_1*y(1);

dydt(2)=+L1*Kons.Kon_Receptor1_pos_1_Ligand1_pos_1*y(1)-
Koffs.Koff_Receptor1_pos_1_Ligand1_pos_1*y(2);
```

2.1 State vector

The state vector contains all possible conformations that can form from binding of receptor and ligand(s). The framework can model complex multivalent binding described by the following rules:

1) binding sites of the receptor(s)

2) binding sites of the ligand(s)
3) rules excluding binding between certain receptor-ligand binding sites
4) rules describing impossible conformations (e.g., due to steric blocking)

MVsim accommodates all models with the following rules: (1) one up to trivalent receptor, (2) up to three trivalent ligands, (3) receptor-site to ligand-site environment to specify exclusions between binding sites, and (4) allowing multiple binding events from the same type of ligand to the receptor.

These rules are phrased using unique identifiers for all states from the perspective of a receptor without crosslinking, with a three-digit code for each receptor binding domain. These three digits are as follows: (1) the identifier of the ligand, (2) the number of the ligand with this identifier that is bound to the same receptor, and (3) the number of the bound ligand binding domain. For example, Ligand A (i.e., '1'), first copy, with second binding site bound is identified as 112. This is applied to all receptor positions, with 000 used as the notation for an unbound receptor binding domain. For example, the 9-digit-long identifier 111 000 113 is the notation that represents a microstate interaction between a single trivalent ligand bound bivalently via its first and third domains to the first and third domains, respectively, of a trivalent receptor. Using these identifiers, all enumerated states comprise the state vector x(t).

2.2 State matrix

The state matrix **A**(t) is populated following the logic that, if two states have the same identifiers except one of the triplets is 000 in one of the states, then the transition can happen (e.g., 111 000 113 to 111 112 113). All of these transitions can be described with three constants:

1) If the transition is a dissociation (the number of 000s increases by one in the identifier), then the transition is described by a k_{off} constant.
2) If the transition is an association (the number of 000s decreases by one in the identifier), then the transition is described by a k_{on} constant multiplied by the ligand concentration.
    a. If the ligand is already bound to the receptor, this ligand concentration is an effective concentration estimated by the model.
    b. If the ligand is not yet bound to the receptor, this concentration is the bulk ligand concentration.

In SPR, both the ligand and effective concentrations are constant, but the model is capable of handling time-dependent changes in these concentrations if needed for the experimental setup. This is implemented in *MVsim* by changing the ligand concentrations at any point in the system.

The overall model structure of *MVsim* is based on the principles of chemical kinetics, and it does not introduce further biases or errors.

Model parameters

The model uses four types of parameters: association rate constant, k_{on}; dissociation rate constant, k_{off}; ligand concentration, [*L*]; and effective ligand concentrations, [L_{eff}].

The values for the association and dissociation rate constants are obtained from experiments with monovalent ligand and monovalent receptor (either in the literature or performed by the

researcher). The ligand concentration is typically constant in a given phase of the experimental setup (nonzero during association and zero during dissociation), and SPR results are usually presented as a time-dependent plot of output (response units), often with an overlay of response curves from multiple ligand concentrations.

The last parameter is the effective ligand concentration, which can be crudely estimated from the radius of the receptor and ligand linkers, but which is computed more accurately by *MVsim* using a probabilistic approach that incorporates detailed biophysical properties of the interacting molecules.

2.3 Estimation of effective ligand concentrations

From a probabilistic point of view, the effective ligand concentration is the concentration of a free monovalent ligand that has the same probability of binding to a free site on a partially bound receptor as the already partially bound multivalent ligand binding to the same free site on the same receptor.

To model the effective concentration, we use a probability density function (PDF) of the end-to-end distance, which in our case is the distance between the occupied binding site and the free binding site. PDF(r) for ligand and receptor describes the probability that the two ends are a distance r apart.

2.3.1 Binding probability

For calculating a binding probability, we need PDF(x,y,z), a three-dimensional probability density function. The binding probability is the integral of the product of the ligand and receptor PDFs over the volume.

In the simplest case, the PDF is a uniform distribution of one molecule in a given volume. In the case of free ligand, we have a uniform distribution with a volume occupied on average by one molecule; in the case of a 1 M solution, the volume is $1/(6\times10^{23})$ dm^3.

MVsim uses more complex PDFs to model the receptor and ligand, based on literature on linker PDFs and an iterative process to calculate PDFs for joint linkers or linkers and spherical units. These derived PDFs can be used in a convolution integral to calculate the probability of binding and, by comparing it to that of the free ligand, we obtain effective concentrations.

2.3.1.1 Ligand and receptor PDFs

For the ligand and receptor PDFs, *MVsim* uses an iterative process which calculates the joint PDFs of linkers and binding domains. For example, a linear trivalent receptor molecule contains a binding domain, a linker, a second binding domain, a second linker, and a third binding domain. Therefore, a trivalent receptor is described by the joint PDF of these five components.

Since we are interested in the position of the two ends regardless of the positions of any intervening joining points of the components, we can integrate all the joining points leading to the following integral (see sections 2.5 and 2.6 for additional details):

$$(f * g)(x_2, y_2, z_2) = \int_{-l}^{l}\int_{-l}^{l}\int_{-l}^{l} f\,(x, y, z) * g(x_2 - x, y_2 - y, y_2 - z)dxdydz$$

The iterative MATLAB implementation of the joint PDF is the following, where either a binding

domain or a linker is added to the PDF:

```
fr = @(r0, fi0,r) f_root(r0).*f_toadd(sqrt(r.^2+r0.^2-
2.*r.*r0.*cos(fi0))).*r0.^2.*sin(fi0);

y(i) = quad2d(@(r0, fi0) fr(r0,fi0,x(i)) ,r0_min,r0_max,0, fi_max, 'AbsTol',
1e-10,'RelTol', 1e-4, 'MaxFunEvals', 20000);
```

2.3.1.2 Calculating effective ligand concentration from binding probability

The equation for converting the multivalent binding probability to an effective concentration is as follows (see section 2.4 for details):

$$C_{eff} = \frac{\int PDF_{ligand}(V) * PDF_{receptor}(V)dV}{constant_{PDF\ normalisation}}$$

which is implemented in *MVsim* with the following code:

```
final_conv =  @(r,z)
f_Receptor(sqrt(r.^2+z.^2)).*f_Ligand(sqrt(r.^2+(z+shift).^2)).*r;

value = 2*pi*integral2(final_conv, 0,min(length_Receptor, length_Ligand), -
min(length_Receptor, length_Ligand,min(length_Receptor, length_Ligand);

Ceff = value/(c_norm_Receptor*c_norm_Ligand)/(6*(10^-4));
```

2.3.1.3 Solving integrals for the binding probability

To determine binding probabilities in *MVsim*, we face the practical problem of computing triple integrals with complex or arbitrary functions, which are infeasible to solve over a reasonable time period. We therefore rewrite all integrals in polar coordinates using the spherically symmetric nature of the PDF, resulting in more tractable double integrals (see sections 2.6 and 2.7 for details).

Output

The y(t) output vector and the **C** output matrix convert the microstate concentrations to the desired output format.

In the case of SPR, y(t) has only one element, the output in RU, which is proportional to the molecular weight(s) of the bound ligands. To get this output, C is a one-dimensional matrix with the sum of the molecular weights of the ligands bound to the receptor times the volume of the SPR chip times the conversion constant from grams to picograms.

MVsim also provides additional output options. The output can be in the form of molar concentration or weight/RU and y(t) can contain all the states or the sum of states based on their

binding valency or the type of the bound ligands.

The model outputs are presented in conventional time-dependent plots as well as in a graphical representation of the microstates that sheds light on the species driving noncanonical binding dynamics in multivalent reactions.

Mathematical details of the model

2.4 Calculating effective concentrations

The effective molecule number (Eff_{num}) is the number of monovalent ligands that are equally probable to bind a free binding domain on a multivalent receptor as the probability of an already-receptor-bound multivalent ligand binding with another one of its domains to the same free binding domain on the receptor:

$$Eff_{num} * P(uniform\ ligand\ position = receptor\ free\ end\ position) \\ = P(ligand\ free\ end\ position = receptor\ free\ end\ position)$$

$$Eff_{num} = \frac{P(ligand\ free\ end\ position = receptor\ free\ end\ position)}{P(uniform\ ligand\ position = receptor\ free\ end\ position)}$$

To get the probability of the two ends meeting, we can examine all points in space, note the probability of the ends meeting at each point, and integrate over all these values to get the overall probability in question.

$$P(ligand\ free\ end\ position = receptor\ free\ end\ position) \\ = \int PDF_{ligand}(V) * PDF_{receptor}(V)dV$$

and

$$P(uniform\ ligand\ position = receptor\ free\ end\ position) \\ = \int 1 * constant_{PDF\ normalisation} * PDF_{receptor}(V)dV \\ = constant_{PDF\ normalisation} * \int PDF_{receptor}(V)dV = constant_{PDF\ normalisation}$$

Therefore, the effective number of ligands can be calculated by:

$$Eff_{num} = \frac{\int PDF_{ligand}(V) * PDF_{receptor}(V)dV}{constant_{PDF\ normalisation}}$$

2.4.1 Effective ligand concentration

We can convert the effective number of ligands to an effective ligand concentration by dividing this ligand number by Avogadro's number and the accessible volume.

2.5 PDF for linkers and binding domains

2.5.1 Linker PDF

The PDF for the ligand end-to-end distance in terms of contour length (L_c) and persistence length

(L_p) is (6,7):

$$PDF(r, L_p, L_c) \propto \frac{1}{\left(1-\frac{r^2}{L_c^2}\right)^{\frac{9}{2}}} e^{\left(-\frac{9L_c}{8L_p}\frac{1}{1-\frac{r^2}{L_c^2}}\right)}$$

or

$$PDF(x, y, z, L_p, L_c) \propto \frac{1}{\left(1-\frac{x^2+y^2+z^2}{L_c^2}\right)^{\frac{9}{2}}} e^{\left(-\frac{9L_c}{8L_p}\frac{1}{1-\frac{x^2+y^2+z^2}{L_c^2}}\right)}$$

2.5.2 Binding domain PDF

The PDF of the binding domain is based on its diameter (d) as follows:

$$PDF(r, d) \propto \{if\ d-\epsilon \le r \le d+\epsilon = 1, otherwise = 0\}$$

or

$$PDF(x, y, z, d) \propto \{if (d-\epsilon)^2 \le x^2+y^2+z^2 \le (d+\epsilon)^2 = 1, otherwise = 0\}$$

where ϵ represents the uncertainty related to the domain diameter (e.g., standard deviation of its measurement).

2.6 Convolution of two PDFs

The base equation of convolution is:

$$(f * g)(t) = \int_{-\infty}^{\infty} f(\tau) g(t-\tau)\, d\tau$$

The same equation using (x,y,z) coordinates and starting from a non-zero point is:

$$(f * g)(x_0, y_0, y_0, x_2, y_2, z_2) = \int_{-l}^{l}\int_{-l}^{l}\int_{-l}^{l} f(x-x_0, y-y_0, z-z_0) * g(x_2-x, y_2-y, z_2-z) dxdydz$$

If $x_0 = 0, y_0 = 0, z_0 = 0$, this reduces to:

$$(f * g)(x_2, y_2, z_2) = \int_{-l}^{l}\int_{-l}^{l}\int_{-l}^{l} f(x, y, z) * g(x_2-x, y_2-y, y_2-z) dxdydz$$

And switching to spherical coordinates:

$$(f * g)(R \mid r_0) = \int_0^{2\pi}\int_0^{\pi}\int_0^{r_{max}} f(\| \hat{r} - \hat{r_0} \|) * g(\| \hat{R} - \hat{r} \|) r^2 sin\phi dr d\phi d\theta$$

$$x = r\,sin(\phi)cos(\theta), y = rsin(\phi)sin(\theta), z = rcos(\phi)$$

$$\| \hat{R} - \hat{r} \| = \sqrt{R^2 + r^2 - 2Rr[sin(\phi_R)sin(\phi)cos(\theta_R)cos(\theta) + sin(\phi_R)sin(\phi)sin(\theta_R)sin(\theta) + cos(\phi_R)cos(\phi)]}$$

Setting ϕ_R to zero, we get:

$$\| \hat{R} - \hat{r} \| = \sqrt{R^2 + r^2 - 2Rr[0 * sin(\phi)cos(\theta_R)cos(\theta) + 0 * sin(\phi)sin(\theta_R)sin(\theta) + 1 * cos(\phi)]}$$

$$\Longrightarrow \| \hat{R} - \hat{r} \| = \sqrt{R^2 + r^2 - 2Rrcos(\phi)}$$

where r is the linker end-to-end distance, R – r is the hinge-to-binding site distance in the domain, and R is the point where we are interested in the probability of the PDF:

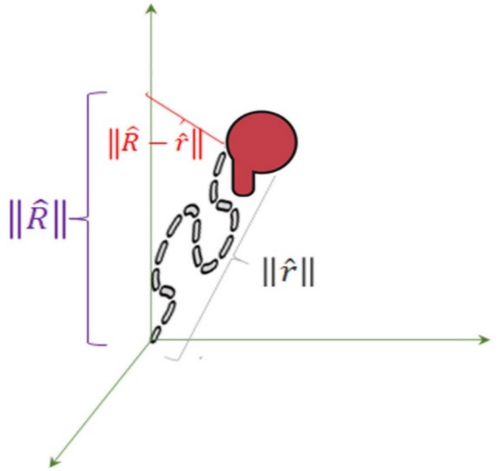

If r_0 is zero and we position R to fall to the z-axis, then ϕ_R is zero, and we get the following function for PDF(R):

$$(f * g)(R) = \int_0^{2\pi} \int_0^{\pi} \int_0^{r_{max}} f(r) * g\left(\sqrt{R^2 + r^2 - 2Rrcos\phi}\right) r^2 sin\phi drd\phi d\theta$$

$$(f * g)(R) = \int_0^{2\pi} 1\, d\theta * \int_0^{\pi} \int_0^{r_{max}} f(r) * g\left(\sqrt{R^2 + r^2 - 2Rrcos\phi}\right) r^2 sin\phi drd\phi$$

$$(f * g)(R) = 2\pi \int_0^{\pi} \int_0^{r_{max}} f(r) * g\left(\sqrt{R^2 + r^2 - 2Rrcos\phi}\right) r^2 sin\phi drd\phi$$

2.7 Convolution with actual functions

Let us consider a domain-linker-domain construct starting at the origin (r_0 = 0), with the first domain ending at distance r_1, the linker ending at distance r_2, and the second domain ending at distance r_3. We can further define R as the endpoint of the entire construct of interest (here, equal to r_3) and also define r as any intermediate endpoint of interest (here, r_1 or r_2).

The convolution of two functions corresponding to the first domain and the linker is therefore given by:

$$(f_{domain,1} * g_{linker})(r_2 \mid r_0 = 0)$$
$$= 2\pi \int_0^{\pi} \int_0^{r_{max}} f_{domain,1}(r_1) * g_{linker}\left(\sqrt{r_2^2 + r_1^2 - 2r_2 r_1 cos\phi_1}\right) r_1^2 sin\phi_1 dr_1 d\phi_1$$

And the convolution of three functions, corresponding to the full domain-linker-domain construct, is:

$$(f_{domain,1} * g_{linker} * f_{domain,2})(R \mid r_0 = 0)$$
$$= 2\pi \int_0^{\pi} \int_0^{r_{max}} \left(2\pi \int_0^{\pi} \int_0^{r_{max}} f_{domain,1}(r_1) * g_{linker}\left(\sqrt{r_2^2 + r_1^2 - 2r_2 r_1 cos\phi_1}\right) r_1^2 sin\phi_1 dr_1 d\phi_1\right) * f_{domain,2}\left(\sqrt{R^2 + r_2^2 - 2Rr_2 cos\phi_2}\right) r_2^2 sin\phi_2 dr_2 d\phi_2$$

Since the two-function convolution only depends on one parameter, r_2, the value of this convolution can be fit to a new function, f_{root}, to reduce the three-function convolution to a two-function convolution of f_{root} and the remaining function, f_{to_add}. In the present example, this would be written as:

$$(f_{domain,1} * g_{linker} * f_{domain,2})(R \mid r_0 = 0)$$
$$= 2\pi \int_0^{\pi} \int_0^{r_{max}} f_{root}(r_2) * f_{to\_add}(\| \hat{R} - \hat{r_2} \|) r_2^2 sin\phi dr_2 d\phi$$

where $f_{root} = (f_{domain,1} * g_{linker})(r \mid r_0 = 0)$ and $f_{to\_add} = f_{domain,2}(\| \hat{R} - \hat{r} \|)$.

This is a more computationally efficient double integral and thus allows for faster calculation of convolution chains.

2.8 Effective ligand number with predicted PDFs

For calculating effective ligand number, we need the free-end to bound-end PDFs for the ligand and the receptor, and we can predict their non-normalized PDFs using the technique described in section 2.7.

Therefore, the equation for the effective number of ligands is:

$$Eff_{num} = \frac{\int C_L * f_{ligand}(V) * C_R * g_{receptor}(V) dV}{C_U}$$

where C_U, C_L, C_R are normalization constants for the uniformly distributed ligand, the bound ligand, and the bound receptor, respectively. And,

$$f_{ligand}(V) = L\left(\left(f_{L,domain,i} * g_{L,linker,i}\right)_{m-1} * f_{L,domain,m}\right)(r_m, \phi, \theta)$$

$$g_{receptor}(V) = R\left(\left(f_{R,domain,i} * g_{R,linker,i}\right)_{n-1} * f_{R,domain,n}\right)(r_n, \phi, \theta)$$

where m and n are the valencies of the ligand and receptor, respectively and $L(r_m, \phi, \theta)$ and

$R(r_n, \phi, \theta)$ are the entire ligand and receptor PDFs. The rank of the domains and linkers is i = 1 to m for the ligand and i = 1 to n for the receptor.

Together, this yields the equation

$$Eff_{num} = \frac{4\pi C_L C_R \int R\left(\left(f_{R,domain,i} * g_{R,linker,i}\right)_{n-1} * f_{R,domain,n}\right)(r_n = r) * L\left(\left(f_{L,domain,i} * g_{L,linker,i}\right)_{m-1} * f_{L,domain,m}\right)(r_m = r) r^2 dr}{C_U}$$

2.9 Further technical considerations

2.9.1 Integral bounds

Matching the integral bounds of a convolution of f_{root} and f_{to_add} to the input ranges of these functions significantly speeds up numerical integration. For PDF calculations using spherical integral convolution, the optimized bounds are:

θ: $[0,2\pi]$ (in our case, it integrates to 2π)

ϕ: if $R > L_{root}$, then the bounds are $\left[0, arcsin\left(\frac{L_{to\_add}}{R}\right)\right]$; otherwise, $[0,\pi]$

r: $\left[max\left(0, R - L_{to\_add}\right), min\left(L_{root}, R + L_{to\_add}\right)\right]$

where L_{root} is the maximum of the input range of the first function (f_{root}), L_{to_add} is the maximum of the input range of the second function (f_{to_add}), and R is the position at which we want to evaluate the integrals.

If a binding domain is added, the bounds of r are $[d - \epsilon, d + \epsilon]$.

2.9.2 Flipping the origin

Due to the commutative nature of the convolution, the following applies:

$$(f * g)(t) := \int_{-\infty}^{\infty} f(\tau) g(t - \tau)\, d\tau = \int_{-\infty}^{\infty} f(t - \tau) g(t)\, d\tau$$

Numerically integrating the convolution generally leads to rapid calculations. However, under certain conditions (e.g., when the domains of the two functions are of different orders of magnitude and thus large regions of the sampled space have zero values), it takes significant time for the algorithm to converge. In these cases, it is beneficial to use the function with the smaller coordinate domain as $f(\tau)$. It does not change the outcome, but can greatly reduce the computational time.

2.9.3 Spatial shift

For the final convolution, the PDFs of the ligand and the receptor can start from the same origin, the center of the binding site. However, in cases where two domains are not binding in an inline configuration but in a twisted configuration, it is computationally useful to introduce a spatial shift to compute the final convolution since the twisted binding configuration introduces complex angular restrictions due to the shifted relative positioning of the domain-linker hinges. The magnitude of the shift parameter is determined by the diameters of the receptor and ligand binding

domains. In these cases, the convolution changes from $Eff_{num} = \frac{C_L * C_R \int\int\int R(r) * L(r) r^2 \sin(\phi) dr d\phi d\theta}{C_U}$ to $Eff_{num} = \frac{C_L * C_R * 4\pi \int R(r) * L(r) r^2 dr}{C_U}$ in the case of no applied shift or to $Eff_{num} = \frac{C_L * C_R \int\int\int R(r) * L\left(\sqrt{shift^2 + r^2 - 2shift * rcos\phi}\right) r^2 sin(\phi) dr d\phi d\theta}{C_U}$ in the case of an applied shift. This translational shift is more easily and quickly applied in cylindrical coordinates along the z axis: $Eff_{num} = \frac{C_L * C_R * 2 * \pi \int\int R(\sqrt{r^2 + z^2}) * L(\sqrt{r^2 + (z + shift)^2} * r \, dr \, dz}{C_U}$.

2.10 Goodness of fit to experimental data

2.10.1 Calculating root-mean-square error (RMSE) between model and experiment

For quantifying the accuracy of the model, the experimental and simulated curves were globally compared by calculating the root-mean-square error (RMSE) between the experimental and simulated response units at each timepoint. Since the sampling of timepoints can result in varying RMSE values, the timepoints were either evenly sampled (e.g., for SPR data) or selected by the ode15s solver in MATLAB. The difference between these two RMSE values was minimal, but the even-sampling approach was more sensitive to the overall curves whereas the method using ode15s timepoints was more sensitive to regions of the curves with the largest rates of change. The reported RMSE values are for even sampling due to its more general applicability, even though its RMSE values were slightly larger than for the approach using sampling from the stiff ODE solver. For raw SPR outputs, experiments could have signal artifacts at the injection time(s) and due to high-frequency noise. The injection artifacts were removed and a 10-Hz lowpass filter was applied to the data if the noise reached 0.1 RU.

2.10.2 Fitting routine to extract rate constants of S protein RBD conformational switching

For fitting k_{down} and k_{up} in Fig. 6d, the MATLAB Bayesian optimization algorithm *bayesopt* (https://www.mathworks.com/help/stats/bayesian-optimization-algorithm.html) was incorporated into the *MVsim* code. After data pre-processing, RMSE was used as the scalar objective function to be minimized in the algorithm in order to identify the optimal values of k_{down} and k_{up}. More generally, this optimization routine can be used in conjunction with *MVsim* to estimate any parameter in a simulation of interest.

3. Supplementary Figures and Tables

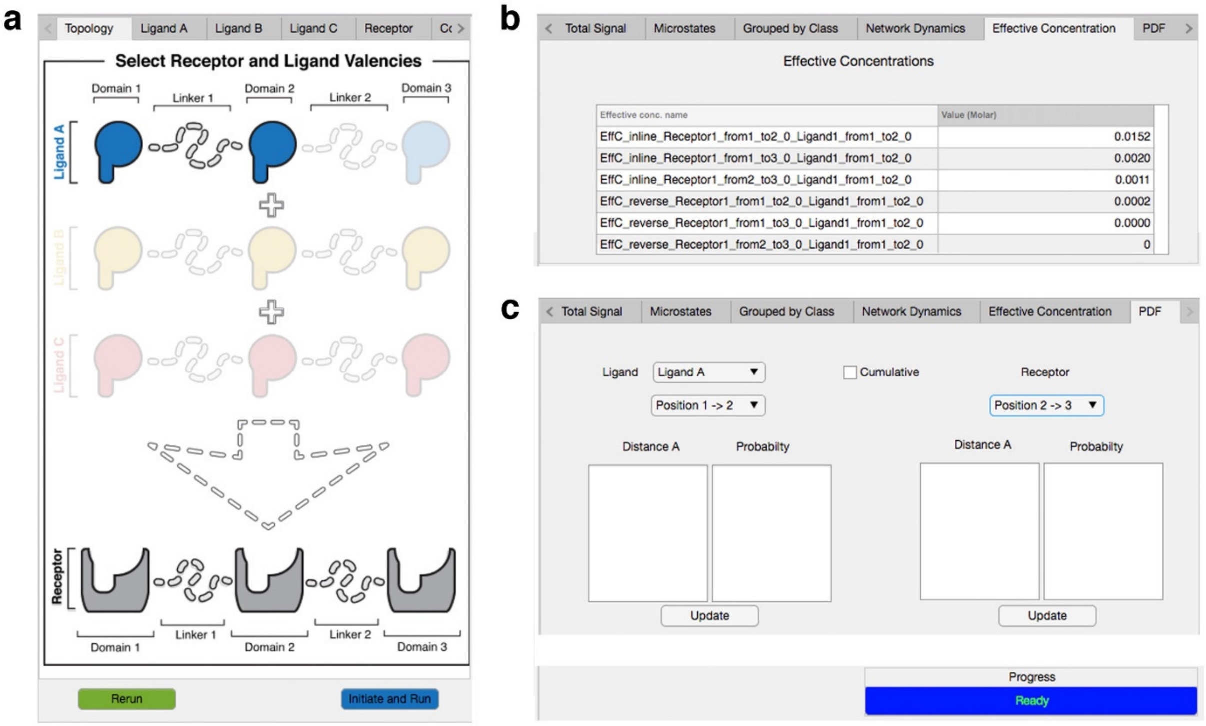

Supplementary Fig. 1: GUI Inputs. *MVsim* enables bypassing of the probability density and effective concentration calculations by directly specifying either a set of effective concentrations, [L_{eff}], or by uploading a set of probability density functions (PDFs) for a multivalent system of interest. a, An example of a multivalent system. Here, a bivalent ligand (blue) interacts with a trivalent receptor (grey). **b**, After initiating the simulation, and upon its completion, the "Effective Concentration" tab in the output window displays the name of each calculated [L_{eff}] (left column) and its calculated value in units of molar concentration (right column). The user can edit each [L_{eff}] value by clicking on corresponding field in the right column. The user-specified [L_{eff}] values are computed through use of the "Rerun" button (colored green; **a**, bottom left). **c**, The "PDF" tab in the output window features an interface through which PDFs can be crafted and applied to the user-specified multivalent system; this can be performed for any or all of the ligand(s) and the receptor, as well as for any or all of the [L_{eff}] values. To begin, the user selects the multivalent molecule they wish to edit from the "Ligand" drop-down menu. Next, for ligand and/or receptor, the user selects the positional distribution they wish to edit from the lower drop-down menu. Here, for example, the selection of "Ligand A" and "Position 1 $\rightarrow$ 2" allows the user to edit the PDF that describes the spatial distribution of the second binding domain relative to the first within the trivalent structure of Ligand A. Coordinates for the PDF are entered in the "Distance" (x-axis values measured in Angstroms) and "Probability" fields and are uploaded in place of the existing calculated values using the "Update" button. To perform simulations with updated PDFs, the "Rerun" button is used.

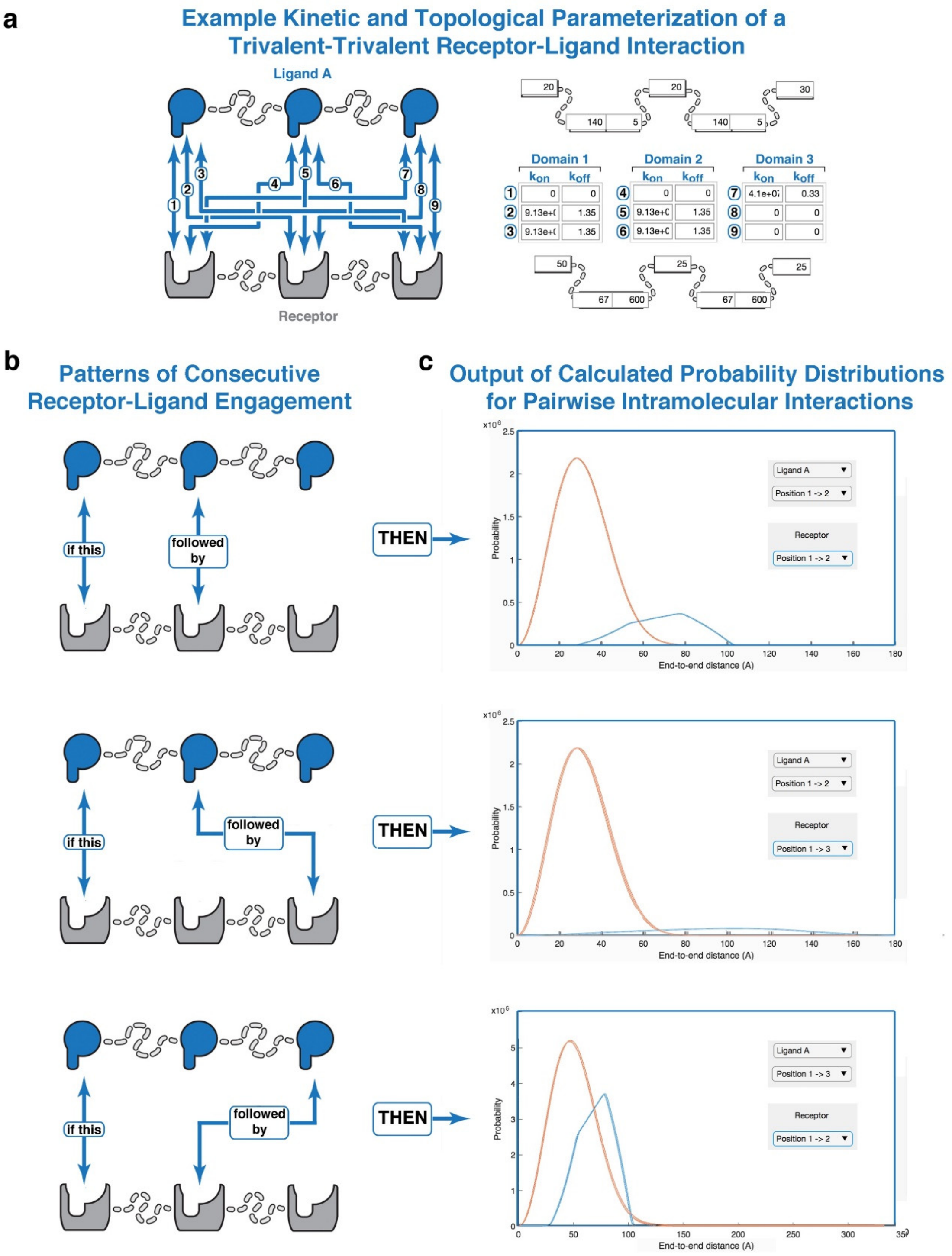

Supplementary Fig. 2: GUI Outputs. *MVsim* enables visualization of the computed set of probability density functions (PDFs) for a specified multivalent system. a, As an example, a trivalent-trivalent receptor-ligand interaction is selected (left panel) and parameterized with a set of kinetic rate constants and topological parameters (right panel). **b**, Effective ligand concentrations [L_{eff}] are derived from the spatial proximity between ligand and receptor binding domains that results from the tethering via a prior point of interaction. Here, three [L_{eff}] possibilities are shown. In each of the three examples, the initial "if this" interaction occurs between the first binding domain of the ligand and the first binding domain of the receptor. Subsequently, three of the four possible "followed by" binding events are depicted. **c**, For each of these three [L_{eff}] possibilities, one-dimensional representations of the PDFs are shown for each, consisting of both the domain-to-domain distributions for the ligand (orange PDF) and receptor (blue PDF).

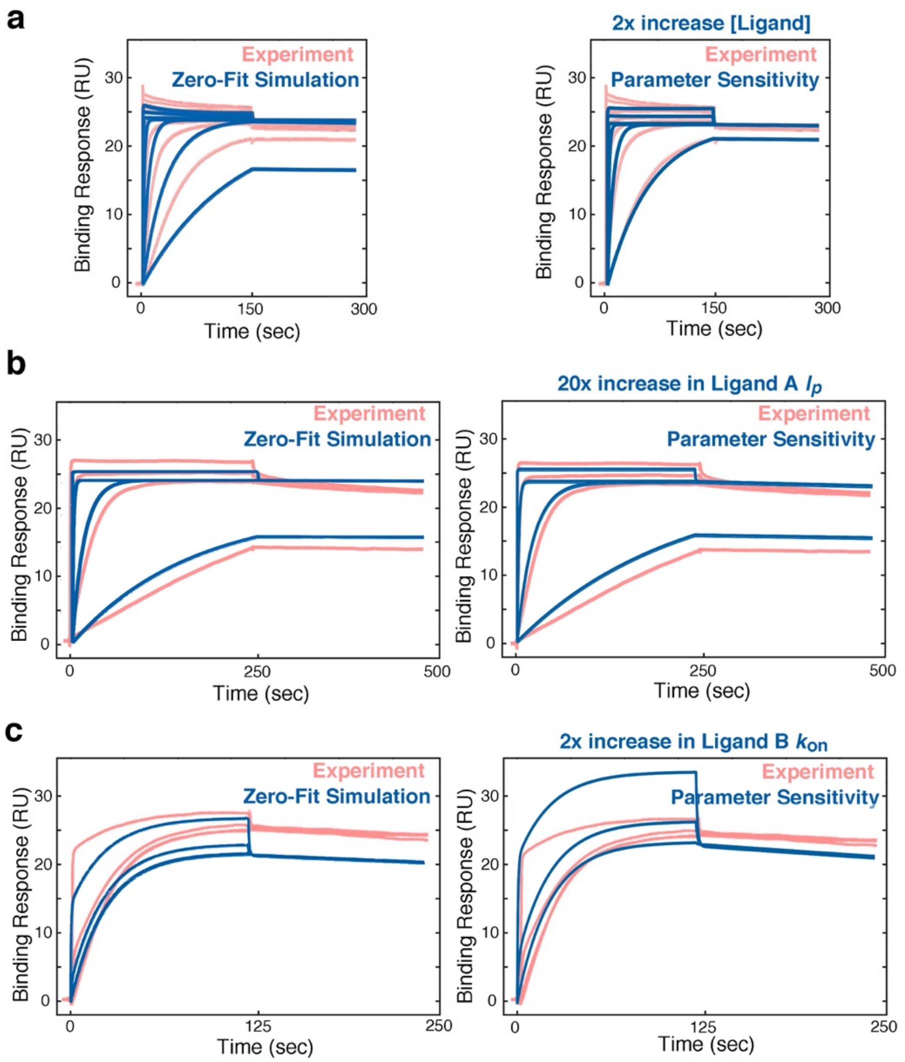

Supplementary Fig. 3: Parameter input variation shows deviations from the zero-fit simulations and identifies key sensitivities relating multivalent structure/kinetics and binding response. a, (related to Fig. 3c) The zero-fit simulations (left plot, blue traces) display good agreement with the experimental data (left plot, pink traces) with regard to the non-canonical, multiphasic features of the association and dissociation phases, with an overall RMSE of 2 RU (7% of the average binding response). A notable disparity between the simulation and the experiment is found in the reduced signal magnitude of the simulated binding responses, particularly at the lowest ligand concentrations. Small systematic increases in the simulated ligand concentration (up to a 2x increase; right plot, blue traces) led to a better overall agreement with the experiment, albeit with a less pronounced initial kinetic burst phase due to a transition from constrained trivalent states to high-stoichiometric states (right plot, red traces). **b,** (related to Fig. 3e,f) Similarly, good, quantitative model-simulation agreement was observed with the trivalent, bispecific interaction (left plot), with an RMSE of 1.5 RU (6% of the average binding response). Again, however, key points of disparity between simulation and experiment are evident. Here, a small under-representation of the biphasic character of the association phase and the rate of the dissociation phase are apparent. Here, systematic increases in the persistence length (l_p) of Ligand A served to slightly destabilize the trivalent interaction, leading to a modest improvement in the simulated representation of the experimental data (right plot). **c,** (related to Fig. 3h,i) The zero-fit simulation of the two-ligand interaction system was observed to offer the best agreement with the experiment by balancing its matching of the strongly biphasic association phase and the moderately biphasic dissociation phase (left plot) with an RMSE of 4 RU (18% of the average binding response). Here, no single parameter was found to be capable of improving the fits to both the association and dissociation phase. For example, a 2x increase in the k_{on} of Ligand B improved the agreement with the initial, fast phase of association in the experiment at the expense of properly capturing the relative proportions of the fast and slow phases of association and dissociation (right plot).

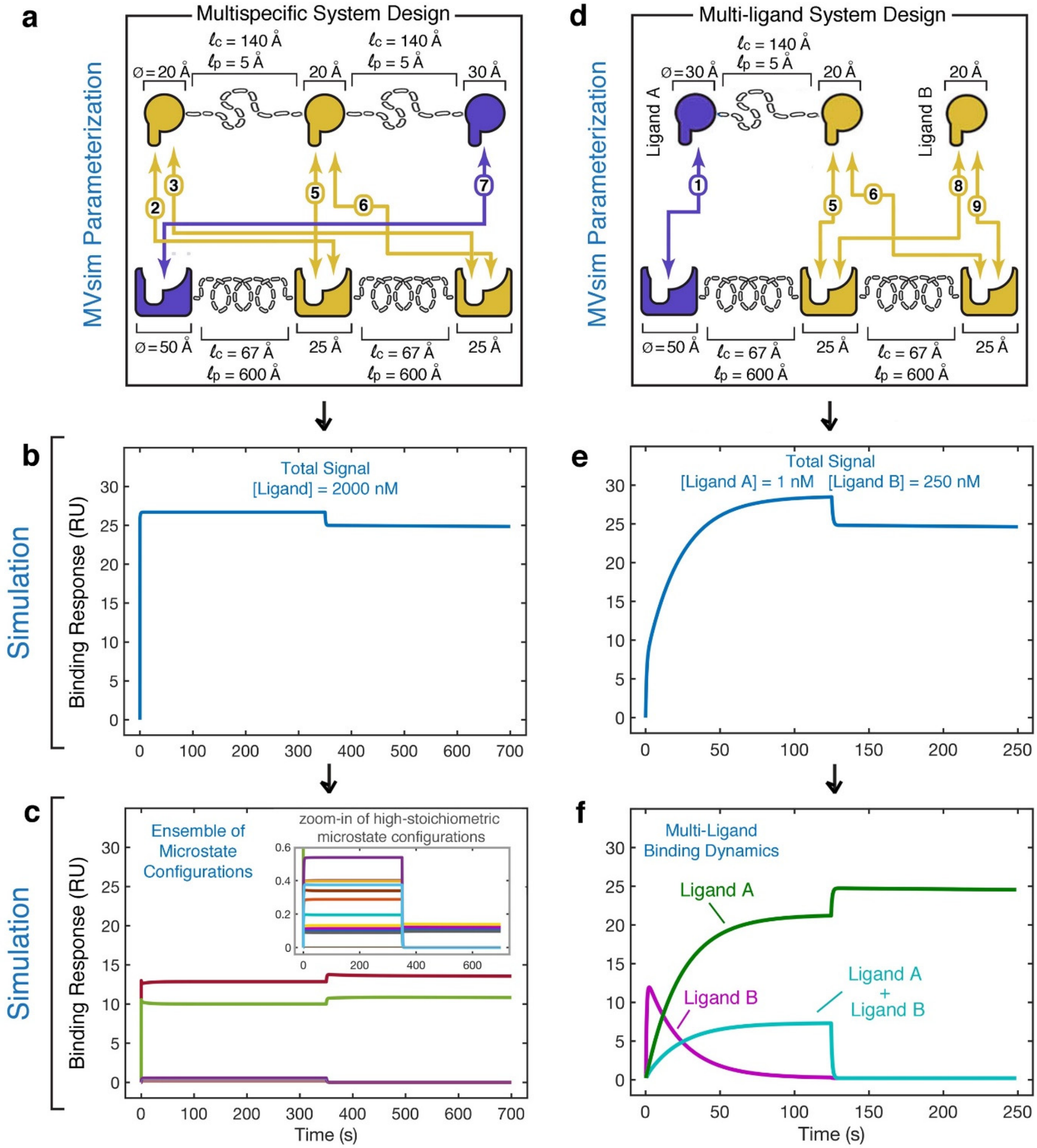

Supplementary Fig. 4: *MVsim* provides mechanistic insights into multivalent interactions by enabling visualization of the underlying configurational microstate dynamics. **a**, *Mvsim* parameterization used to predict and simulate the experimental multispecific binding response dynamics shown in Fig. 3e. **b**, Total signal from the simulated response dynamics with a ligand concentration of 2000 nM. **c**, The ensemble of microstates for the trace in (**b**) shows the respective contributions of the two trivalent configurations (colored dark red and green, in main plot) and the multitude of high-stoichiometric, mixed monovalent-bivalent configurations (zoomed inset) to the overall multiphasic binding response dynamics. **d**, *Mvsim* parameterization used to predict and simulate the experimental multi-ligand binding response dynamics shown in Fig. 3h. **e**, Total signal from the simulated response dynamics with a Ligand A concentration of 1 nM and a Ligand B concentration of 250 nM. **f**, The respective ligand-bound states for the trace in (**e**) show the respective contributions of the bivalent, higher-avidity/slower-associating Ligand A and fast on/off binding of the monovalent Ligand B towards the multiphasic binding response dynamics.

related to Figure 3c

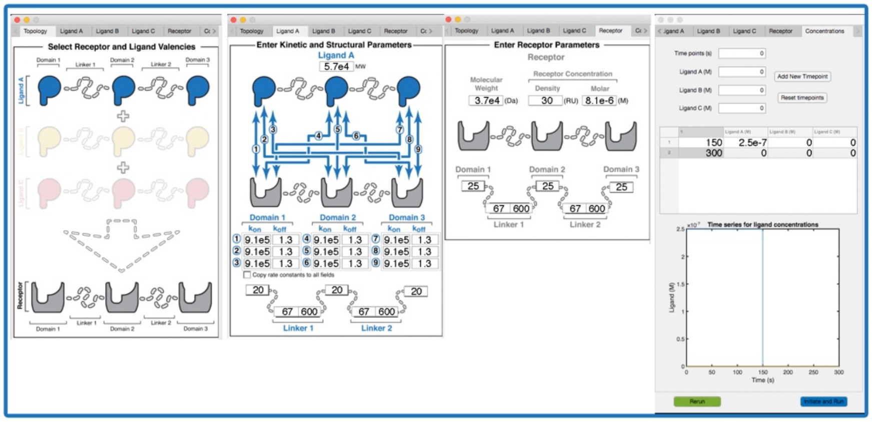

related to Figure 3e

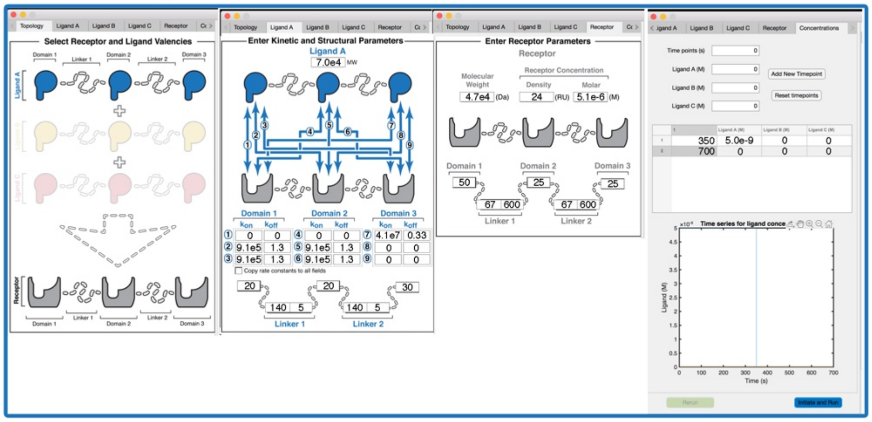

related to Figure 3h

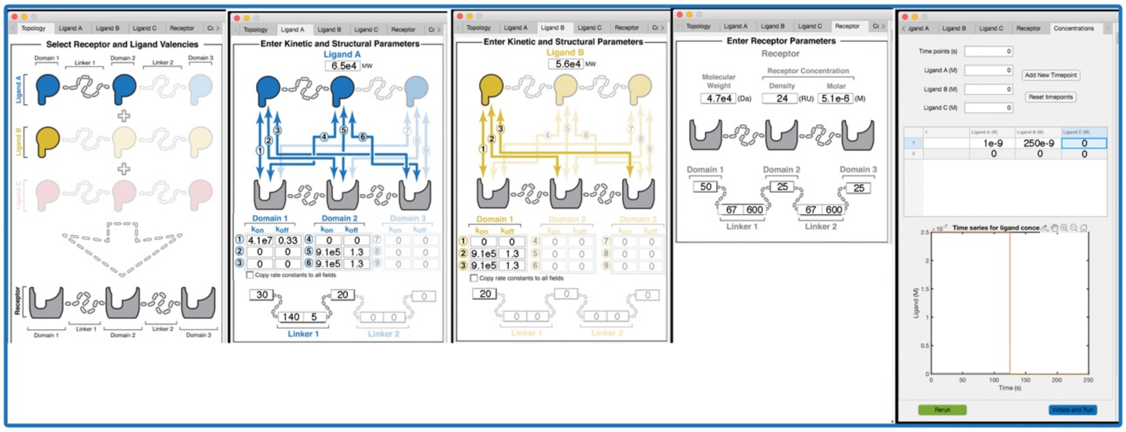

Supplementary Fig. 5. *MVsim* input parameters and representative output binding responses that were used to perform the simulations presented in Fig. 3. To facilitate the use of *MVsim* and reproduction of the simulations presented in Fig. 3, these screen captures detail the full parameterizations for each of the three simulated multivalent systems as directly entered into the *MVsim* graphical user interface.

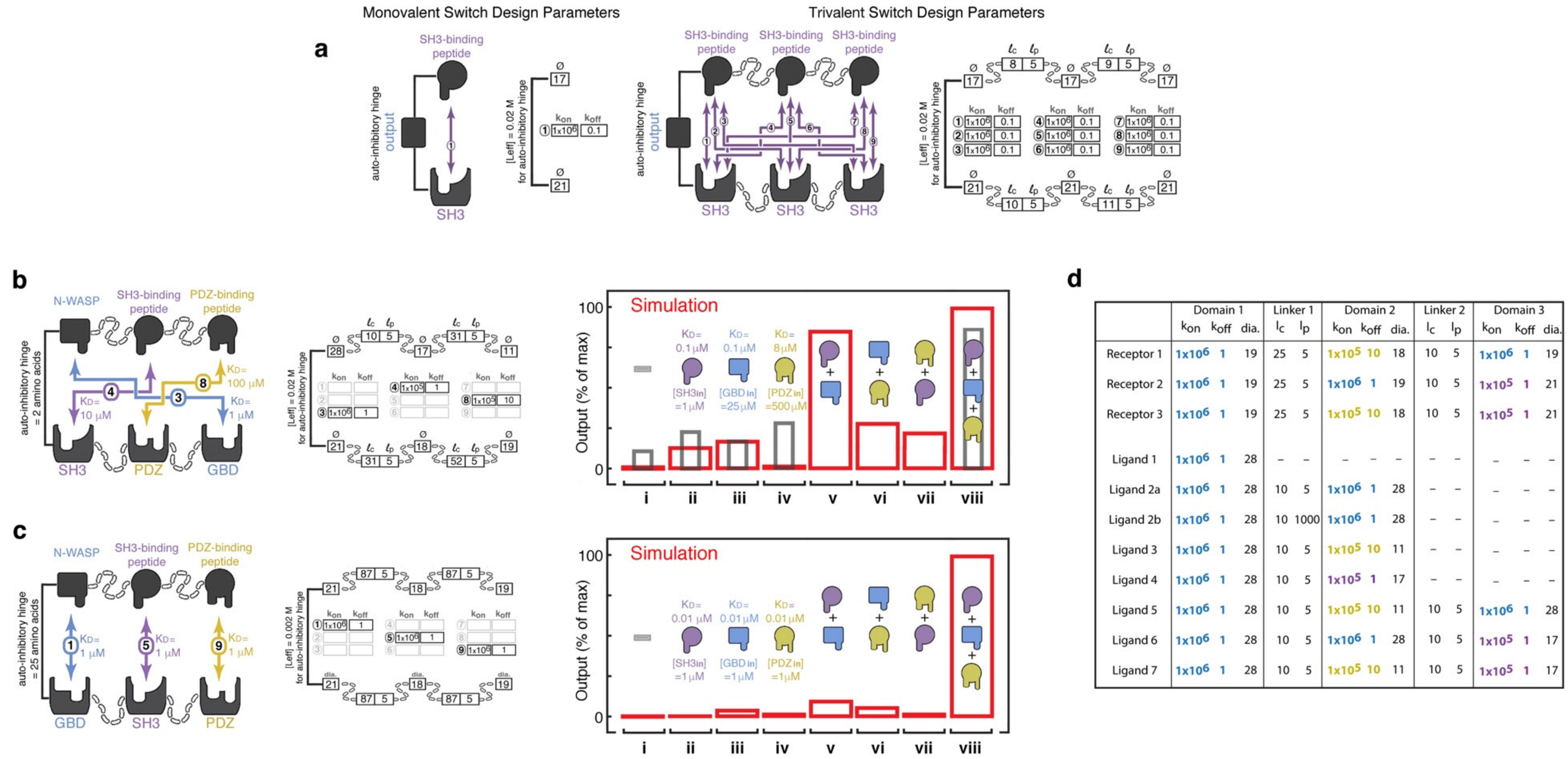

d

	Domain 1			Linker 1		Domain 2			Linker 2		Domain 3		
	k_{on}	k_{off}	dia.	l_c	l_p	k_{on}	k_{off}	dia.	l_c	l_p	k_{on}	k_{off}	dia.
Receptor 1	1×10^6	1	19	25	5	1×10^5	10	18	10	5	1×10^6	1	19
Receptor 2	1×10^6	1	19	25	5	1×10^6	1	19	10	5	1×10^5	1	21
Receptor 3	1×10^6	1	19	25	5	1×10^5	10	18	10	5	1×10^5	1	21
Ligand 1	1×10^6	1	28	–	–	–	–	–	–	–	–	–	–
Ligand 2a	1×10^6	1	28	10	5	1×10^6	1	28	–	–	–	–	–
Ligand 2b	1×10^6	1	28	10	1000	1×10^6	1	28	–	–	–	–	–
Ligand 3	1×10^6	1	28	10	5	1×10^5	10	11	–	–	–	–	–
Ligand 4	1×10^6	1	28	10	5	1×10^5	1	17	–	–	–	–	–
Ligand 5	1×10^6	1	28	10	5	1×10^5	10	11	10	5	1×10^6	1	28
Ligand 6	1×10^6	1	28	10	5	1×10^6	1	28	10	5	1×10^5	1	17
Ligand 7	1×10^6	1	28	10	5	1×10^5	10	11	10	5	1×10^5	1	17

Supplementary Fig. 6: *MVsim* guides the design, building, and testing of molecular logic gates for use in synthetic biology. a, The architectures and literature-derived (Dueber et al., *Nat. Biotechnol.*, 2007) parameterization of the monovalent and trivalent switches simulated in Fig. 4a. **b**, The architecture and literature-based parameterization of the three-input AND gate simulated in Fig. 4b (left and middle panels). Simulated performance (right panel, red bars) and experimental performance (right panel, grey bars) of the AND gate detailed in the presence of (from left to right): i, no input; ii, iii, iv, single inputs; v, vi, vii, dual inputs; and viii, triple input. Significant spurious activation of the AND gate's output is predicted for both single and dual inputs, particularly the dual input comprised of SH3 (purple) and GBD (blue) in bar v. Here, the indicated K_D and concentration values (right panel) refer to those of the input ligands, which differ from those within the AND gate itself (left panel). **c**, Using *MVsim* to sample the parameter space of a three-input AND gate identifies an improved theoretical design (left and middle panels) that avoids contorted and unfavorable binding configurations present in (**b**) through an inline topology, longer linkages, and a matched set of kinetic rate constants for association and dissociation. Simulated performance (right panel) of the optimized three-input design shows suppressed spurious activation in the presence of single and dual inputs. Here, again, the K_D and concentration values (right panel) refer to those of the input ligands, which differ from those within the AND gate (left panel). Values for persistence length were obtained from literature, as described for Fig. 3 (8-11). Domain diameters were obtained from the Protein Data Bank (PDB; www.rcsb.org): N-WASP (PDB id: 2LNH, 1CEE; ref. 12,13); PDZ (PDB id: 1QAV, 2PDZ; ref. 14,15); and SH3 (PDB id: 1CKA; ref. 16). **d**, The kinetic and topological parameters for the multispecific, multivalent in simulations in Fig. 4c. The simulations in Fig. 4c report the receptor-ligand binding responses at equilibrium with ligand concentrations of 4 nM. The kinetic rate constants for the interaction between cognate receptor-ligand domains are indicated by the same font color.

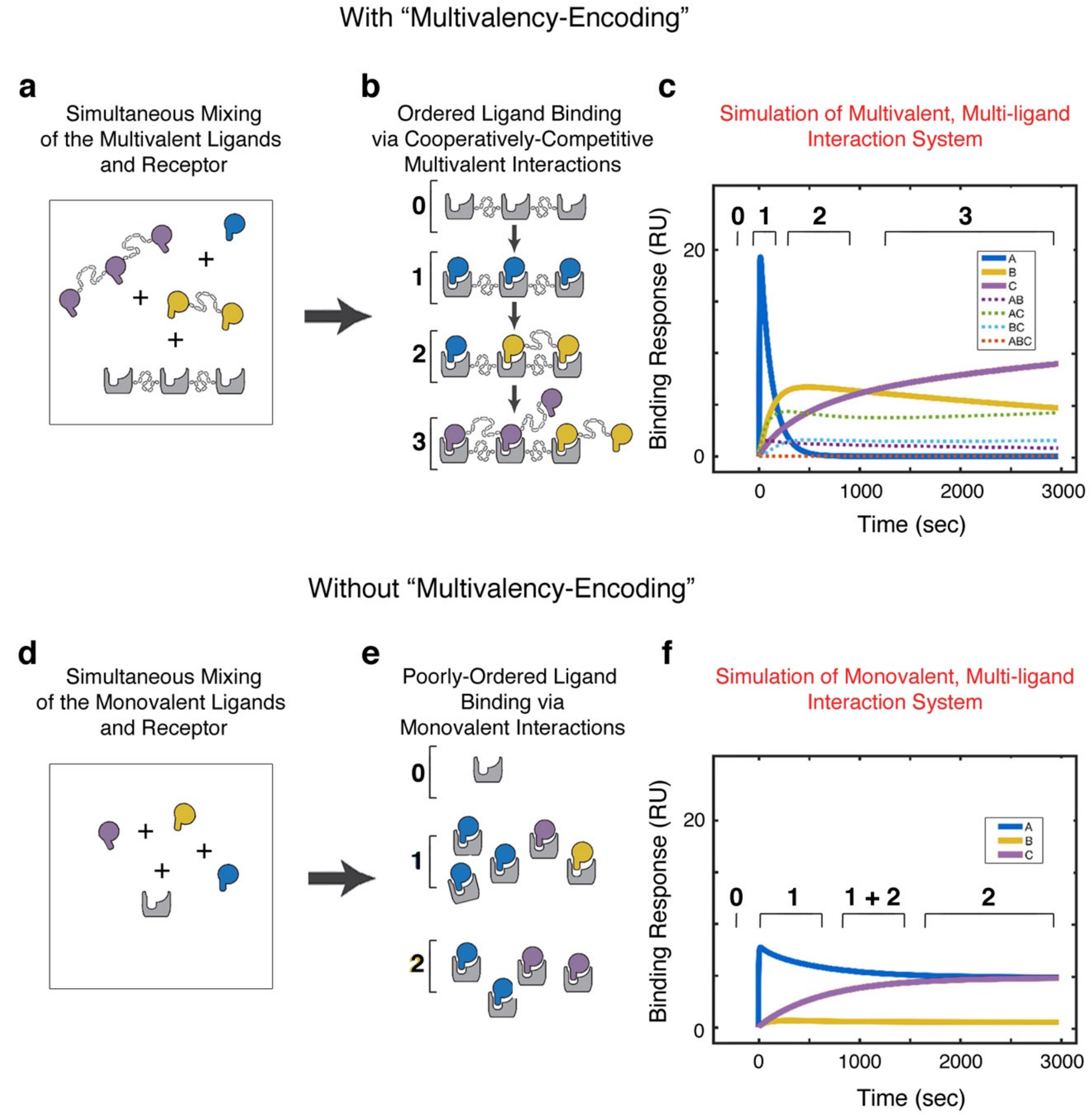

Supplementary Fig. 7: ***MVsim*** **illustrates the use of multivalent interactions in the temporal coding of sequential ligand interactions.** Parameterization of *MVsim* for a model system of interactions between three ligands (colored purple, blue, and yellow) and their common receptor (colored grey) (**a**) uncovers designs for temporally-encoded multivalent barcodes that effect a dynamic and sequential pattern of multi-ligand interactions (**b**). **c**, Here, a simulated multispecific design enables an orderly progression of binding events among the three ligands. The ordered progression of binding events is achieved through cooperative, competitive inter-ligand displacement from the receptor by leveraging a combination of kinetic rate constants of association, avidity, and binding stoichiometry. **d**, An identical simulated approach was applied to corresponding system of monovalent ligands and their receptor with binding kinetics and affinities that are comparable to the effective kinetic rate and equilibrium constants (i.e., k_{on}^{eff} , k_{off}^{eff}, and K_D^{eff}) to their multivalent counterparts. **e**,**f**, Such a parameterized monovalent system was significantly limited in its simulated ability to achieve the ordered sequencing of ligand binding that is displayed by its multivalent counterpart in (**b**,**c**).

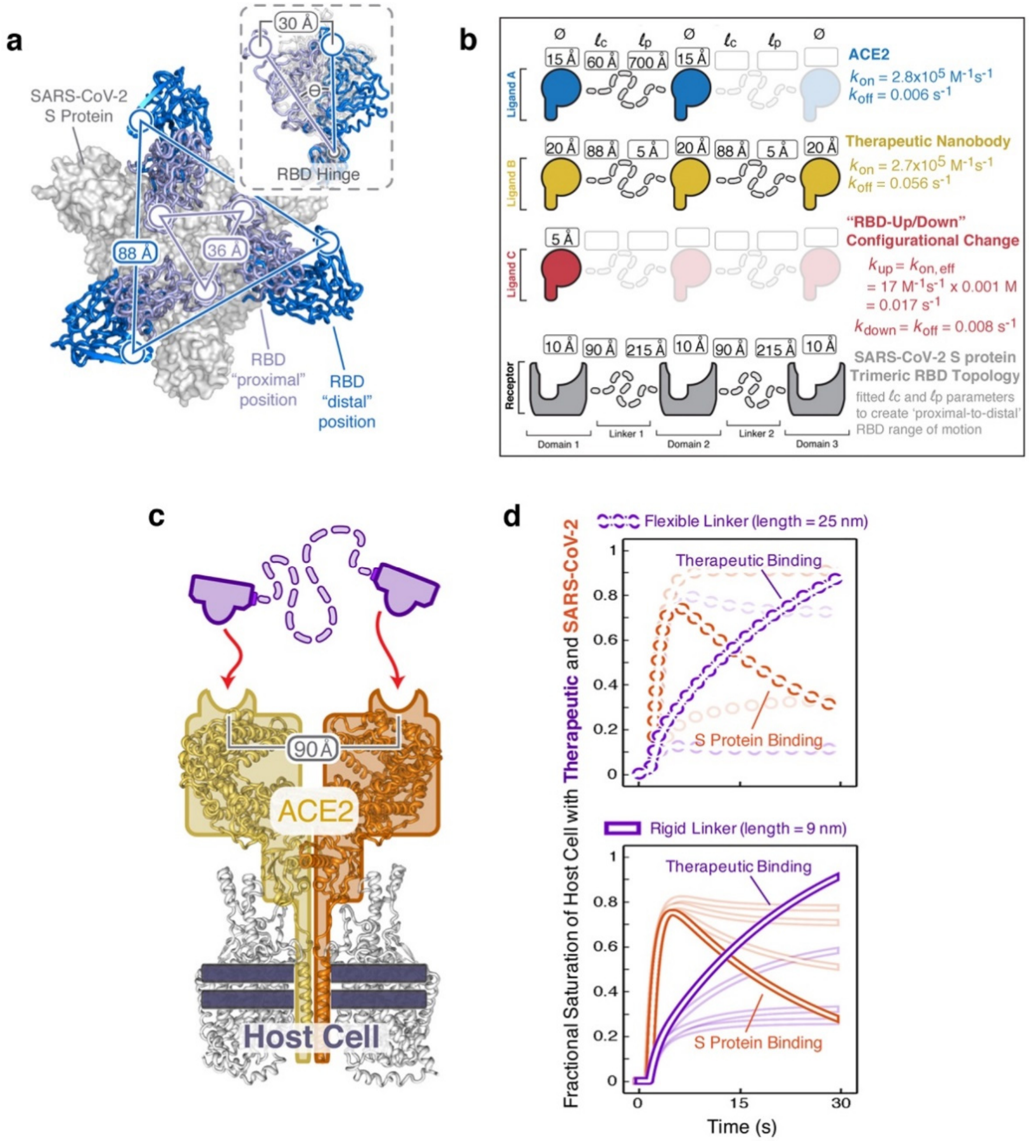

Supplementary Fig. 8: *MVsim* can predict SARS-CoV-2 S protein binding response dynamics using a combination of experimentally-determined parameters and fitted parameters extracted from structural and binding studies. a, Superposition of ten S protein RBD conformations (PDB id: 5X5B, 6CRW, 6NB6, 6NB7, 6VSB, 6VYB; ref. 17-21) show a span of conformations that the three S protein receptor binding domains (RBDs) can sample. The limits of motion captured in these static structures can be used to parameterize *MVsim*. **b**, A set of topological and kinetic parameters (domain diameter, ϕ; linker contour length ℓc; and linker persistence length, ℓp) were derived that can approximate the constraints of the S protein (grey), ACE2 (blue), and RBD (red) configurational dynamics using the linear, beads-on-a-string topology that *MVsim* is based upon. To parameterize the simulations in Fig. 5b, the bivalent topology of ACE2 (blue) was simulated binding to a bivalent RBD receptor with its linker made flexible (ℓp = 5 Å; adapted from the S protein parameters, colored grey). Further, to generate the IC_{50} plots in Fig. 5d, a second "therapeutic" ACE2 was introduced into the simulation (identically parameterized as colored in blue) in lieu of the nanobody (colored yellow). **c**, In addition to the strategic therapeutic targeting of the S protein (as explored in Fig. 5c-f), binders targeted to the RBD-binding surface of the ACE2 dimer have been assessed for their therapeutic potential. Here, available structures for full-length ACE2 and the ACE2-RBD complex (PDB id: 6M17, 6VW1; ref. 22,23) were used to parameterize *MVsim* with descriptive multivalent topologies, domain diameters, kinetic rate constants of association and dissociation, and contour and persistence lengths that provide bounds for the captured ranges of motion. Simulations were performed to determine the optimal lengths for both flexible linkages (**d**, top plot) and rigid linkages (**d**, bottom plot) that provide maximal protective shielding (darker purple curves in both plots) and therefore result in the largest reductions of SARS-CoV-2 infection (darker orange curves in both plots). *MVsim* predicts the level of protection that a shielding therapeutic can provide when the linker connecting the two binding regions is changed.

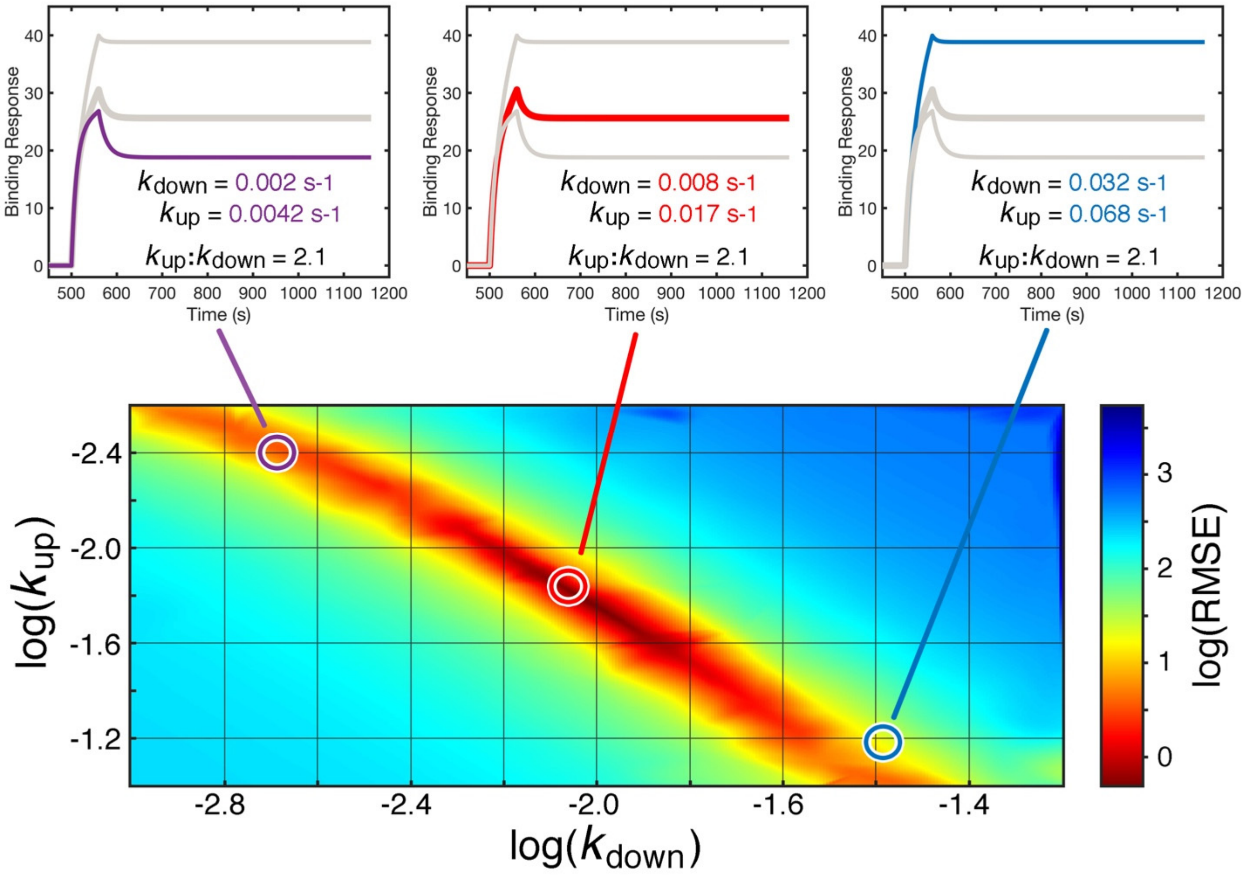

Supplementary Fig. 9: Conformational rates of SARS-CoV-2 S protein RBD switching are diagnostically and quantitatively captured in the multiphasic SPR binding responses. To further show that the rate constants of RBD switching are uniquely determined by the multiphasic binding responses, the fitted rate constants (center plot; colored red) were both changed to be 4-fold slower (left plot; colored purple) or 4-fold faster (right plot; colored blue), such that the k_{up}: k_{down} ratio – and the equilibrium populations of RBD-up and RBD-down – remained constant. Despite the constant ratio, decreasing (indicated by the purple circle) or increasing (indicated by the blue circle) the rate constants from the values producing the global minimum in RMSE (red circle in the middle of the heat map) results in substantial deviation from the best-fit solution. This analysis visually highlights the sensitivity of the multivalent binding response curves to the values of the rate constants of S protein conformational switching.

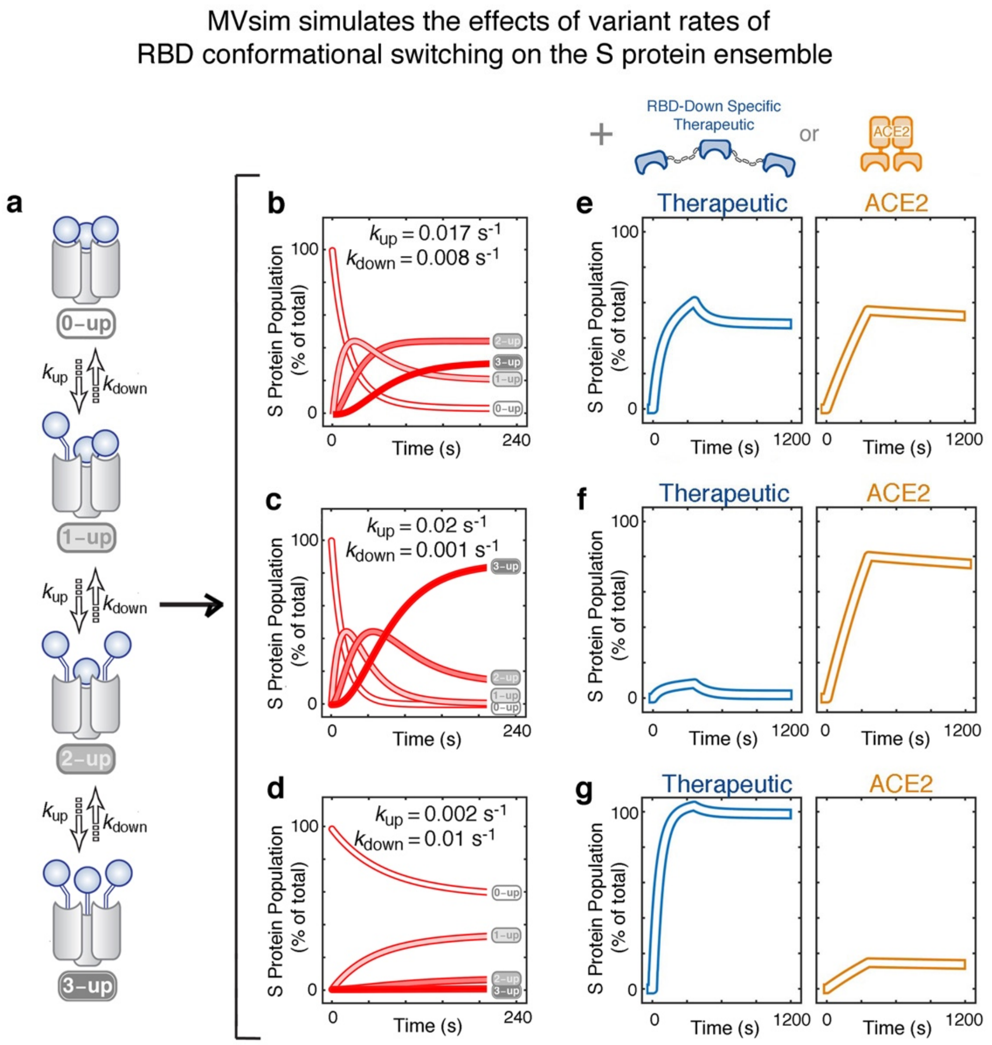

Supplementary Fig. 10: *MVsim* enables the parameterized SARS-CoV-2 S protein simulation to model the consequences of variation of the RBD-up/down rates of switching on the S protein conformational ensemble. **a**, *MVsim* can simulate the conformational ensemble of S protein RBD states as a function of changing RBD rate constants (e.g., those hypothesized to occur in some S protein mutational variants). **b**,**c**,**d**, *MVsim* modeling of the system with k_{up}: k_{down} ratios of (**b**) 2.1, (**c**) 20, and (**d**) 0.2 show simulated effects on the ensemble of four RBD configurations (0-up, 1-up, 2-up, and 3-up). **e**,**f**,**g**, To further simulate the effects of variant rates of RBD conformational switching, the simulated S proteins (**b**,**c**,**d**) were each introduced to either a down-specific trivalent therapeutic nanobody (as treated in Fig. 6) or a dimeric ACE2. Therapeutic and ACE2 binding were performed as simulated SPR experiments with an association phase (0-300 s) and a dissociation phase (300-1200 s).

Supplementary Table 1: Protein sequences of the multivalent receptors and ligands used in this study.

Protein Name	Type	Amino Acid Sequence
His$_6$-AviTag -<br>GADS SH3 -<br>GADS SH3 -<br>GADS SH3<br><br>*monospecific, trivalent with long rigid linkers*	Receptor<br><br>*used in Fig. 3b,c*	MGSSHHHHHHSSGLVPRGSHMAGLNDIFEAQKIEWHEGSMGSNTIDLPMS<br>PRTLDSLMQFGNNGTSVRWARALYDFEALEEDELGFRSGEVVEVLDSSNP<br>SWWTGRLHNKLGLFPANYVAPMMRSGTGSTGPAEAAAKEAAAKEAAAKEA<br>AAKEAAAKEAAAKEAAAKEAAAKAPASTSVRWARALYDFEALEEDELGFR<br>SGEVVEVLDSSNPSWWTGRLHNKLGLFPANYVAPMMRVDTGPAEAAAKEA<br>AAKEAAAKEAAAKEAAAKEAAAKEAAAKEAAAKAPASVRWARALYDFEAL<br>EEDELGFRSGEVVEVLDSSNPSWWTGRLHNKLGLFPANYVAPMMR
His$_6$-AviTag -<br>Pdar -<br>GADS SH3 -<br>GADS SH3<br><br>*bispecific, trivalent with long rigid linkers*	Receptor<br><br>*used in Fig. 3d,f and Fig. 3g,i*	MGSSHHHHHHSSGLVPRGSHMAGLNDIFEAQKIEWHEGSMGSNTIDLPMS<br>PRTLDSLMQFGNNGTSGDNLNYQSGGQDLGKKLLEAAAAGQDDEVRILMA<br>NGADVNATDDNGLTPLHLAAANGQLEIVEVLLKNGADVNASDSAGITPLH<br>LAAYDGHLEIVEVLLKHGADVNAYDRAGWTPLHLAALSGQLEIVEVLLKH<br>GADVNAQDALGLTAFDISINQGQEDLAEILQSGTGSTGPAEAAAKEAAAK<br>EAAAKEAAAKEAAAKEAAAKEAAAKEAAAKAPASTSVRWARALYDFEALE<br>EDELGFRSGEVVEVLDSSNPSWWTGRLHNKLGLFPANYVAPMMRVDTGPA<br>EAAAKEAAAKEAAAKEAAAKEAAAKEAAAKEAAAKEAAAKAPASVRWARA<br>LYDFEALEEDELGFRSGEVVEVLDSSNPSWWTGRLHNKLGLFPANYVAPM<br>MR
MBP -<br>SLP76 peptide -<br>SLP76 peptide -<br>SLP76 peptide -<br><br>*monospecific, trivalent with long rigid linkers*	Ligand<br><br>*used in Fig. 3b,c*	MKIEEGKLVIWINGDKGYNGLAEVGKKFEKDTGIKVTVEHPDKLEEKFPQ<br>VAATGDGPDIIFWAHDRFGGYAQSGLLAEITPDKAFQDKLYPFTWDAVRY<br>NGKLIAYPIAVEALSLIYNKDLLPNPPKTWEEIPALDKELKAKGKSALMF<br>NLQEPYFTWPLIAADGGYAFKYENGKYDIKDVGVDNAGAKAGLTFLVDLI<br>KNKHMNADTDYSIAEAAFNKGETAMTINGPWAWSNIDTSKVNYGVTVLPT<br>FKGQPSKPFVGVLSAGINAASPNKELAKEFLENYLLTDEGLEAVNKDKPL<br>GAVALKSYEEELVKDPRIAATMENAQKGEIMPNIPQMSAFWYAVRTAVIN<br>AASGRQTVDEALKDAQTNSSSNNNNNNNNNNLGIEGRISHMGTSGSPAPS<br>ID**R**ST**K**PPLAGSPAEAAAKEAAAKEAAAKEAAAKEAAAKEAAAKEAAAKE<br>AAAKAPSGSPAPSID**R**ST**K**PPLASPAEAAAKEAAAKEAAAKEAAAKEAAA<br>KEAAAKEAAAKEAAAKAPSGSPAPSID**R**ST**K**PPLAGSNGSAS
MBP -<br>SLP76 peptide -<br>SLP76 peptide -<br>Prb<br><br>*bispecific, trivalent with long flexible linkers*	Ligand<br><br>*used in Fig. 3d,f*	MKIEEGKLVIWINGDKGYNGLAEVGKKFEKDTGIKVTVEHPDKLEEKFPQ<br>VAATGDGPDIIFWAHDRFGGYAQSGLLAEITPDKAFQDKLYPFTWDAVRY<br>NGKLIAYPIAVEALSLIYNKDLLPNPPKTWEEIPALDKELKAKGKSALMF<br>NLQEPYFTWPLIAADGGYAFKYENGKYDIKDVGVDNAGAKAGLTFLVDLI<br>KNKHMNADTDYSIAEAAFNKGETAMTINGPWAWSNIDTSKVNYGVTVLPT<br>FKGQSPSKPFVGVLSAGINAASPNKELAKEFLENYLLTDEGLEAVNKDKP<br>LGAVALKSYEEELVKDPRIAATMENAQKGEIMPNIPQMSAFWYAVRTAVI<br>NAASGRQTVDEALKDAQTNSSSNNNNNNNNNNLGIEGRISHMGTSGSPAP<br>SID**R**ST**K**PPLAGSTGGDNSNSGGSGNSGGSGGNSGSTSGDNSNSGGSGNS<br>GGASGNSPAPSID**R**ST**K**PPLAGSTGGDNSNSGGSGNSGGSGGNSGSTSGD<br>NSNSGGSGNSGGASGNSATRPIDGLTDEGIRETLTRYKKIALVGASPKPE<br>RDANIVMKYLLEHGYDVYPVNPNYEEVLGRKCYPSVLDIPDKIEVVDLFV<br>NPAKAWRFVAYAIKKGAKVVWFQYNTYYPLAARQAKGAGLIIVANRCMMR<br>EHKRLLGE
MBP -<br>SLP76 peptide -<br>Prb	Ligand<br><br>*used in Fig.*	MKIEEGKLVIWINGDKGYNGLAEVGKKFEKDTGIKVTVEHPDKLEEKFPQ<br>VAATGDGPDIIFWAHDRFGGYAQSGLLAEITPDKAFQDKLYPFTWDAVRY<br>NGKLIAYPIAVEALSLIYNKDLLPNPPKTWEEIPALDKELKAKGKSALMF

bispecific, bivalent with long flexible linkers	*3g,i*	NLQEPYFTWPLIAADGGYAFKYENGKYDIKDVGVDNAGAKAGLTFLVDLI<br>KNKHMNADTDYSIAEAAFNKGETAMTINGPWAWSNIDTSKVNYGVTVLPT<br>FKGQSPSKPFVGVLSAGINAASPNKELAKEFLENYLLTDEGLEAVNKDKP<br>LGAVALKSYEEELVKDPRIAATMENAQKGEIMPNIPQMSAFWYAVRTAVI<br>NAASGRQTVDEALKDAQTNSSSNNNNNNNNNNLGIEGRISHMGTSGSPAP<br>SID**R**ST**K**PPLAGSTGGDNSNSGGSGNSGGSGGNSGSTSGDNSNSGGSGNS<br>GGASGNSATRPIDGLTDEGIRETLTRYKKIALVGASPKPERDANIVMKYL<br>LEHGYDVYPVNPNYEEVLGRKCYPSVLDIPDKIEVVDLFVNPAKAWRFVA<br>YAIKKGAKVVWFQYNTYYPLAARQAKGAGLIIVANRCMMREHKRLLGE
MBP -<br>SLP76 peptide<br><br>*monovalent* with long flexible linkers*<br><br>** alanine substitutions (**bolded in red**) render protein monovalent*	Ligand<br><br>*used in Fig. 3g,i*	MKIEEGKLVIWINGDKGYNGLAEVGKKFEKDTGIKVTVEHPDKLEEKFPQ<br>VAATGDGPDIIFWAHDRFGGYAQSGLLAEITPDKAFQDKLYPFTWDAVRY<br>NGKLIAYPIAVEALSLIYNKDLLPNPPKTWEEIPALDKELKAKGKSALMF<br>NLQEPYFTWPLIAADGGYAFKYENGKYDIKDVGVDNAGAKAGLTFLVDLI<br>KNKHMNADTDYSIAEAAFNKGETAMTINGPWAWSNIDTSKVNYGVTVLPT<br>FKGQSPSKPFVGVLSAGINAASPNKELAKEFLENYLLTDEGLEAVNKDKP<br>LGAVALKSYEEELVKDPRIAATMENAQKGEIMPNIPQMSAFWYAVRTAVI<br>NAASGRQTVDEALKDAQTNSSSNNNNNNNNNNLGIEGRISHMGTSGSPAP<br>SID**R**ST**K**PPLAGSTGGDNSNSGGSGNSGGSGGNSGSTSGDNSNSGGSGNS<br>GGASGNSPAPSID**A**ST**A**PPLAGSTGGDNSNSGGSGNSGGSGGNSGSTSGD<br>NSNSGGSGNSGGASGNSPAPSID**A**ST**A**PPLAGSNGSAS

4. *MVsim* User Tutorial

The user tutorial covers the following topics:

Tutorial 1: Interacting with the MVsim graphical user interface

Tutorial 2: Advanced application of MVsim for the S protein

A standalone copy of the user tutorial is provided with the *MVsim* software that accompanies this paper and is also available on GitHub (https://sarkarlab.github.io/MVsim/).

Simulating Multivalent Biomolecular Interactions

Tutorial 1: Interacting with the MVsim graphical user interface

Bruncsics et al. (2022)

a. MVsim initiates with a bivalent interaction demo preset

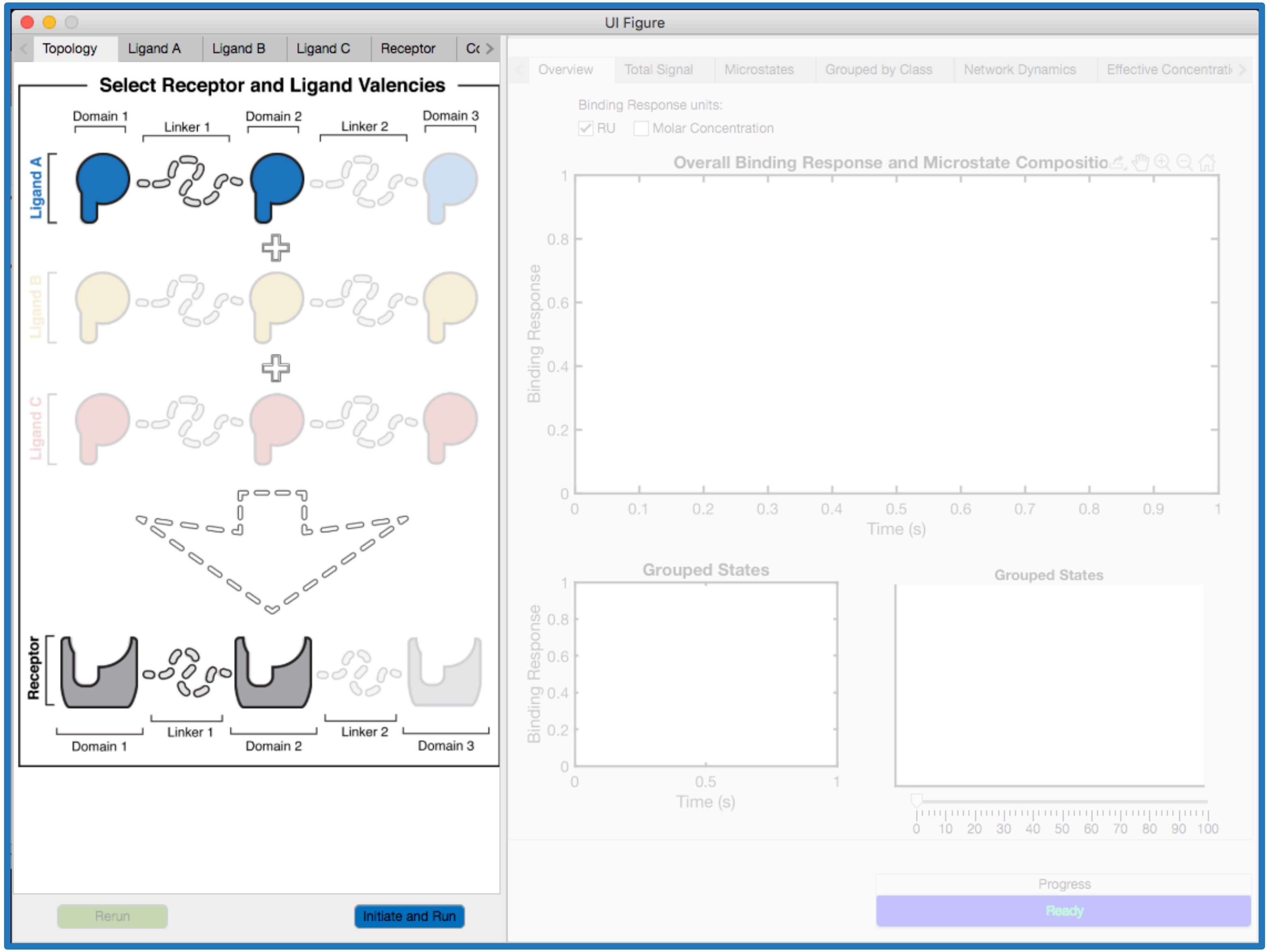

b. A bivalent Ligand A and bivalent Receptor are indicated in the Topology tab

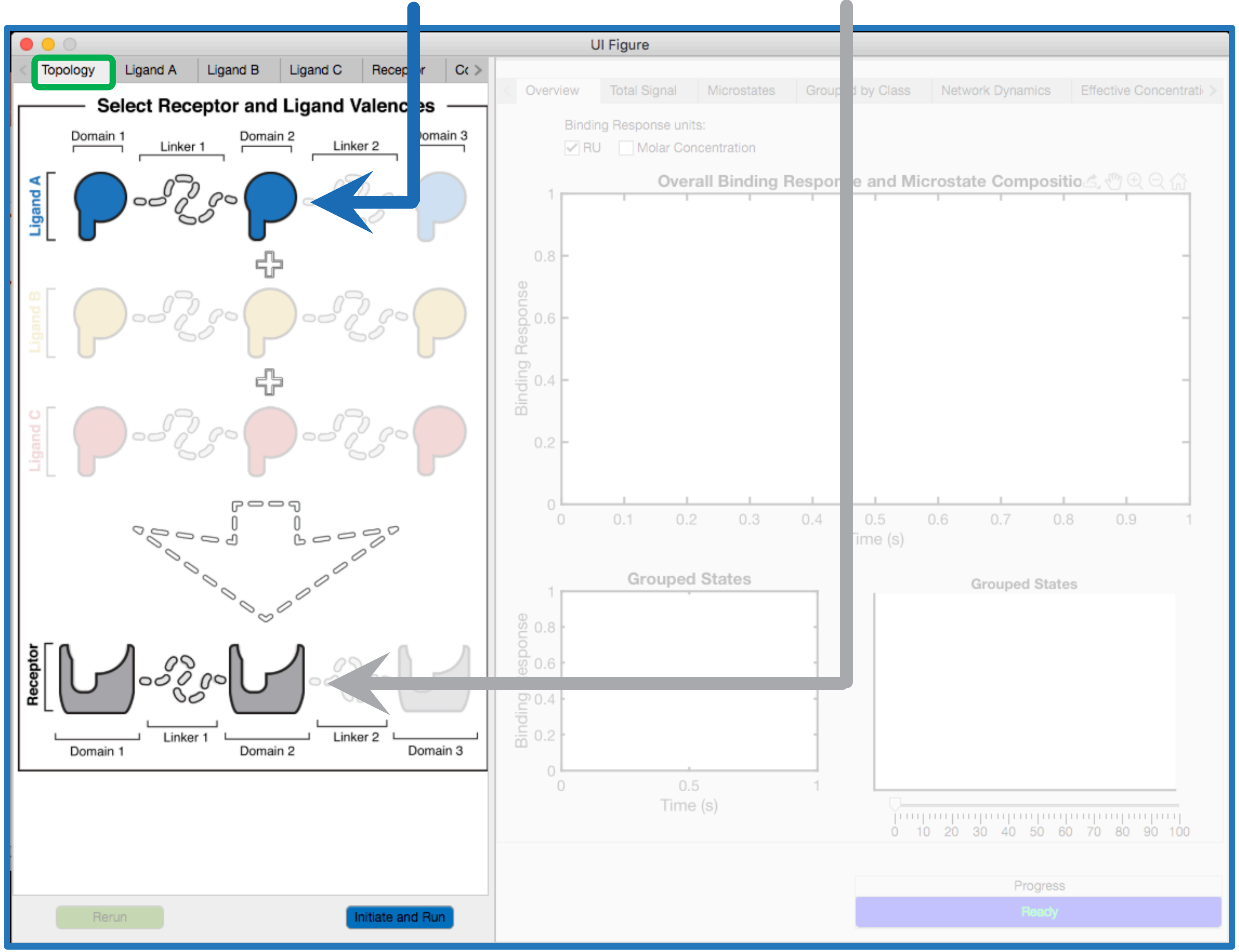

1. Designing a multivalent interaction system in the Topology tab

c. Clicking the binding domain icons will toggle between mono, bi, and trivalent

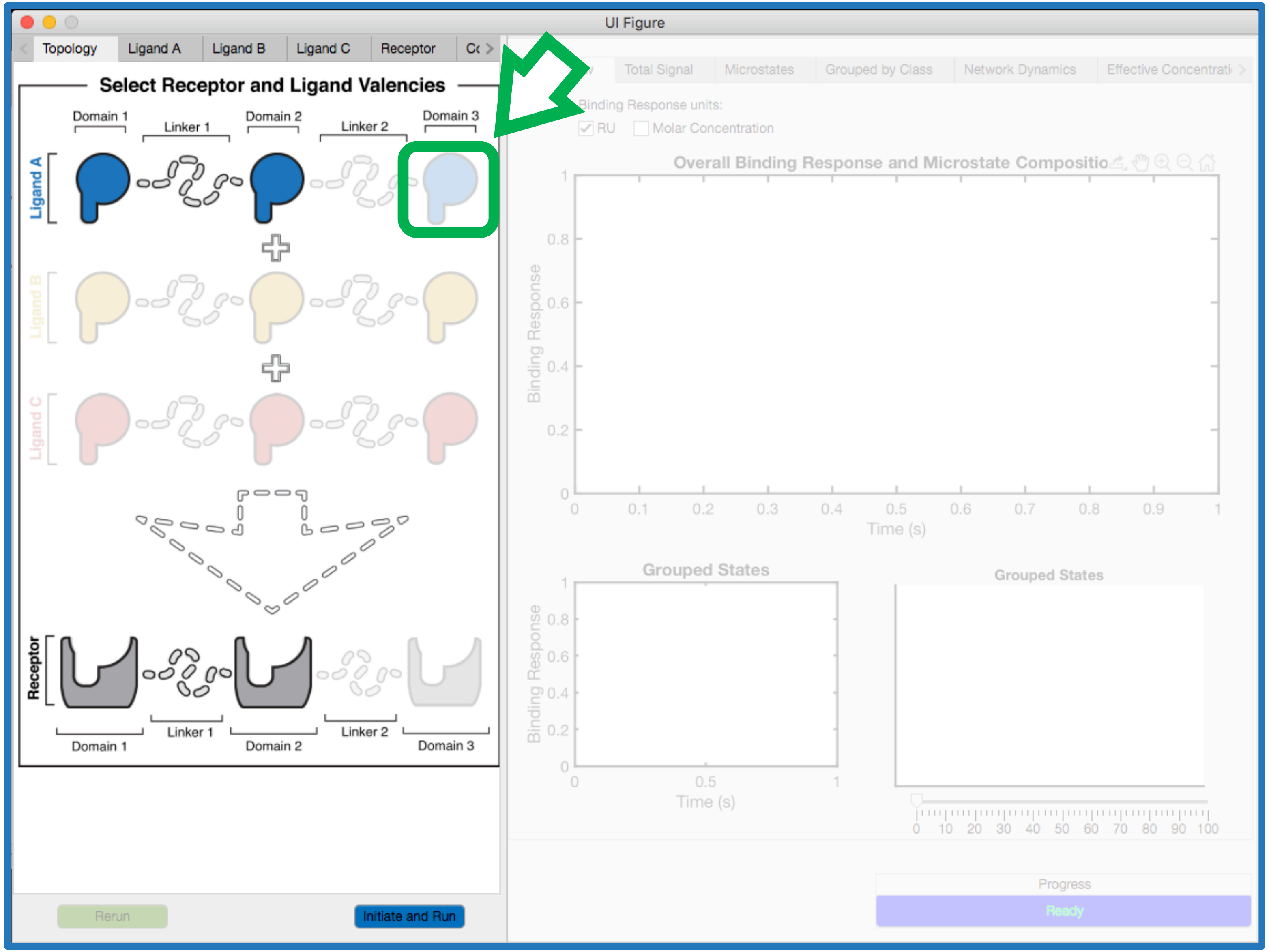

1. Designing a multivalent interaction system in the Topology tab

c. Clicking the binding domain icons will toggle between mono, bi, and trivalent

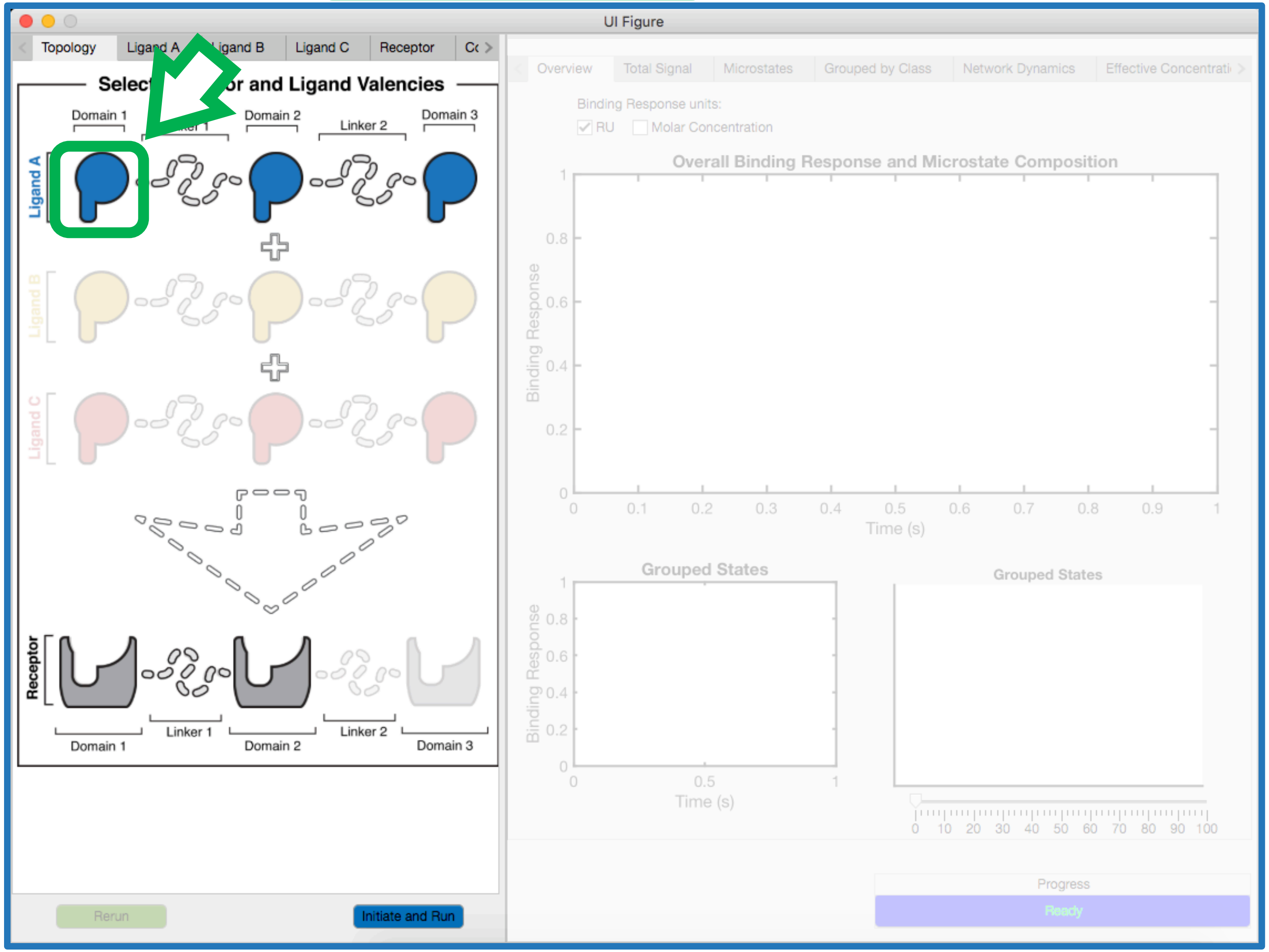

d. Clicking a monovalent ligand a second time will remove it from the simulation

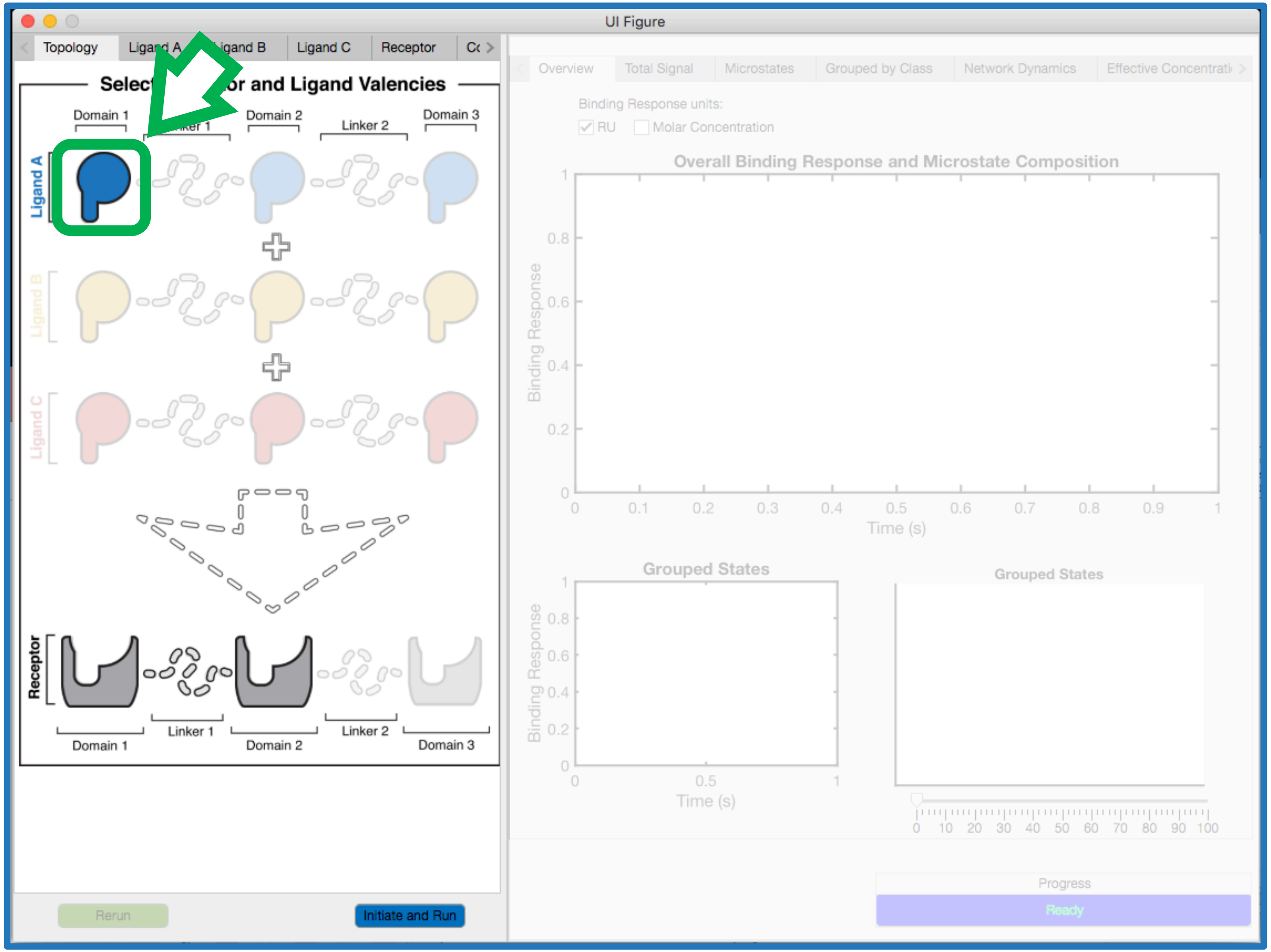

d. Clicking a monovalent ligand a second time will remove it from the simulation

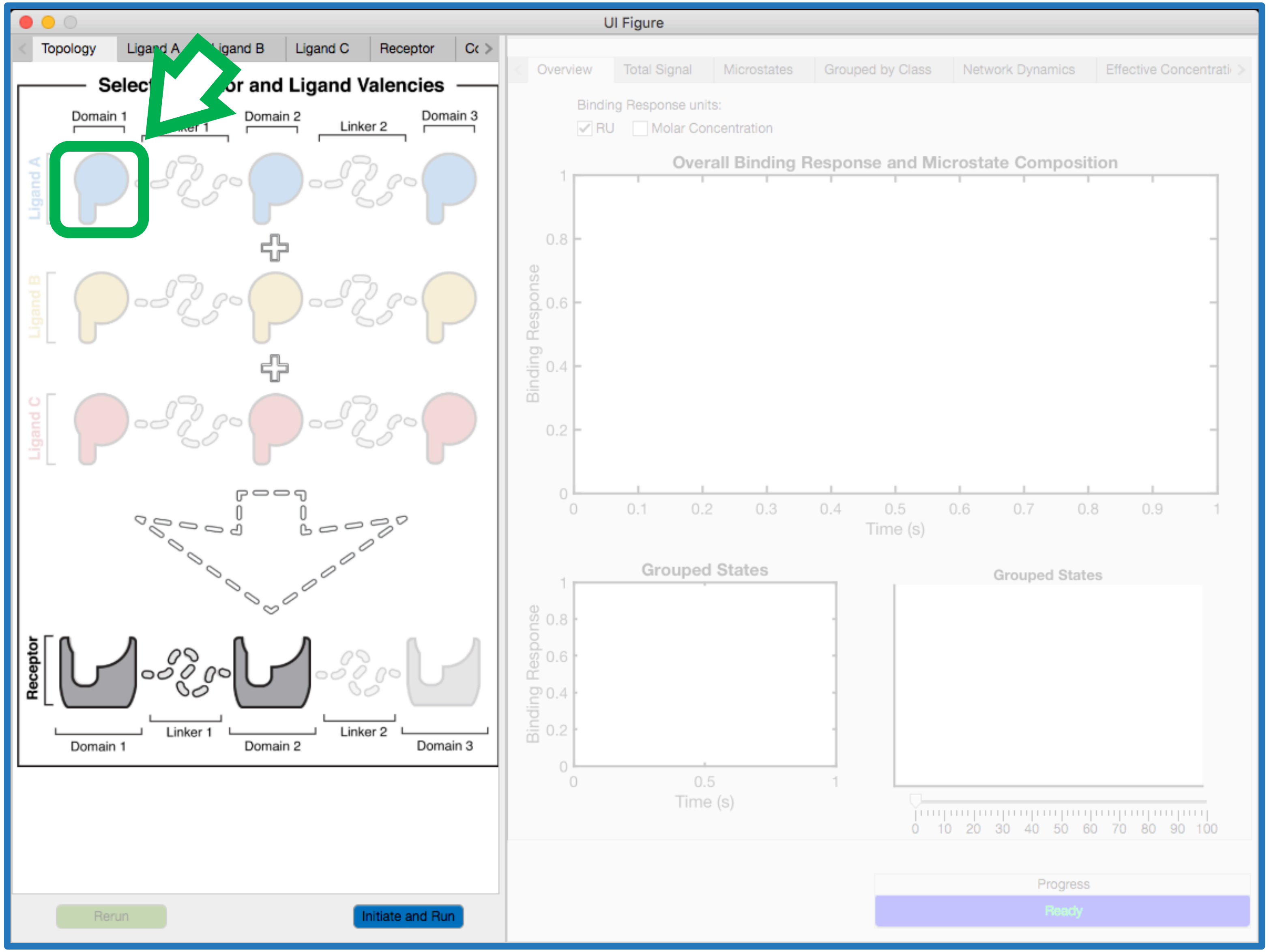

e. Ligand B and Ligand C are similarly added, removed, and their valencies selected

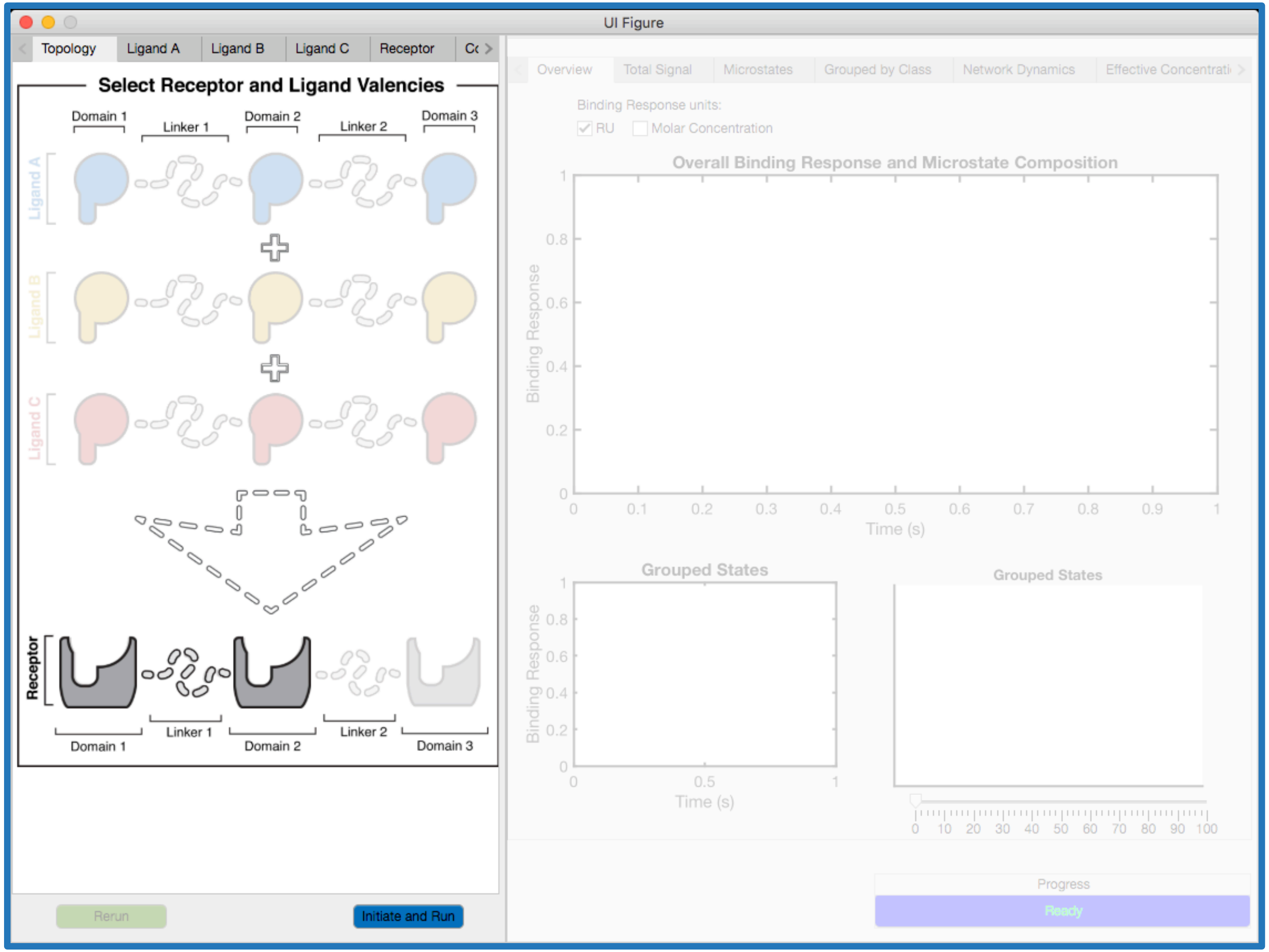

e. Ligand B and Ligand C are similarly added, removed, and their valencies selected

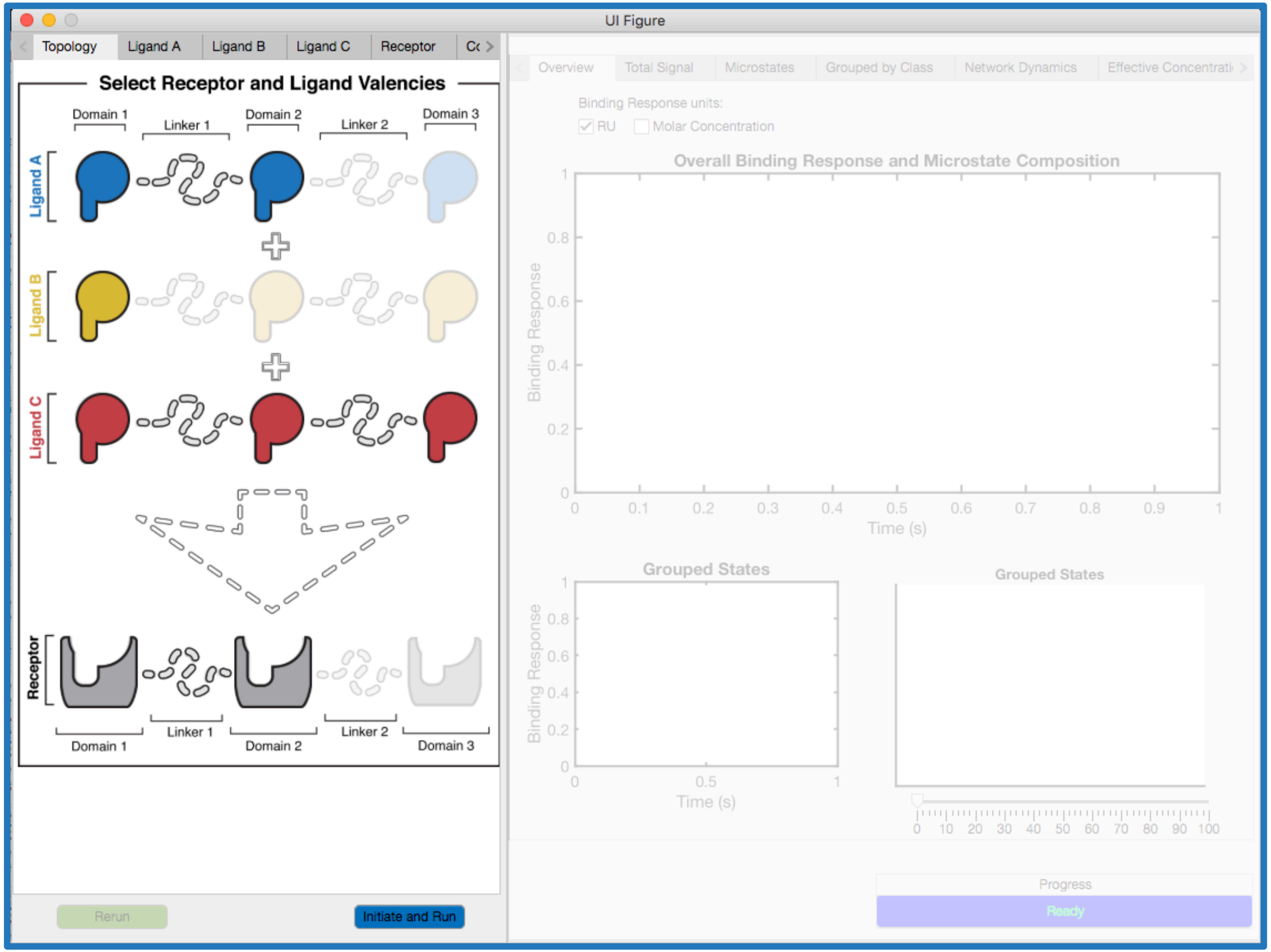

f. The Receptor is permanently active and toggled between mono, bi, and trivalency

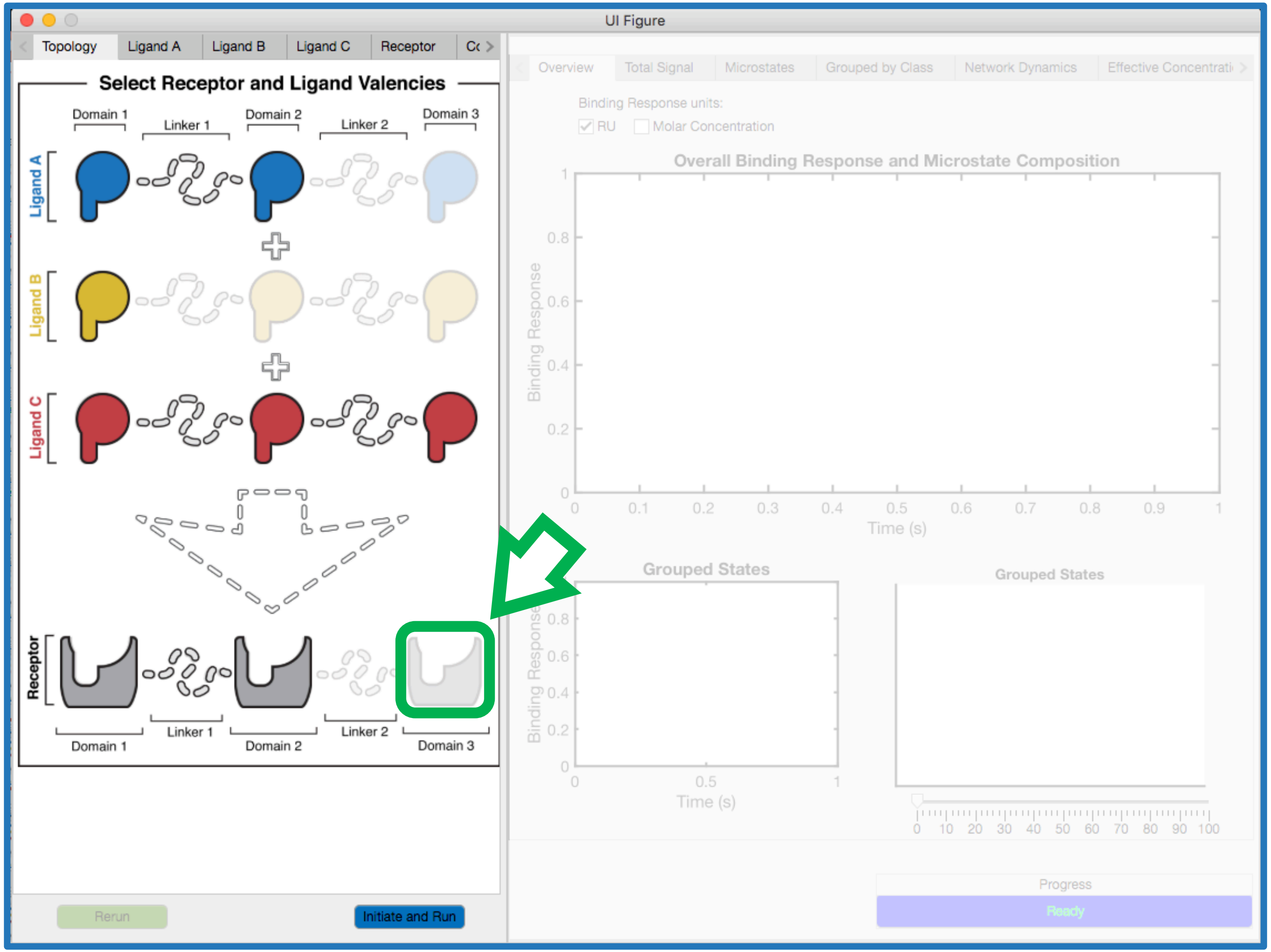

1. Designing a multivalent interaction system in the Topology tab

f. The Receptor is permanently active and toggled between mono, bi, and trivalency

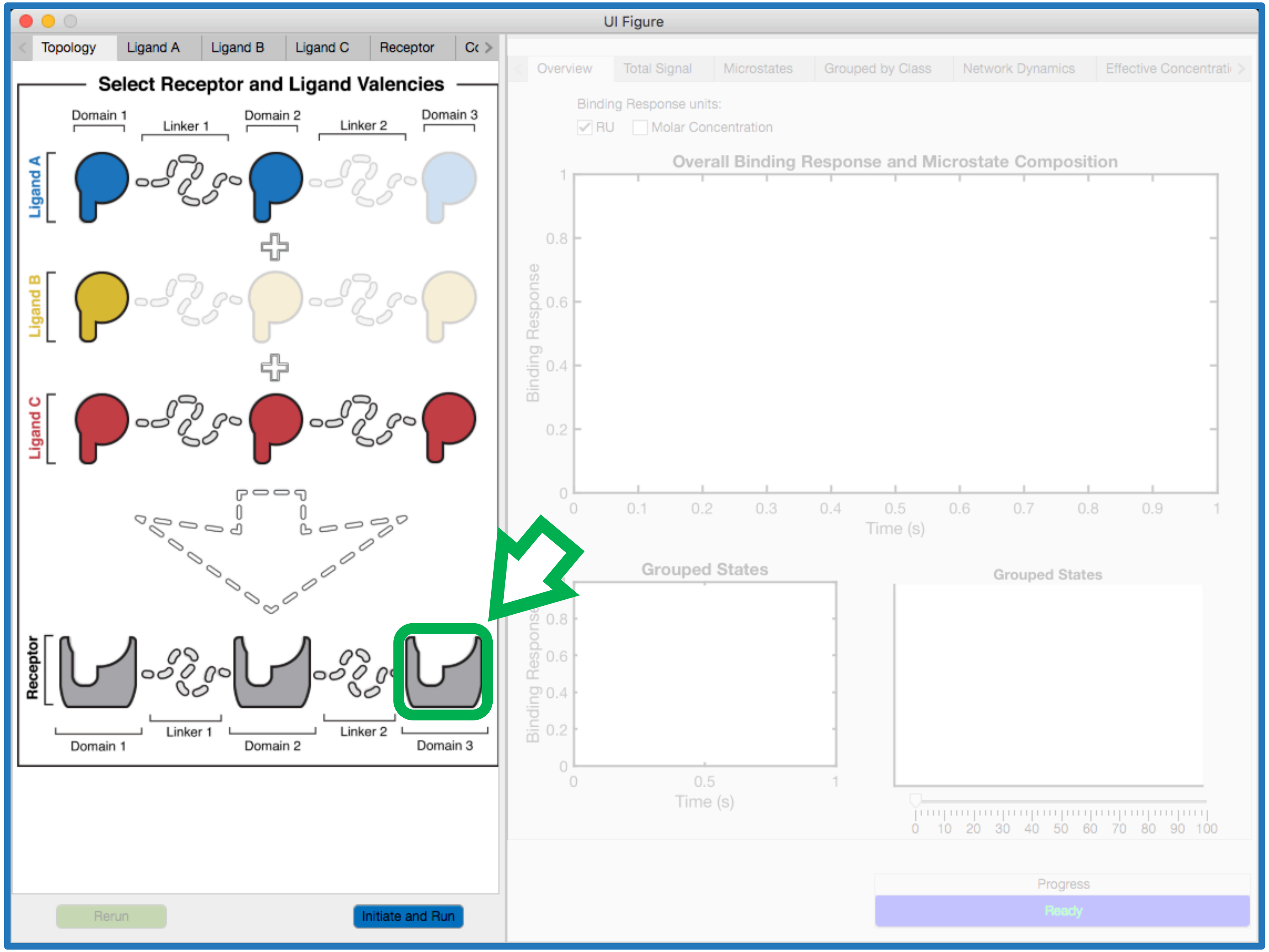

2. Parameterizing the multivalent system in the Ligand tabs

a. For a given topology the Ligand tabs enable input of kinetic and structural parameters

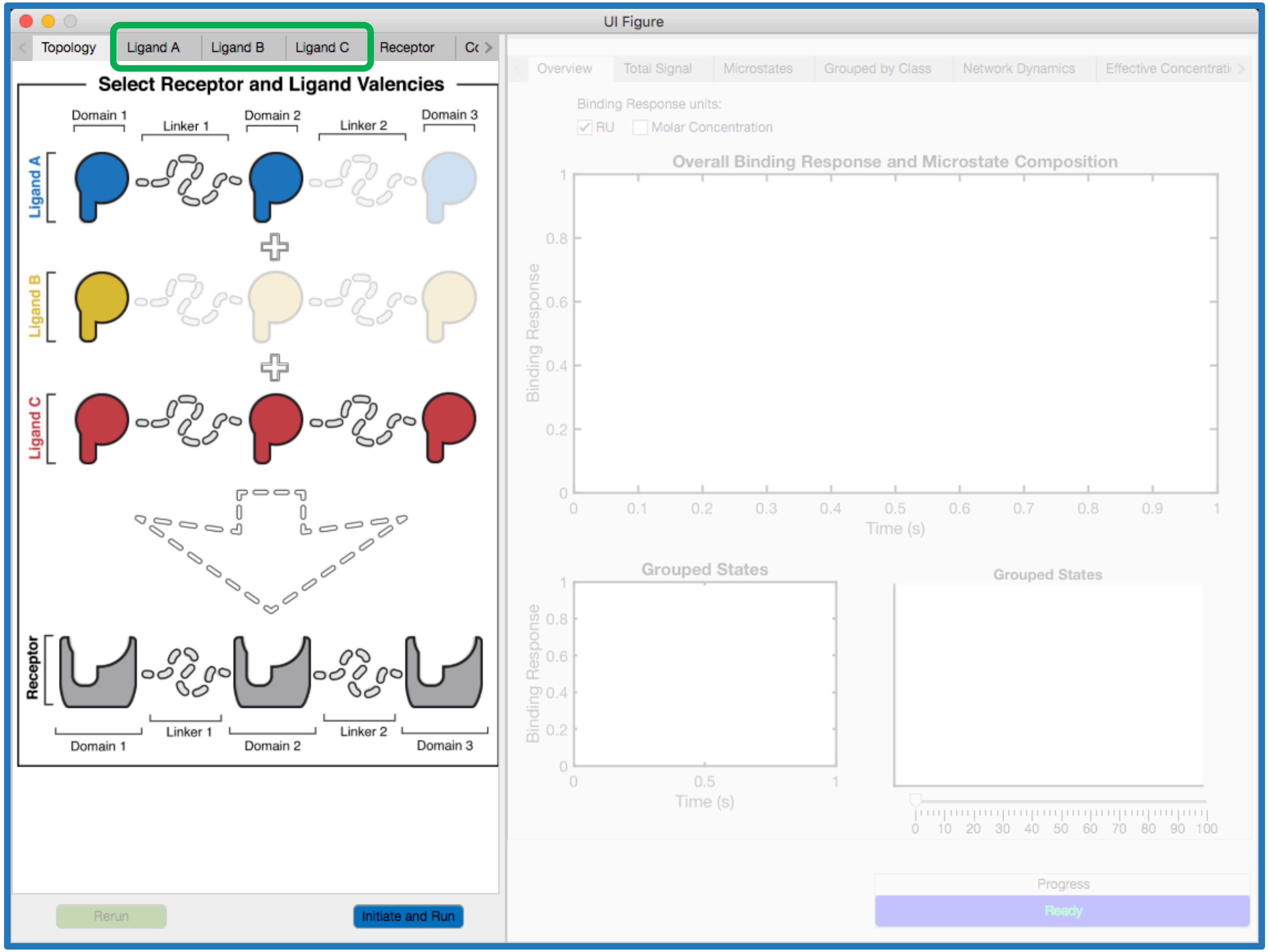

b. User input parameterization of Ligand A

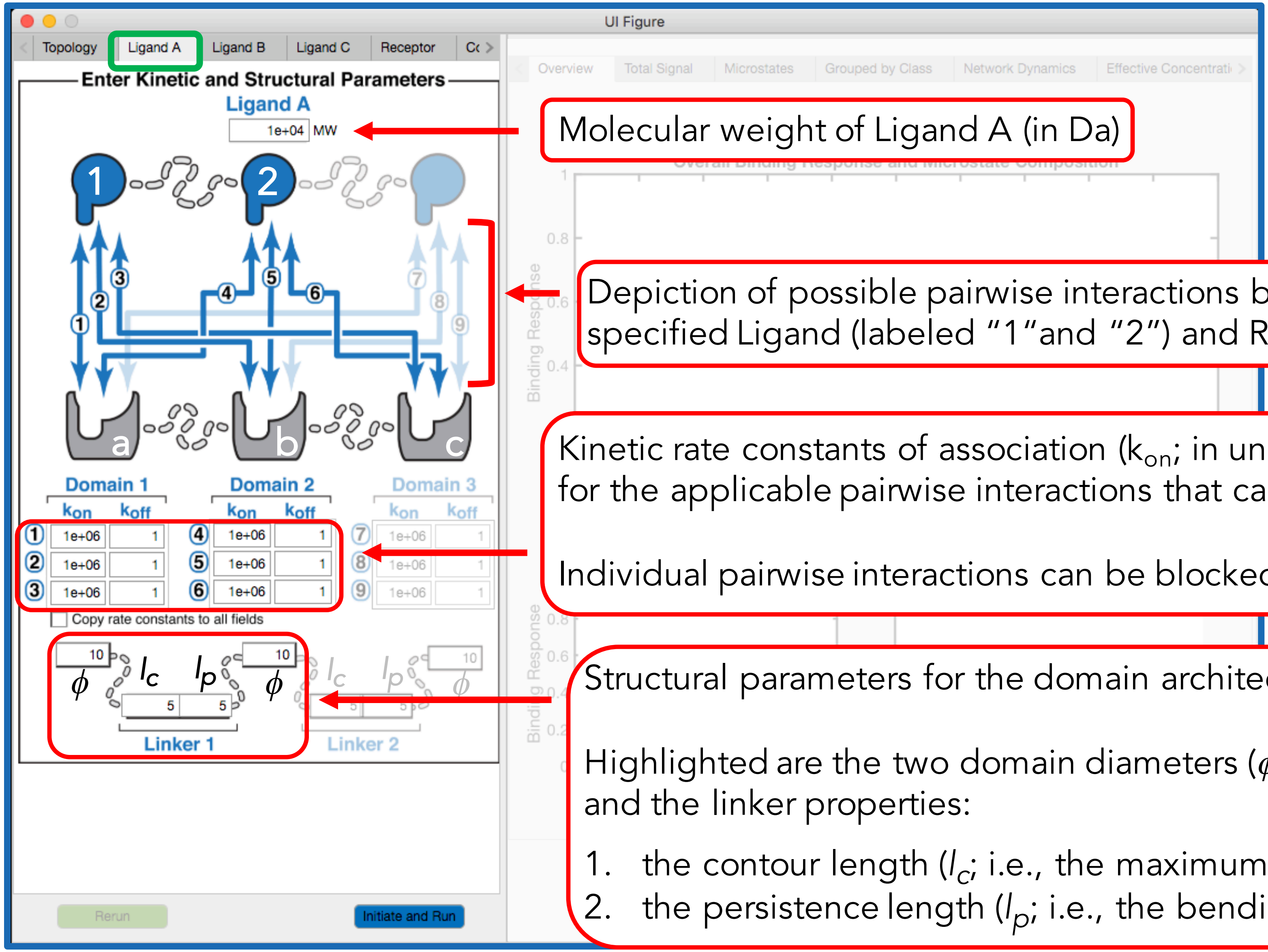

Molecular weight of Ligand A (in Da)

Depiction of possible pairwise interactions between the binding domains of the specified Ligand (labeled “1”and “2”) and Receptor (labeled “a”, “b”, and “c”)

Kinetic rate constants of association (k_{on}; in units of $M^{-1}s^{-1}$) and dissociation (k_{off}; in units of s^{-1}) for the applicable pairwise interactions that can occur between Ligand A and the Receptor

Individual pairwise interactions can be blocked by entering “0” for the respective k_{on} and k_{off}

Structural parameters for the domain architecture of a bivalent Ligand A.

Highlighted are the two domain diameters (ϕ, units of Angstrom) and the linker properties:

1. the contour length (l_c; i.e., the maximum end-to-end distance; units of Angstrom)
2. the persistence length (l_p; i.e., the bending stiffness; units of Angstrom)

2. Parameterizing the multivalent system in the Ligand tabs

c. User input parameterization of Ligand B

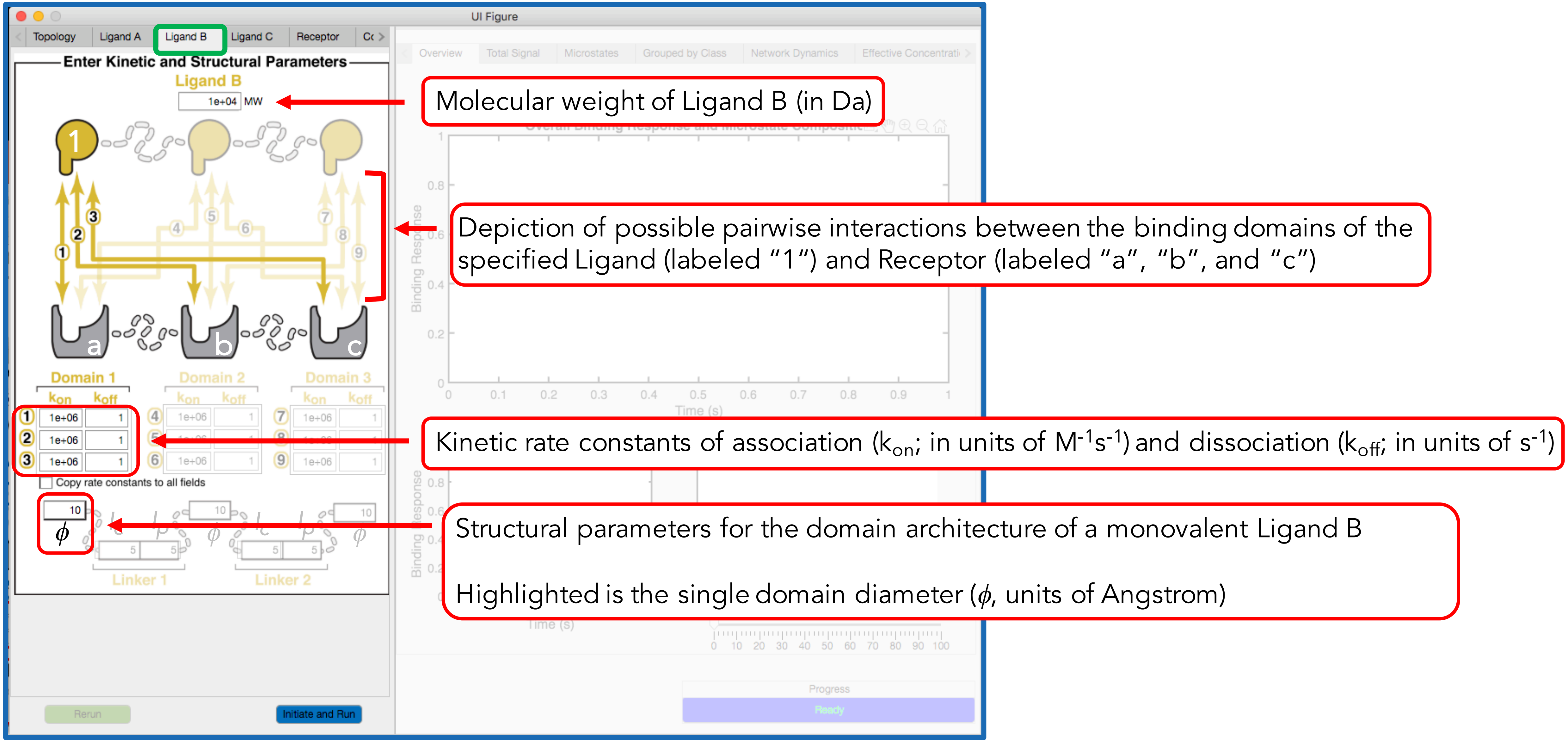

d. User input parameterization of Ligand C

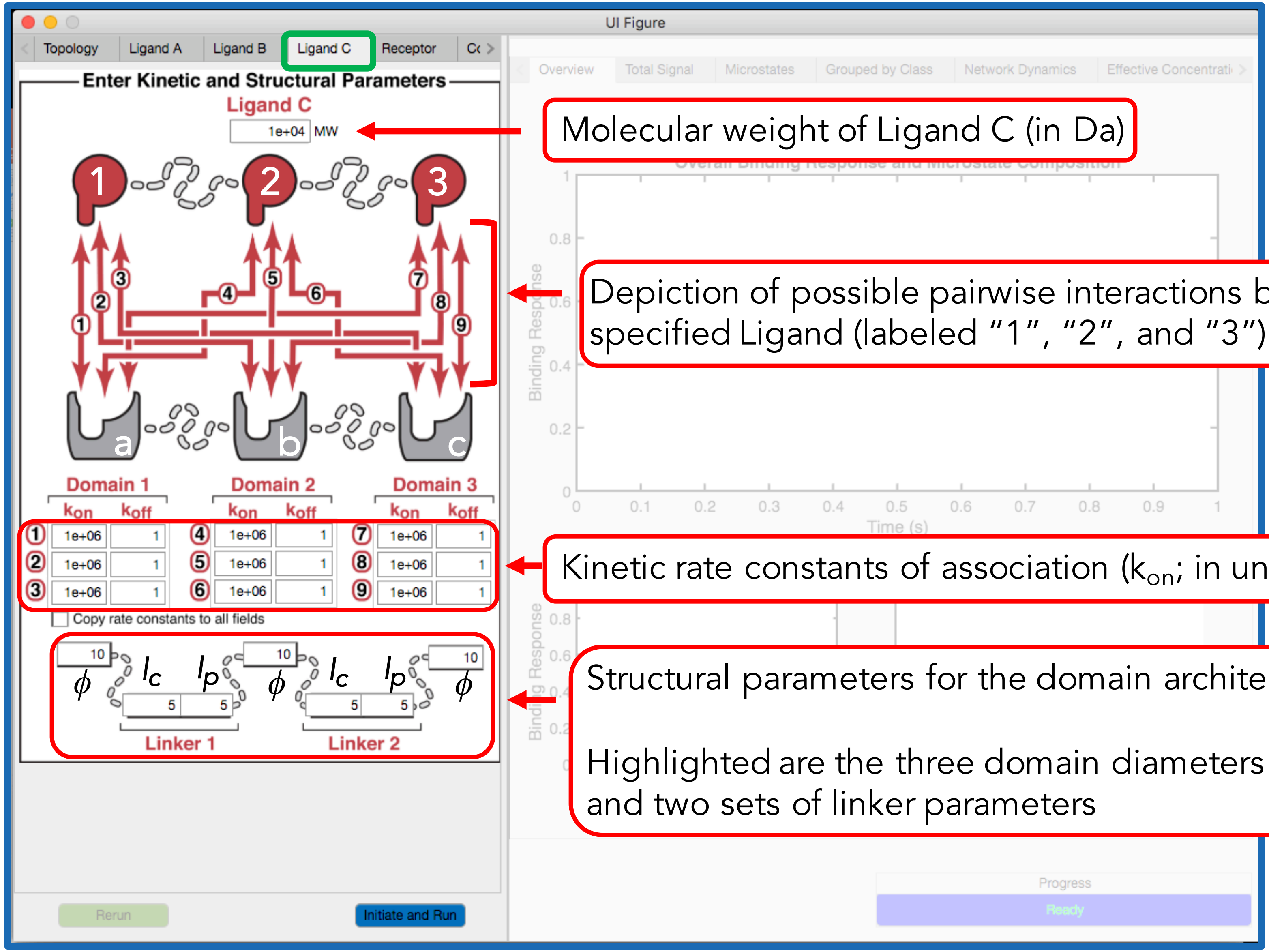

Molecular weight of Ligand C (in Da)

Depiction of possible pairwise interactions between the binding domains of the specified Ligand (labeled "1", "2", and "3") and Receptor (labeled "a", "b", and "c")

Kinetic rate constants of association (k_{on}; in units of $M^{-1}s^{-1}$) and dissociation (k_{off}; in units of s^{-1})

Structural parameters for the domain architecture of a trivalent Ligand C

Highlighted are the three domain diameters (ϕ, units of Angstrom) and two sets of linker parameters

e. User input parameterization of the Receptor

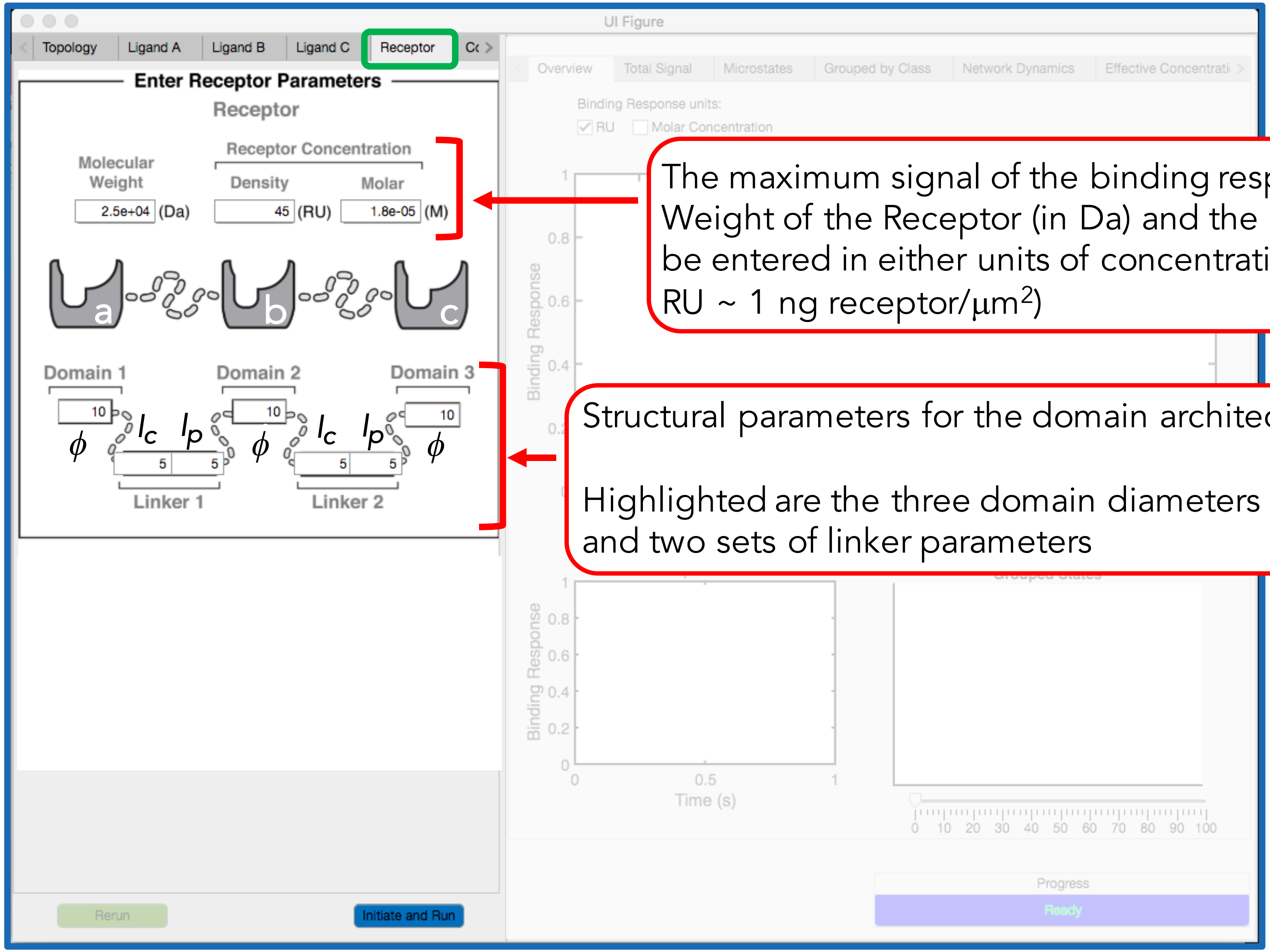

The maximum signal of the binding response is related to the Molecular Weight of the Receptor (in Da) and the Receptor Concentration, which can be entered in either units of concentration (M) or surface density (where 1 RU ~ 1 ng receptor/μm^2)

Structural parameters for the domain architecture of a trivalent Receptor

Highlighted are the three domain diameters (ϕ, units of Angstrom) and two sets of linker parameters

f. User input parameterization of the Ligand Concentrations

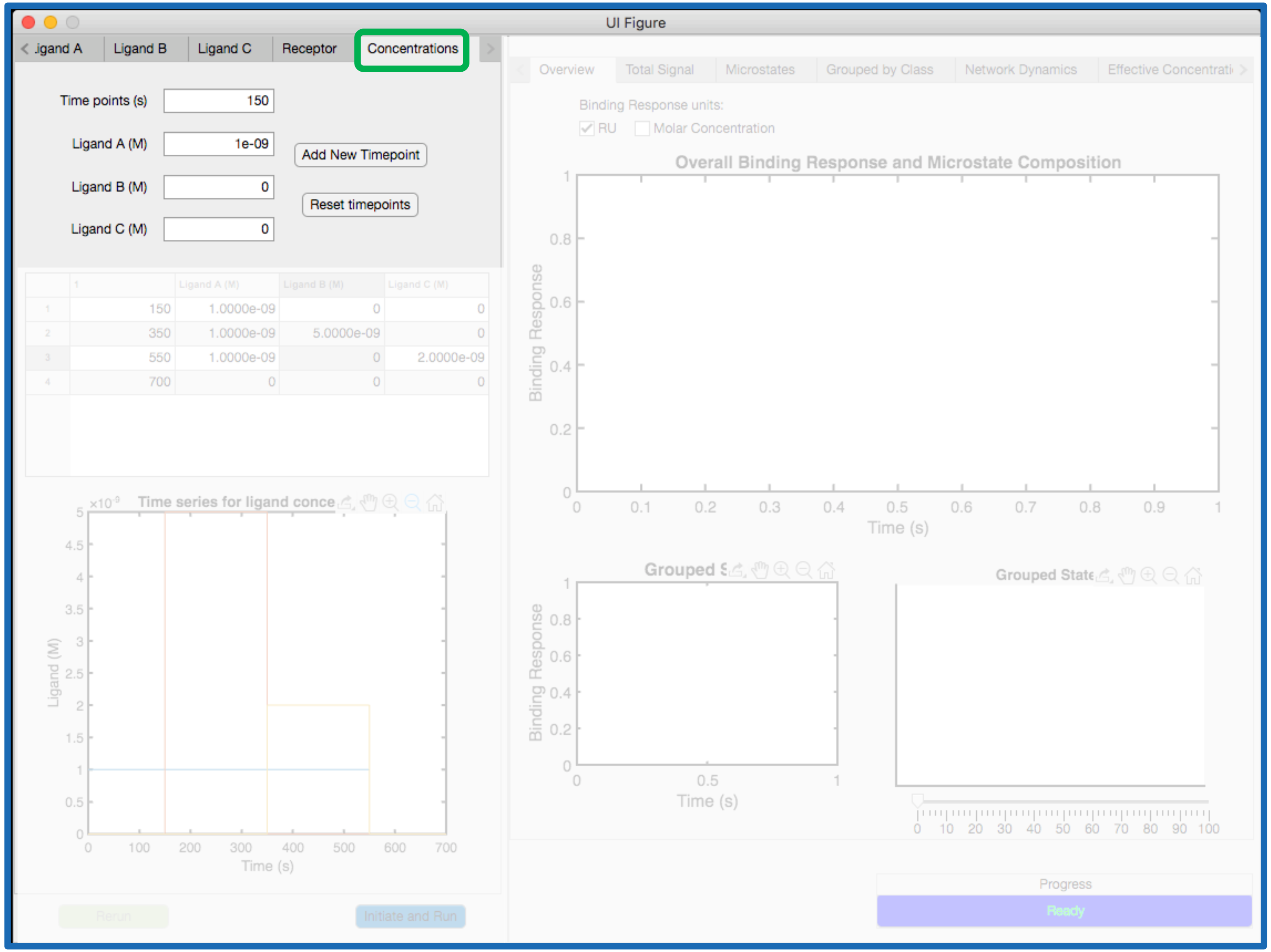

f. User input parameterization of the Ligand Concentrations

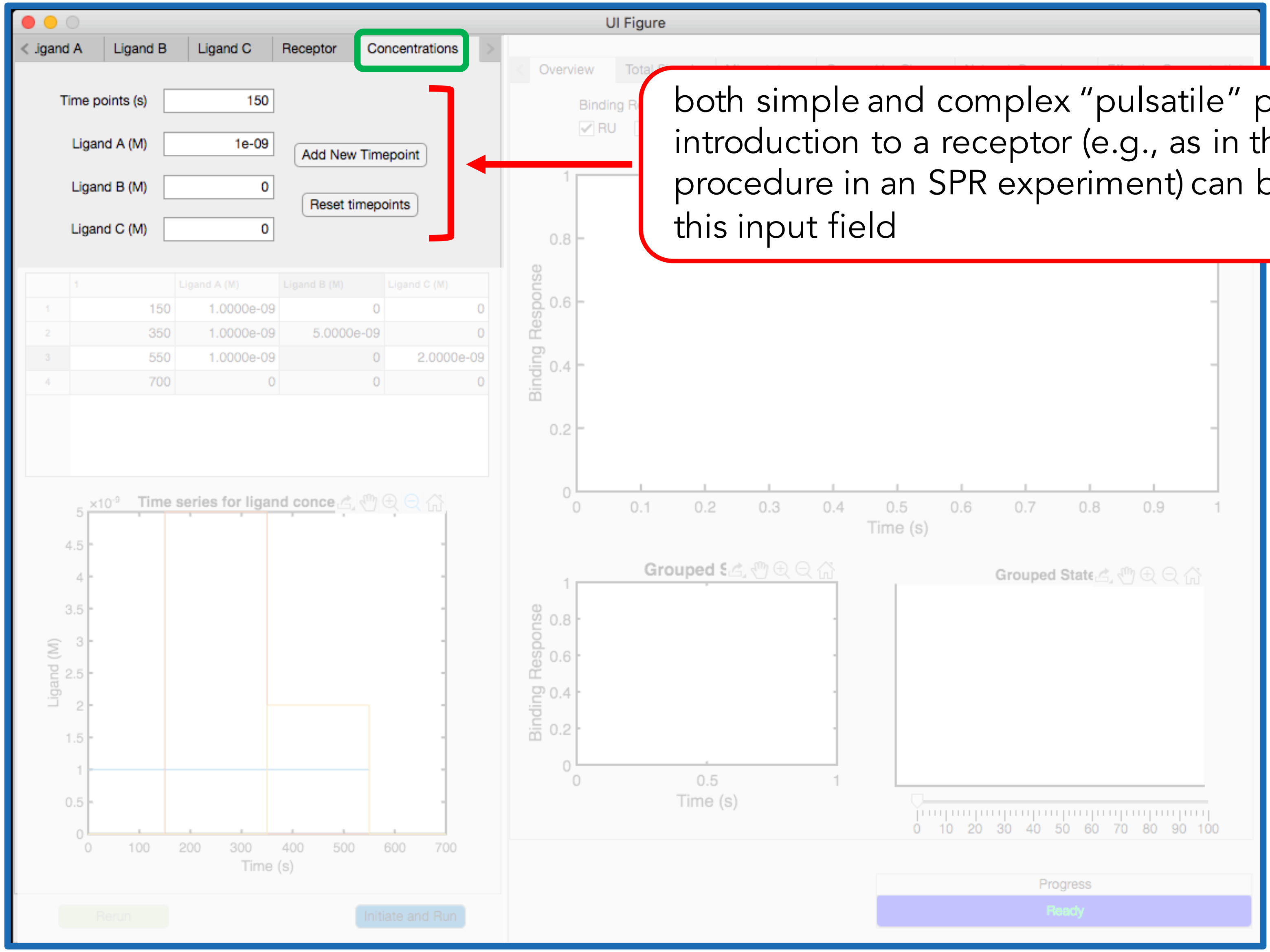

		Ligand A (M)	Ligand B (M)	Ligand C (M)
1	150	1.0000e-09	0	0
2	350	1.0000e-09	5.0000e-09	0
3	550	1.0000e-09	0	2.0000e-09
4	700	0	0	0

both simple and complex "pulsatile" patterns of ligand introduction to a receptor (e.g., as in the analyte injection procedure in an SPR experiment) can be specified through this input field

f. User input parameterization of the Ligand Concentrations

the “Time points” field specifies the breakpoint at which a specified ligand “injection” ends

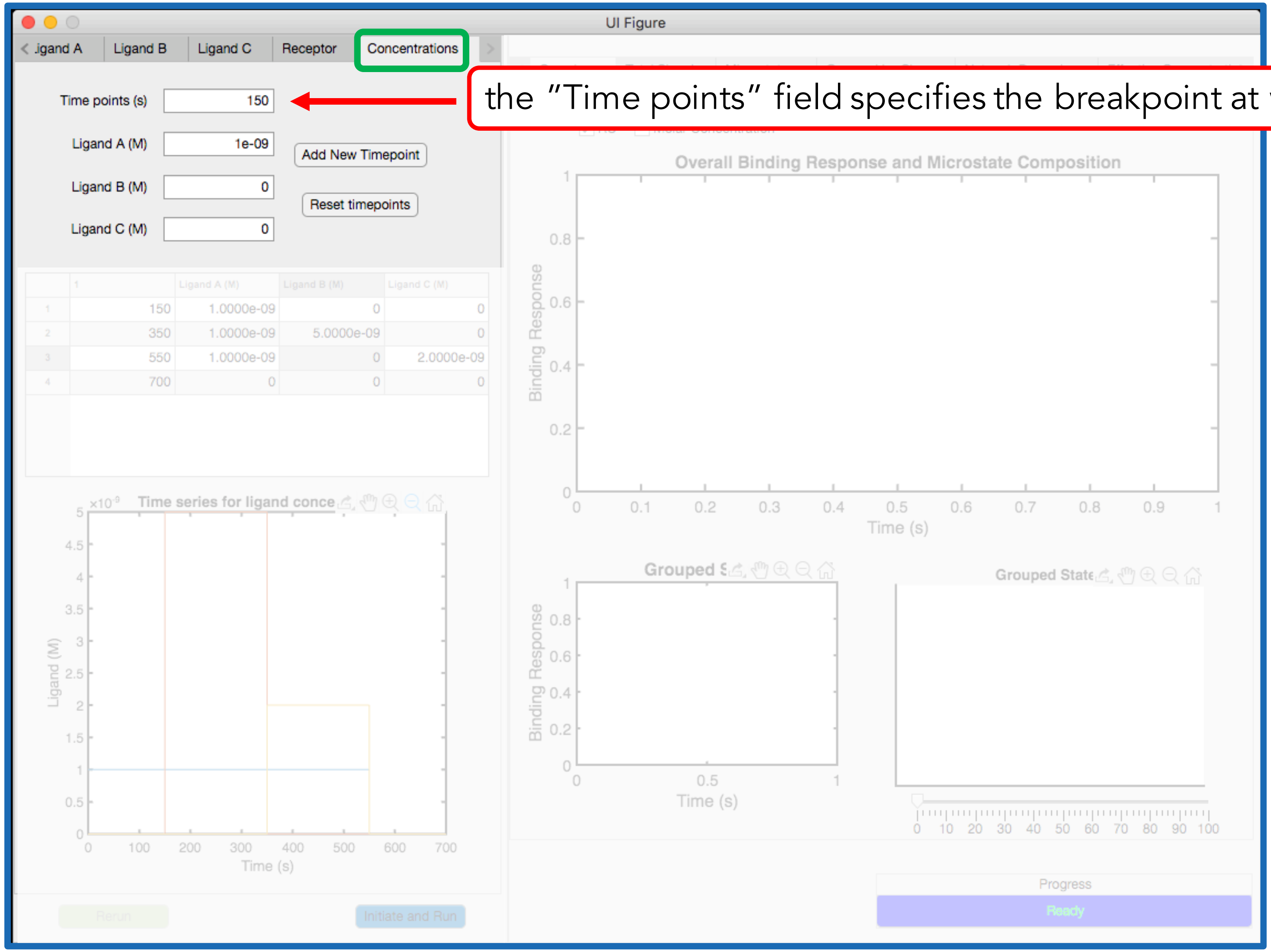

f. User input parameterization of the Ligand Concentrations

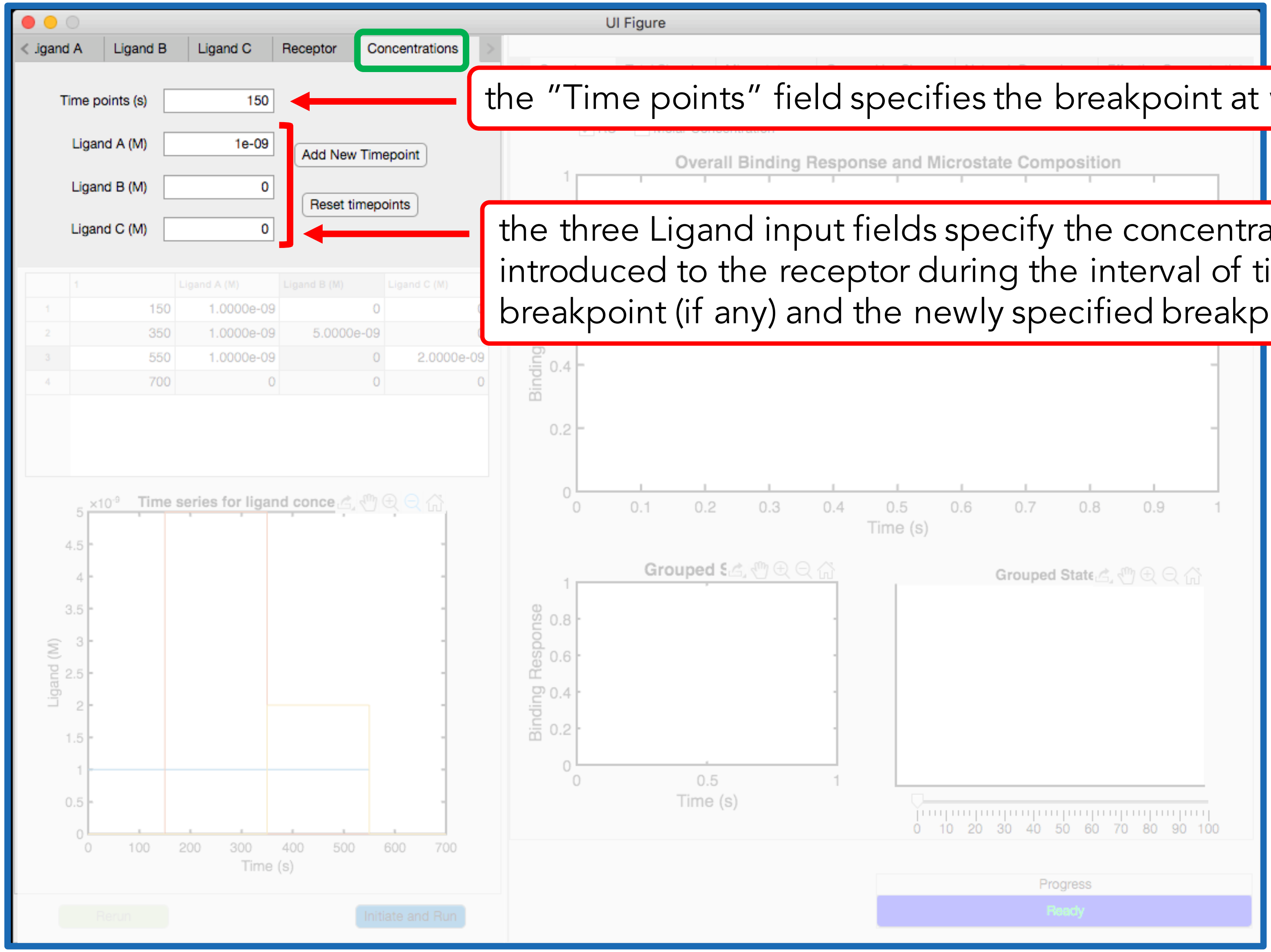

the "Time points" field specifies the breakpoint at which a specified ligand "injection" ends

the three Ligand input fields specify the concentrations of each ligand to be introduced to the receptor during the interval of time between the previous breakpoint (if any) and the newly specified breakpoint (e.g., 150 s, as above)

f. User input parameterization of the Ligand Concentrations

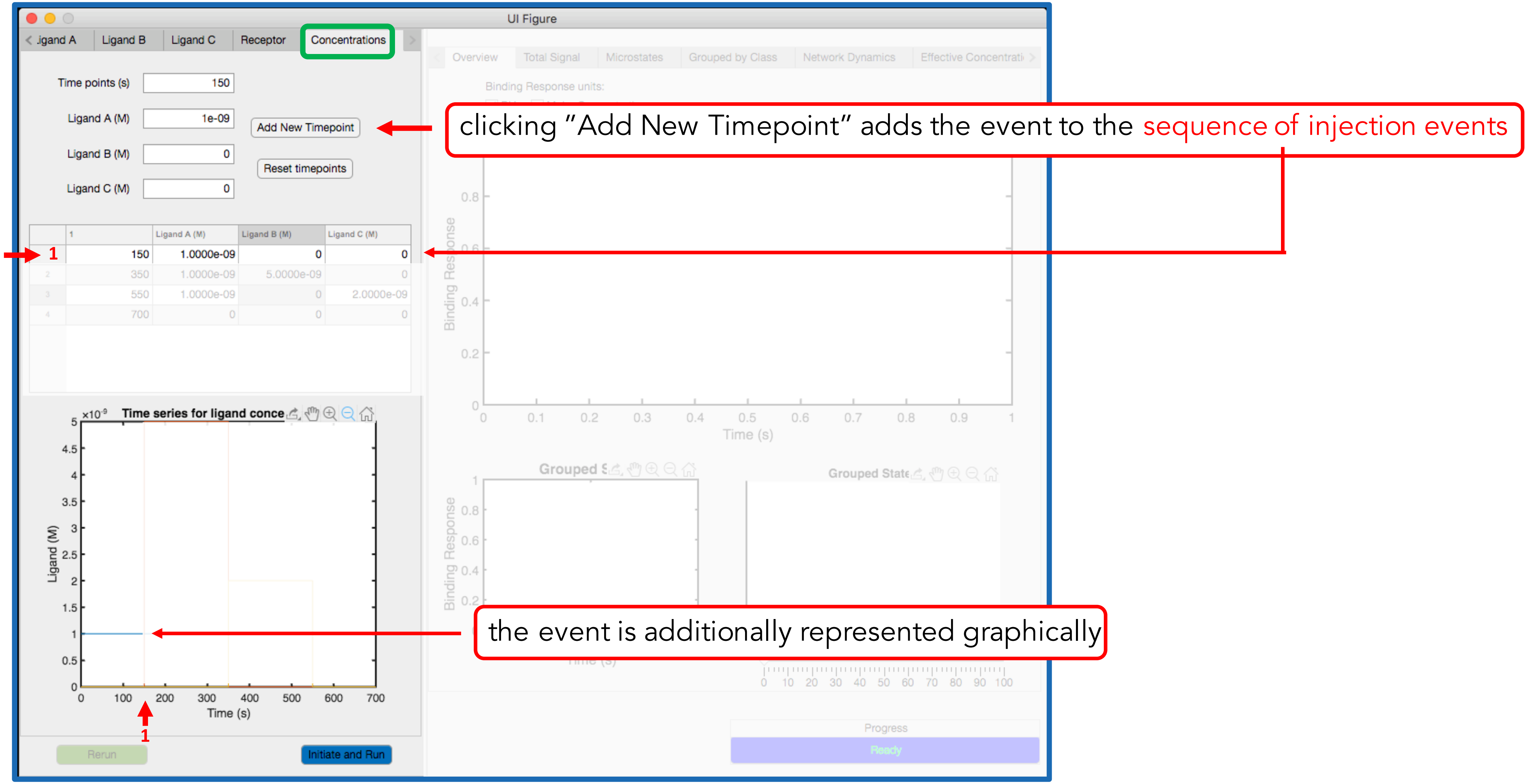

f. User input parameterization of the Ligand Concentrations

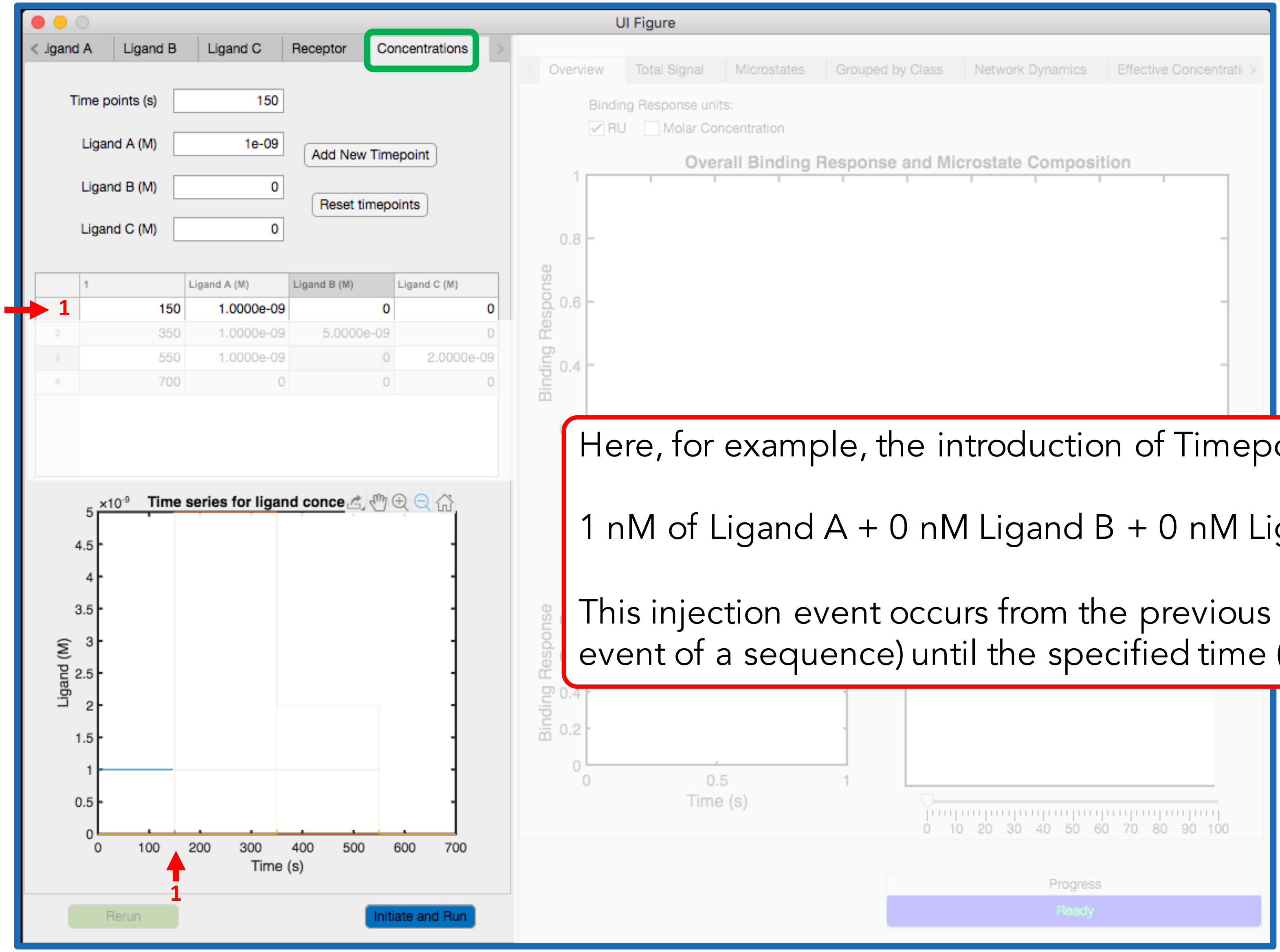

	1	Ligand A (M)	Ligand B (M)	Ligand C (M)
1	150	1.0000e-09	0	0
2	350	1.0000e-09	5.0000e-09	0
3	550	1.0000e-09	0	2.0000e-09
4	700	0	0	0

Here, for example, the introduction of Timepoint 1 specifies the following event:

1 nM of Ligand A + 0 nM Ligand B + 0 nM Ligand C

This injection event occurs from the previous timepoint (by default, 0 s for the first event of a sequence) until the specified time (here, 150 s)

f. User input parameterization of the Ligand Concentrations

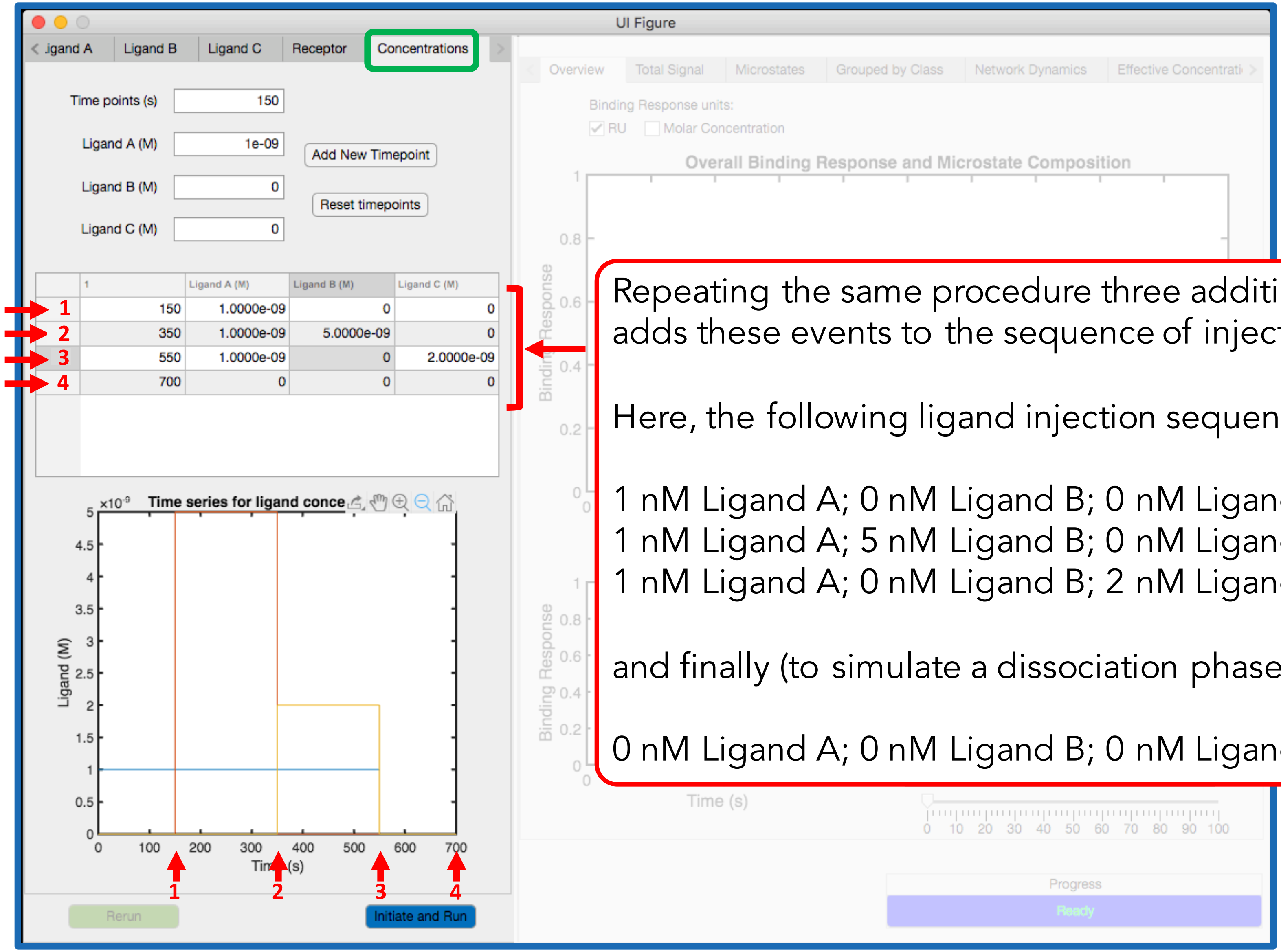

	1	Ligand A (M)	Ligand B (M)	Ligand C (M)
1	150	1.0000e-09	0	0
2	350	1.0000e-09	5.0000e-09	0
3	550	1.0000e-09	0	2.0000e-09
4	700	0	0	0

Repeating the same procedure three additional times (@ 350 s, 550 s, and 700 s) adds these events to the sequence of injections

Here, the following ligand injection sequence is programmed:

1 nM Ligand A; 0 nM Ligand B; 0 nM Ligand C; from 0 s – 150 s
1 nM Ligand A; 5 nM Ligand B; 0 nM Ligand C; from 150 s – 350 s
1 nM Ligand A; 0 nM Ligand B; 2 nM Ligand C from 350 s – 550 s

and finally (to simulate a dissociation phase),

0 nM Ligand A; 0 nM Ligand B; 0 nM Ligand C; from 550 s – 700 s

f. User input parameterization of the Ligand Concentrations

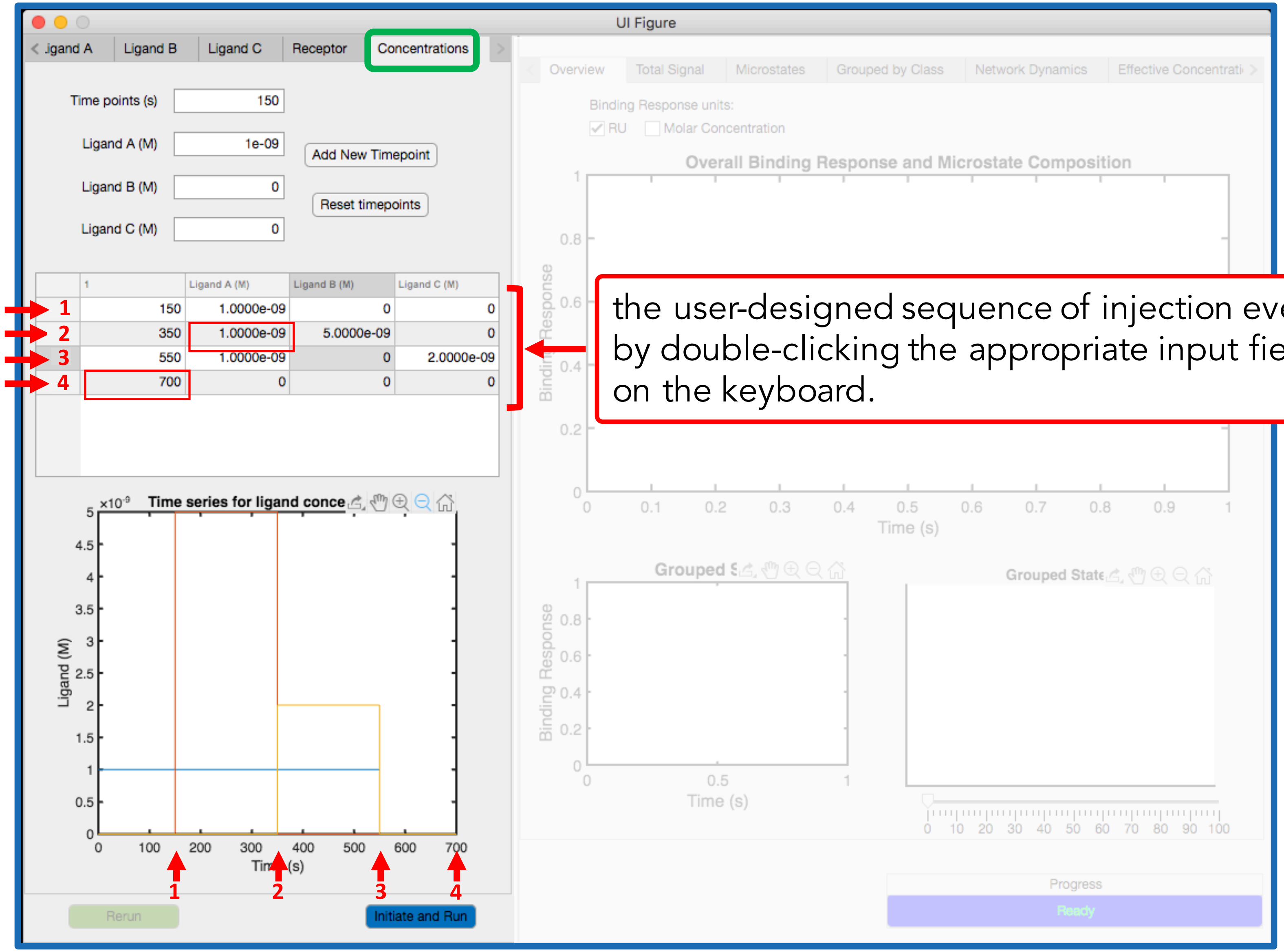

the user-designed sequence of injection events can be manually edited (one-by-one) by double-clicking the appropriate input field, editing the value, and pressing "enter" on the keyboard.

f. User input parameterization of the Ligand Concentrations

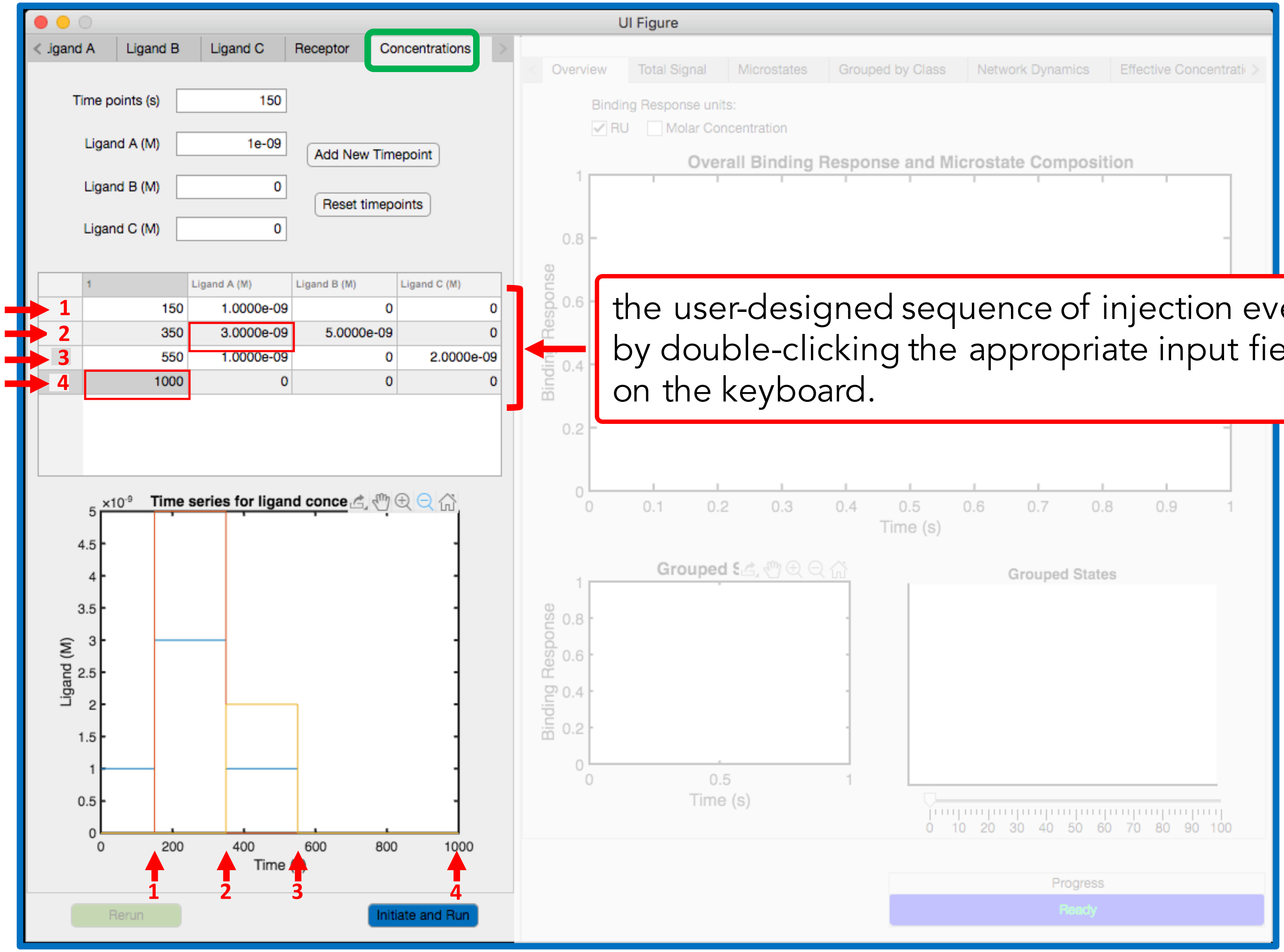

the user-designed sequence of injection events can be manually edited (one-by-one) by double-clicking the appropriate input field, editing the value, and pressing "enter" on the keyboard.

f. User input parameterization of the Ligand Concentrations

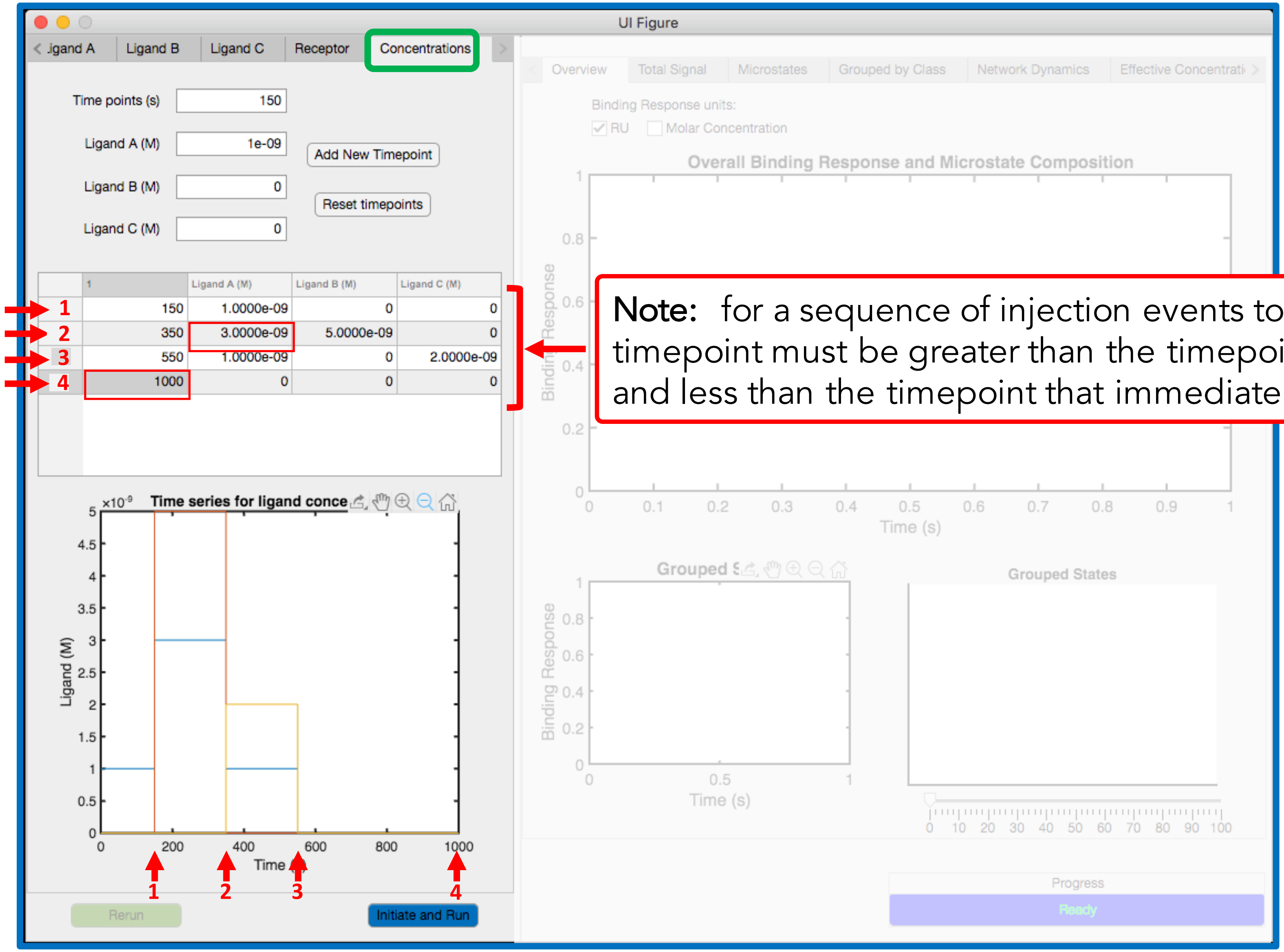

Note: for a sequence of injection events to be valid the designated time for each timepoint must be greater than the timepoint that precedes it (i.e., it must occur after) and less than the timepoint that immediately follows it (i.e., it must occur before)

f. User input parameterization of the Ligand Concentrations

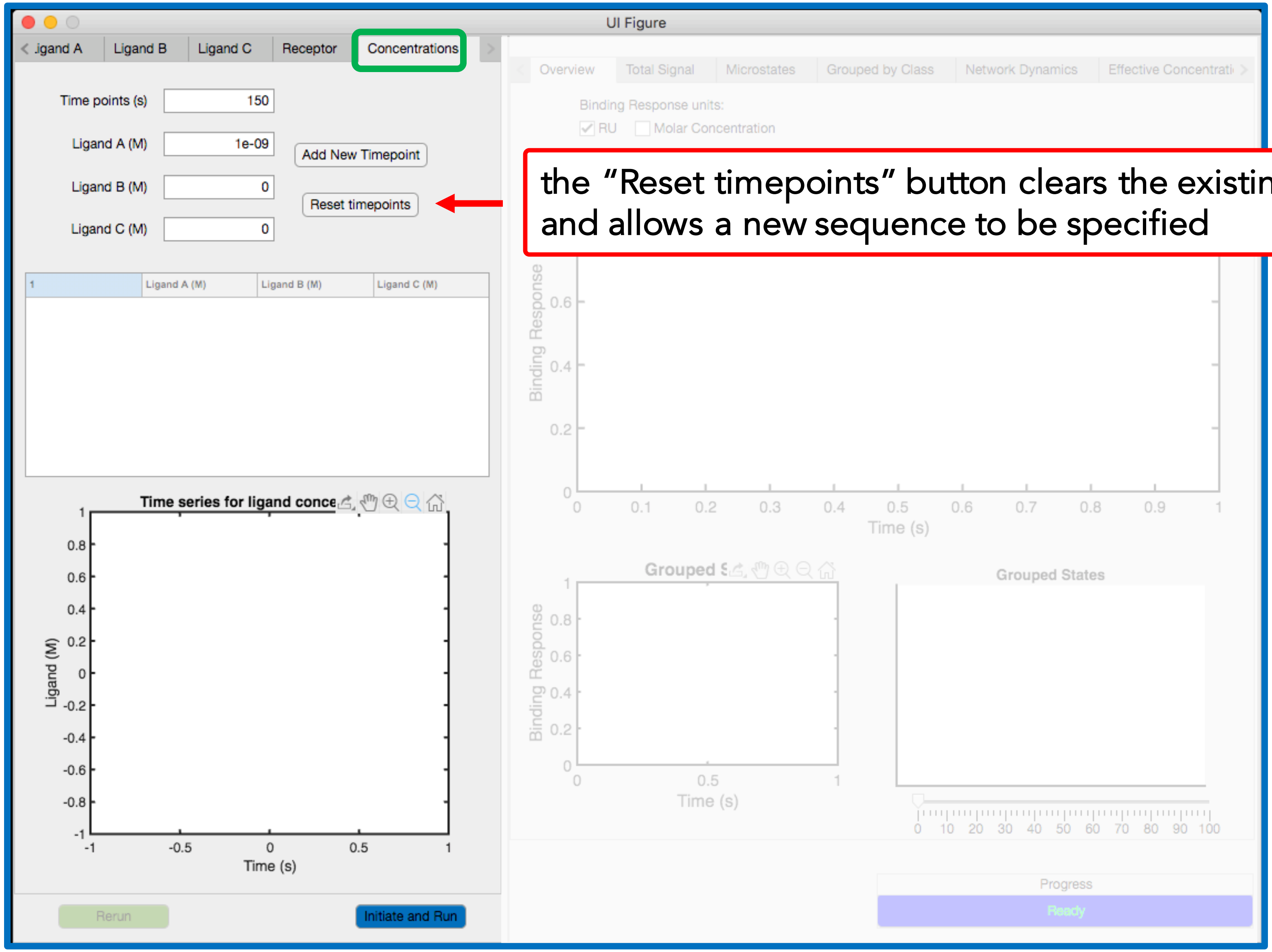

the "Reset timepoints" button clears the existing sequence of injection events and allows a new sequence to be specified

g. Initiate and Run *MVsim*

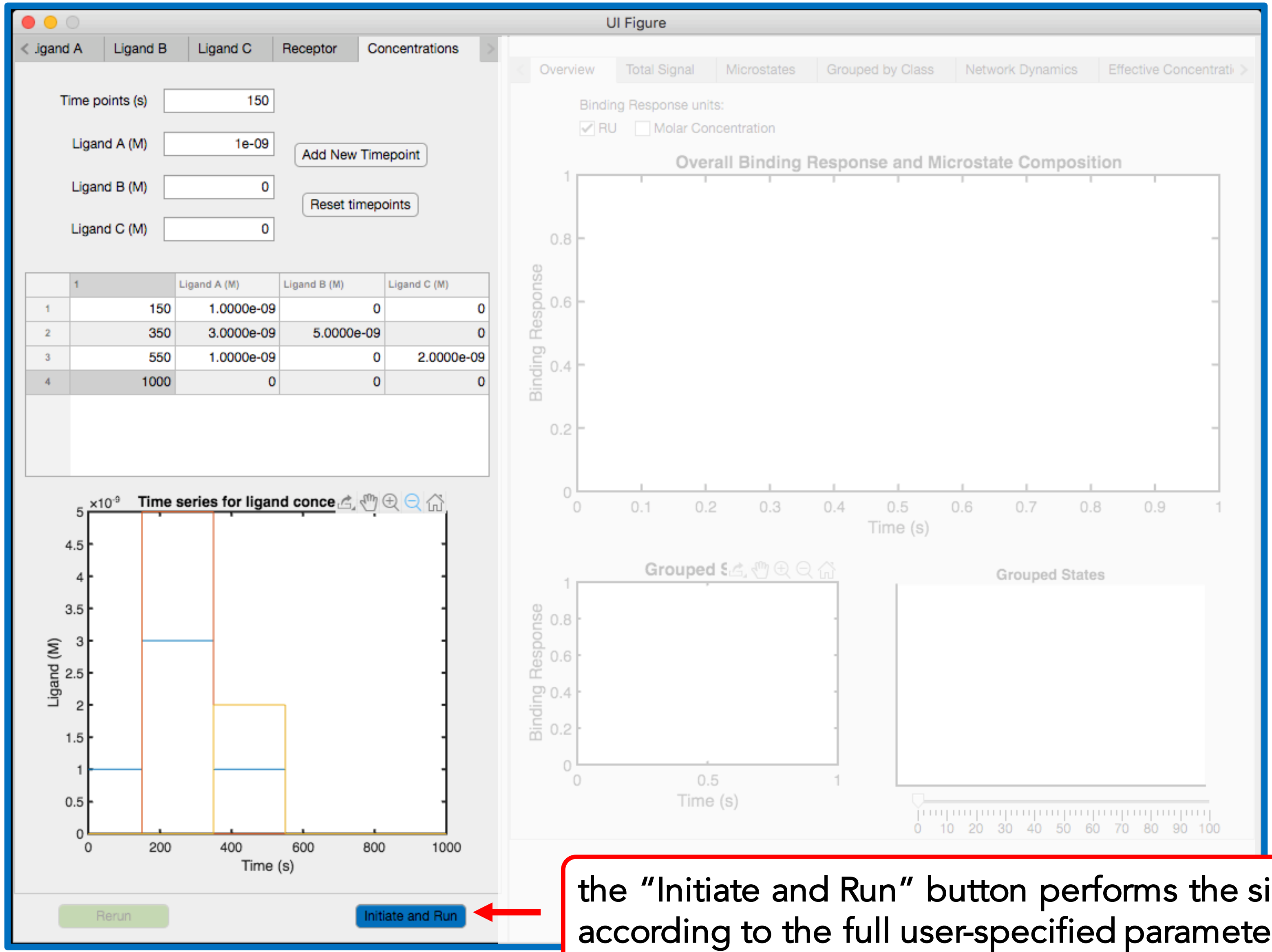

the "Initiate and Run" button performs the simulation according to the full user-specified parameterization

4. Navigating the *MVsim* output tabs

a. Output Overview

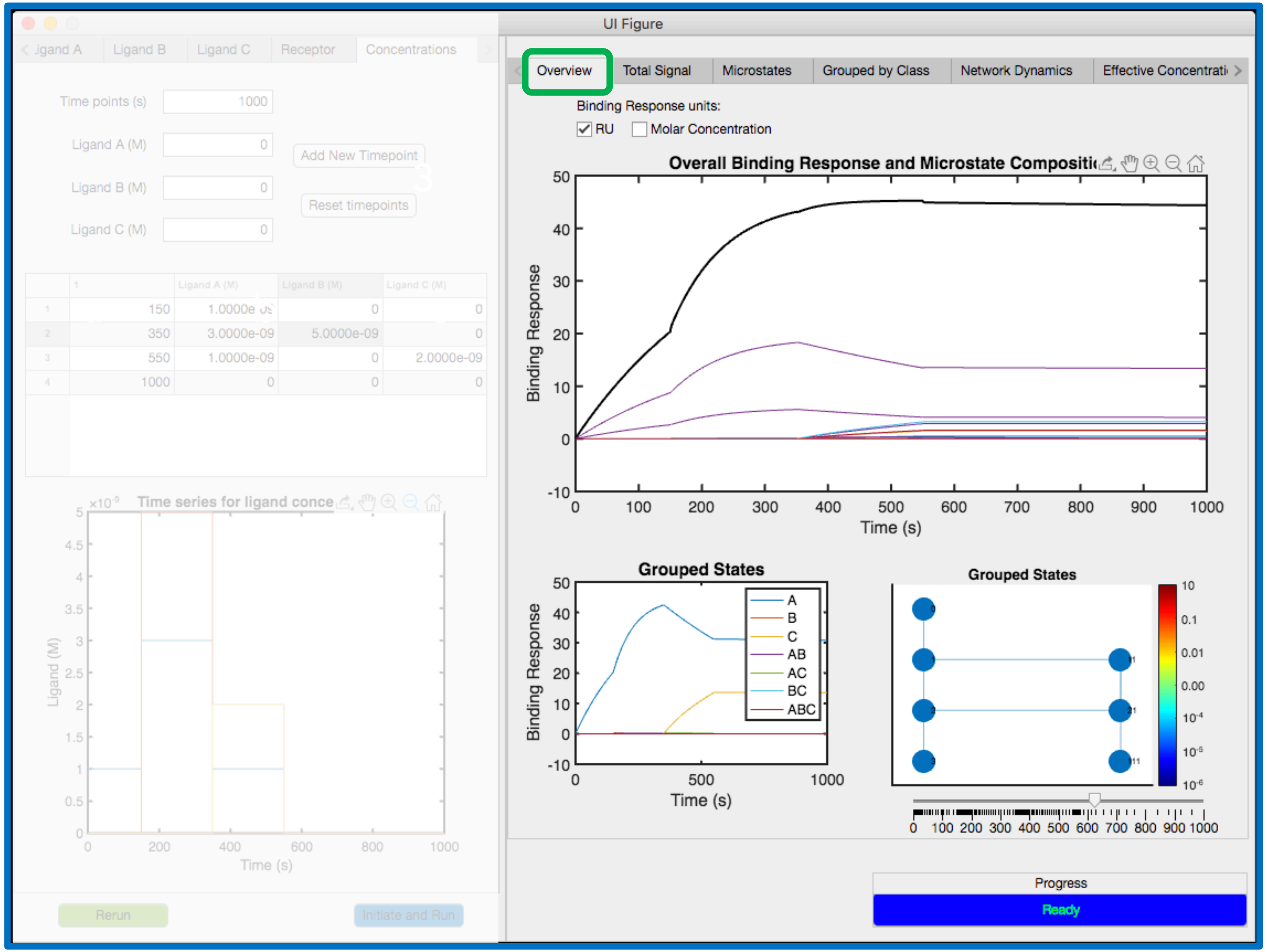

4. Navigating the *MVsim* output tabs

a. Output Overview

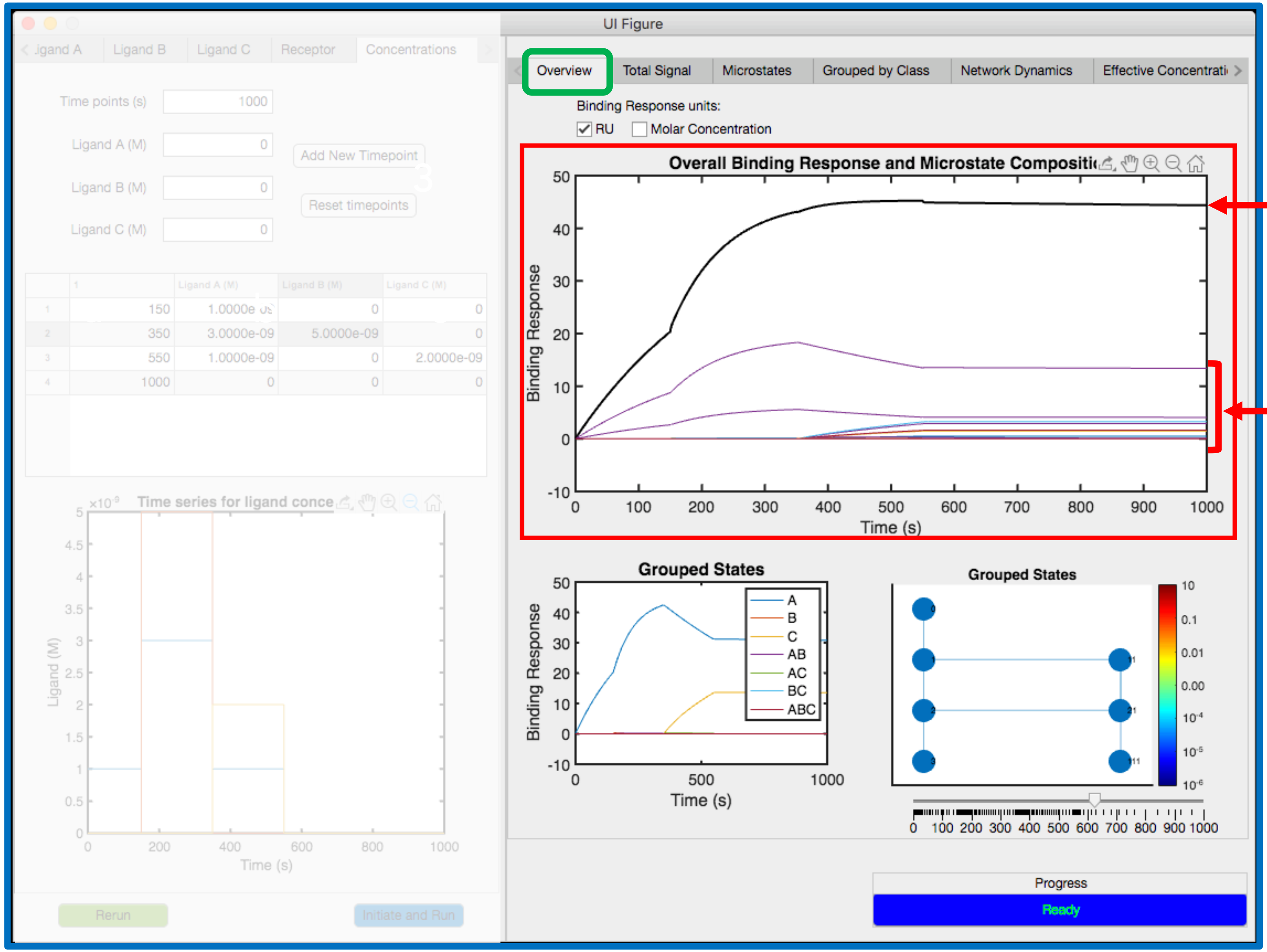

the total binding response dynamics (top black trace) represents the cumulative signal for all binding events that occur during the simulation (i.e., the measurable signal in an SPR experiment)

the conformational microstates (i.e., all monovalent, bivalent, and trivalent patterns of configuration that the multivalent and/or multi-ligand system populates)

4. Navigating the *MVsim* output tabs

a. Output Overview

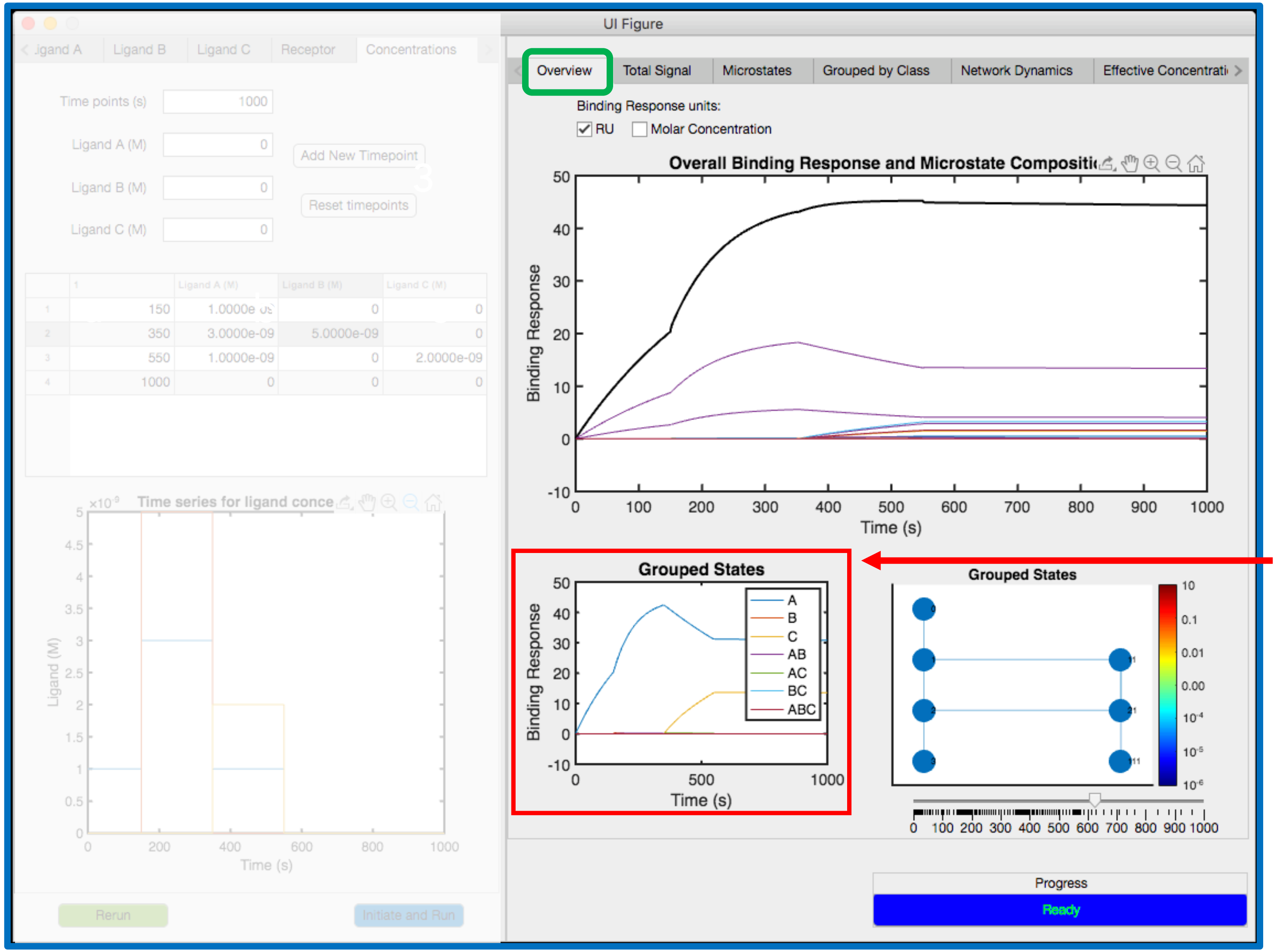

the "Grouped States" plots a simplified view of the overall binding response in which microstates are summed together based on their class. Here, the class represents microstates based on the ligand(s) from which they are composed

E.g., "ABC" represents the binding response from all microstates which entail a receptor bound simultaneously by Ligands A, B, and C

4. Navigating the *MVsim* output tabs

a. Output Overview

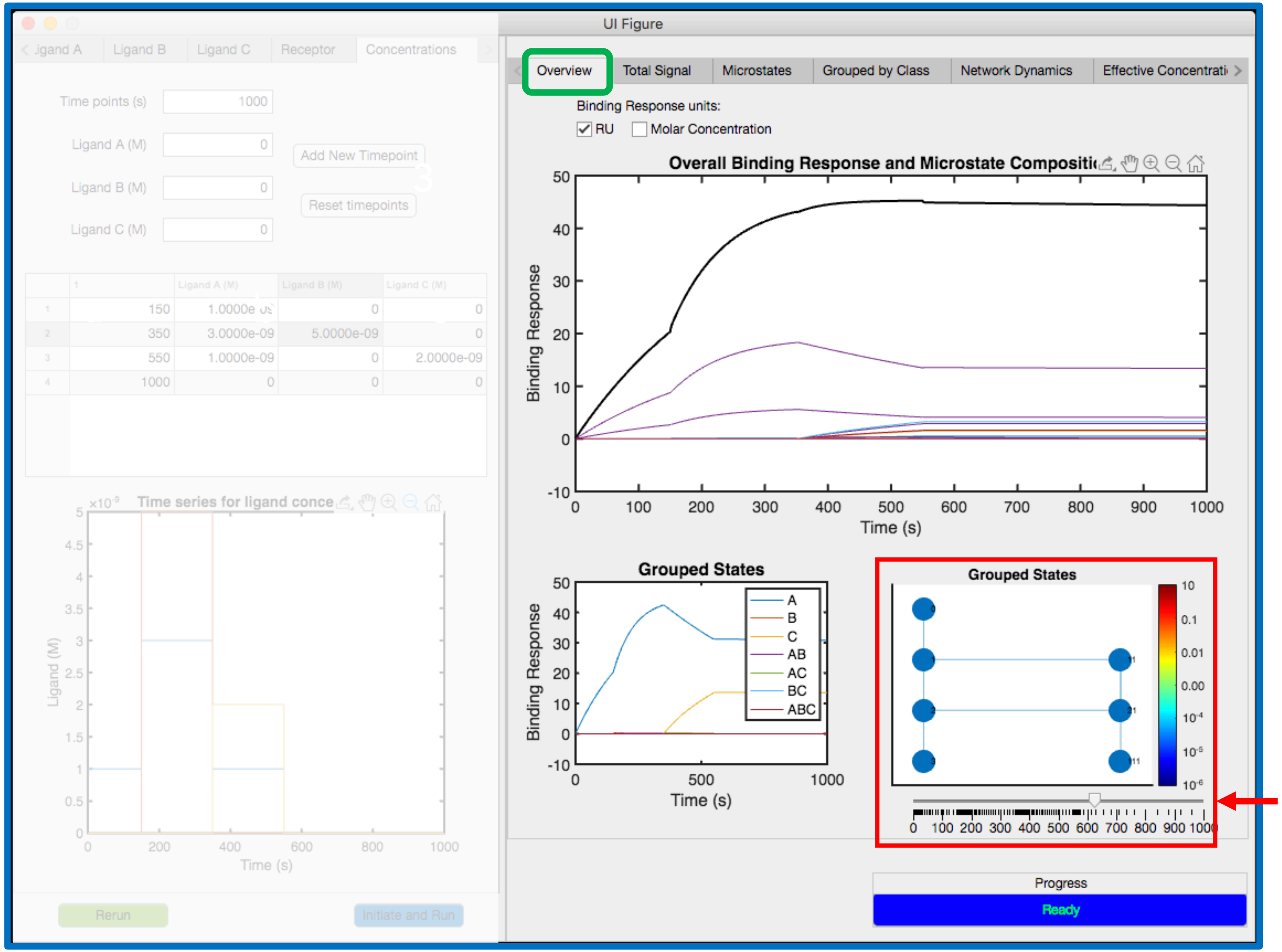

a "click-and-drag" slide bar enables the grouped states to be visualized at individual timepoints sampled during the entire length of the simulated interaction

b. Output Total Signal

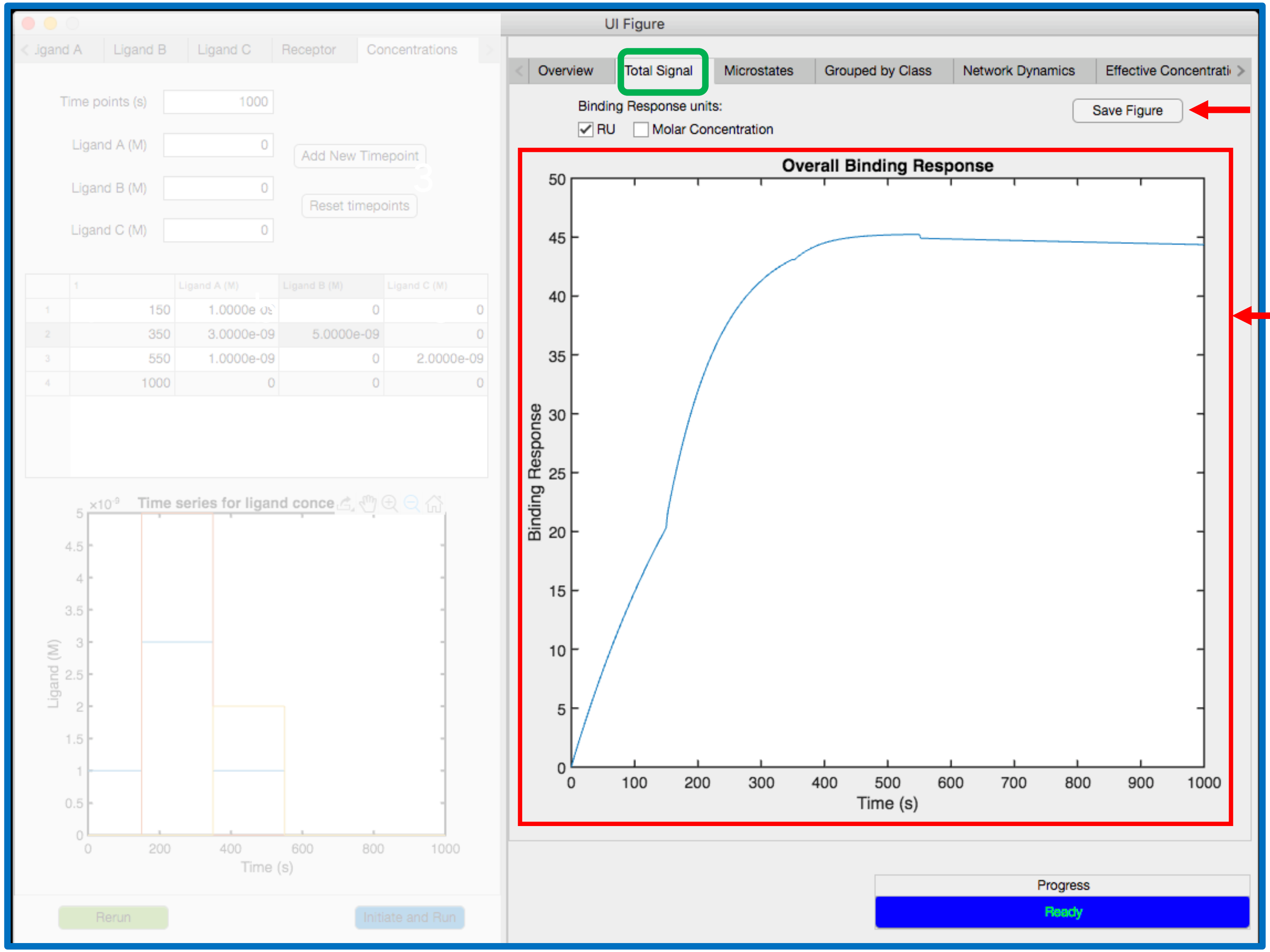

"Save Figure" exports the plot as a .fig file

single view of the overall binding response (i.e., the black trace from the Overview tab)

4. Navigating the *MVsim* output tabs

b. Output Total Signal

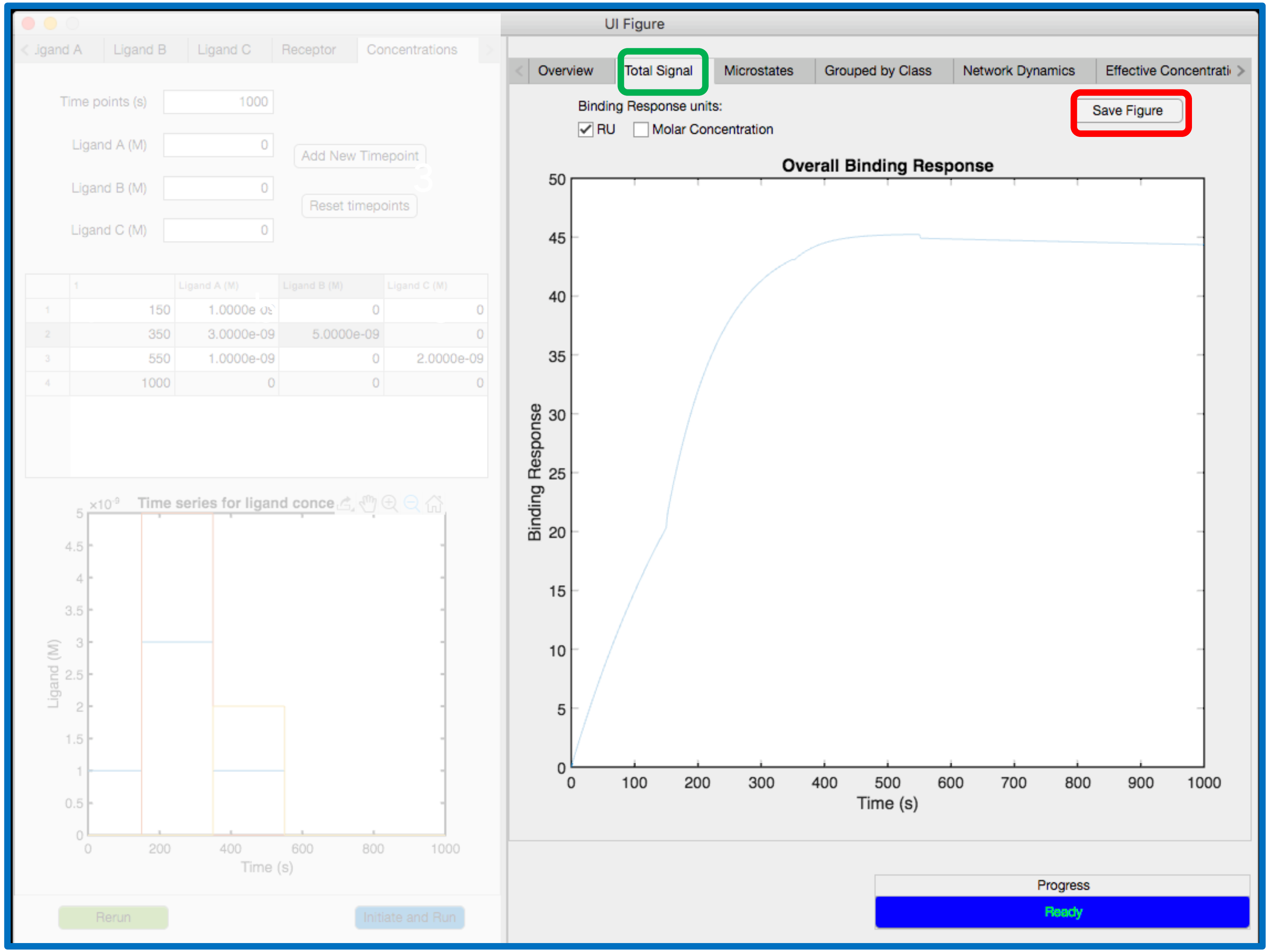

"Save Figure" exports the plot as a .fig file

4. Navigating the *MVsim* output tabs

b. Output Total Signal

"Save Figure" exports the plot as a .fig file

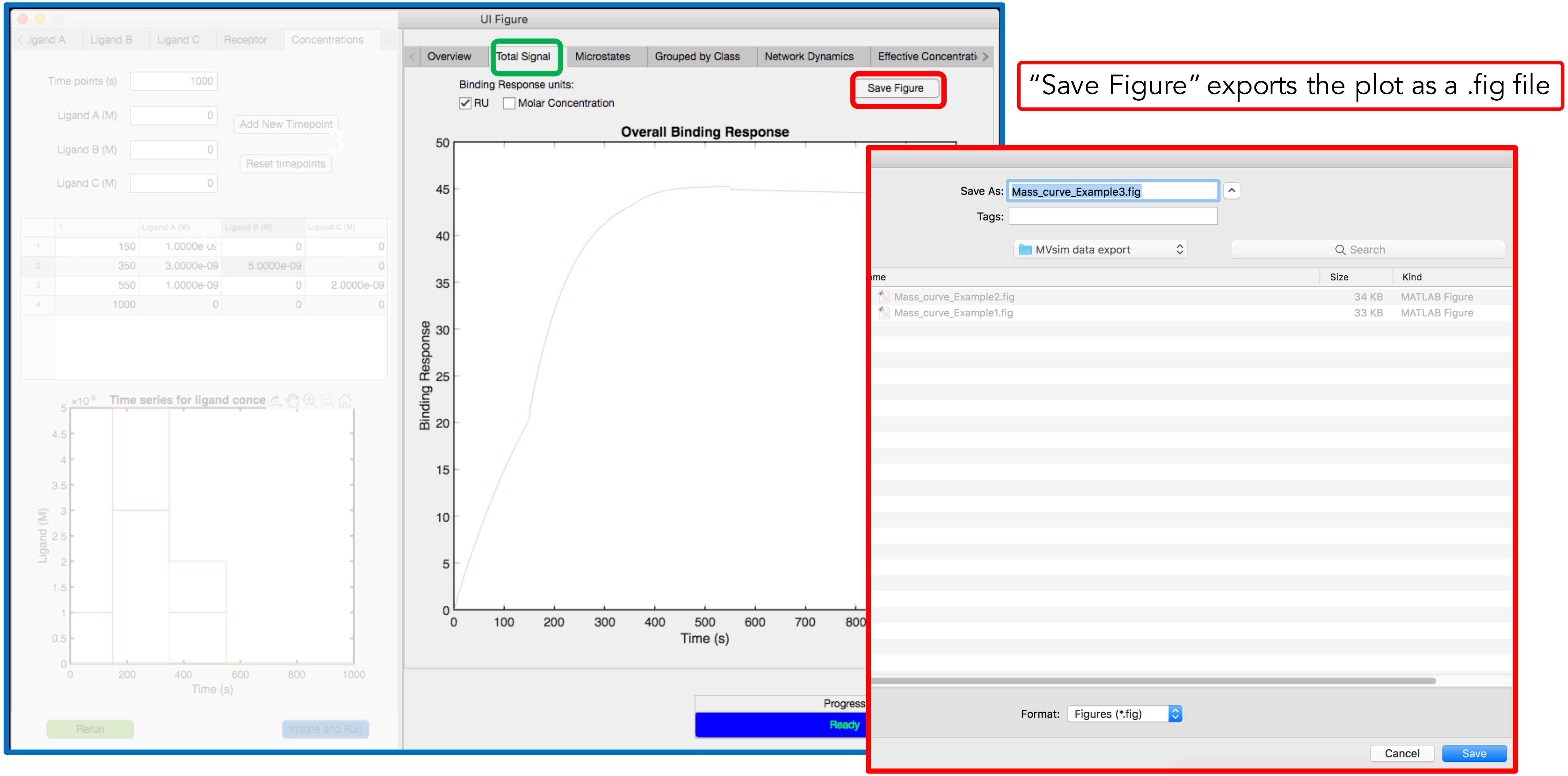

b. Output Total Signal

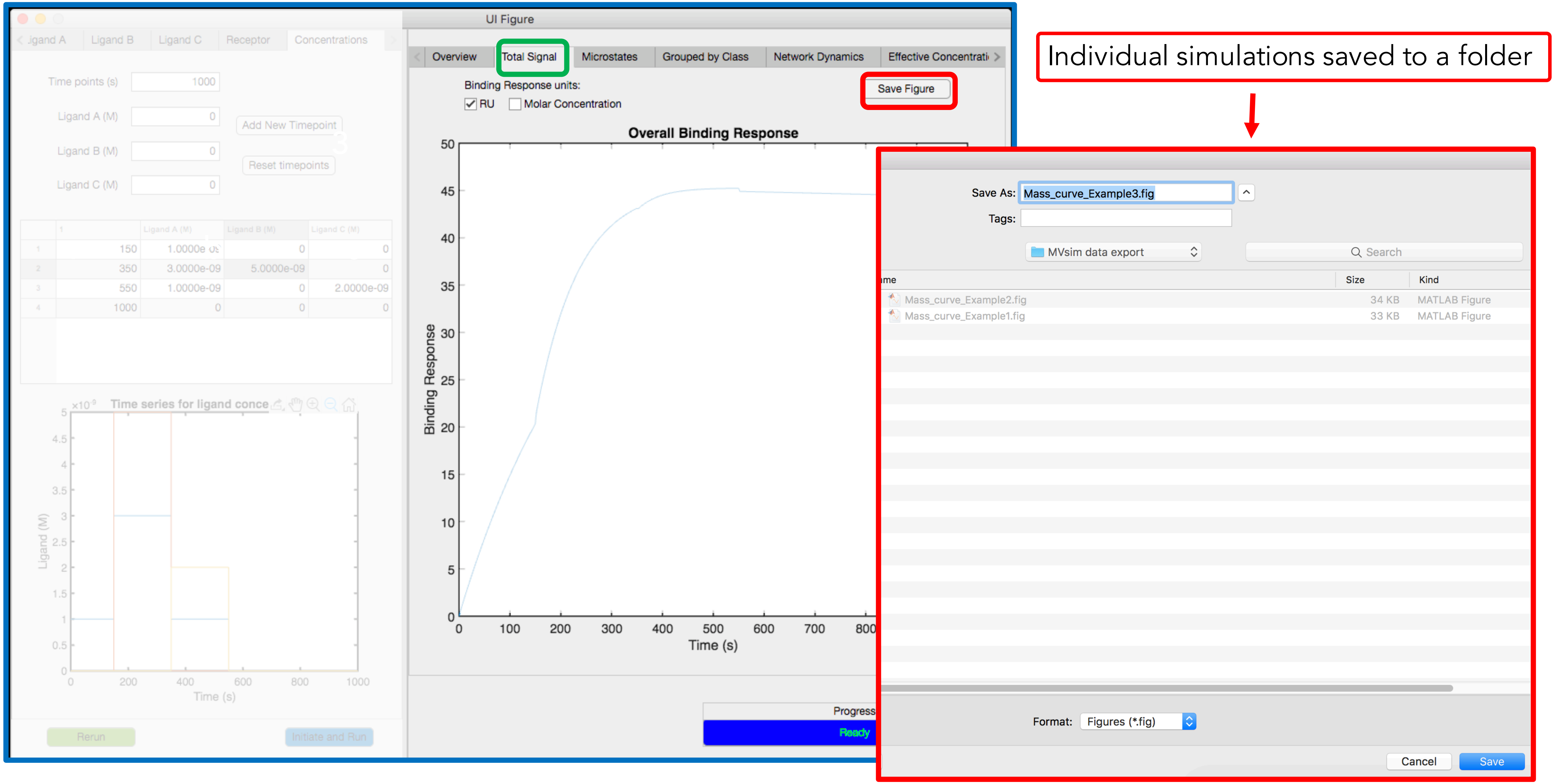

Individual simulations saved to a folder

b. Output Total Signal

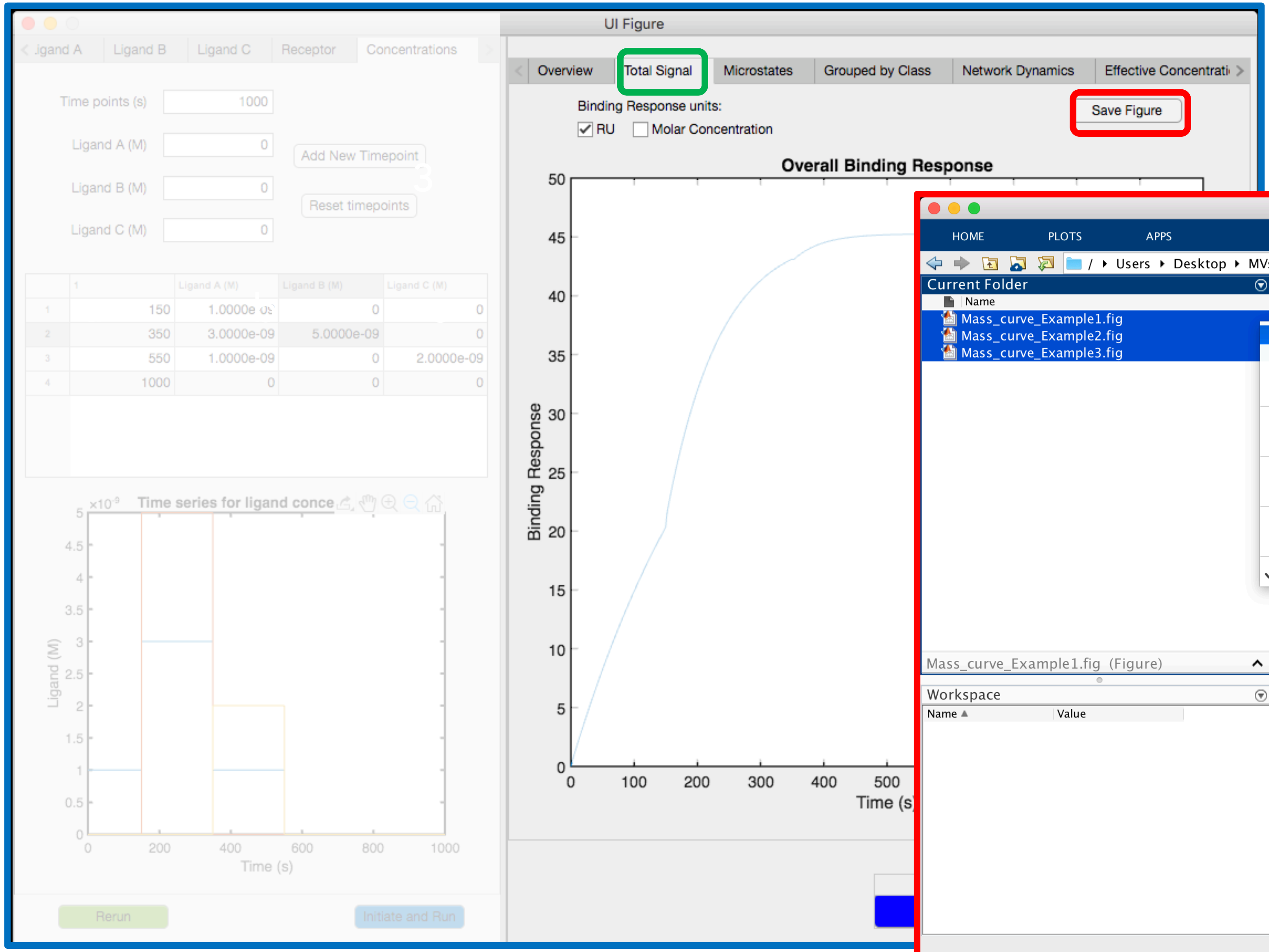

Navigate to the export folder in MATLAB and batch open .fig files

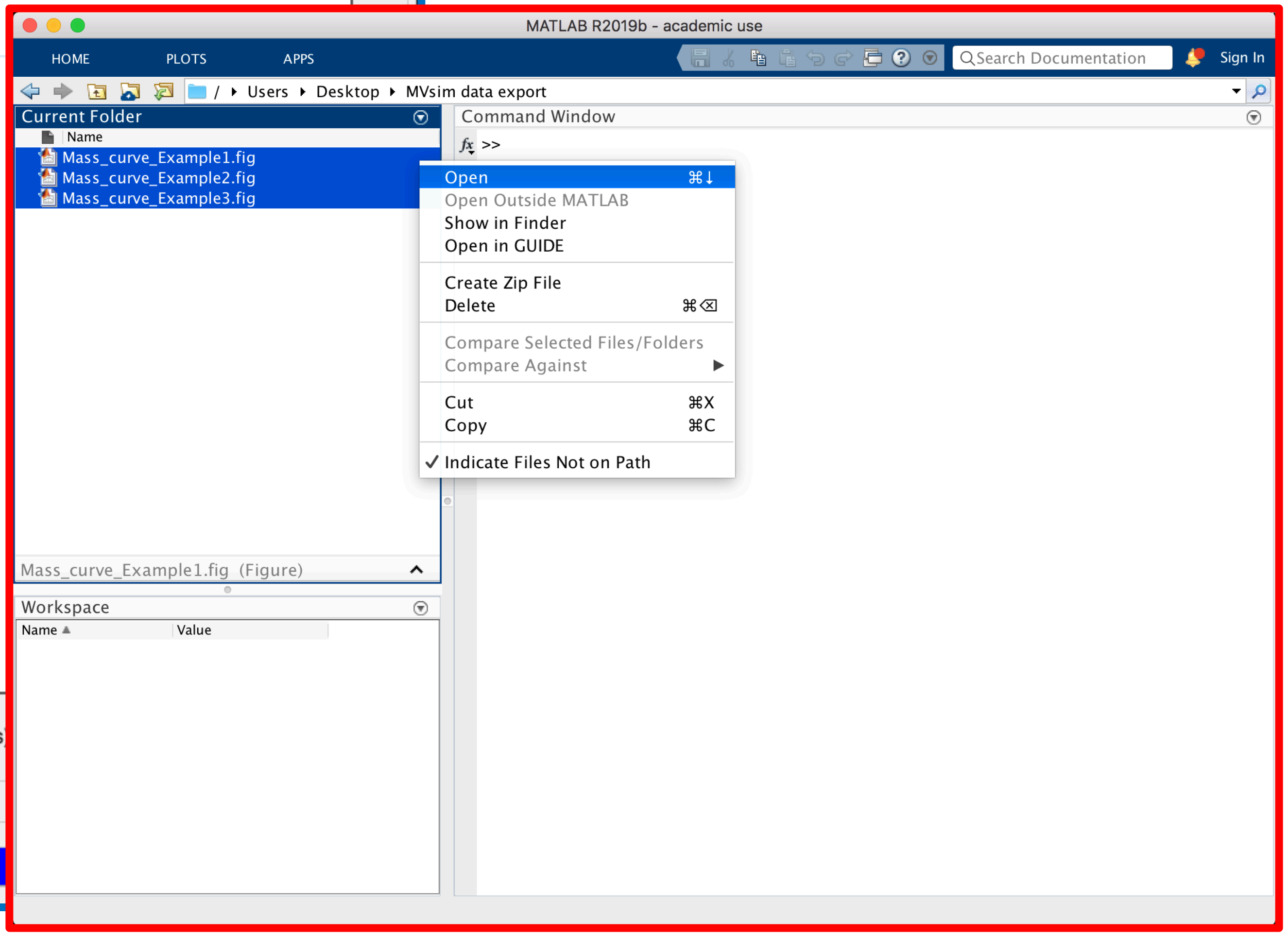

b. Output Total Signal

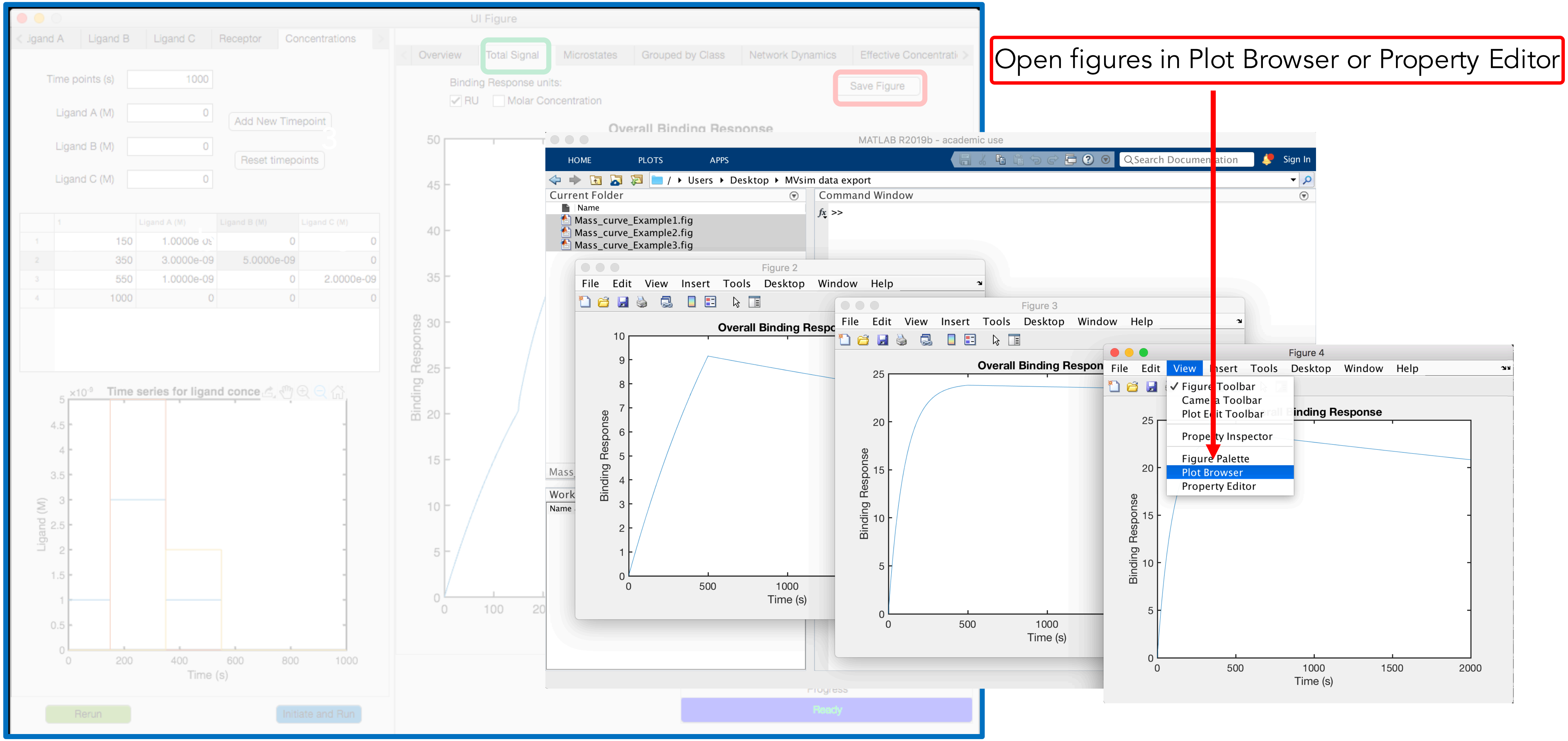

b. Output Total Signal

Copying and Pasting individual sensorgram curves into a common tab enables batch viewing of multiple superimposed simulations

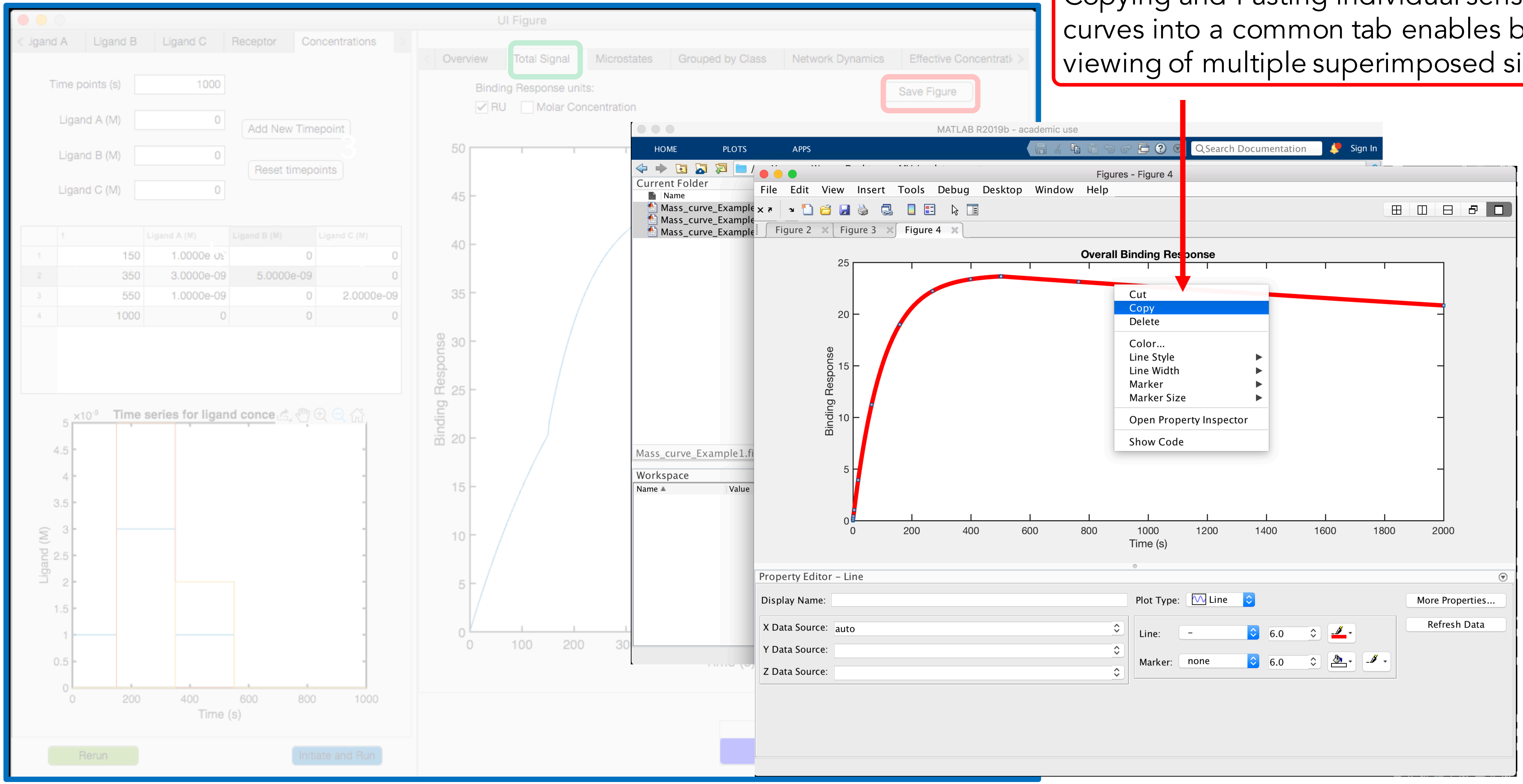

4. Navigating the *MVsim* output tabs

b. Output Total Signal

Overlay batch view of three simulations

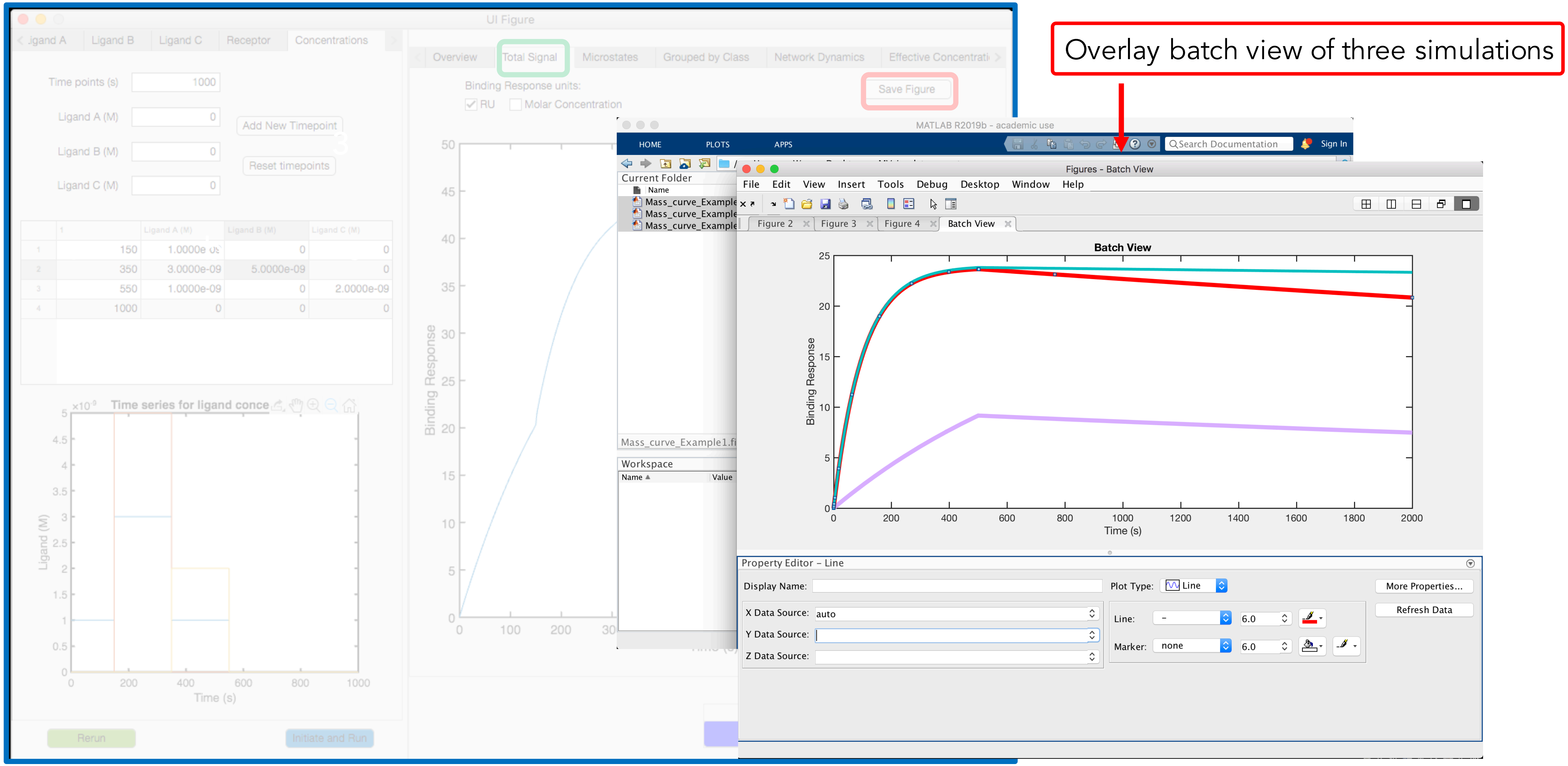

4. Navigating the *MVsim* output tabs

b. Output Total Signal

Extraction of x,y datapoints for exported simulations enables further ‘offline’ analyses

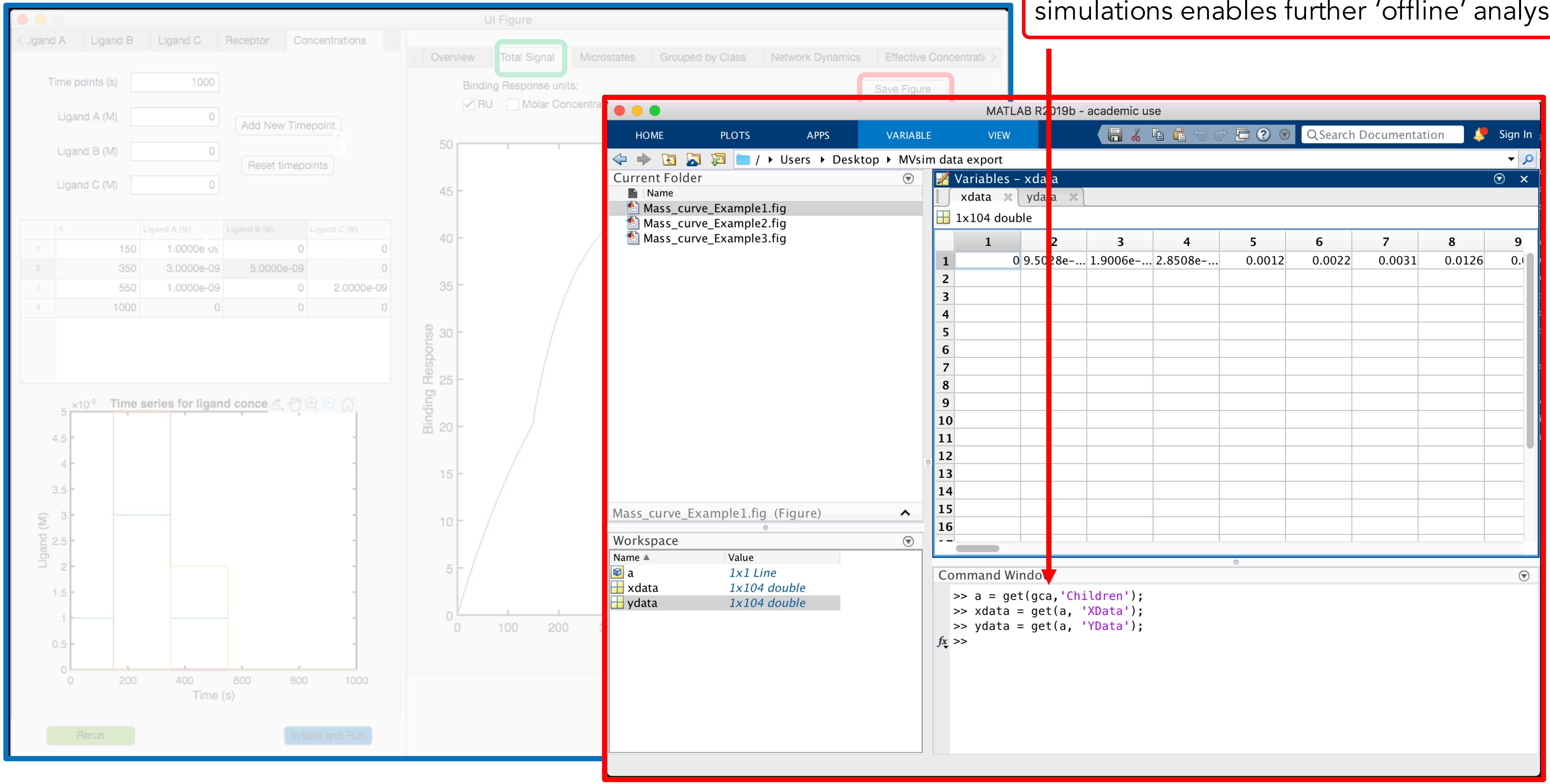

c. Output Microstates

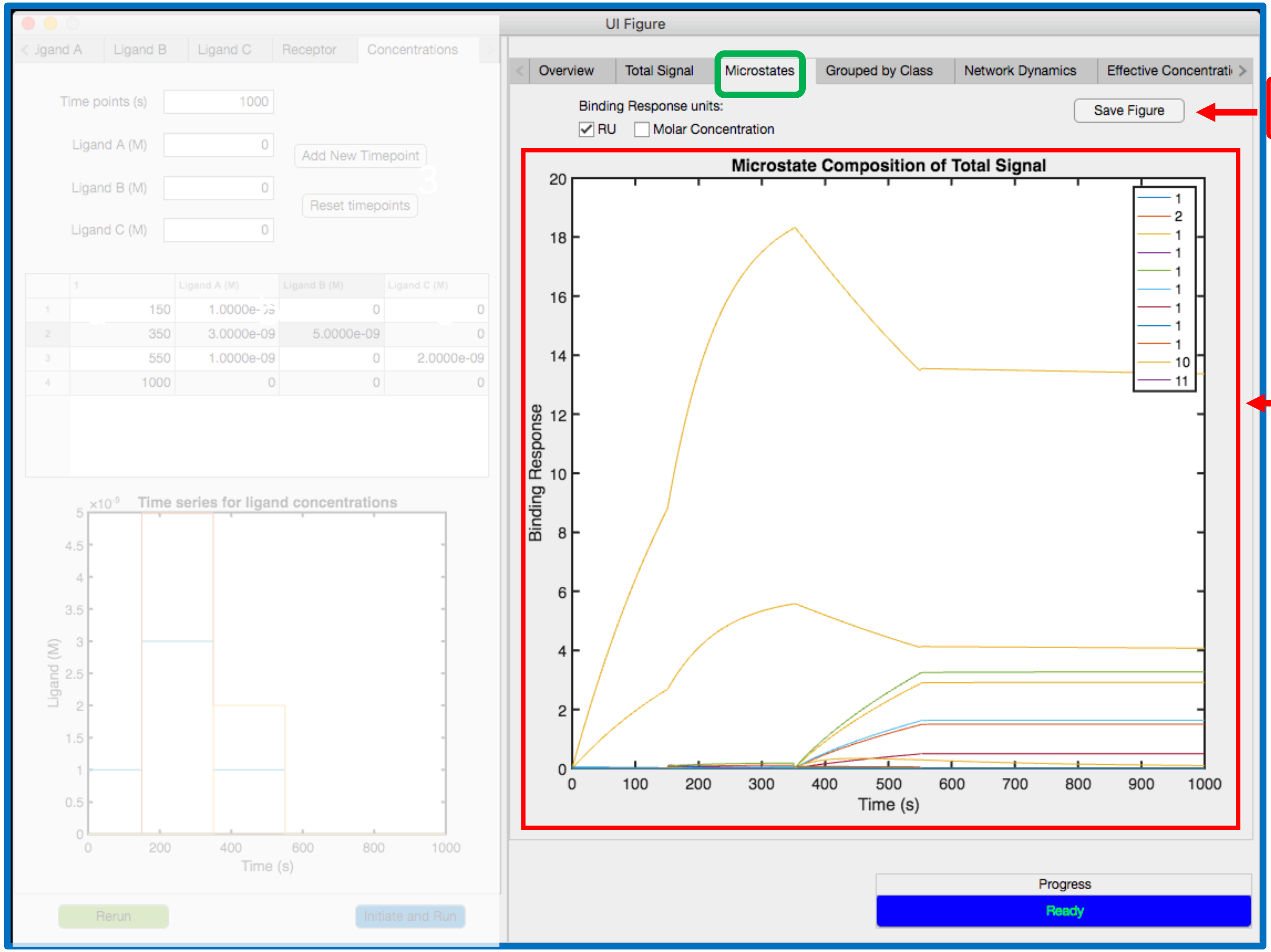

"Save Figure" exports the plot as a .fig file

plot showing all composite microstates that underlie the overall binding response

d. Output Grouped by Class

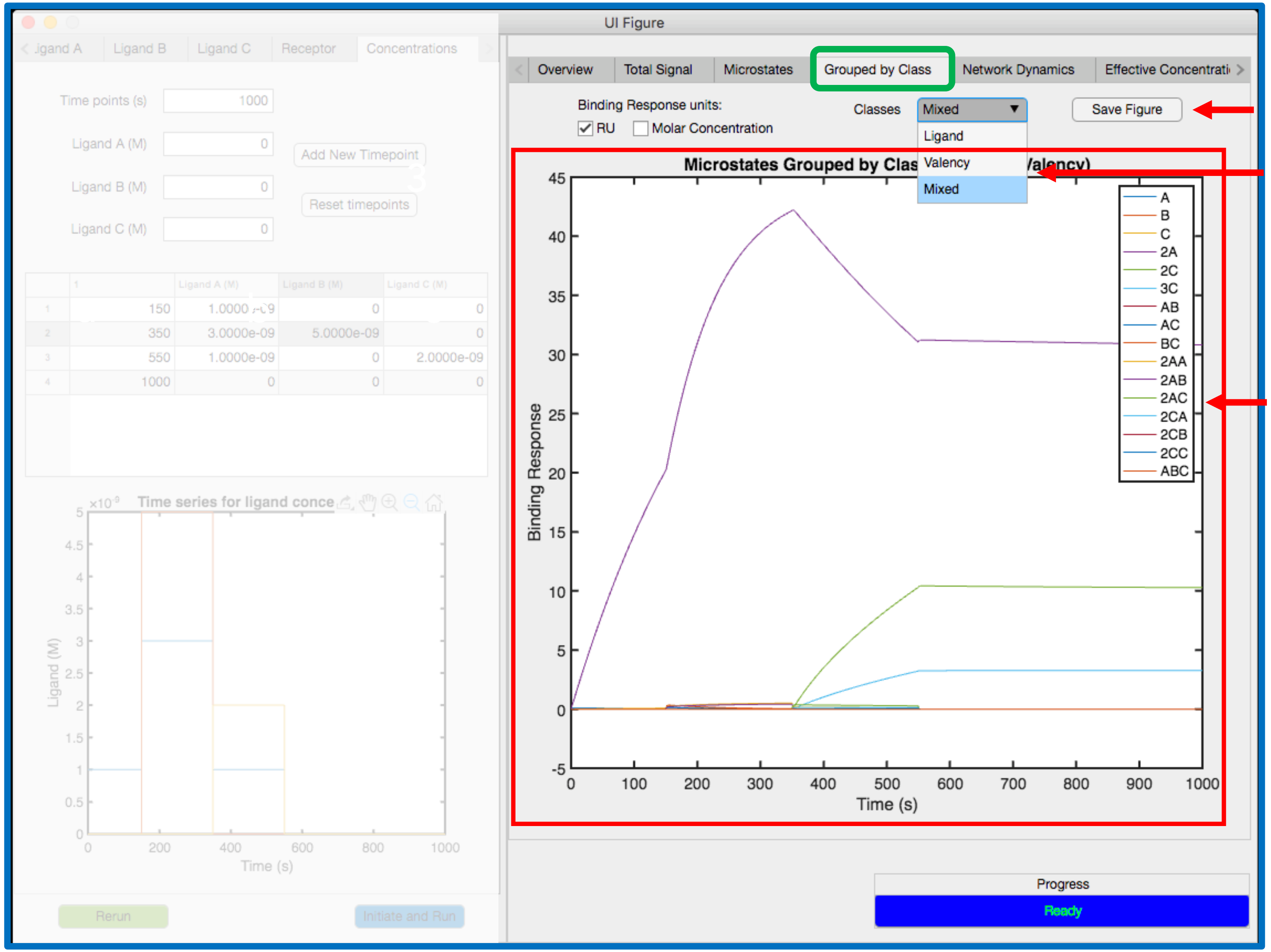

"Save Figure" exports the plot as a .fig file

three "Group by Class" options:

1. group by Ligand
2. group by Valency
3. group by Mixed (i.e., Ligand and Valency)

Plot Legend (for Group by "Mixed")

Here, the letters represent the Ligands composing the grouped microstates, and the numbers represent the valency

E.g., "C" = all microstates composed of one monovalently bound Ligand C

"3C" = all microstates composed of one trivalently bound Ligand C

"2CA" = all microstates composed of one bivalently bound Ligand C and one monovalently bound Ligand A

e. Output Network Dynamics

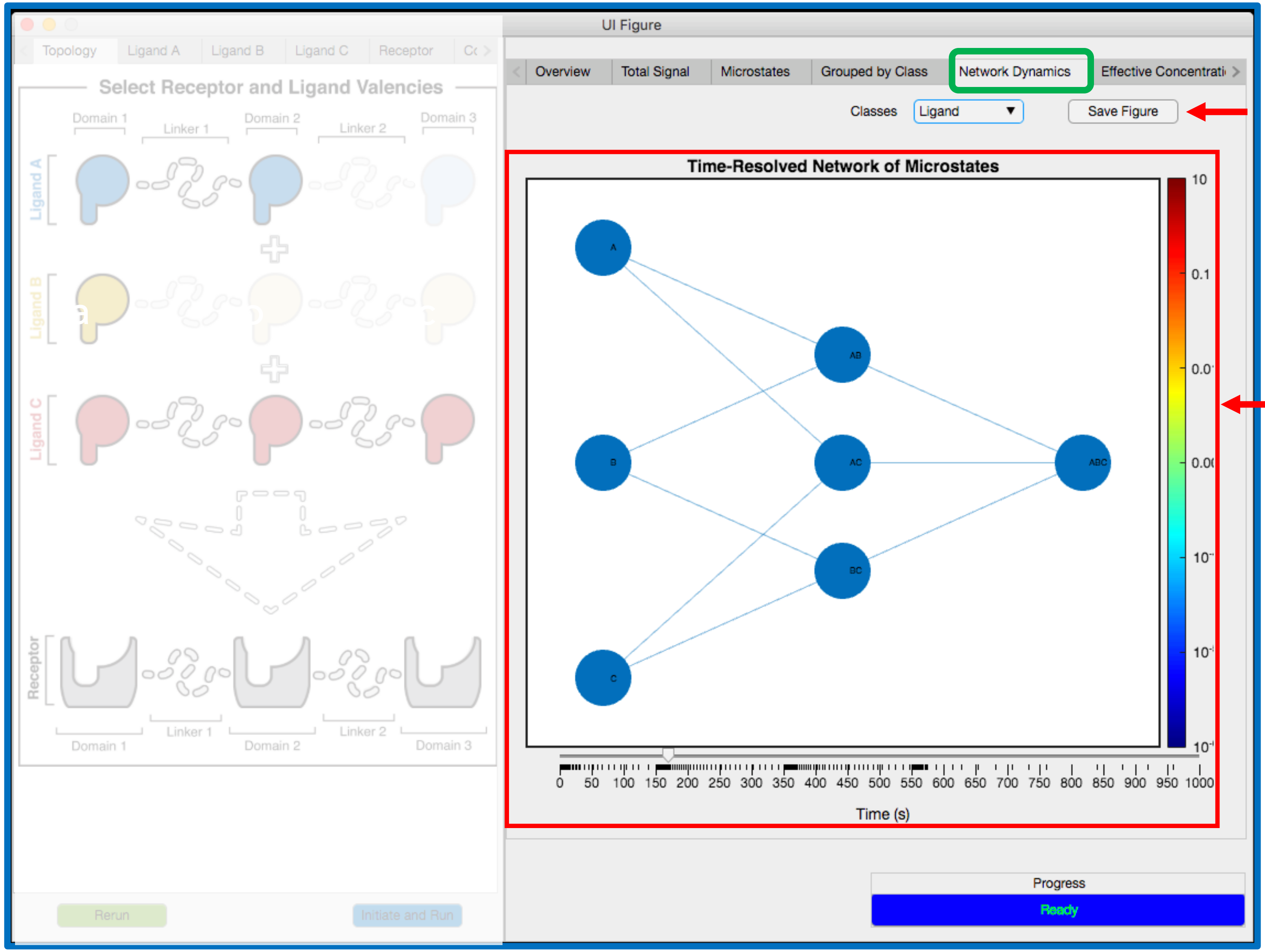

"Save Figure" exports the plot as a .fig file

the "Network Dynamics" tab displays a single view of the animated, "click-and-drag" representation of the microstate binding response dynamics presented in the Overview tab.

This visualization can be rendered according to two grouped classes of microstates: Ligand and Valency

f. Output Effective Concentration

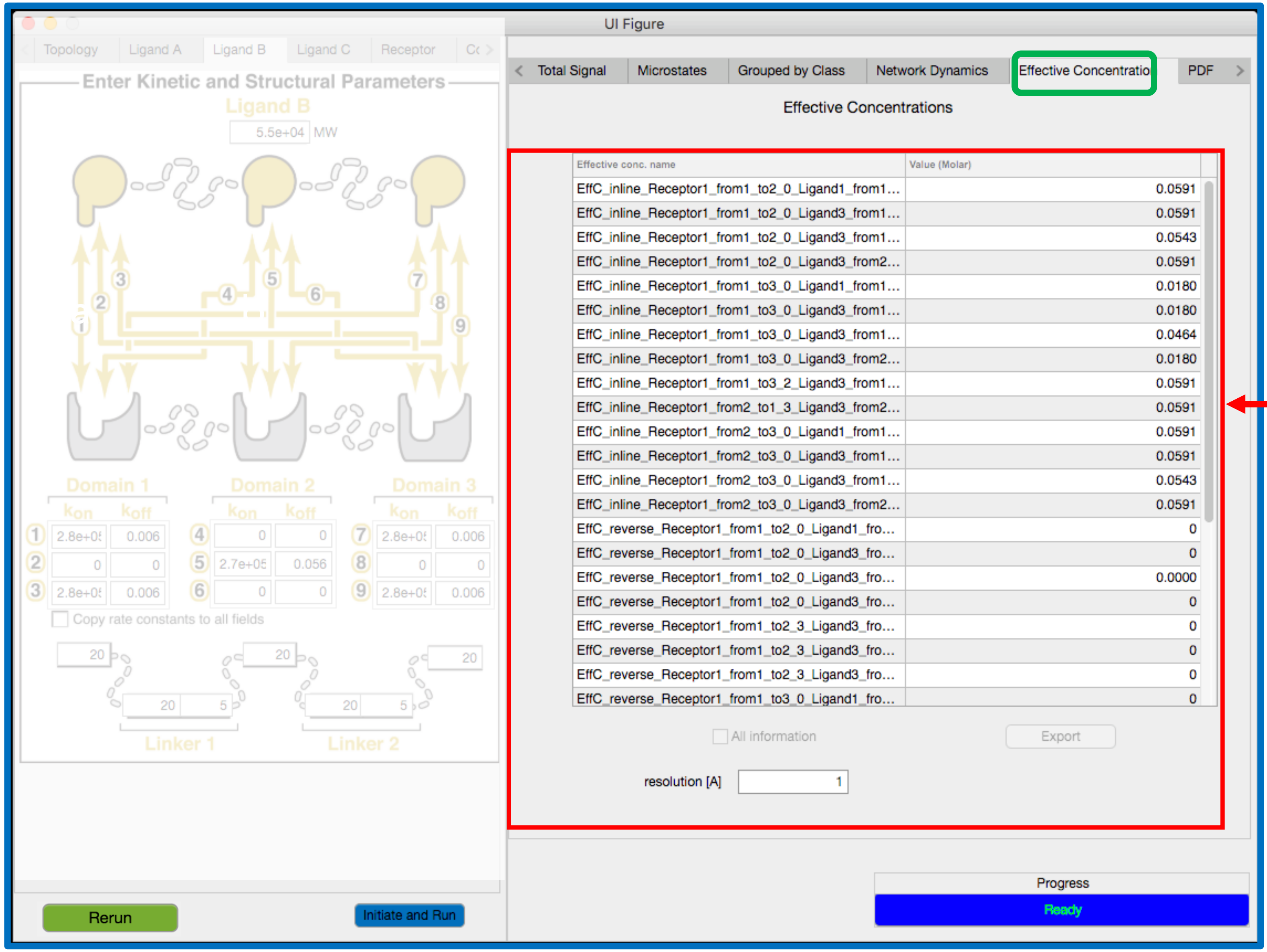

The Effective Concentration tab outputs the list of first-order rate constants of association that correlate with the permissibility of every possible "intra-complex" interaction (i.e., point of multivalent contact) that exists between the simulated ligands and receptor.

The left column lists each type of interaction.

The right column lists the effective concentration in molar units. These values can be manually edited: double click field, re-enter value, press "enter" on keyboard.

The simulation can be re-run with these user-specified values by clicking the "ReRun" button (bottom left). Note: the "ReRun" is highlighted in green when it can be used following an "Initiate and Run"

4. Navigating the *MVsim* output tabs

g. Output PDF (Probability Density Functions)

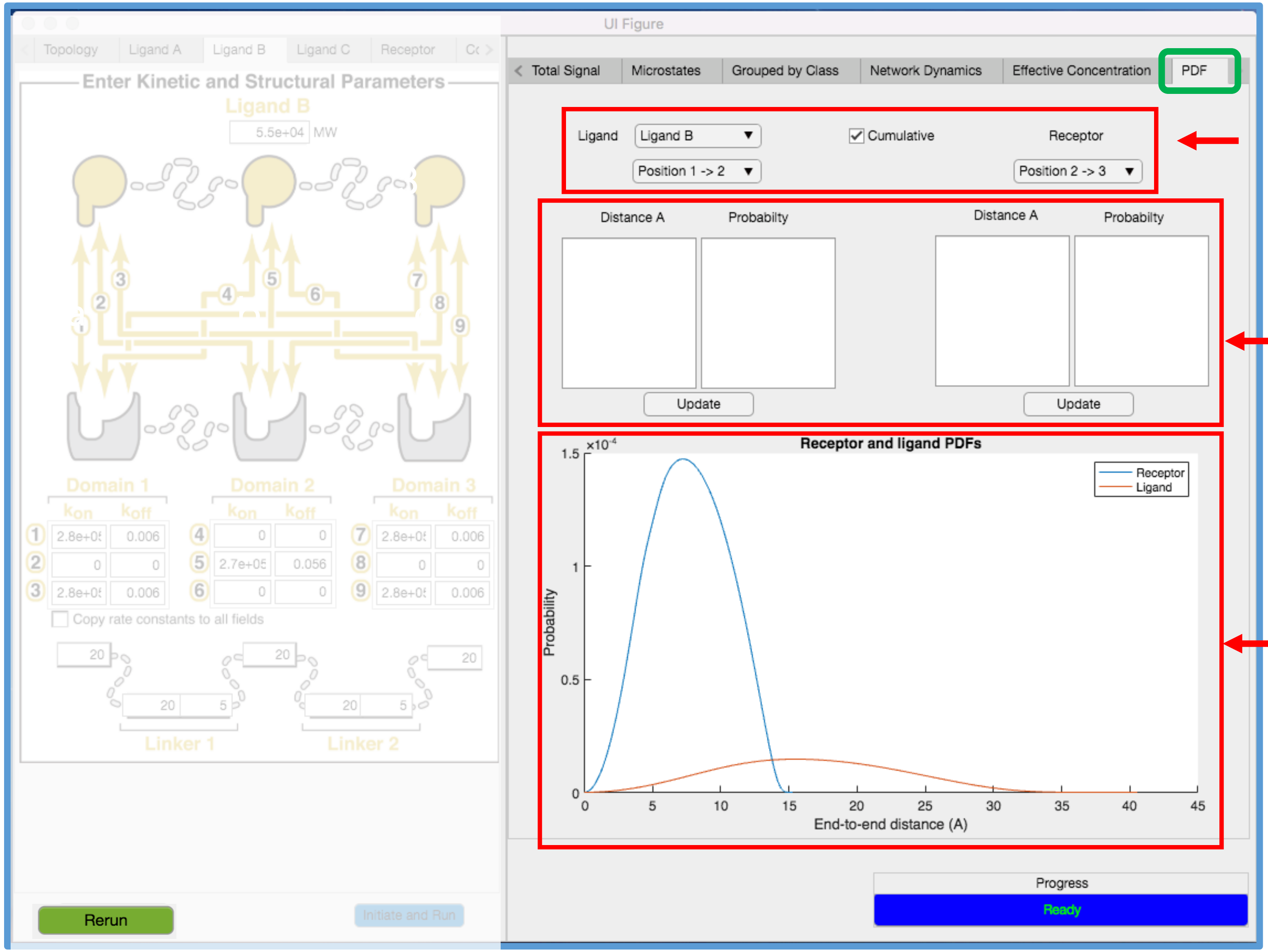

user selection of the PDF describing the probability of finding a domain *x* Å from an origin point. E.g., here, the PDF for Ligand B's domain 2 relative to domain 1; and the Receptor's domain 3 relative to domain 2 are selected from the drop down menus

input fields enabling the user to create their own PDF for the ligand and receptor domains selected above.

The "ReRun" button will re-run the simulation with the user-designed PDFs

graphical visualization of the two PDFs showing the overlap between the distributions of Ligand B domain 2 and Receptor domain 3

h. Batch Input/Output of a Ligand Concentration Series

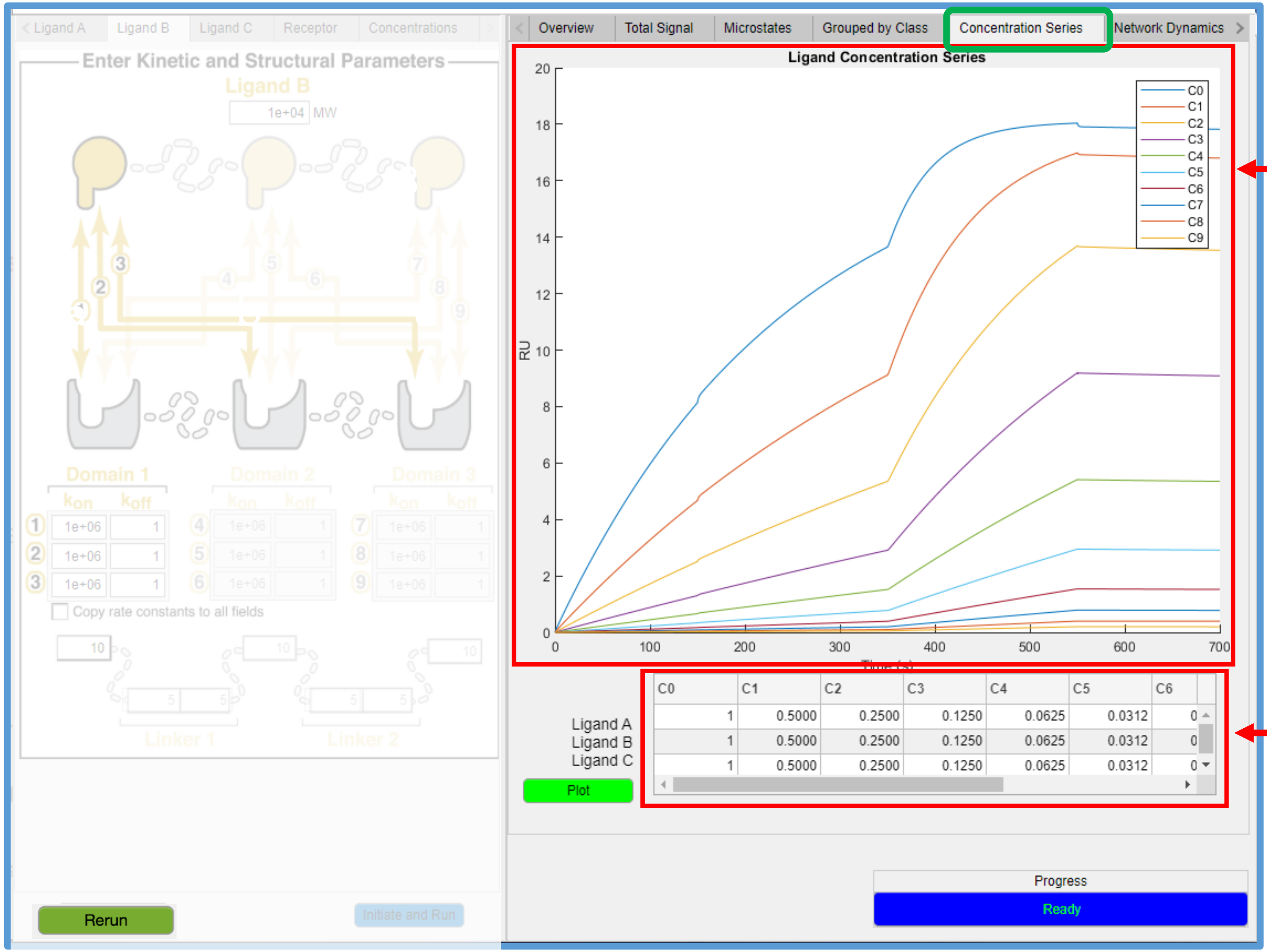

Plot showing the output for nine curves with varying ligand concentrations

The "Plot" button will output the curves for the given ligand concentrations.

The relative concentrations of the ligands compared to the original ligand concentration given in the concentration input tab.

Initially, a twofold dilution series is given for all the ligands. The concentration of the ligands can be kept at the original constant values by entering "1" for each cell in a row. Ligands can be excluded by entering "0" to any cells.

Any concentration can be specified, including relative values larger than 1.

Simulating Multivalent Biomolecular Interactions

Tutorial 2: Advanced Application of MVsim for the S protein

Bruncsics et al. (2022)

1. Parameterizing the competitive inhibition of the SARS-CoV-2 S protein - ACE2 interaction with a trivalent, RBD-binding inhibitor [example taken from Figure 5, Bruncsics et al., 2022]

a. Overview of the components of the multivalent interaction system in the Topology tab

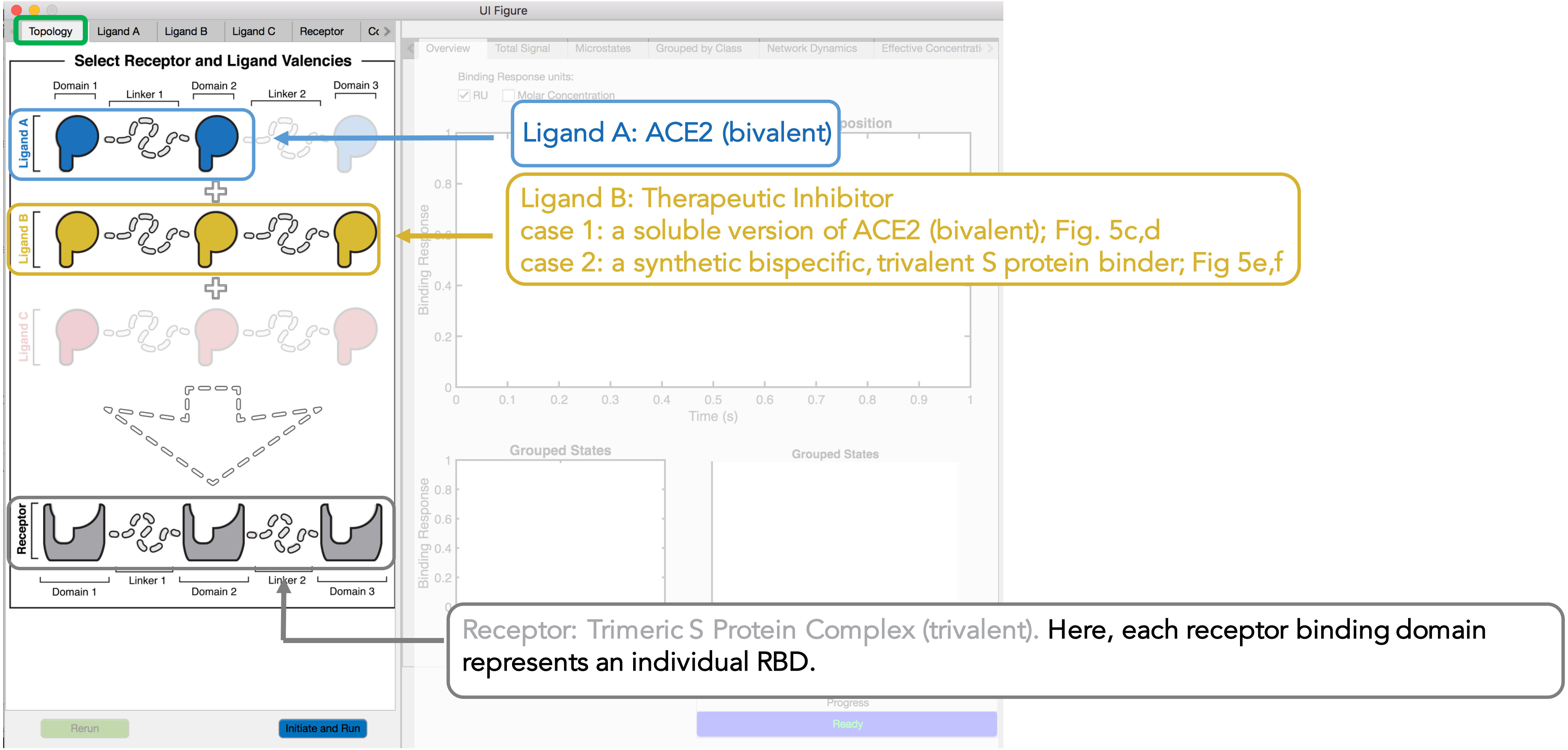

b. Parameterizing ACE2 from kinetic and structural data derived from the literature

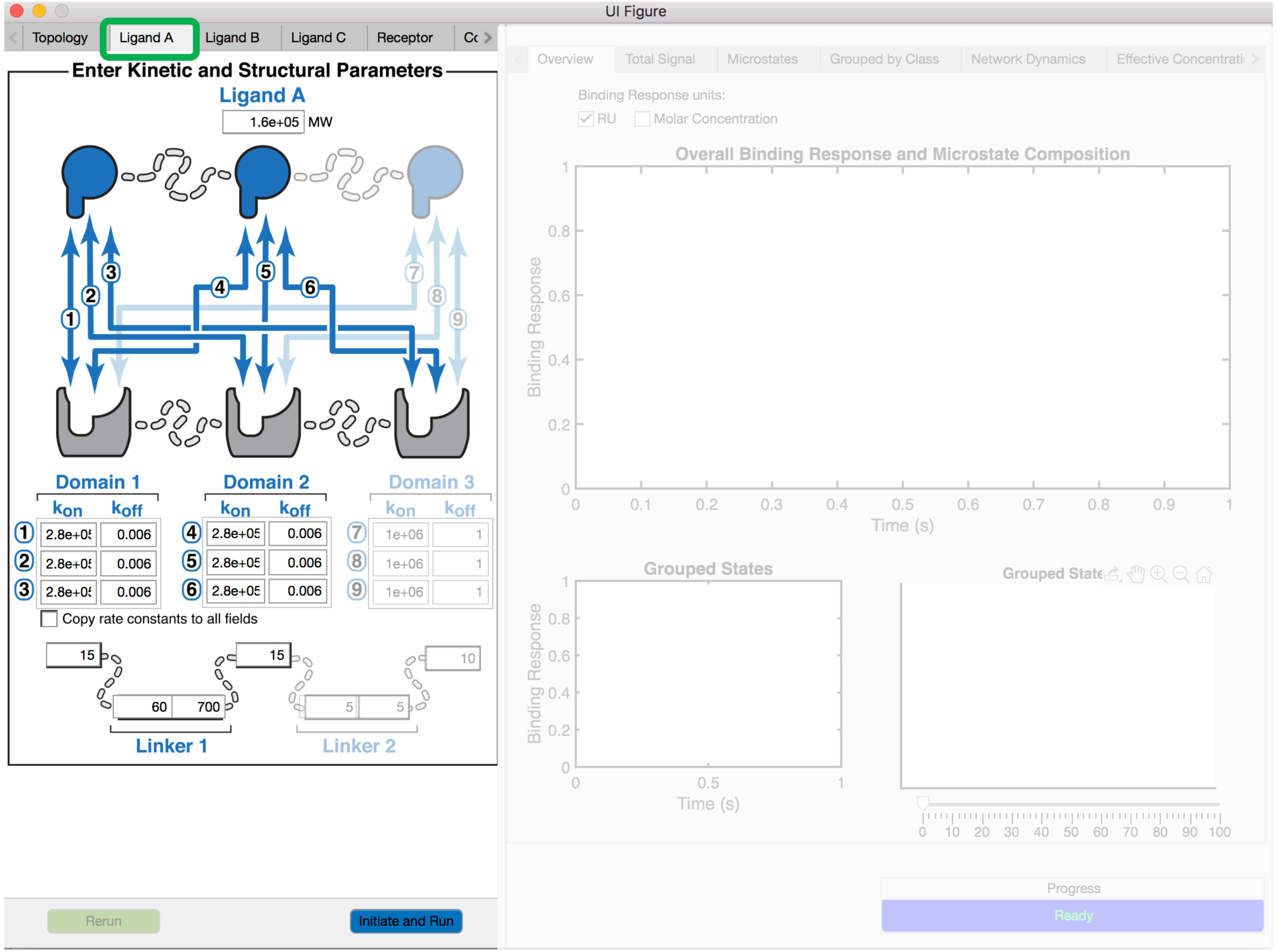

1. Parameterizing the competitive inhibition of the SARS-CoV-2 S protein - ACE2 interaction with a trivalent, RBD-binding inhibitor [example taken from Figure 5, Bruncsics et al., 2022]

c. Case 1: Parameterizing the bivalent, soluble inhibitory ACE2 parameterized from Chan et al. 2020

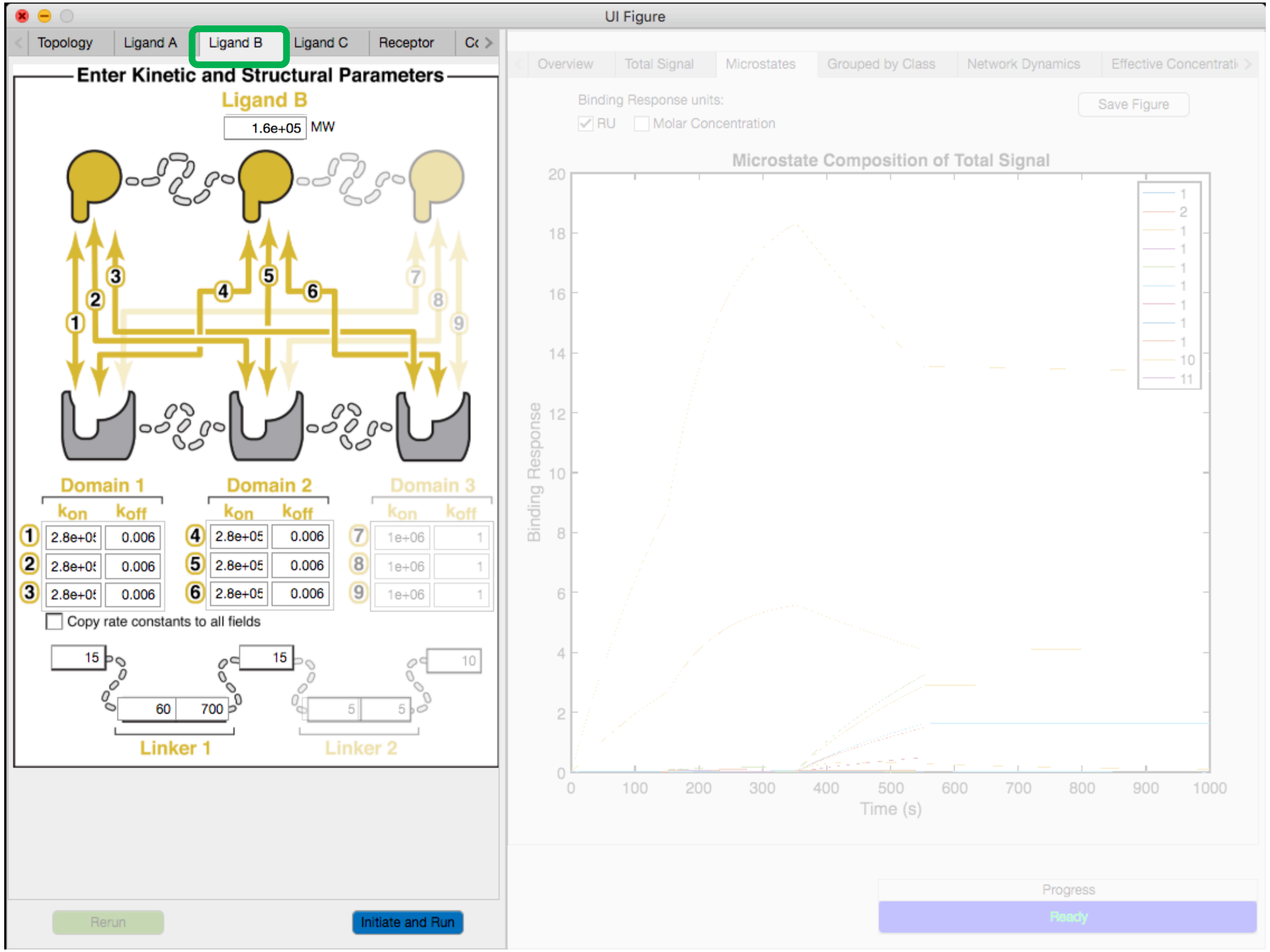

1. Parameterizing the competitive inhibition of the SARS-CoV-2 S protein - ACE2 interaction with a trivalent, RBD-binding inhibitor [example taken from Figure 5, Bruncsics et al., 2022]

d. Case 2: Parameterizing the computationally derived bispecific, trivalent S protein inhibitor

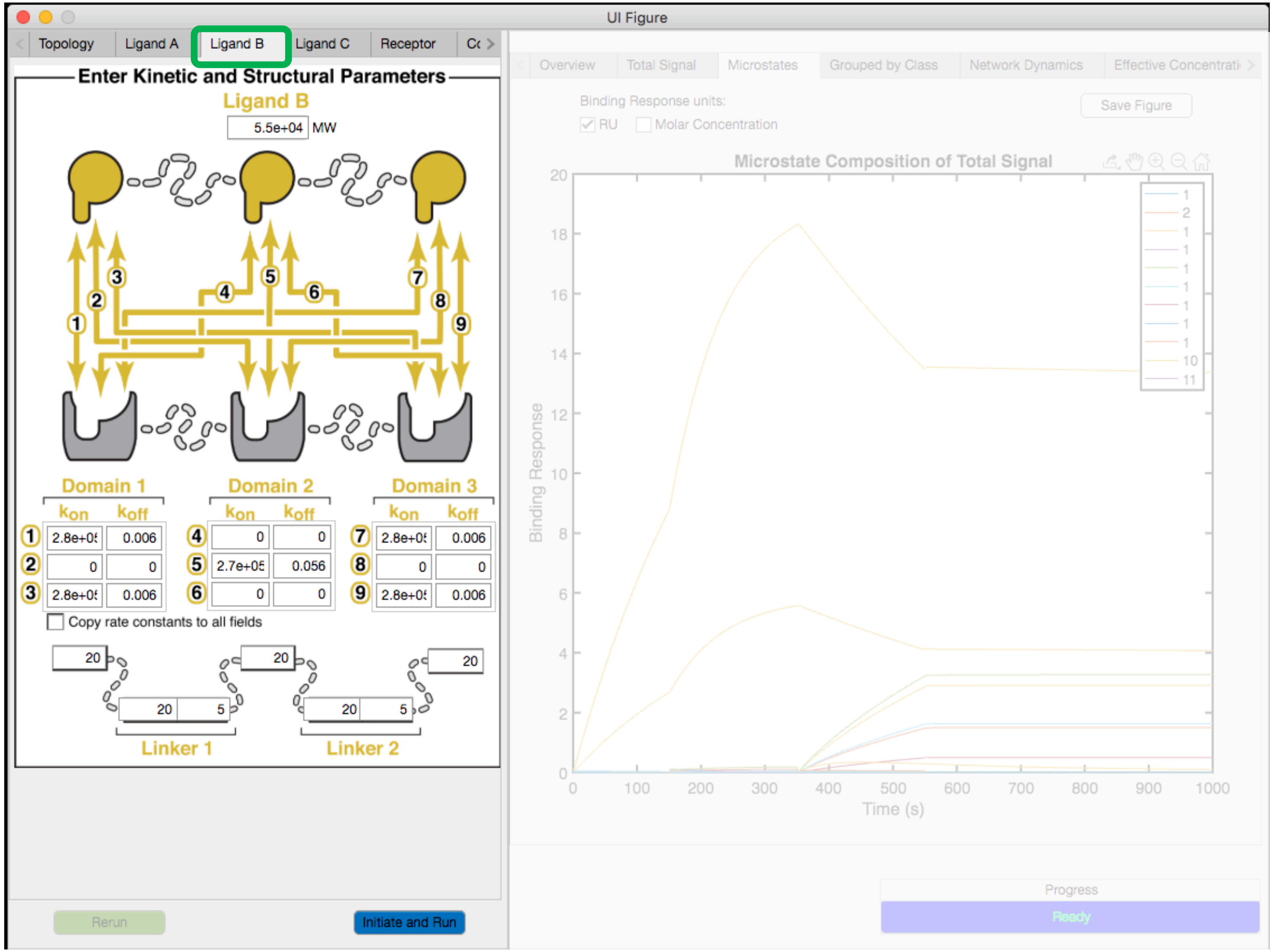

1. Parameterizing the competitive inhibition of the SARS-CoV-2 S protein - ACE2 interaction with a trivalent, RBD-binding inhibitor [example taken from Figure 5, Bruncsics et al., 2022]

e. Parameterizing the trimeric and trivalent S protein from a suite of structural data from the literature

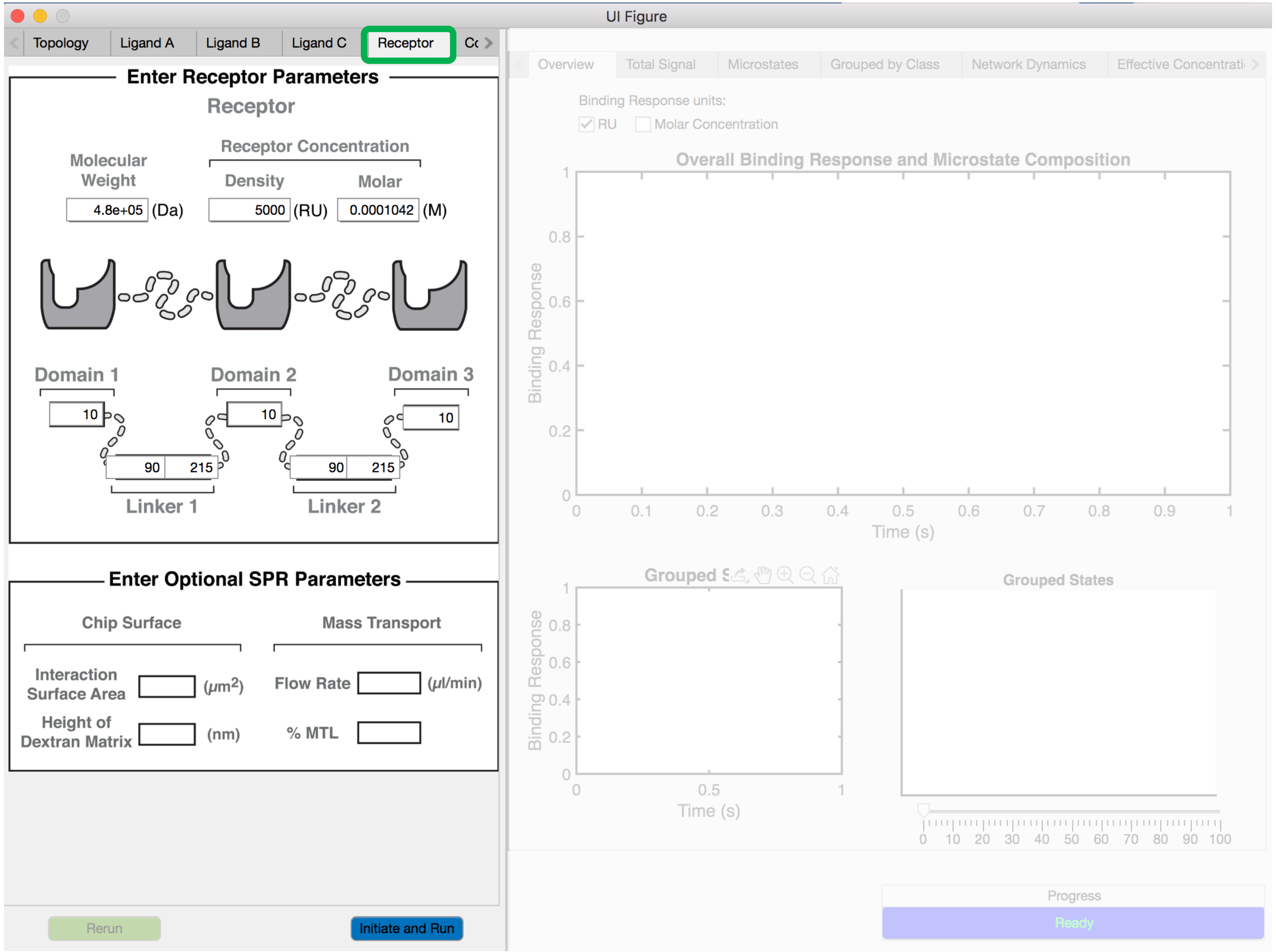

2. Parameterizing the competitive inhibition of the SARS-CoV-2 S protein - S protein interaction with a trivalent, RBD-down-specific inhibitor [example taken from Figure 6, Bruncsics et al., 2022]

a. Overview of the components of the multivalent interaction system in the Topology tab

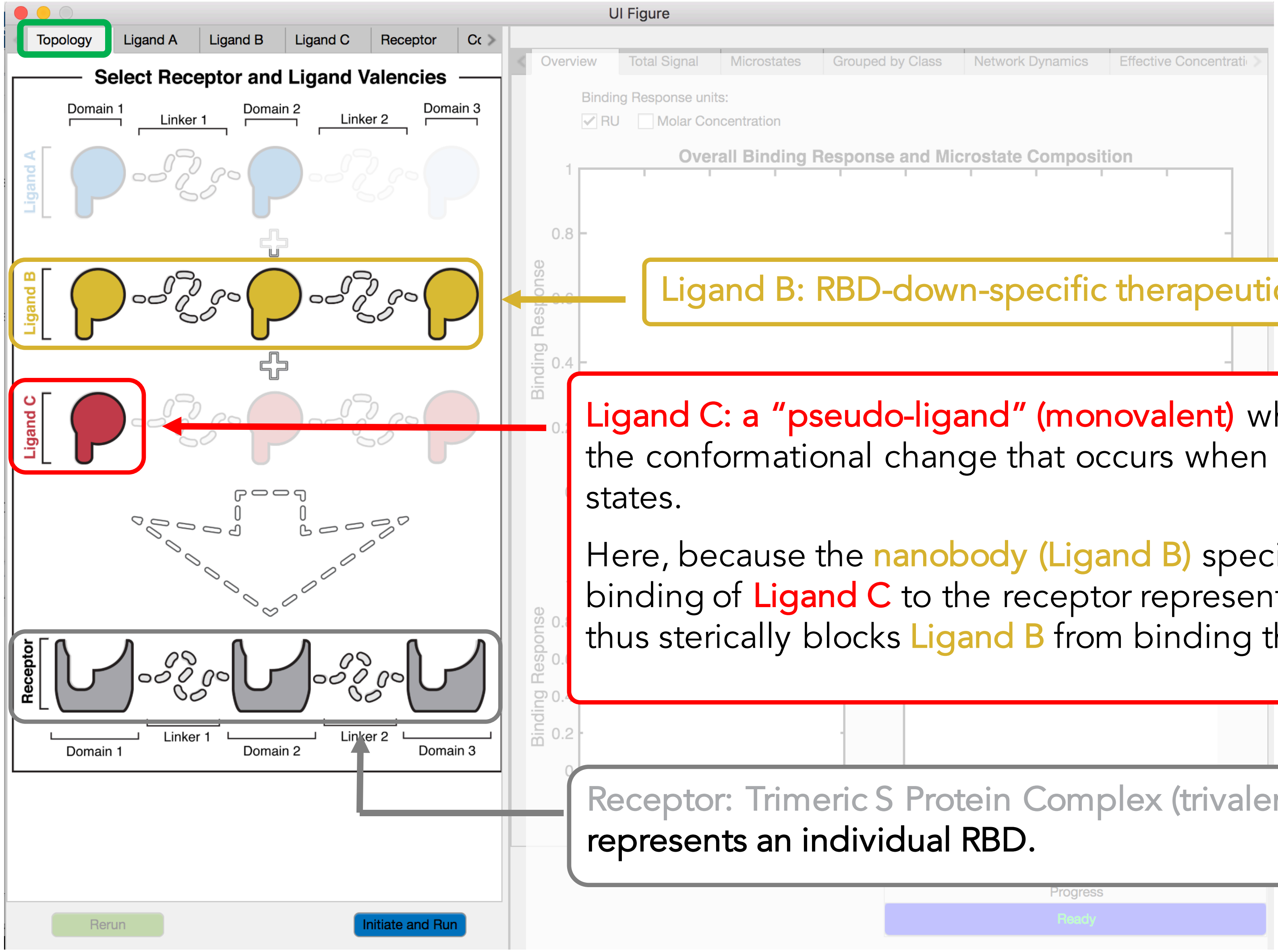

Ligand B: RBD-down-specific therapeutic inhibitor

Ligand C: a "pseudo-ligand" (monovalent) whose binding to the S protein receptor mimics the conformational change that occurs when an RBD "flips" between its "down" and "up" states.

Here, because the nanobody (Ligand B) specifically binds the RBD-down conformation, the binding of Ligand C to the receptor represents an instance of an RBD-up conformation, and thus sterically blocks Ligand B from binding that Receptor **binding domain.**

Receptor: Trimeric S Protein Complex (trivalent). **Here, each receptor binding domain represents an individual RBD.**

2. Parameterizing the competitive inhibition of the SARS-CoV-2 S protein - ACE2 interaction with a trivalent, RBD-binding inhibitor [example taken from Figure 6, Bruncsics et al., 2022]

b. Parameterizing the trivalent nanobody from kinetic and structural data derived from its primary report

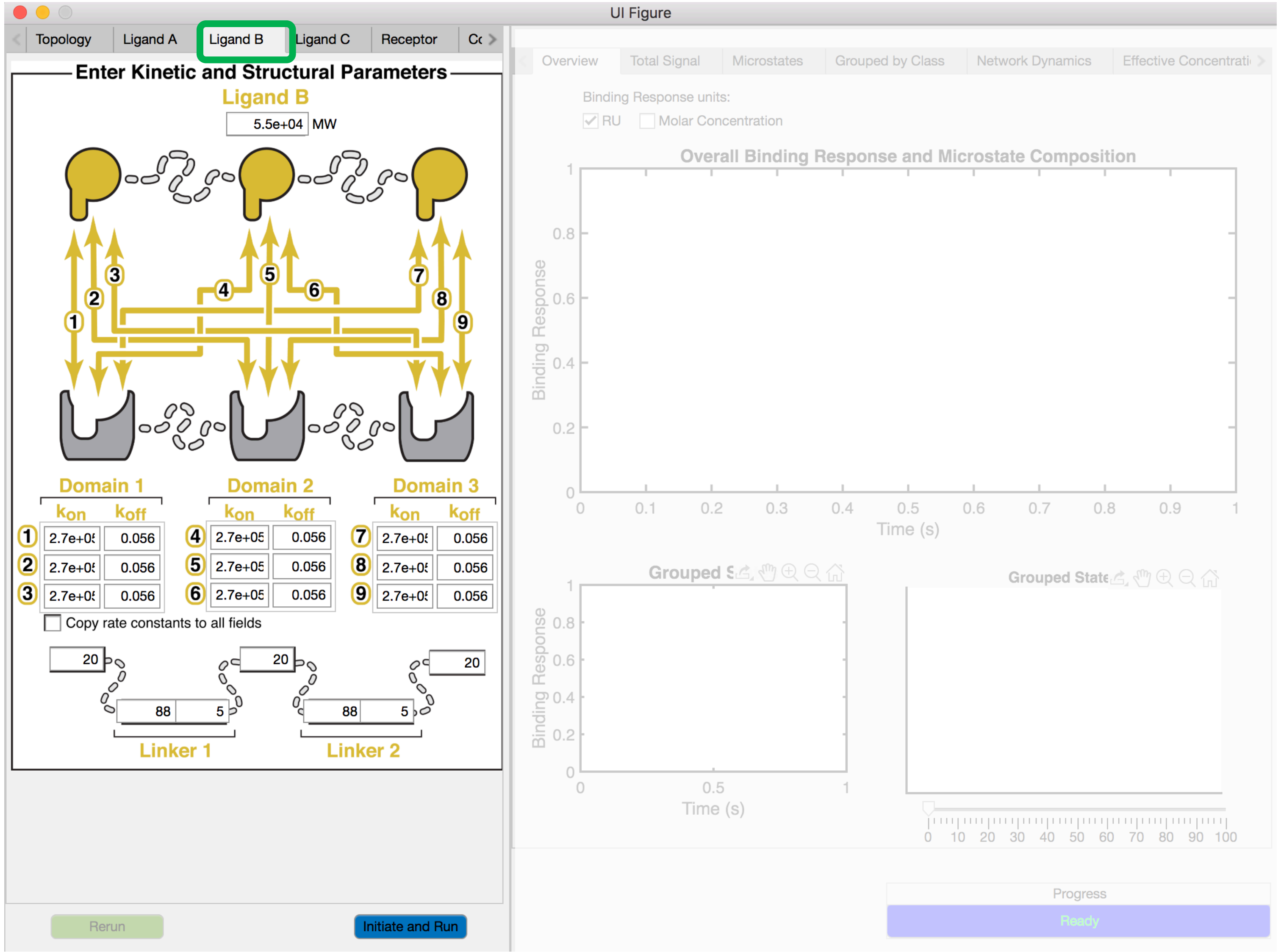

2. Parameterizing the competitive inhibition of the SARS-CoV-2 S protein - ACE2 interaction with a trivalent, RBD-binding inhibitor [example taken from Figure 6, Bruncsics et al., 2022]

c. Parameterizing the S protein RBD "up"/ "down" conformational change

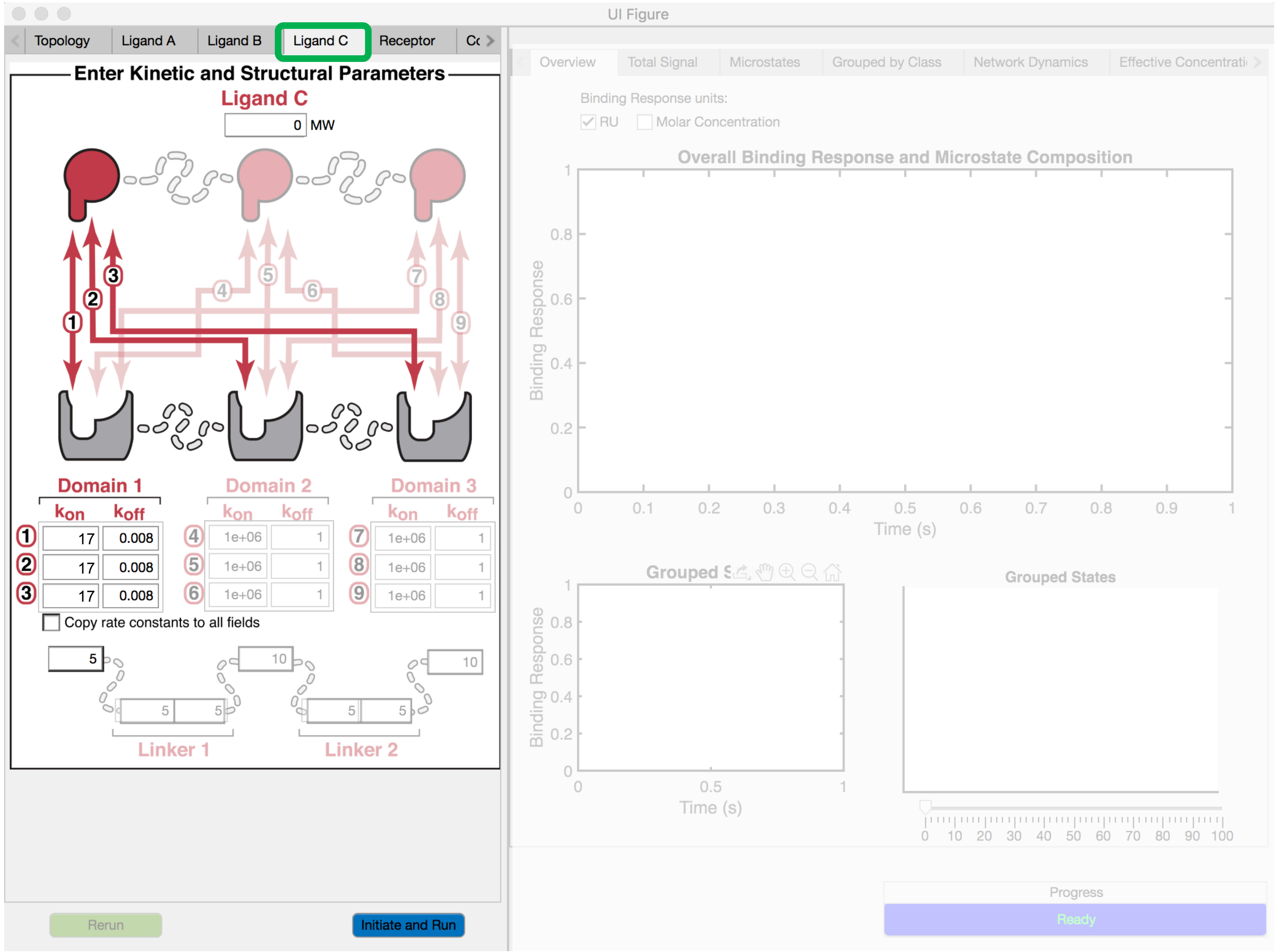

2. Parameterizing the competitive inhibition of the SARS-CoV-2 S protein - ACE2 interaction with a trivalent, RBD-binding inhibitor [example taken from Figure 6, Bruncsics et al., 2022]

d. Parameterizing the ligand concentrations

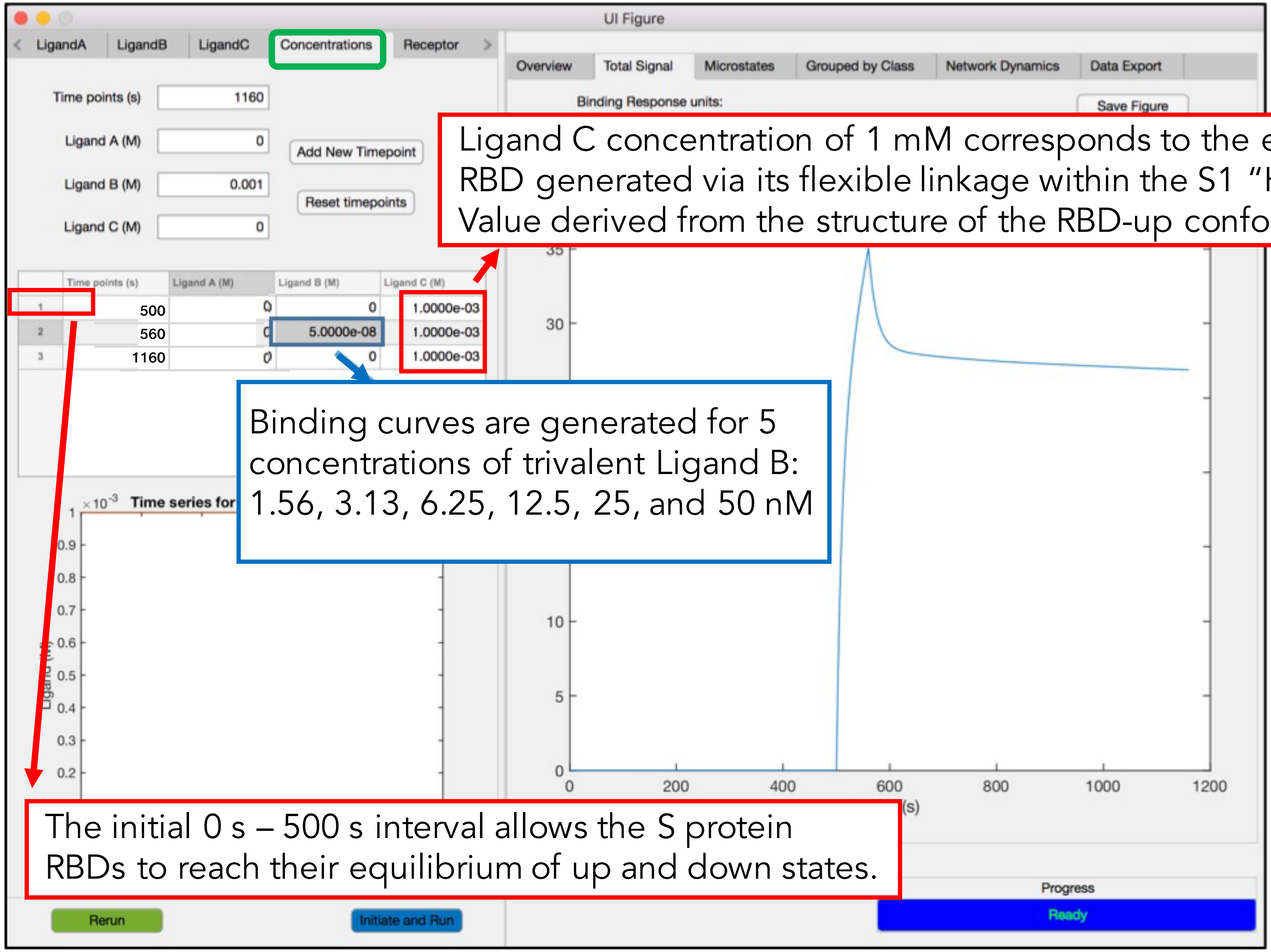

Time points (s)	Ligand A (M)	Ligand B (M)	Ligand C (M)
500	0	0	1.0000e-03
560	0	5.0000e-08	1.0000e-03
1160	0	0	1.0000e-03

2. Parameterizing the competitive inhibition of the SARS-CoV-2 S protein - ACE2 interaction with a trivalent, RBD-binding inhibitor [example taken from Figure 6, Bruncsics et al., 2022]

e. Parameterizing the trimeric and trivalent S protein from a suite of structural data from the literature

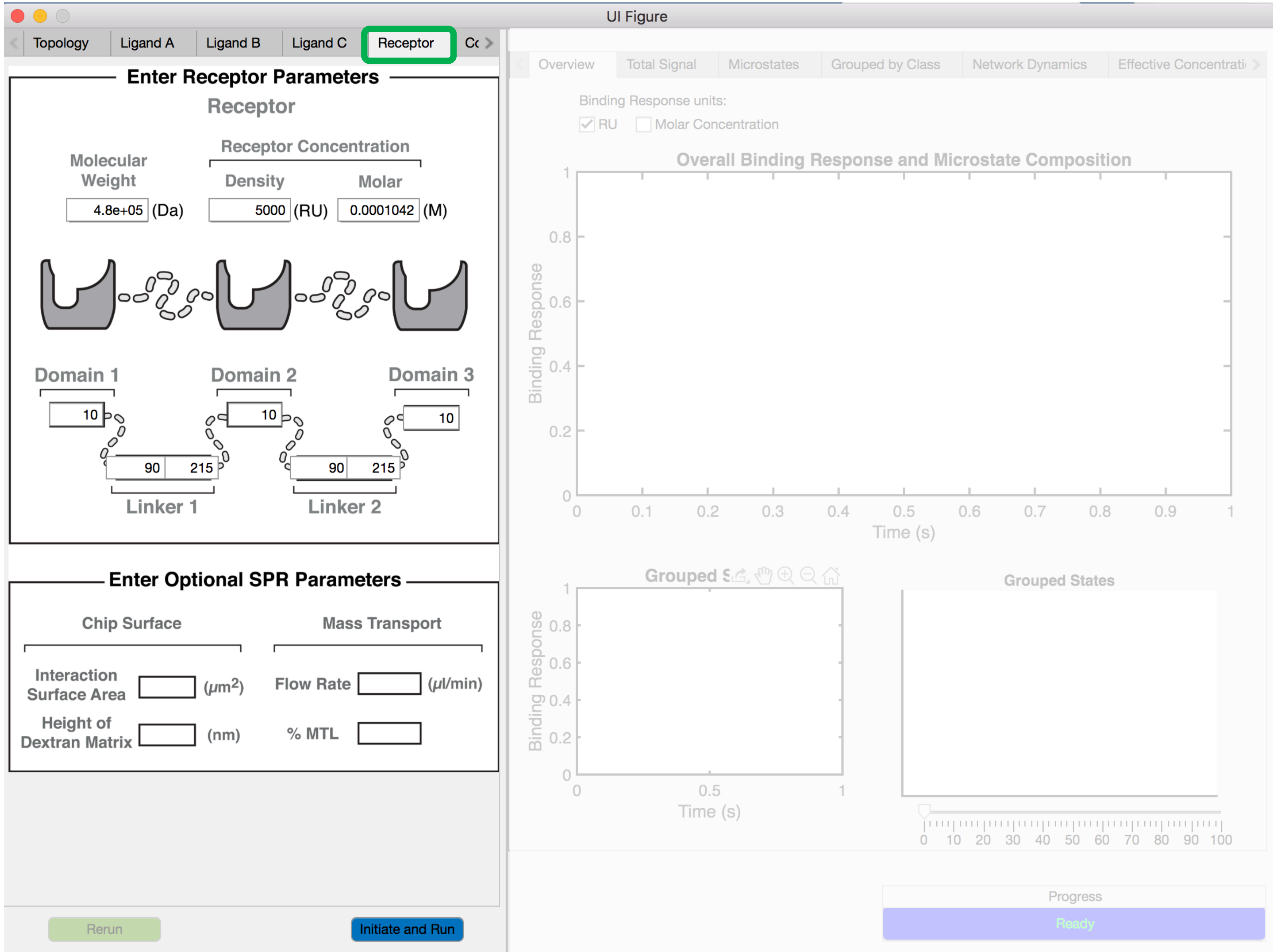

5. Supplementary References

[1] Liu, Q. et al. Structural basis for specific binding of the Gads SH3 domain to an RxxK motif-containing SLP-76 peptide: a novel mode of peptide recognition. *Mol. Cell* **11,** 471-481 (2003).

[2] Karanicolas, J. et al. A de novo protein binding pair by computational design and directed evolution. *Mol. Cell* **42,** 250-260 (2011).

[3] Myszka, D.G. et al. Extending the range of rate constants available from BIACORE: interpreting mass transport-influenced binding data. *Biophys. J.* **75,** 583-594 (1998).

[4] Rich, R.L. & Myszka, D.G. Survey of the year 2005 commercial optical biosensor literature. *J. Mol. Recognit.* **19,** 478-534 (2006).

[5] Hangos, K.M., Bokor, J. & Szederkényi, G. *Analysis and Control of Nonlinear Process Systems.* Grimble, M.J., Johnson, M.A. eds. (Springer, 2004).

[6] Zinth, U.G. End-to-end distance distribution and intra-chain diffusion in unfolded polypeptide chains. Doctoral dissertation, Technischen Universität München (11.11.2013).

[7] Thirumalai, D. & Ha, B.Y. Statistical mechanics of semiflexible chains: A meanfield variational approach. *Theoretical and Mathematical Models in Polymer Research*. Grosberg, A. ed. (Academic Press, 1997).

[8] Choe, S. & Sun, S.X. The elasticity of alpha-helices. *J. Chem. Phys.* **122,** 244912 (2005).

[9] Palenčár, P. & Bleha, T. Buckling transition in long α-helices. *J. Chem. Phys.* **141,** 174901 (2014).

[10] Li, G. et al. Construction of a linker library with widely controllable flexibility for fusion protein design. *Appl. Microbiol. Biotechnol.* **100,** 215-225 (2016).

[11] van Rosmalen, M., Krom, M. & Merkx, M. Tuning the flexibility of glycine-serine linkers to allow rational design of multidomain proteins. *Biochemistry* **56,** 6565-6574 (2017).

[12] Aitio, O., Hellman, M., Skehan, B., Kesti, T., Leong, J.M., Saksela, K. & Permi, P. Enterohaemorrhagic Escherichia coli exploits a tryptophan switch to hijack host F-actin assembly. *Structure* **20**, 1692-1703 (2012).

[13] Abdul-Manan, N., Aghazadeh, B., Liu, G.A., Majumdar, A., Ouerfelli, O. & Rosen, M.K. Solution structure of Cdc42 in complex with the GTPase binding domain of WASP. *Nature* **399**, 379-383 (1999).

[14] Hillier, B.J., Christopherson, K.S., Prehoda, K.E., Bredt, D.S. & Lim, W.A. Unexpected modes of PDZ domain scaffolding revealed by structure of nNOS-syntrophin complex. *Science* **284**, 812-815 (1999).

[15] Schultz, J., Hoffmueller, U., Ashurst, J., Krause, G., Schmieder, P., Macias, M., Schneider-Mergener & J., Oschkinat, H. Solution structure of the syntrophin PDZ domain in complex with the peptide GVKESLV. *Nat. Struct. Biol.* **5**, 19-24 (1998).

[16] Wu, X., Knudsen, B., Feller, S.M., Zheng, J., Sali, A., Cowburn, D., Hanafusa, H. & Kuriyan, J. Structural basis for the specific interaction of lysine-containing proline-rich peptides with the N-terminal SH3 domain of c-Crk. *Structure* **3**, 215-226 (1995).

[17] Yuan, Y., Cao, D., Zhang, Y., Ma, J., Qi, J., Wang, Q., Lu, G., Wu, Y., Yan, J., Shi, Y., Zhang, X. & Gao, G.F. Cryo-EM structures of MERS-CoV and SARS-CoV spike glycoproteins reveal the dynamic receptor binding domains. *Nat. Commun.* **8**, 15092-15092 (2017).

[18] Kirchdoerfer, R.N., Wang, N., Pallesen, J., Turner, H.L., Cottrell, C.A., McLellan, J.S. & Ward, A.B. SARS Spike Glycoprotein, Stabilized variant, single upwards S1 CTD conformation. *Sci. Rep.* **8**, 15701-15701 (2018).

[19] Walls, A.C., Xiong, X., Park, Y.J., Tortorici, M.A., Snijder, J., Quispe, J., Cameroni, E., Gopal, R., Dai, M., Lanzavecchia, A., Zambon, M., Rey, F.A., Corti, D. & Veesler, D. Unexpected Receptor Functional Mimicry Elucidates Activation of Coronavirus Fusion. *Cell* **176**, 1026-1039.e15 (2019).

[20] Wrapp, D., Wang, N., Corbett, K.S., Goldsmith, J.A., Hsieh, C.L., Abiona, O., Graham, B.S. & McLellan, J.S. Cryo-EM structure of the 2019-nCoV spike in the prefusion conformation. *Science* **367**, 1260-1263 (2020).

[21] Walls, A.C., Park, Y.J., Tortorici, M.A., Wall, A., McGuire, A.T. & Veesler, D. Structure, Function, and Antigenicity of the SARS-CoV-2 Spike Glycoprotein. *Cell* **181**, 281 (2020).

[22] Yan, R., Zhang, Y., Li, Y., Xia, L., Guo, Y. & Zhou, Q. Structural basis for the recognition of SARS-CoV-2 by full-length human ACE2. *Science* **367**, 1444-1448 (2020).

[23] Shang, J., Ye, G., Shi, K., Wan, Y., Luo, C., Aihara, H., Geng, Q., Auerbach, A. & Li, F. Structural basis of receptor recognition by SARS-CoV-2. *Nature* **581**, 221-224 (2020).
