# Supplementary material for: MVsim is a toolset for quantifying and designing multivalent interactions
Source: Nat Commun. 2022 Sep 6;13:5029. doi: 10.1038/s41467-022-32496-6 (PMC9448752; doi:10.1038/s41467-022-32496-6)
Supplement: Supplementary file 2 — Reporting Summary [file 41467_2022_32496_MOESM2_ESM.pdf]

nature portfolio

Corresponding author(s): Casim A. Sarkar

Last updated by author(s): Jul 29, 2022

Reporting Summary

Nature Portfolio wishes to improve the reproducibility of the work that we publish. This form provides structure for consistency and transparency in reporting. For further information on Nature Portfolio policies, see our Editorial Policies and the Editorial Policy Checklist.

Statistics

For all statistical analyses, confirm that the following items are present in the figure legend, table legend, main text, or Methods section.

n/a	Confirmed	
☒	☐	The exact sample size (*n*) for each experimental group/condition, given as a discrete number and unit of measurement
☒	☐	A statement on whether measurements were taken from distinct samples or whether the same sample was measured repeatedly
☒	☐	The statistical test(s) used AND whether they are one- or two-sided<br>*Only common tests should be described solely by name; describe more complex techniques in the Methods section.*
☒	☐	A description of all covariates tested
☒	☐	A description of any assumptions or corrections, such as tests of normality and adjustment for multiple comparisons
☐	☒	A full description of the statistical parameters including central tendency (e.g. means) or other basic estimates (e.g. regression coefficient) AND variation (e.g. standard deviation) or associated estimates of uncertainty (e.g. confidence intervals)
☒	☐	For null hypothesis testing, the test statistic (e.g. *F*, *t*, *r*) with confidence intervals, effect sizes, degrees of freedom and *P* value noted<br>*Give P values as exact values whenever suitable.*
☒	☐	For Bayesian analysis, information on the choice of priors and Markov chain Monte Carlo settings
☒	☐	For hierarchical and complex designs, identification of the appropriate level for tests and full reporting of outcomes
☒	☐	Estimates of effect sizes (e.g. Cohen's *d*, Pearson's *r*), indicating how they were calculated

Our web collection on statistics for biologists contains articles on many of the points above.

Software and code

Policy information about availability of computer code

Data collection	The simulated datasets that were generated for this study were produced using the custom MVsim software (v0.9) developed in MATLAB 2019b (v9.7). The MVsim application and source code are available on GitHub (https://github.com/sarkarlab/MVsim) under the MIT License.
Data analysis	All analyses of experimental data in this study were performed using the MVsim software. Where indicated, further analyses (i.e., curve-fitting, data plotting, and figure formatting) were performed outside of MVsim using standard, commercially-available MATLAB toolsets (Curve Fitting and Plot Editor Toolsets; MATLAB 2019b v9.7) and the Cytoscape software (v3.7.1).

For manuscripts utilizing custom algorithms or software that are central to the research but not yet described in published literature, software must be made available to editors and reviewers. We strongly encourage code deposition in a community repository (e.g. GitHub). See the Nature Portfolio guidelines for submitting code & software for further information.

Data

Policy information about availability of data

All manuscripts must include a data availability statement. This statement should provide the following information, where applicable:

- Accession codes, unique identifiers, or web links for publicly available datasets
- A description of any restrictions on data availability
- For clinical datasets or third party data, please ensure that the statement adheres to our policy

All data generated, analyzed, and used in this study are included in this article and its supplementary information files. The raw data underlying the figures are provided as a source data file with this paper. Where indicated in the paper, parameterization of the MVsim simulations were performed with data obtained from the Protein Data Bank (www.rcsb.org) using the following PDB accession codes: 3Q9N, 1H3H, 2LNH, 1CEE, 1QAV, 2PDZ, 1CKA, 5X5B, 6CRW, 6NB6, 6NB7, 6VSB, 6VYB, 6M17, and 6VW1.

Field-specific reporting

Please select the one below that is the best fit for your research. If you are not sure, read the appropriate sections before making your selection.

[x] Life sciences [ ] Behavioural & social sciences [ ] Ecological, evolutionary & environmental sciences

For a reference copy of the document with all sections, see nature.com/documents/nr-reporting-summary-flat.pdf

Life sciences study design

All studies must disclose on these points even when the disclosure is negative.

Sample size	No samples were taken in this study.
Data exclusions	Experimental surface plasmon resonance (SPR) datasets were collected for a suite of engineered receptor and ligand proteins, whose quality was assessed by size-exclusion chromatography and SPR. Attempts to express specific trivalent proteins with short, rigid alpha-helical linkers were not successful, as the proteins were unstable in solution (i.e., exhibited a tendency to degrade, aggregate, and/or misfold). For this reason, these proteins were not able to be further studied (i.e., no SPR experiments were performed with them).
Replication	Experimental SPR duplicates were performed for select ligand concentrations to ensure reproducibility and stability of the SPR instrumentation. All attempts at replication were successful. Simulated SPR datasets were not performed in replicate, as the MVsim software is deterministic.
Randomization	Randomization was not used, as the datasets generated and analyzed by MVsim software are deterministic.
Blinding	No blinding was performed, as statistical and quantitative analyses (where used) were performed automatically by the computational MVsim software and MATLAB toolsets.

Reporting for specific materials, systems and methods

We require information from authors about some types of materials, experimental systems and methods used in many studies. Here, indicate whether each material, system or method listed is relevant to your study. If you are not sure if a list item applies to your research, read the appropriate section before selecting a response.

Materials & experimental systems

n/a	Involved in the study	
[x]	[ ]	Antibodies
[x]	[ ]	Eukaryotic cell lines
[x]	[ ]	Palaeontology and archaeology
[x]	[ ]	Animals and other organisms
[x]	[ ]	Human research participants
[x]	[ ]	Clinical data
[x]	[ ]	Dual use research of concern

Methods

n/a	Involved in the study	
[x]	[ ]	ChIP-seq
[x]	[ ]	Flow cytometry
[x]	[ ]	MRI-based neuroimaging
