# Supplementary material for: MVsim is a toolset for quantifying and designing multivalent interactions
Source: Nat Commun. 2022 Sep 6;13:5029. doi: 10.1038/s41467-022-32496-6 (PMC9448752; doi:10.1038/s41467-022-32496-6)
Supplement: Supplementary file 3 — Description of Additional Supplementary Files [file 41467_2022_32496_MOESM3_ESM.pdf]

Title: Supplementary Software

Description: MVsim.zip contains installation files for the *MVsim* MATLAB app. Other installation options are available on GitHub (https://sarkarlab.github.io/MVsim/).
