# Supplementary figures and images for: MVsim is a toolset for quantifying and designing multivalent interactions
Source: Nat Commun. 2022 Sep 6;13:5029. doi: 10.1038/s41467-022-32496-6 (PMC9448752; doi:10.1038/s41467-022-32496-6)

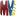

Supplement: Supplementary file 4 — Supplementary Software [file 41467_2022_32496_MOESM4_ESM.zip › MVsim_resources/icon_16.png]

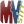

Supplement: Supplementary file 4 — Supplementary Software [file 41467_2022_32496_MOESM4_ESM.zip › MVsim_resources/icon_24.png]

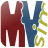

Supplement: Supplementary file 4 — Supplementary Software [file 41467_2022_32496_MOESM4_ESM.zip › MVsim_resources/icon_48.png]

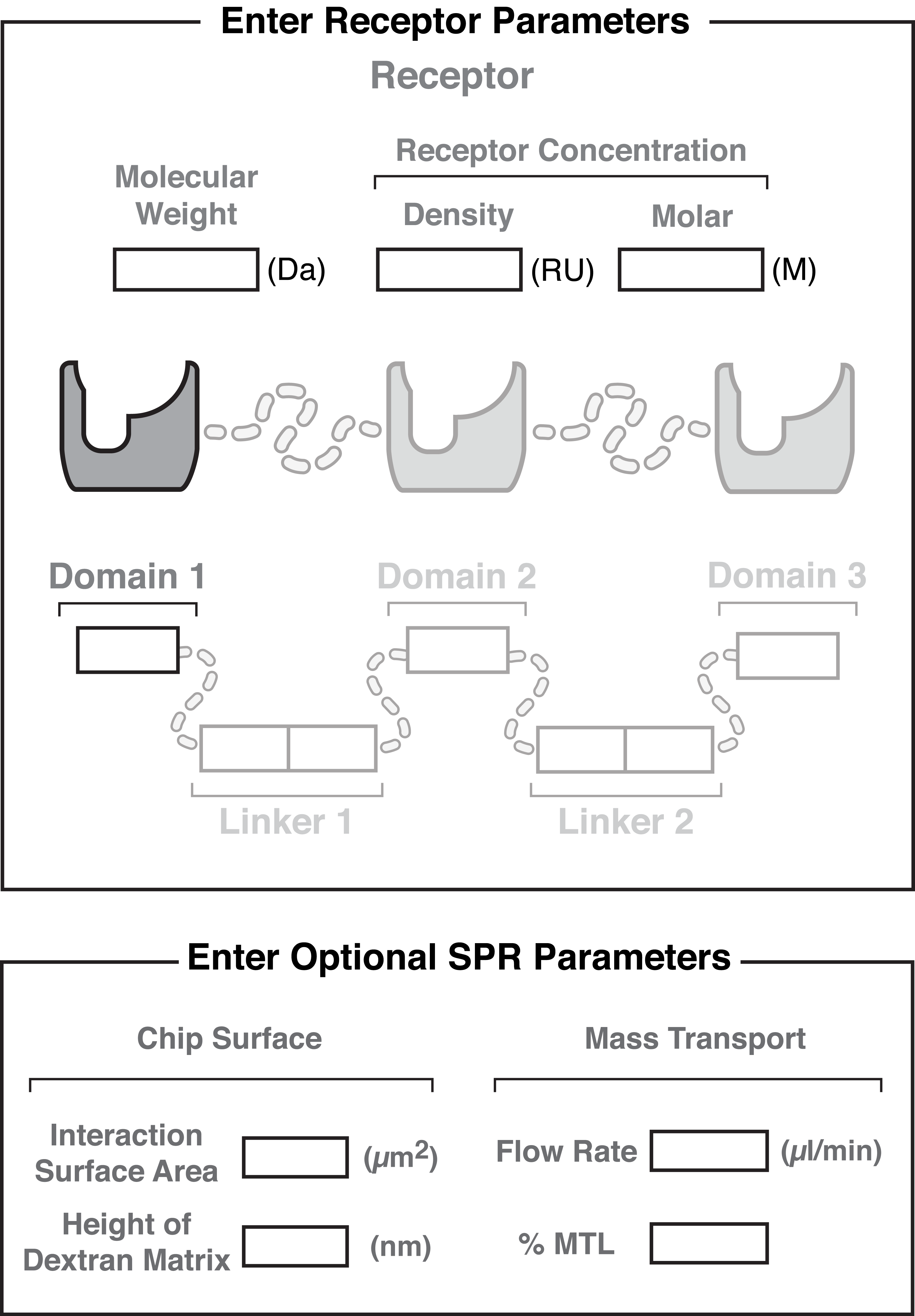

Supplement: Supplementary file 4 — Supplementary Software [file 41467_2022_32496_MOESM4_ESM.zip › Images/Connection_tab_images/GUI Rec_Tab_R1.png]

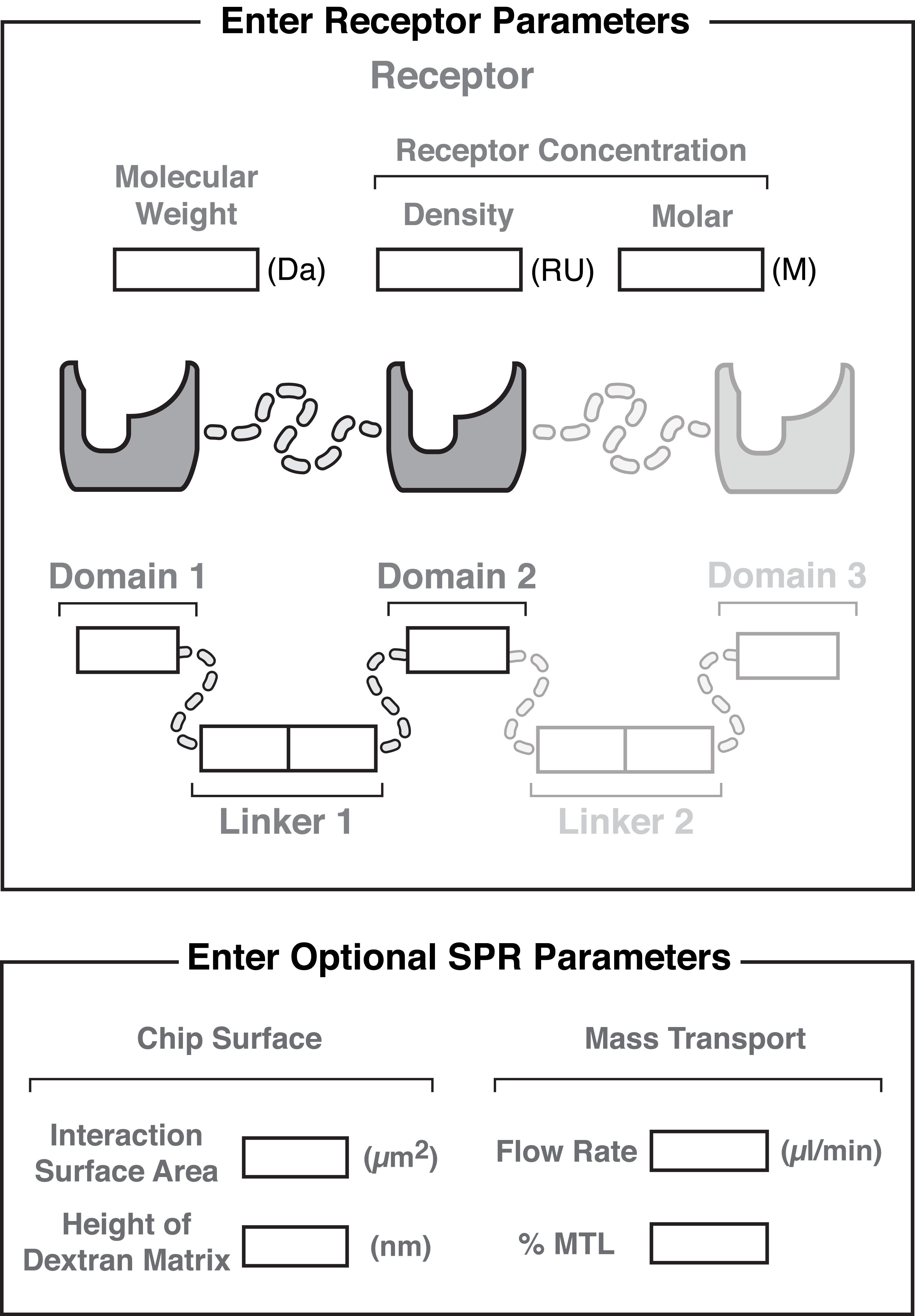

Supplement: Supplementary file 4 — Supplementary Software [file 41467_2022_32496_MOESM4_ESM.zip › Images/Connection_tab_images/GUI Rec_Tab_R2.png]

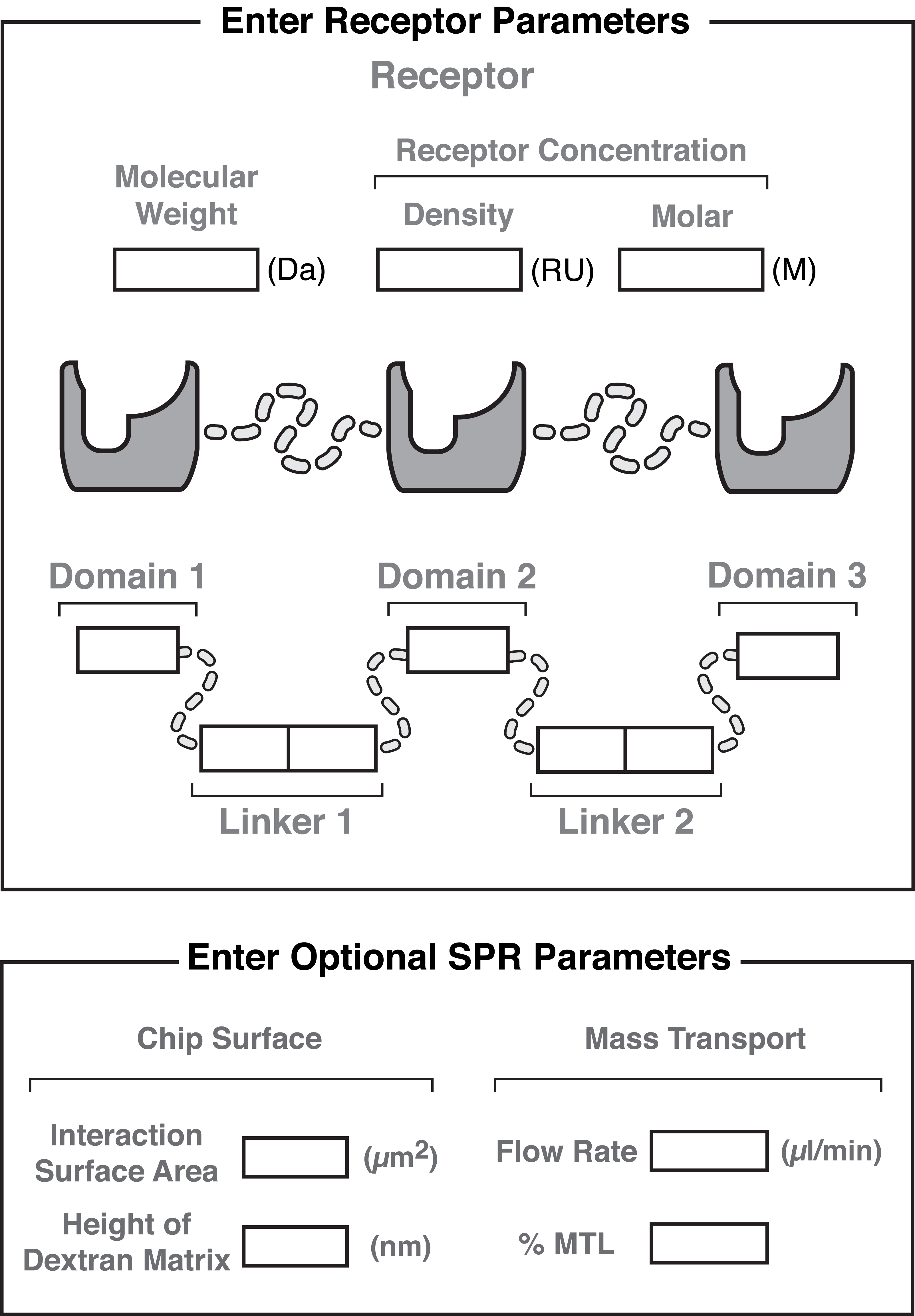

Supplement: Supplementary file 4 — Supplementary Software [file 41467_2022_32496_MOESM4_ESM.zip › Images/Connection_tab_images/GUI Rec_Tab_R3.png]

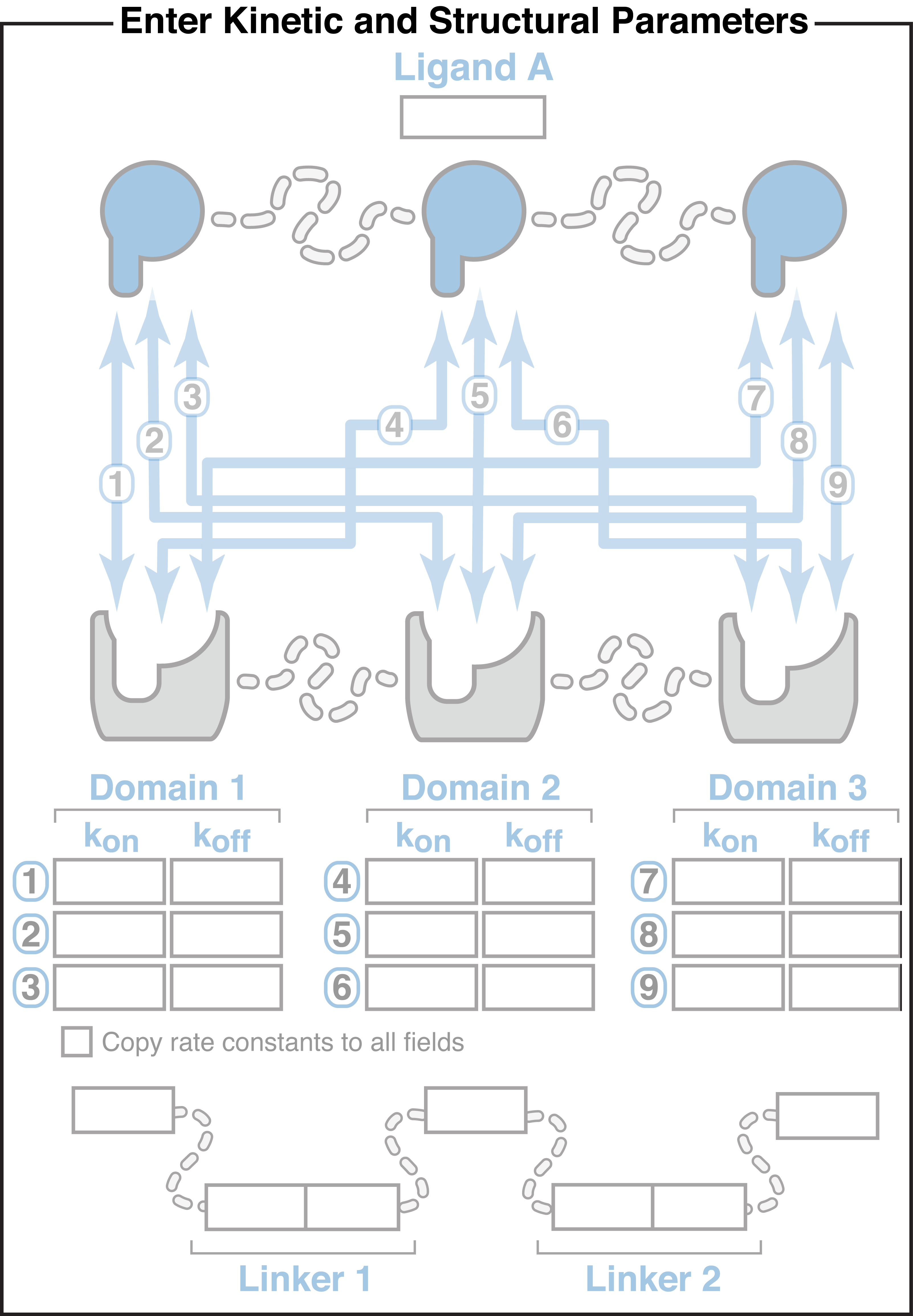

Supplement: Supplementary file 4 — Supplementary Software [file 41467_2022_32496_MOESM4_ESM.zip › Images/Connection_tab_images/MK_Tab2_A0x1.png]

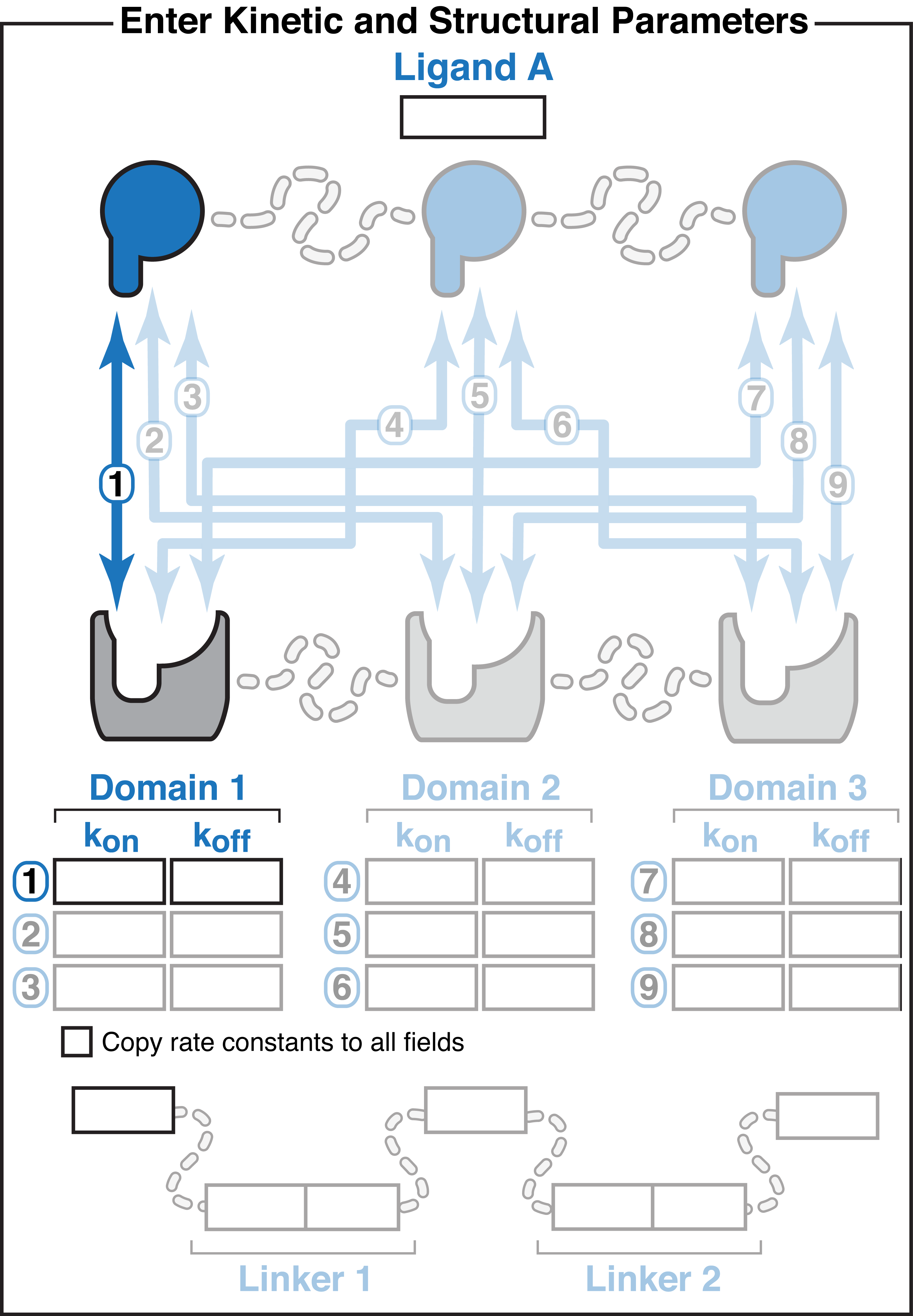

Supplement: Supplementary file 4 — Supplementary Software [file 41467_2022_32496_MOESM4_ESM.zip › Images/Connection_tab_images/MK_Tab2_A1x1.png]

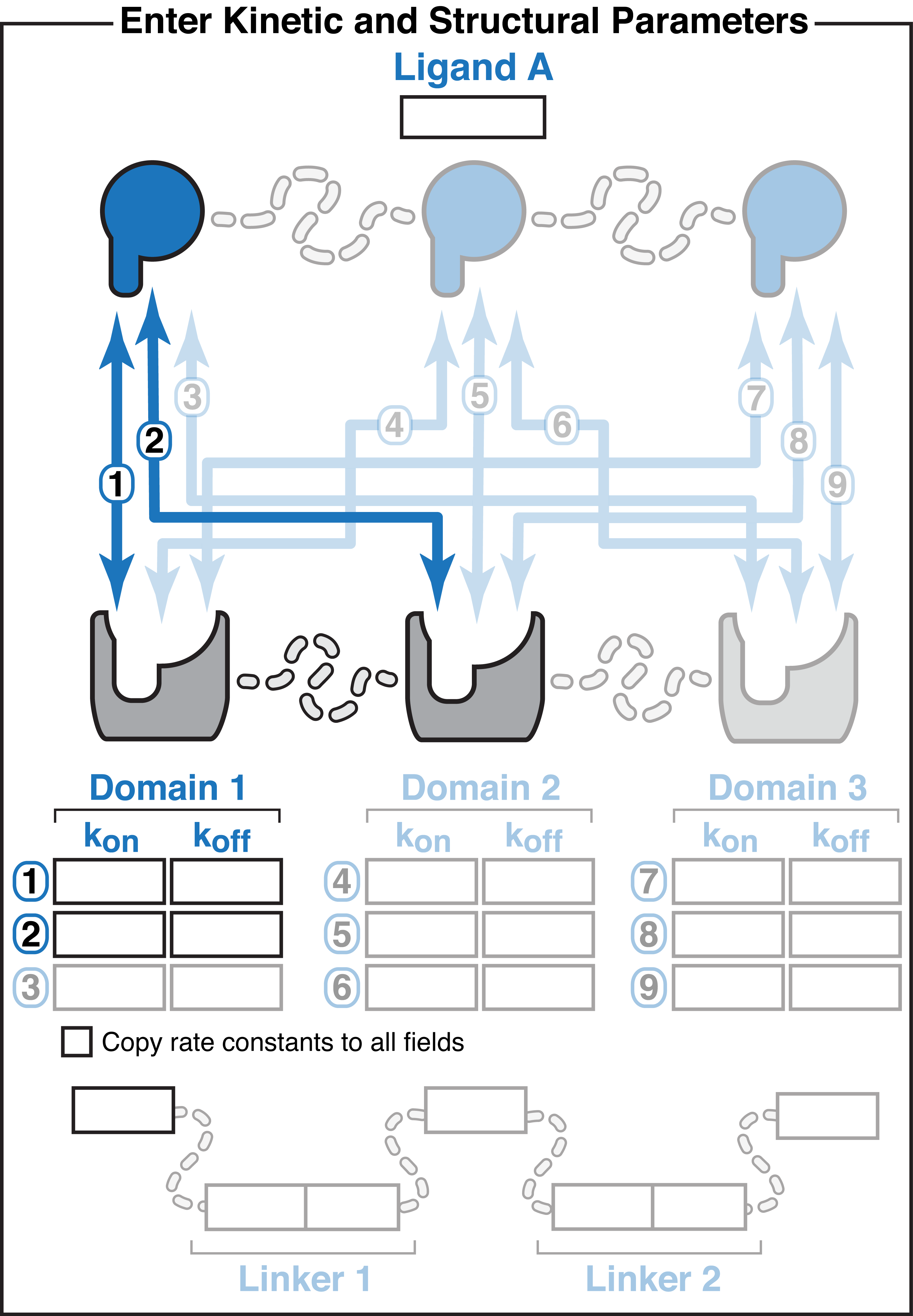

Supplement: Supplementary file 4 — Supplementary Software [file 41467_2022_32496_MOESM4_ESM.zip › Images/Connection_tab_images/MK_Tab2_A1x2.png]

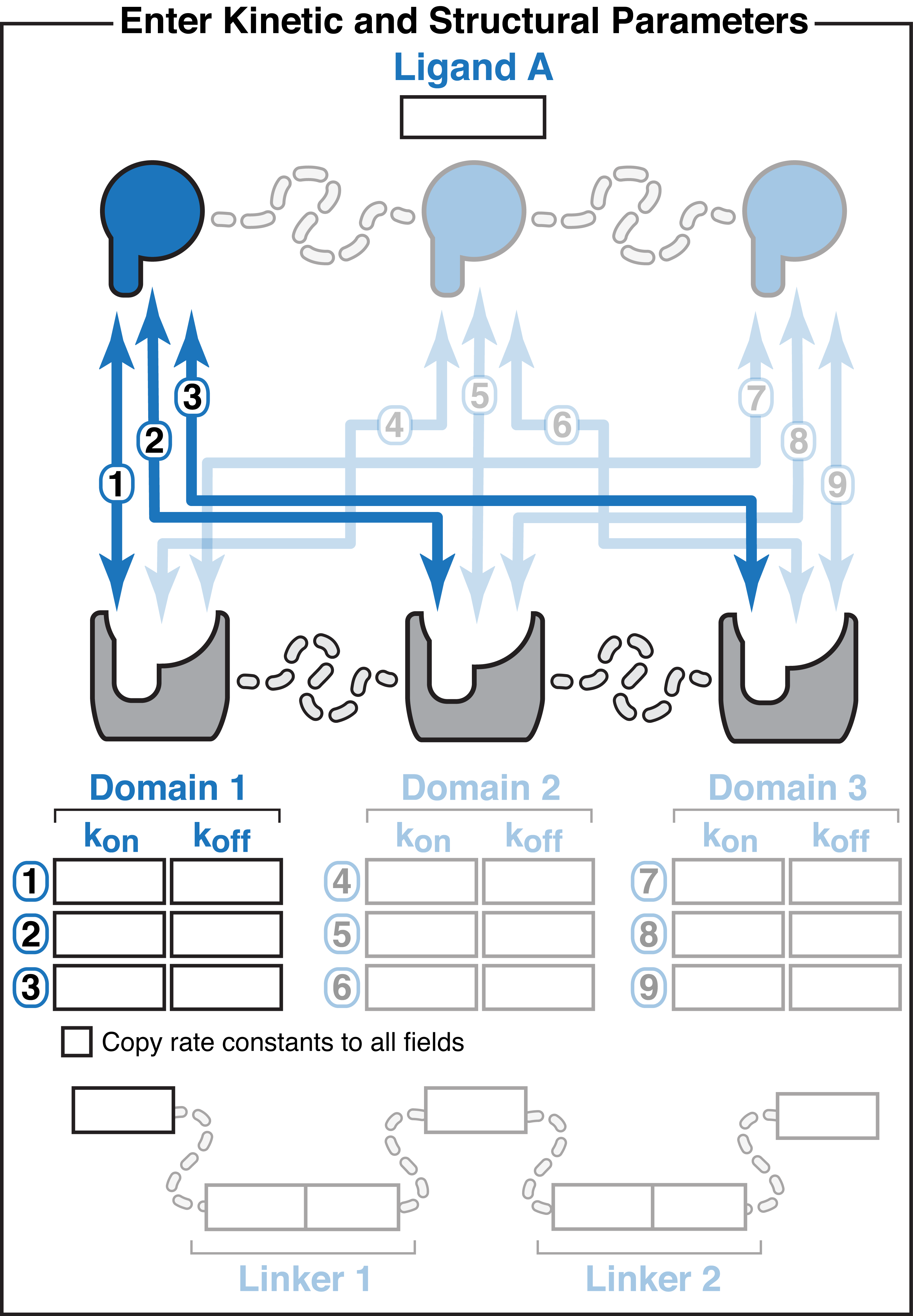

Supplement: Supplementary file 4 — Supplementary Software [file 41467_2022_32496_MOESM4_ESM.zip › Images/Connection_tab_images/MK_Tab2_A1x3.png]

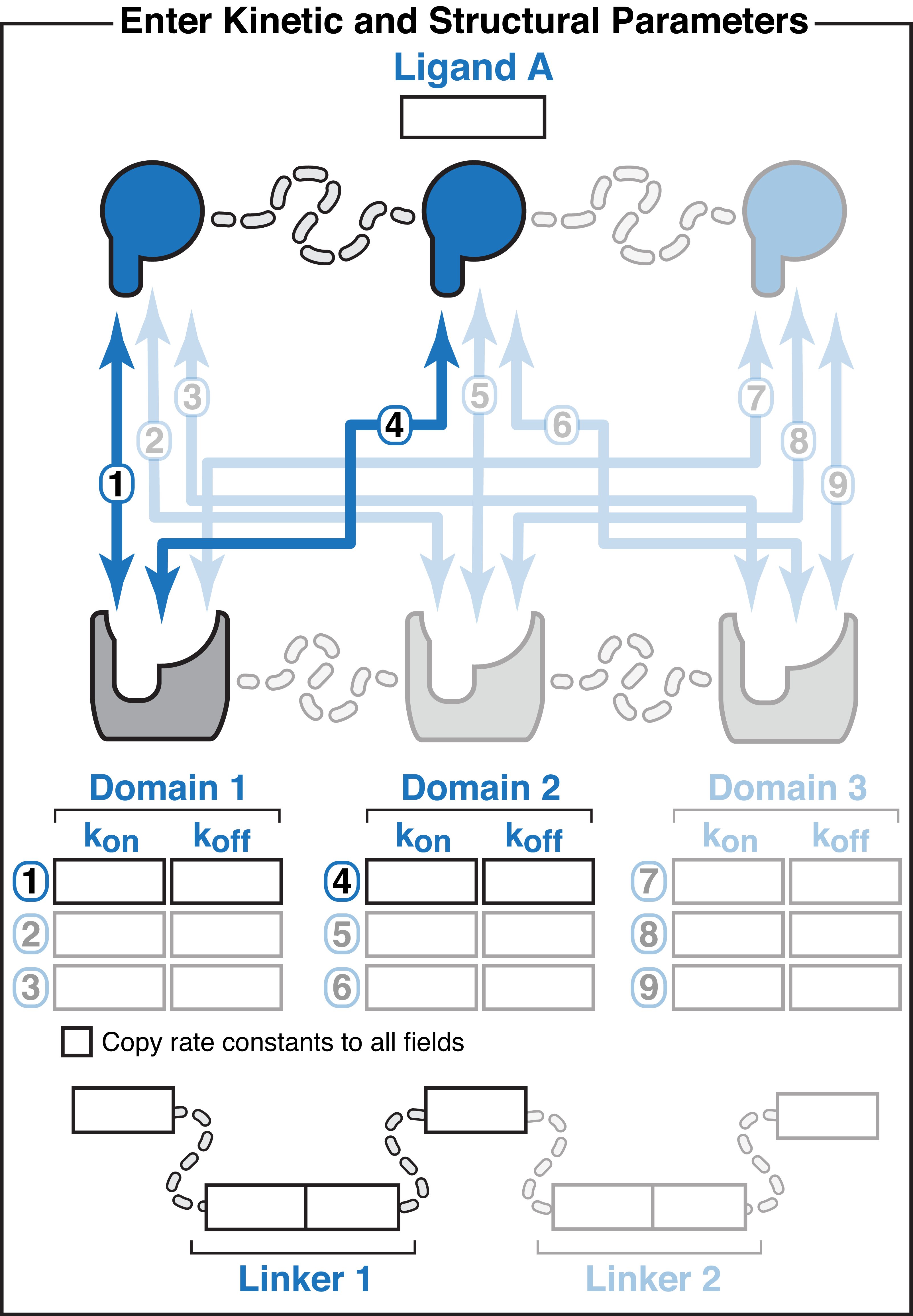

Supplement: Supplementary file 4 — Supplementary Software [file 41467_2022_32496_MOESM4_ESM.zip › Images/Connection_tab_images/MK_Tab2_A2x1.png]

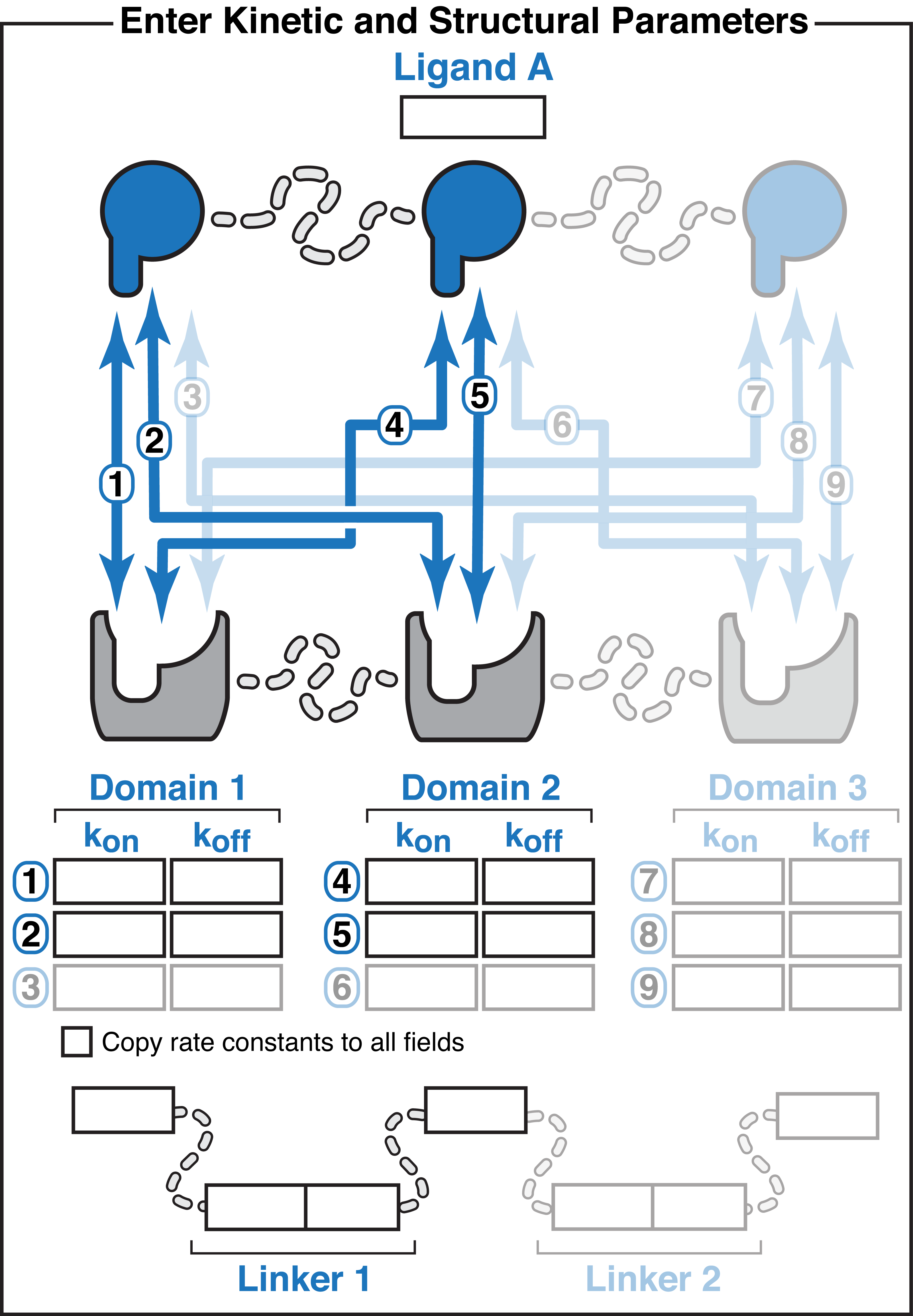

Supplement: Supplementary file 4 — Supplementary Software [file 41467_2022_32496_MOESM4_ESM.zip › Images/Connection_tab_images/MK_Tab2_A2x2.png]

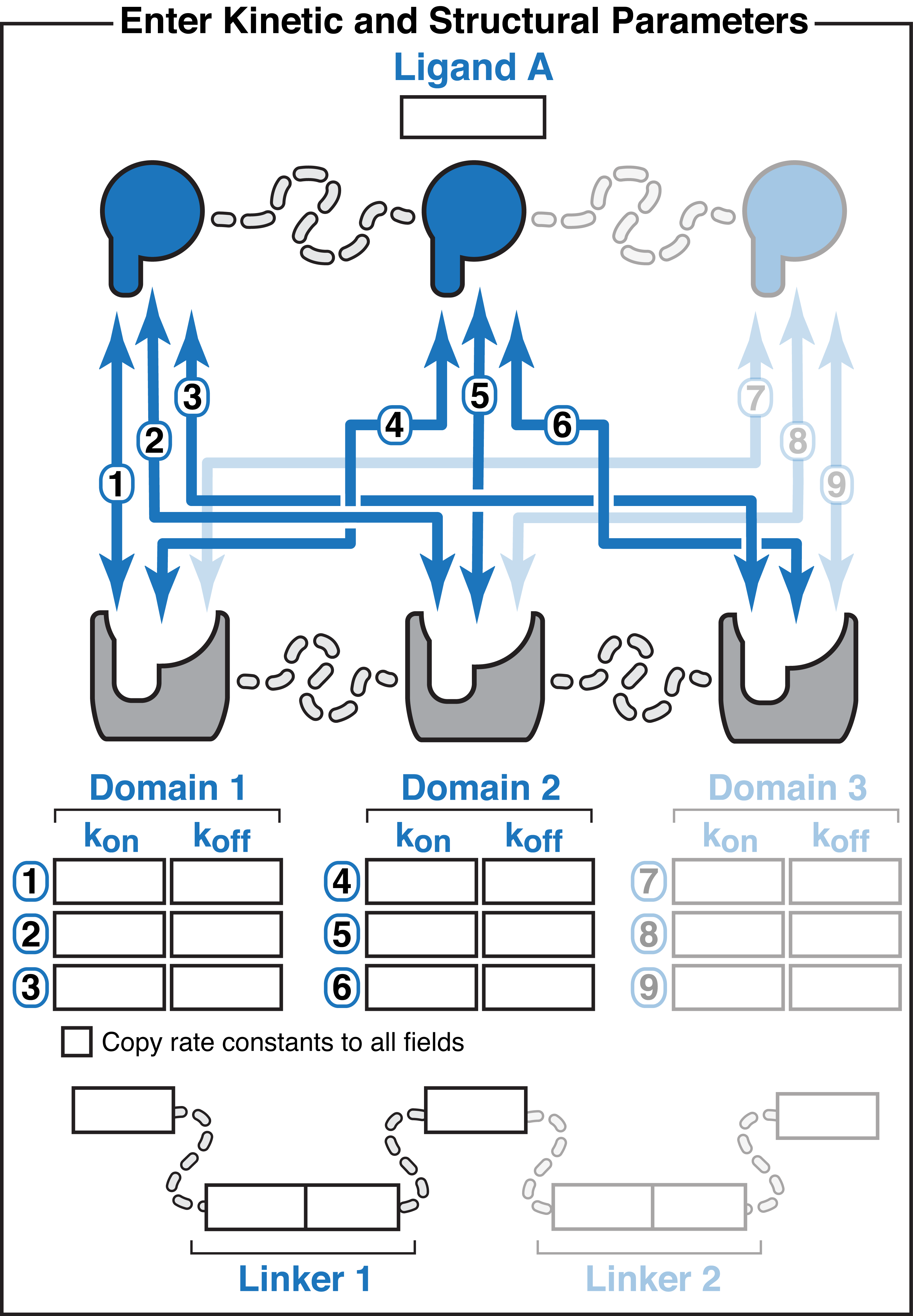

Supplement: Supplementary file 4 — Supplementary Software [file 41467_2022_32496_MOESM4_ESM.zip › Images/Connection_tab_images/MK_Tab2_A2x3.png]

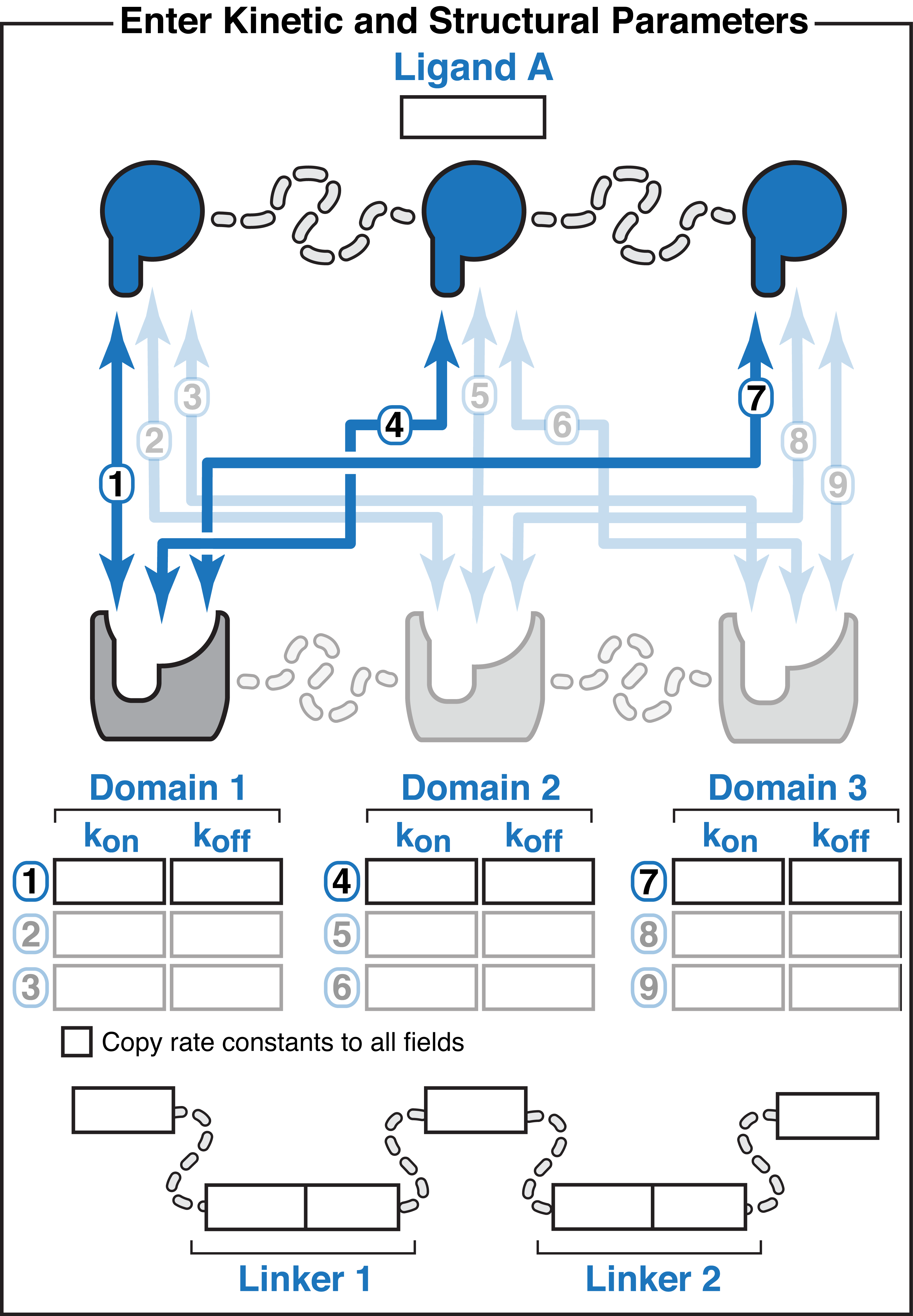

Supplement: Supplementary file 4 — Supplementary Software [file 41467_2022_32496_MOESM4_ESM.zip › Images/Connection_tab_images/MK_Tab2_A3x1.png]

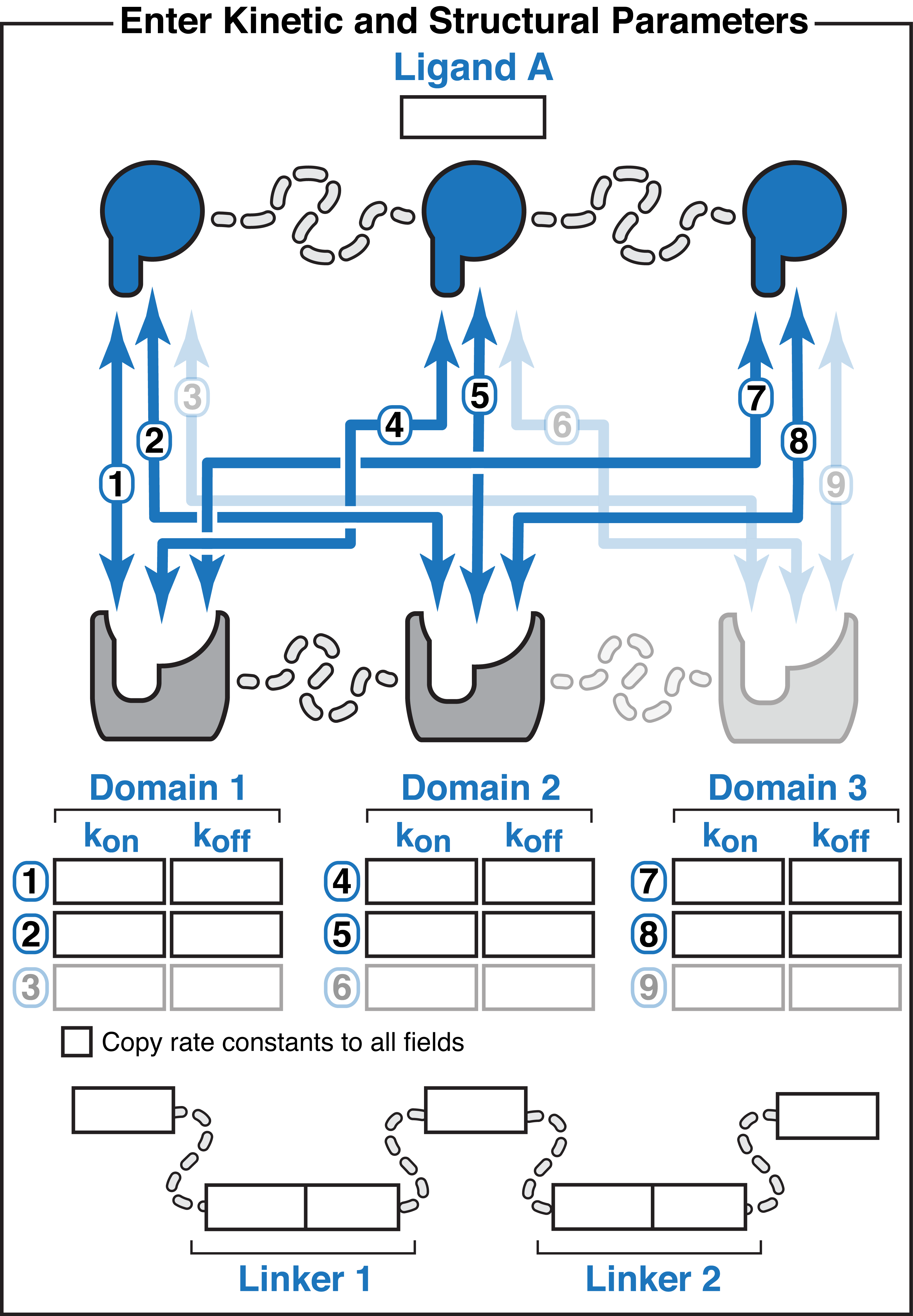

Supplement: Supplementary file 4 — Supplementary Software [file 41467_2022_32496_MOESM4_ESM.zip › Images/Connection_tab_images/MK_Tab2_A3x2.png]

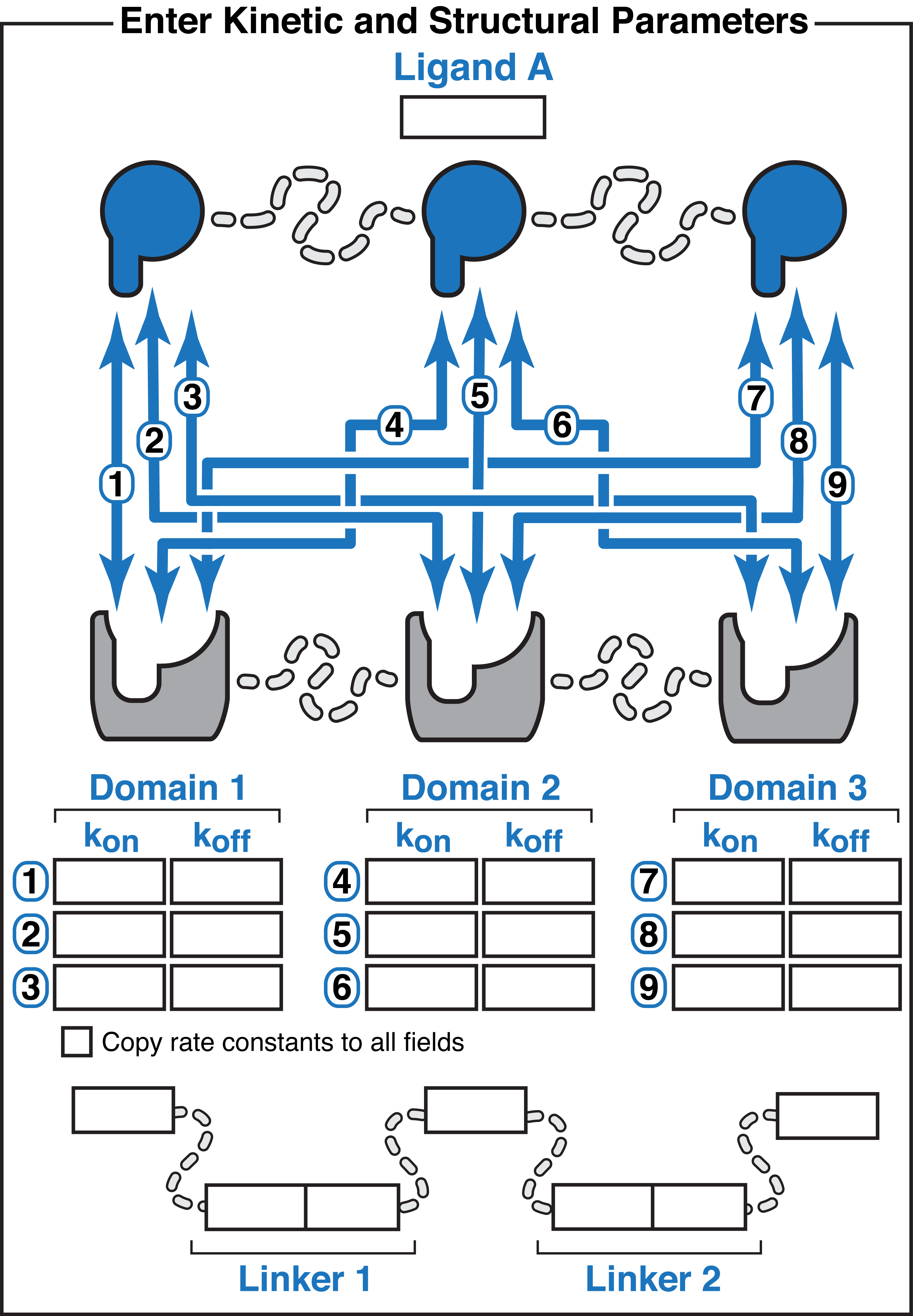

Supplement: Supplementary file 4 — Supplementary Software [file 41467_2022_32496_MOESM4_ESM.zip › Images/Connection_tab_images/MK_Tab2_A3x3.png]

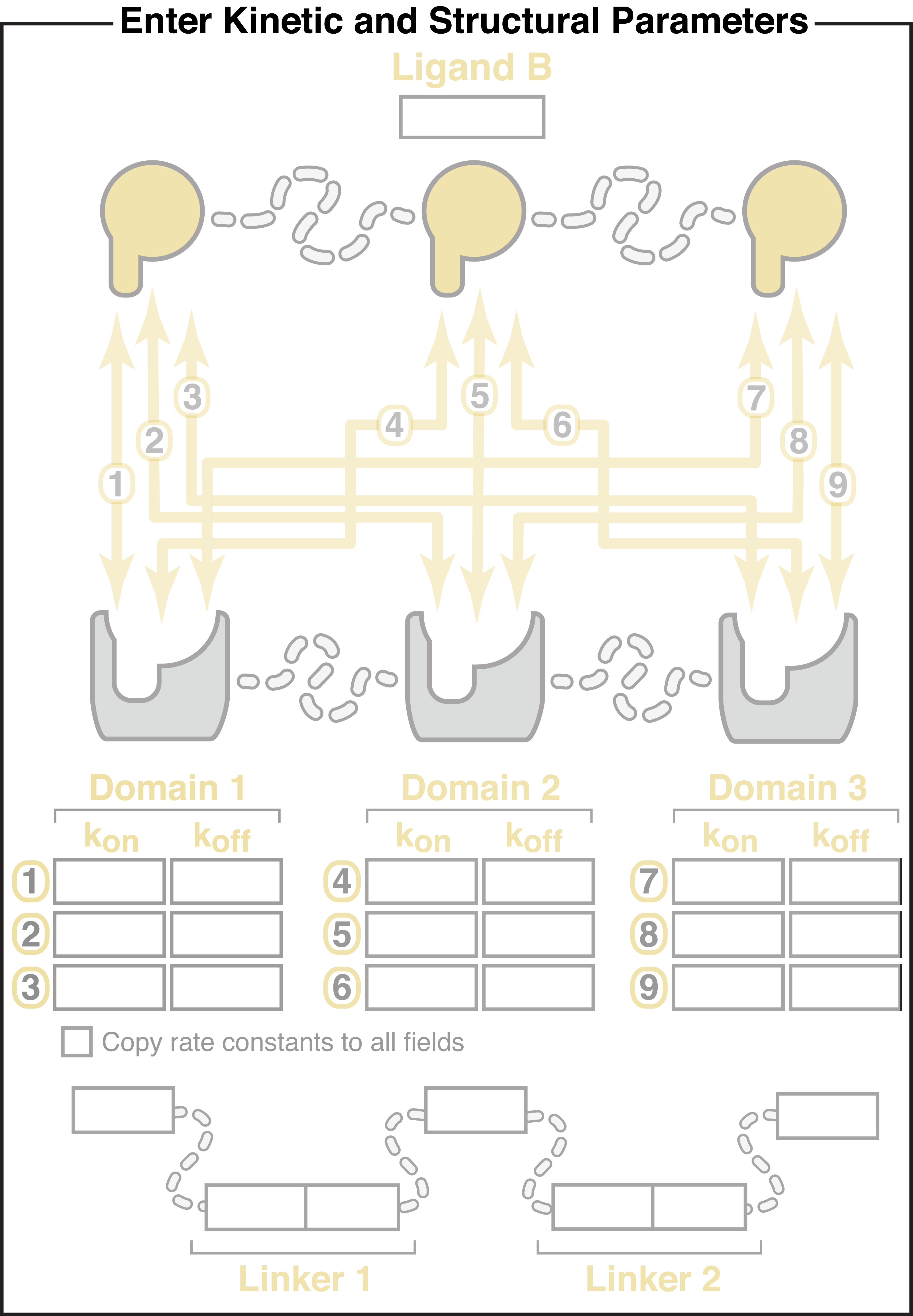

Supplement: Supplementary file 4 — Supplementary Software [file 41467_2022_32496_MOESM4_ESM.zip › Images/Connection_tab_images/MK_Tab2_B0x1.png]

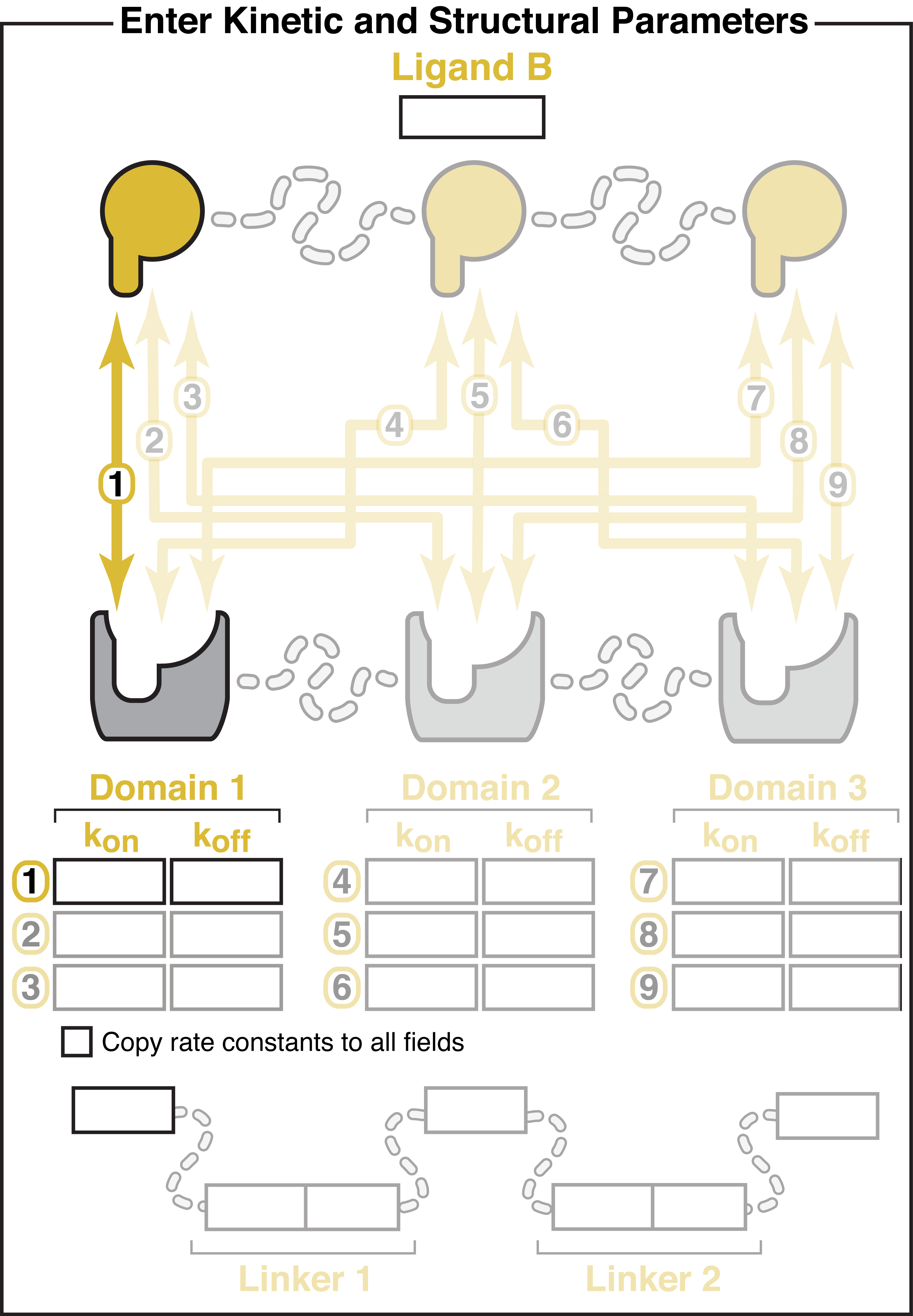

Supplement: Supplementary file 4 — Supplementary Software [file 41467_2022_32496_MOESM4_ESM.zip › Images/Connection_tab_images/MK_Tab2_B1x1.png]

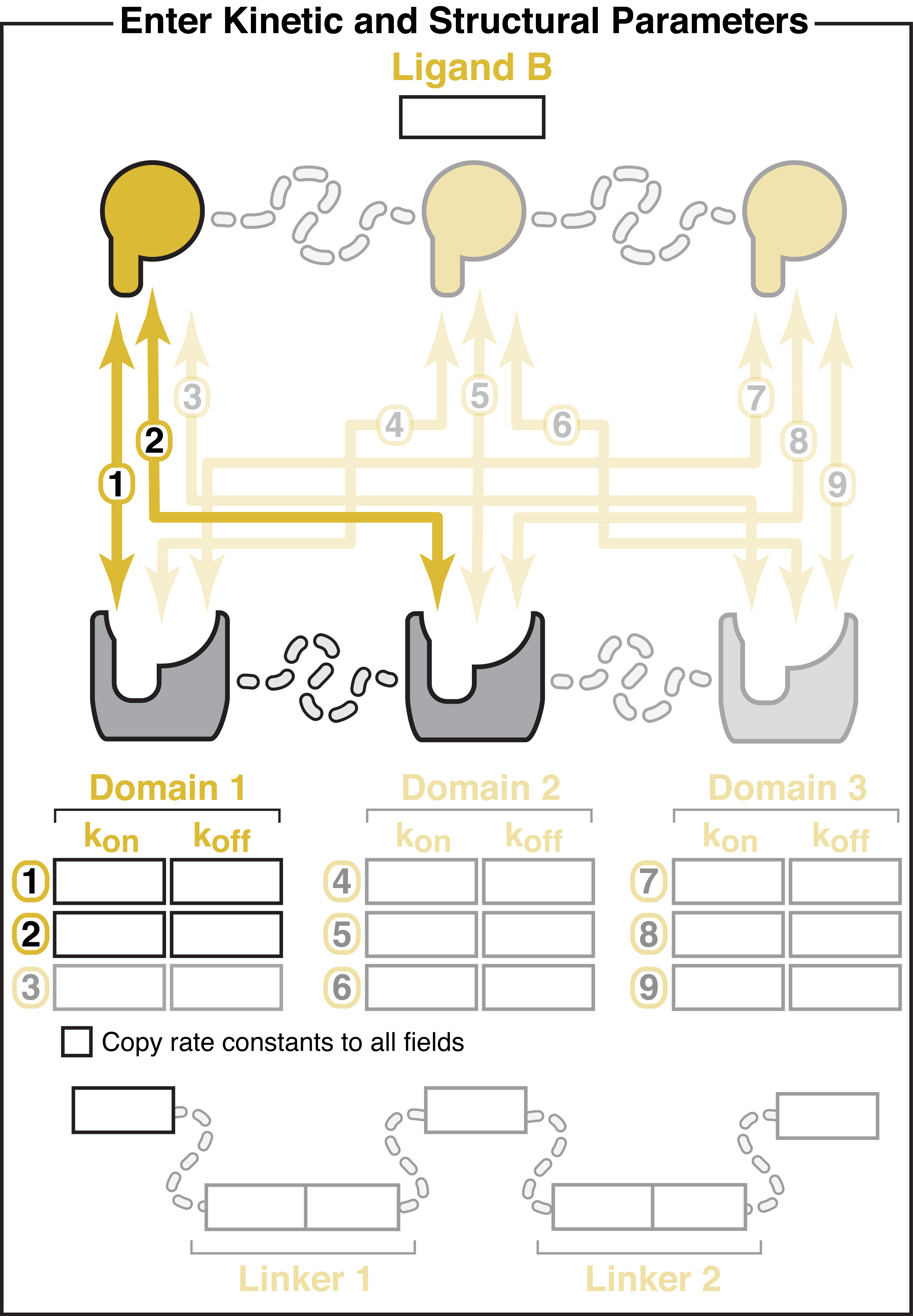

Supplement: Supplementary file 4 — Supplementary Software [file 41467_2022_32496_MOESM4_ESM.zip › Images/Connection_tab_images/MK_Tab2_B1x2.png]

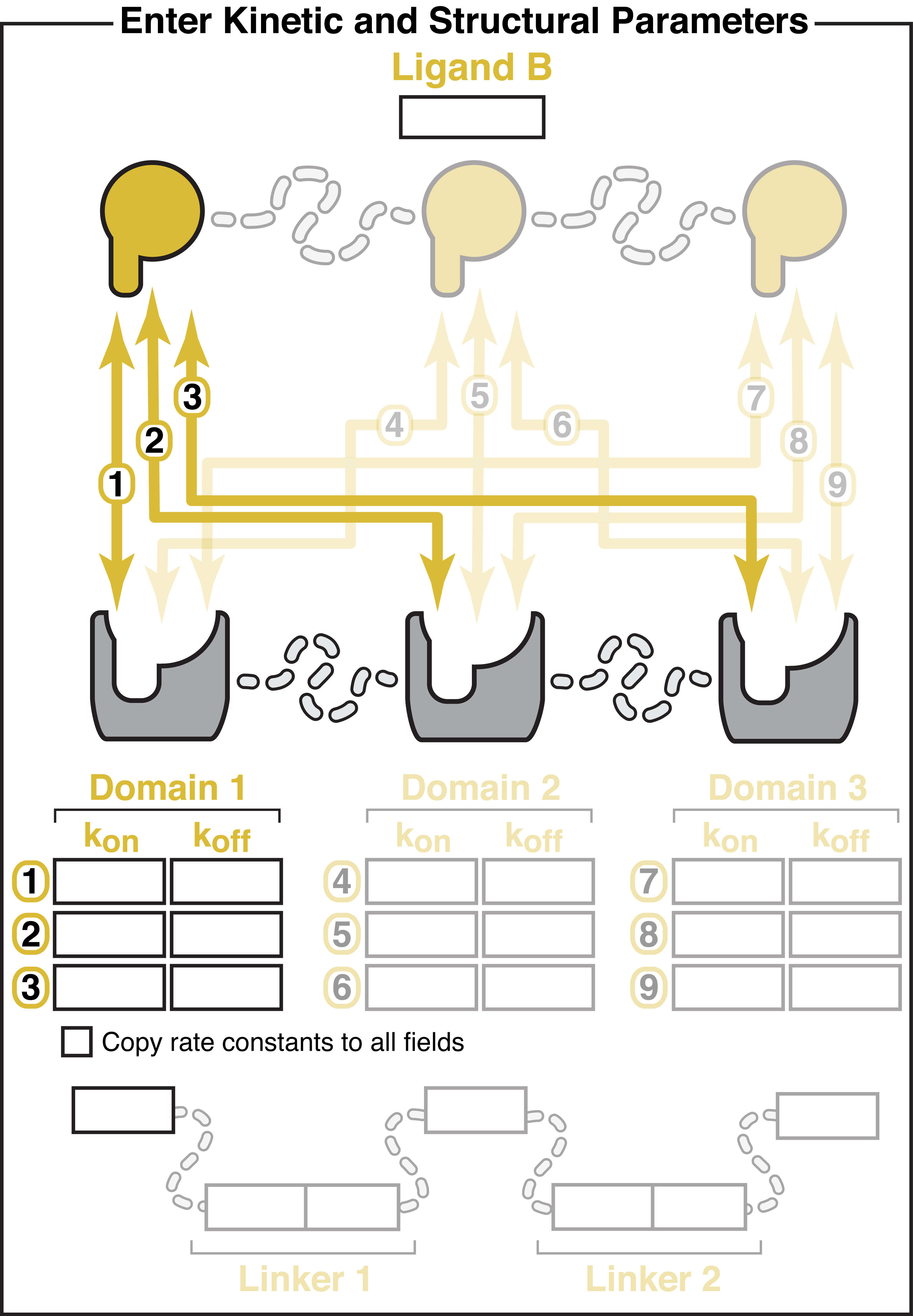

Supplement: Supplementary file 4 — Supplementary Software [file 41467_2022_32496_MOESM4_ESM.zip › Images/Connection_tab_images/MK_Tab2_B1x3.png]

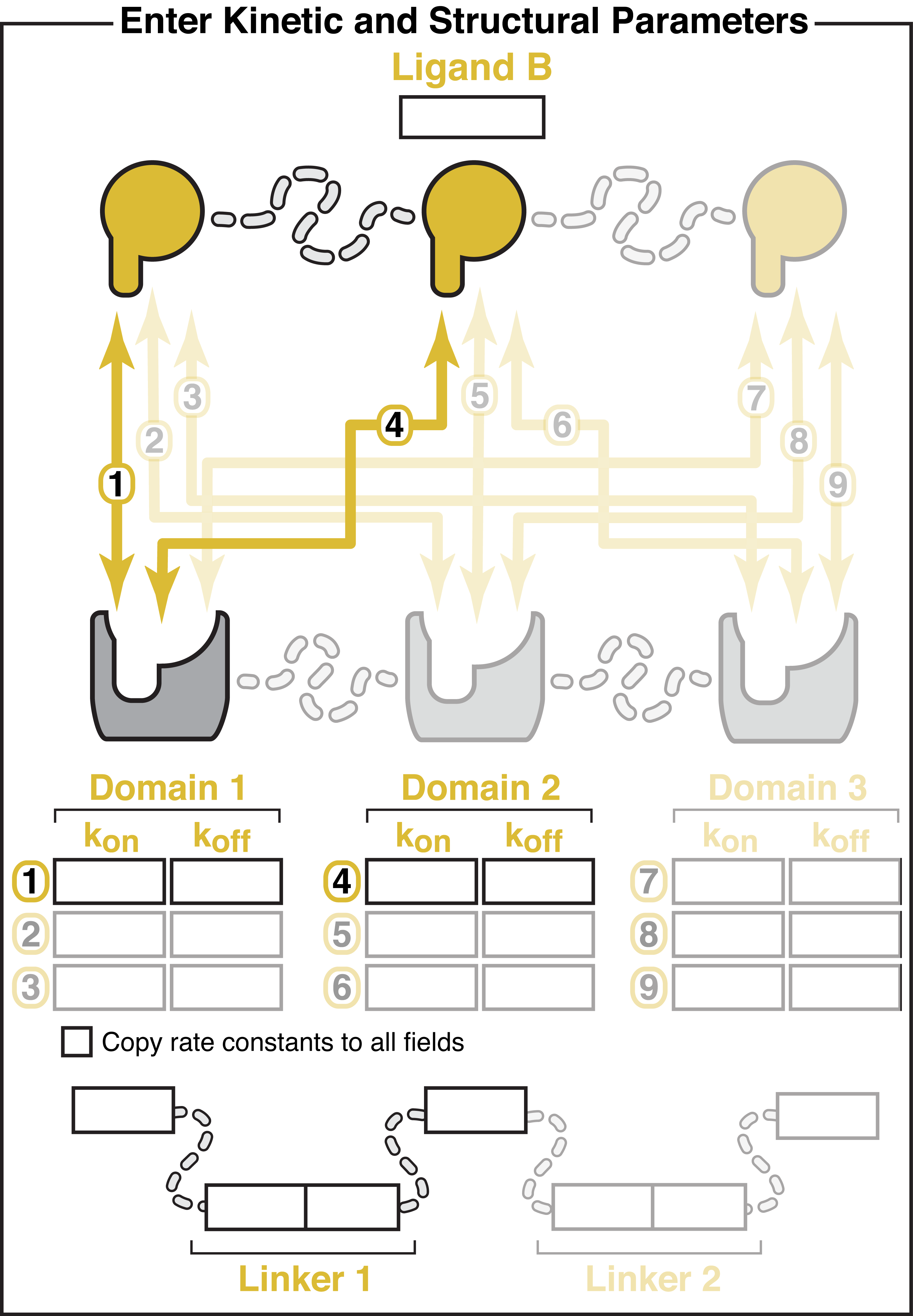

Supplement: Supplementary file 4 — Supplementary Software [file 41467_2022_32496_MOESM4_ESM.zip › Images/Connection_tab_images/MK_Tab2_B2x1.png]

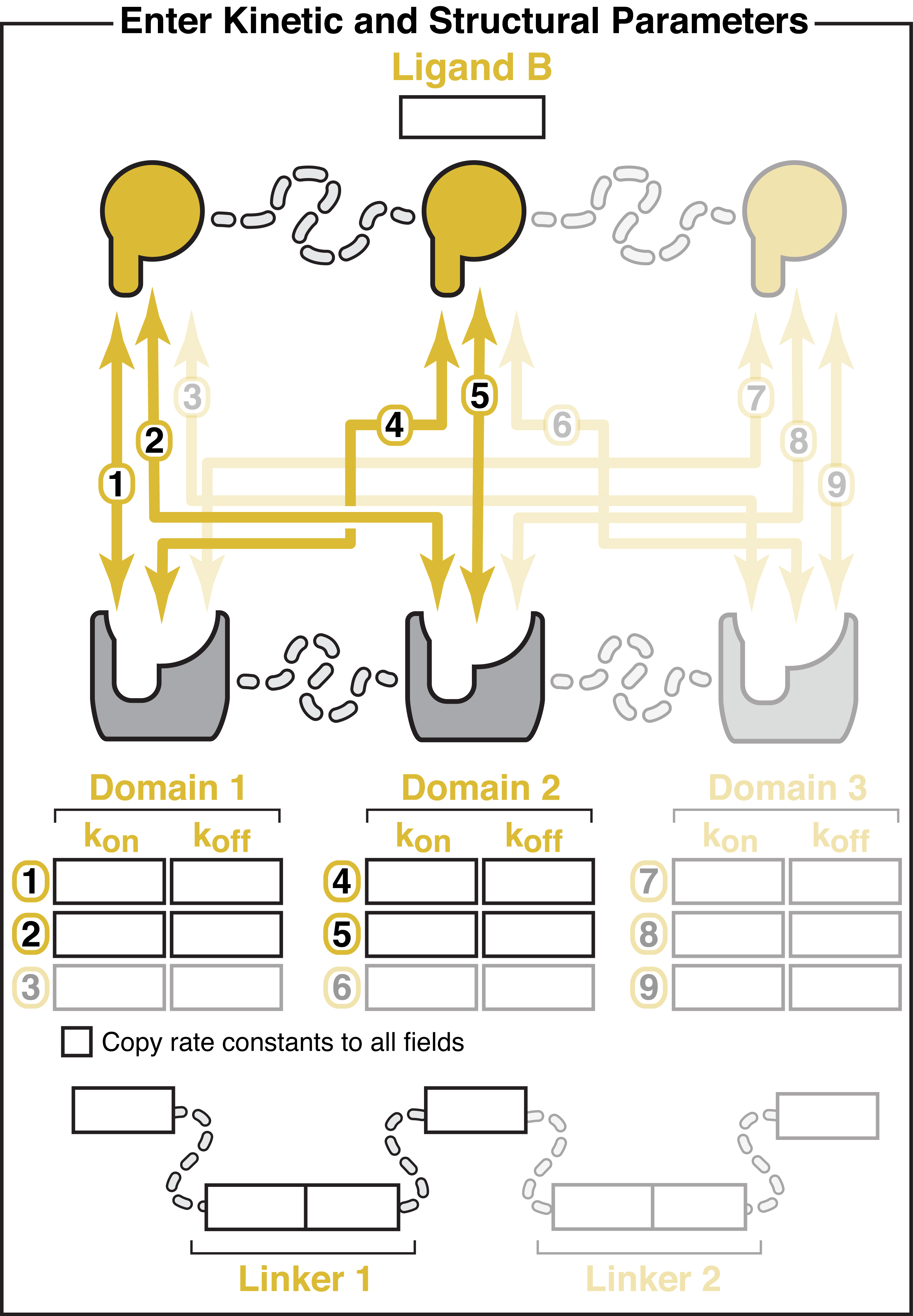

Supplement: Supplementary file 4 — Supplementary Software [file 41467_2022_32496_MOESM4_ESM.zip › Images/Connection_tab_images/MK_Tab2_B2x2.png]

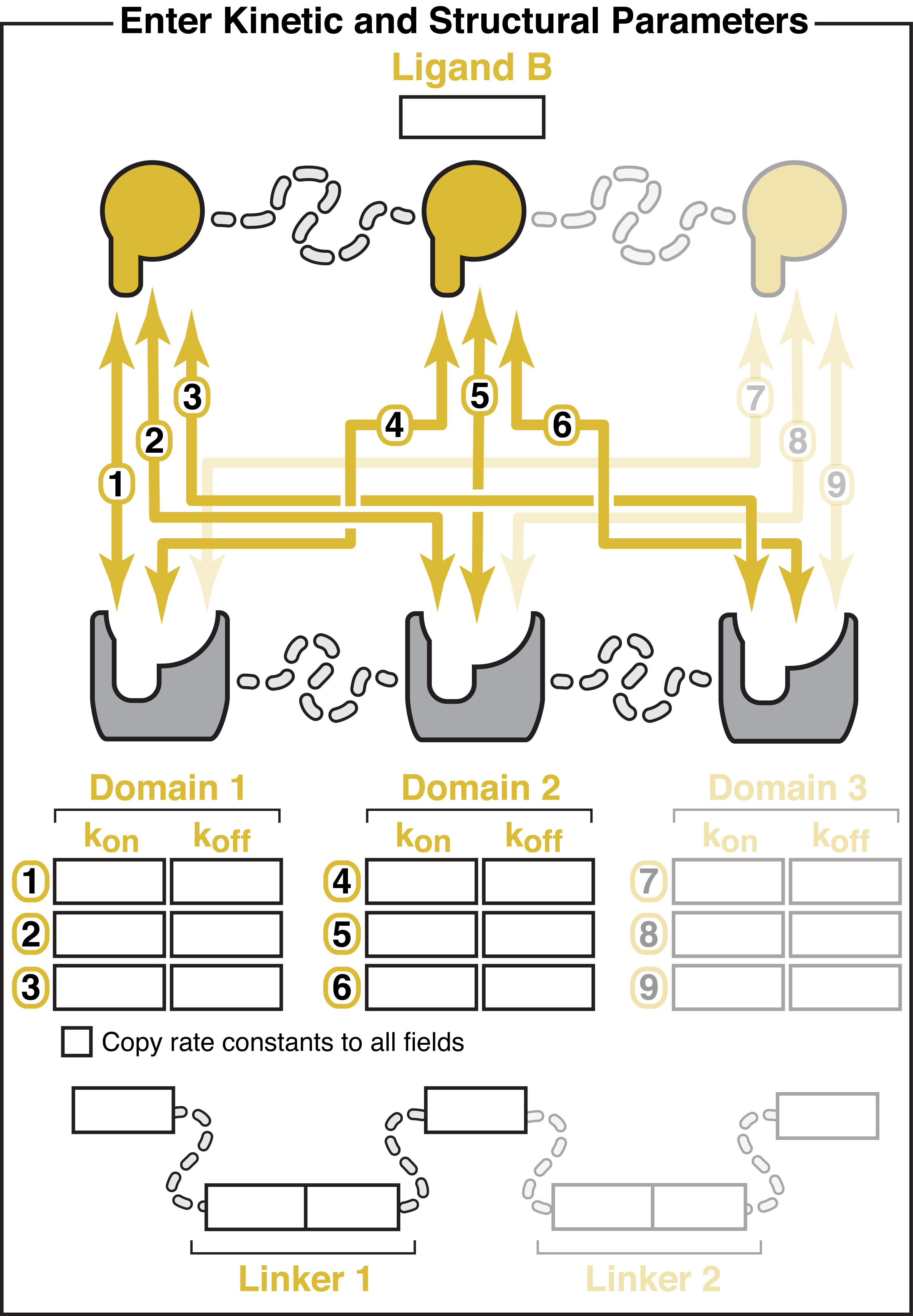

Supplement: Supplementary file 4 — Supplementary Software [file 41467_2022_32496_MOESM4_ESM.zip › Images/Connection_tab_images/MK_Tab2_B2x3.png]

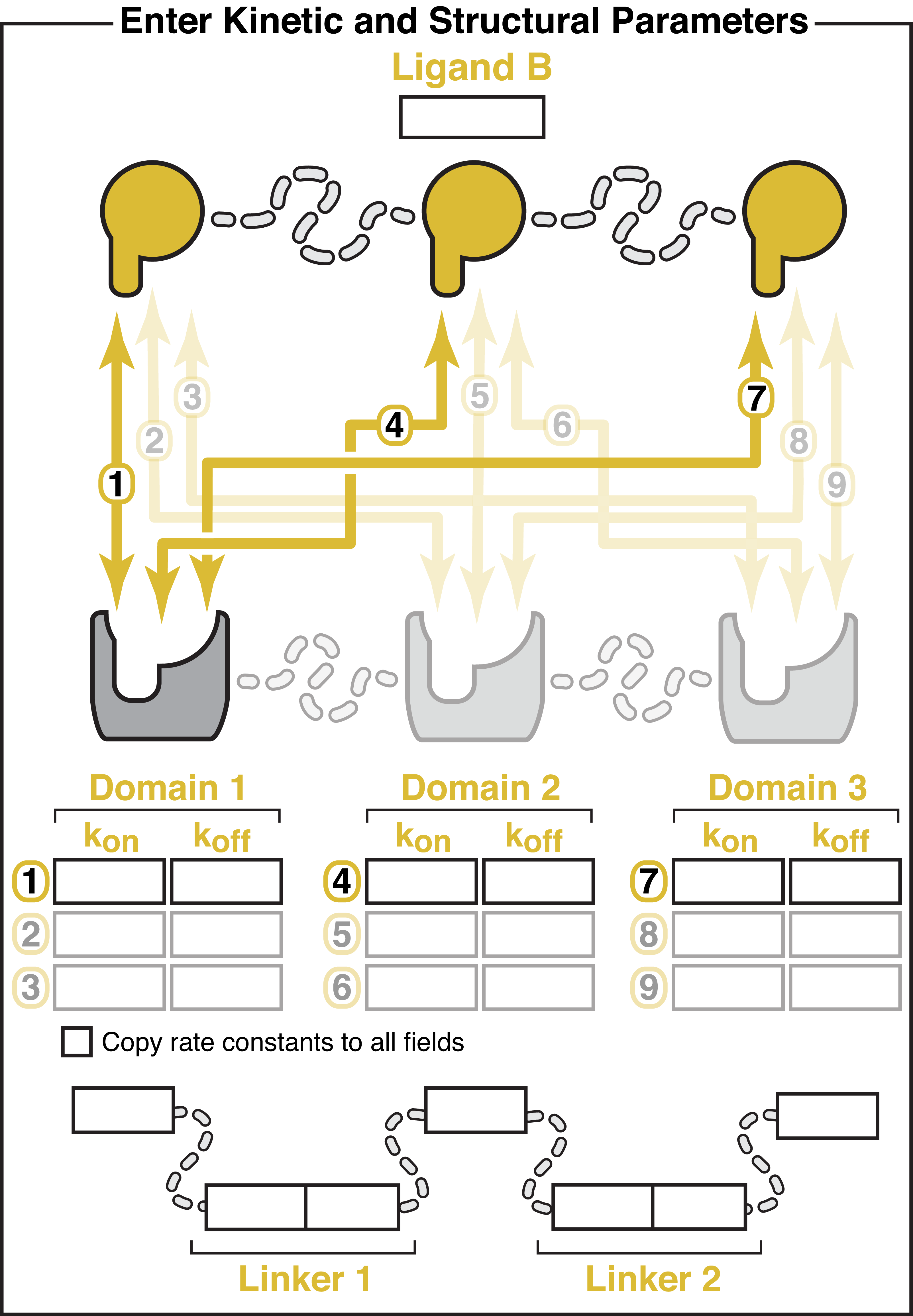

Supplement: Supplementary file 4 — Supplementary Software [file 41467_2022_32496_MOESM4_ESM.zip › Images/Connection_tab_images/MK_Tab2_B3x1.png]

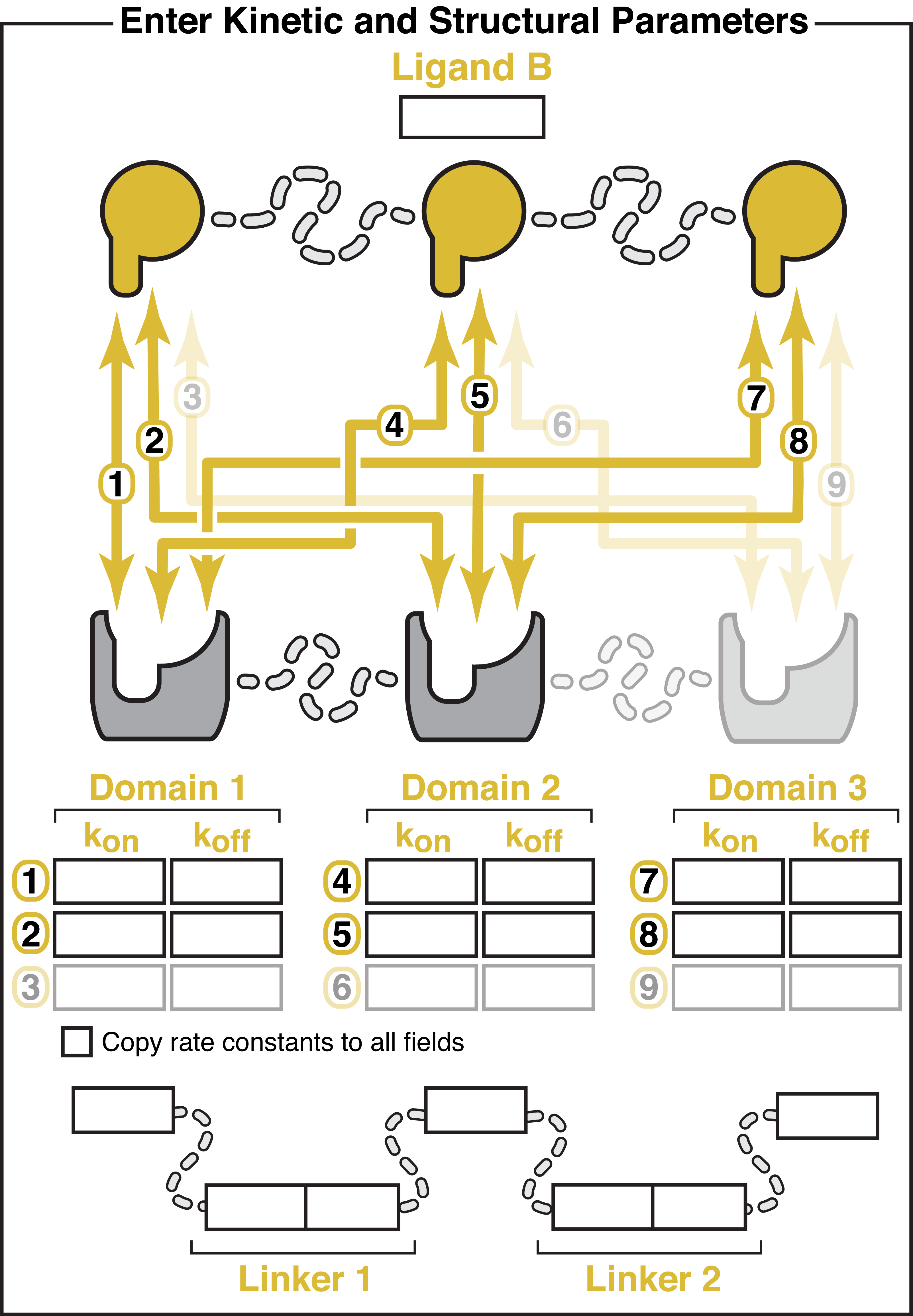

Supplement: Supplementary file 4 — Supplementary Software [file 41467_2022_32496_MOESM4_ESM.zip › Images/Connection_tab_images/MK_Tab2_B3x2.png]

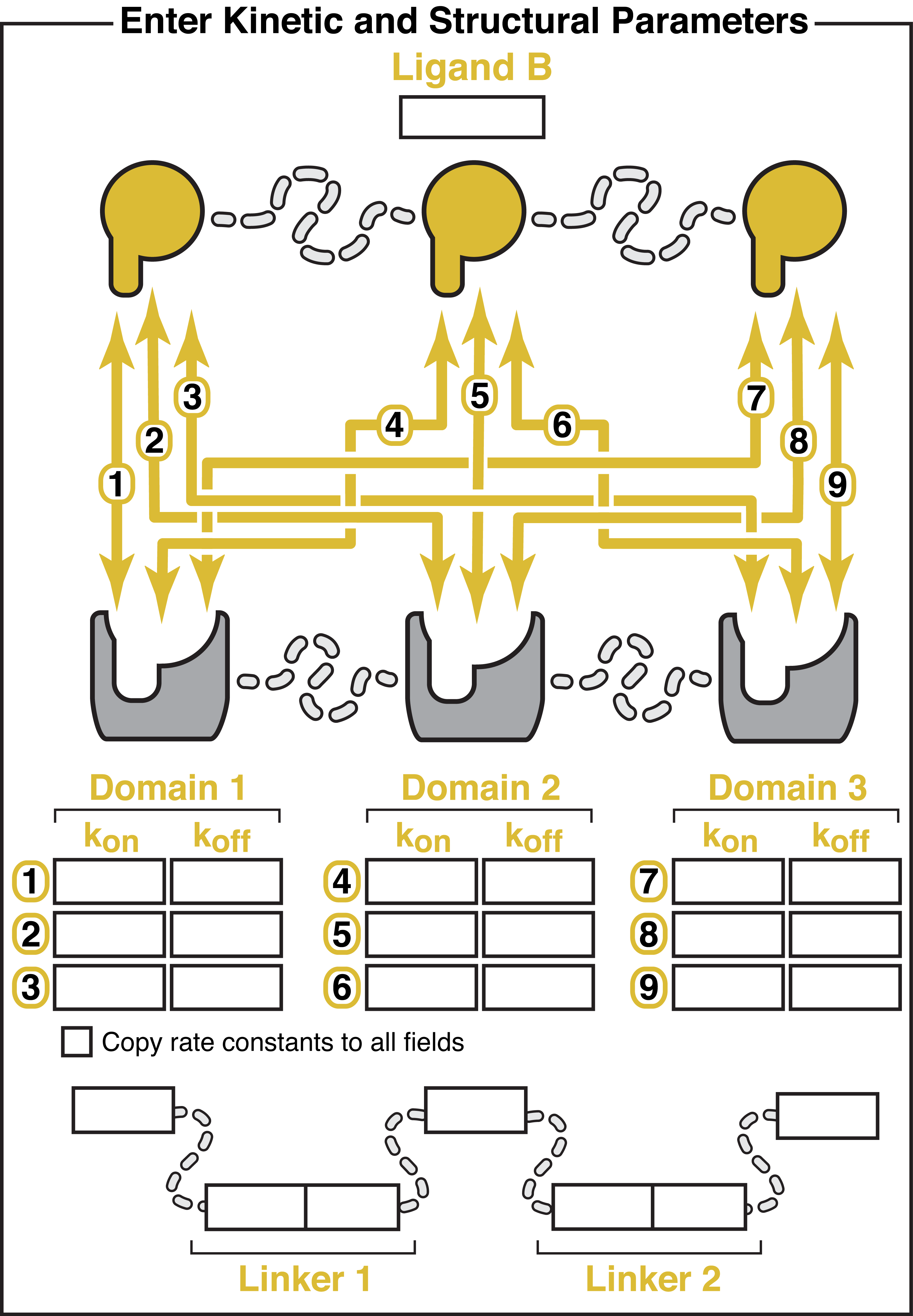

Supplement: Supplementary file 4 — Supplementary Software [file 41467_2022_32496_MOESM4_ESM.zip › Images/Connection_tab_images/MK_Tab2_B3x3.png]

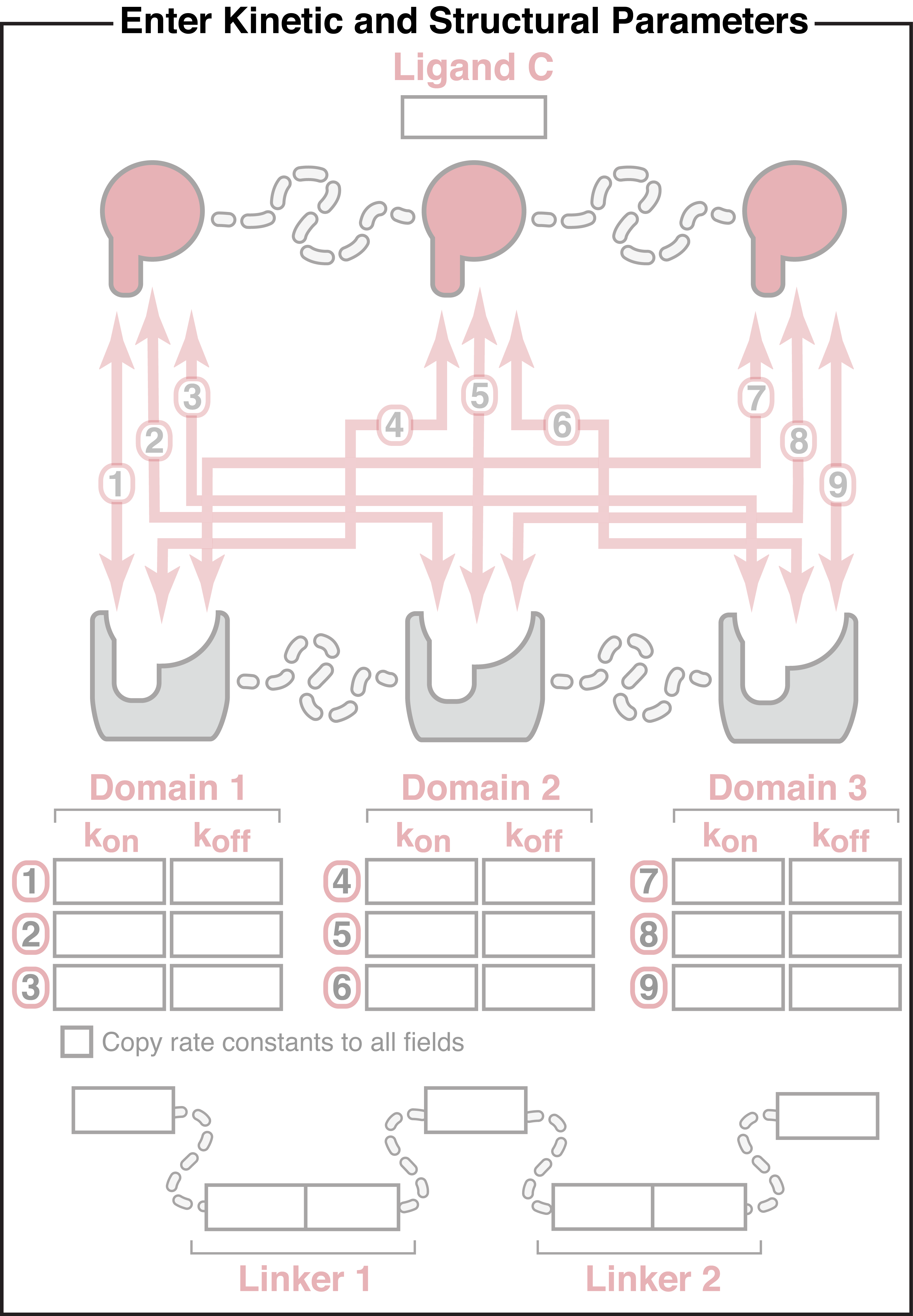

Supplement: Supplementary file 4 — Supplementary Software [file 41467_2022_32496_MOESM4_ESM.zip › Images/Connection_tab_images/MK_Tab2_C0x1.png]

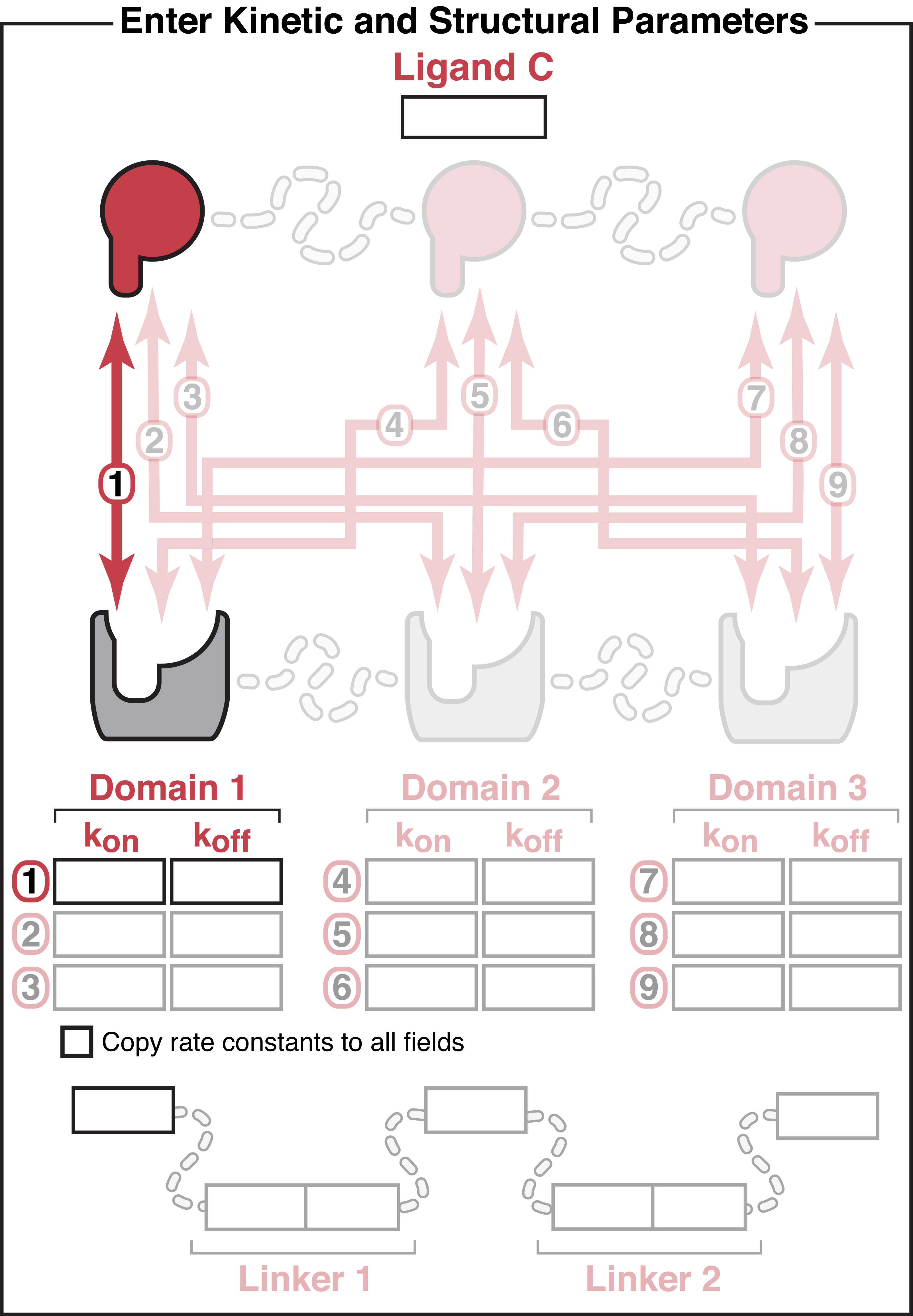

Supplement: Supplementary file 4 — Supplementary Software [file 41467_2022_32496_MOESM4_ESM.zip › Images/Connection_tab_images/MK_Tab2_C1x1.png]

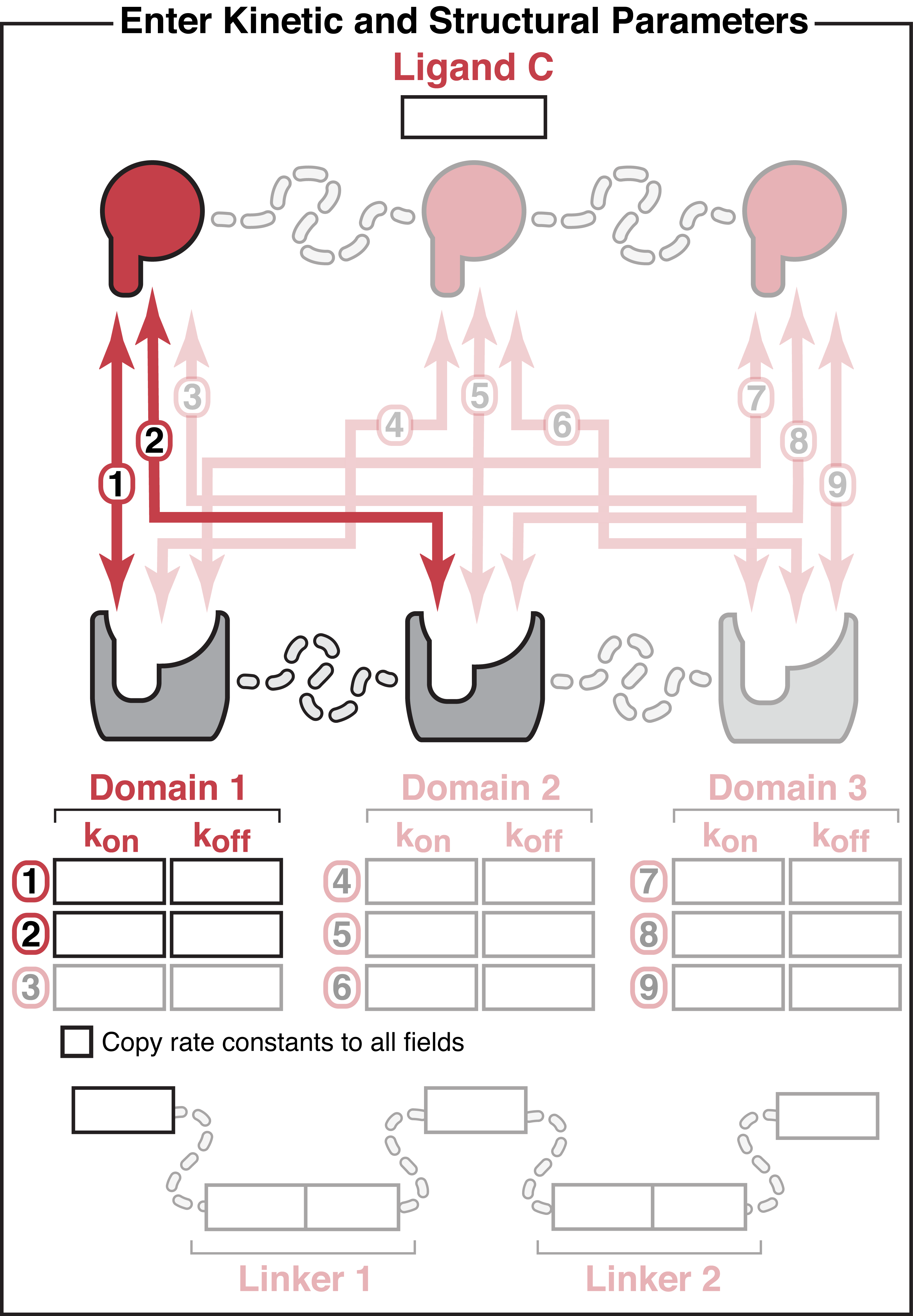

Supplement: Supplementary file 4 — Supplementary Software [file 41467_2022_32496_MOESM4_ESM.zip › Images/Connection_tab_images/MK_Tab2_C1x2.png]

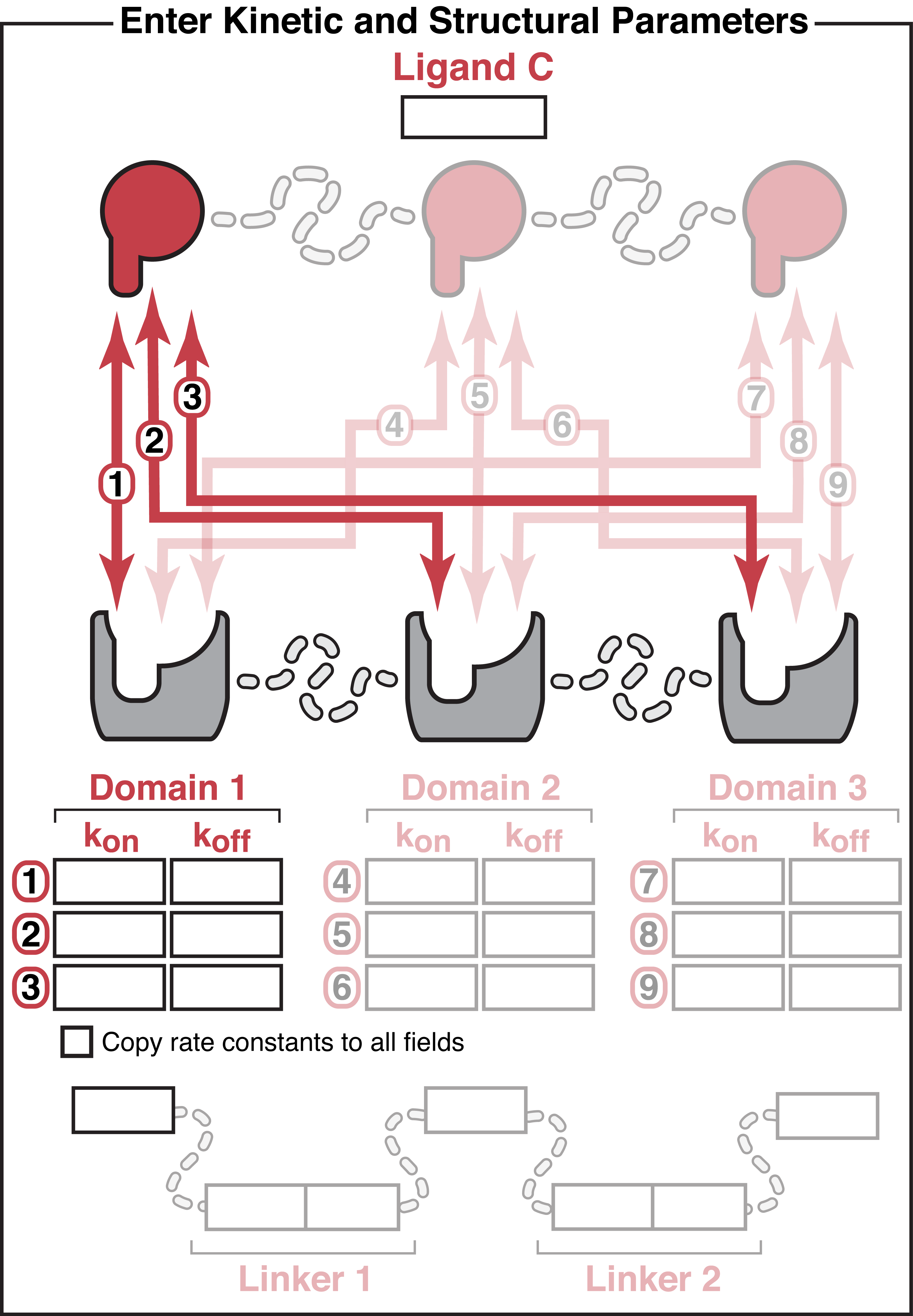

Supplement: Supplementary file 4 — Supplementary Software [file 41467_2022_32496_MOESM4_ESM.zip › Images/Connection_tab_images/MK_Tab2_C1x3.png]

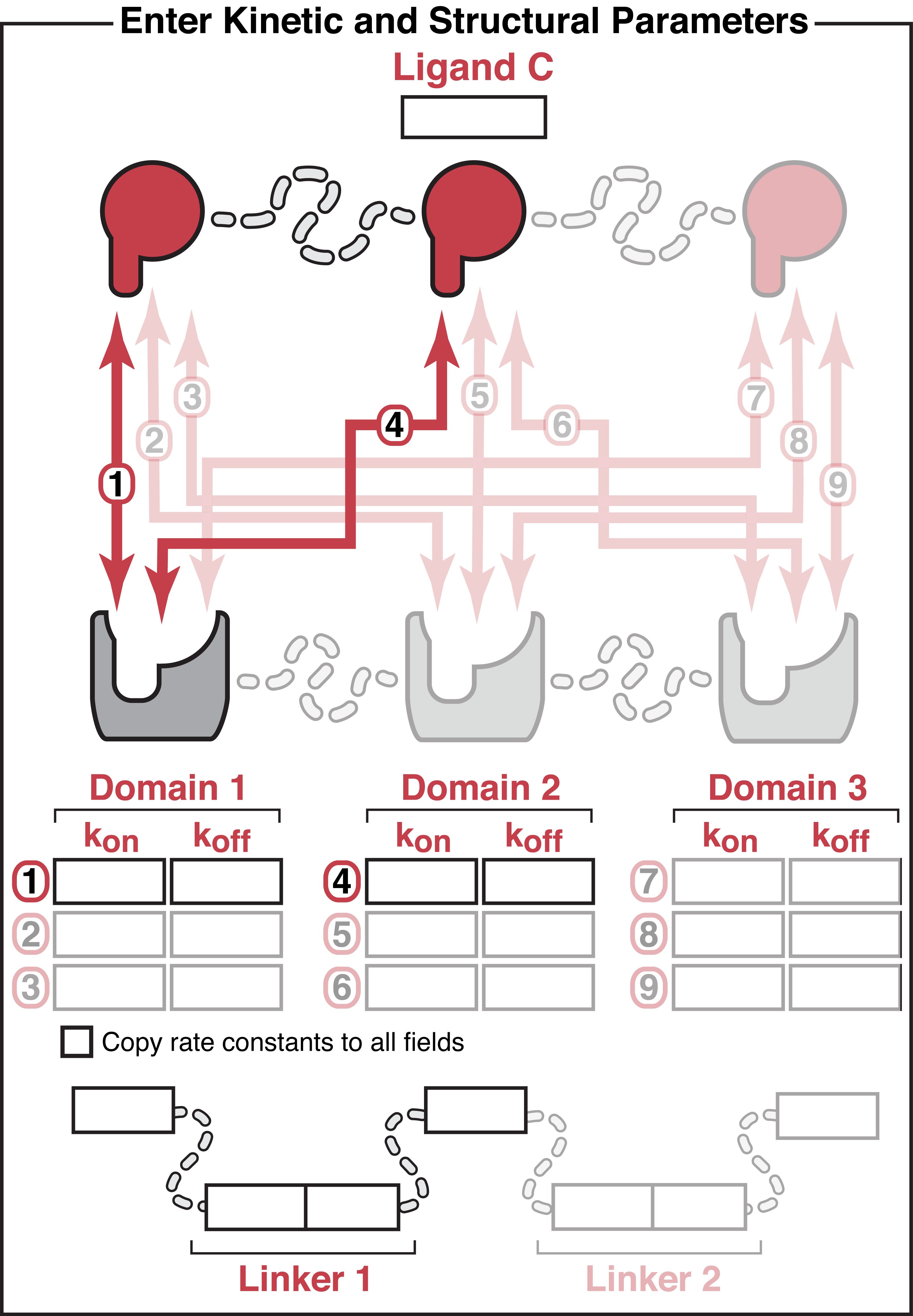

Supplement: Supplementary file 4 — Supplementary Software [file 41467_2022_32496_MOESM4_ESM.zip › Images/Connection_tab_images/MK_Tab2_C2x1.png]

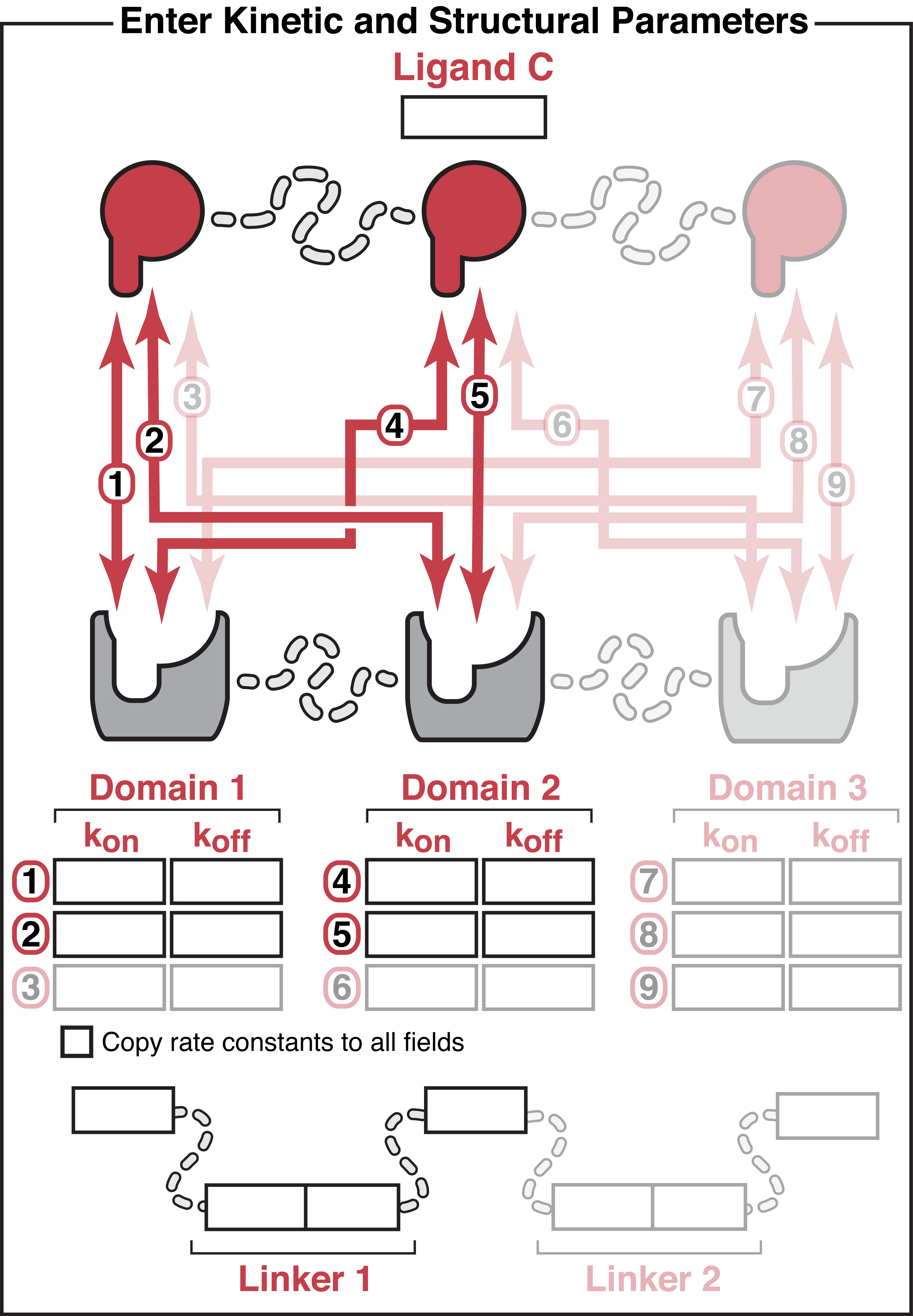

Supplement: Supplementary file 4 — Supplementary Software [file 41467_2022_32496_MOESM4_ESM.zip › Images/Connection_tab_images/MK_Tab2_C2x2.png]

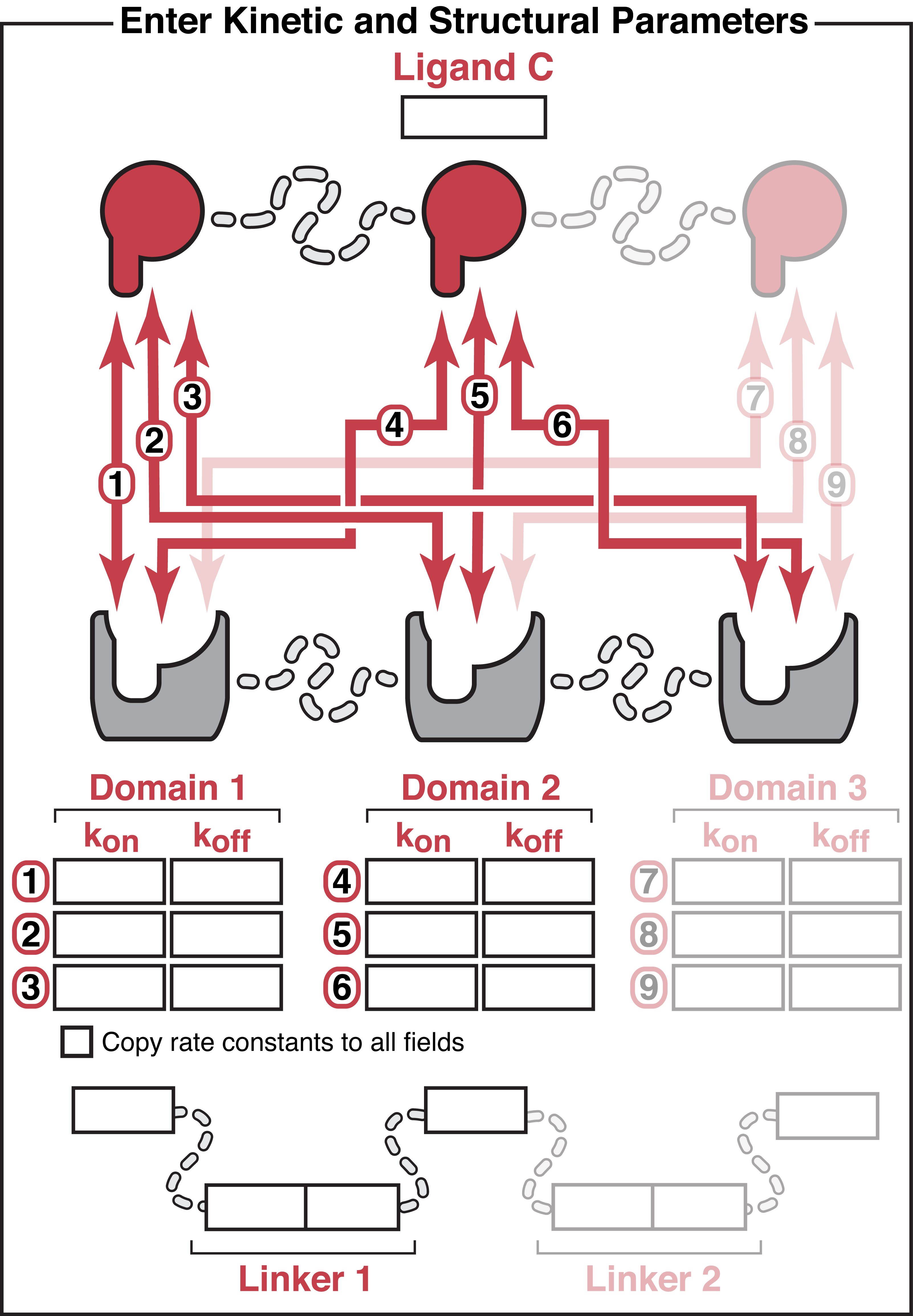

Supplement: Supplementary file 4 — Supplementary Software [file 41467_2022_32496_MOESM4_ESM.zip › Images/Connection_tab_images/MK_Tab2_C2x3.png]

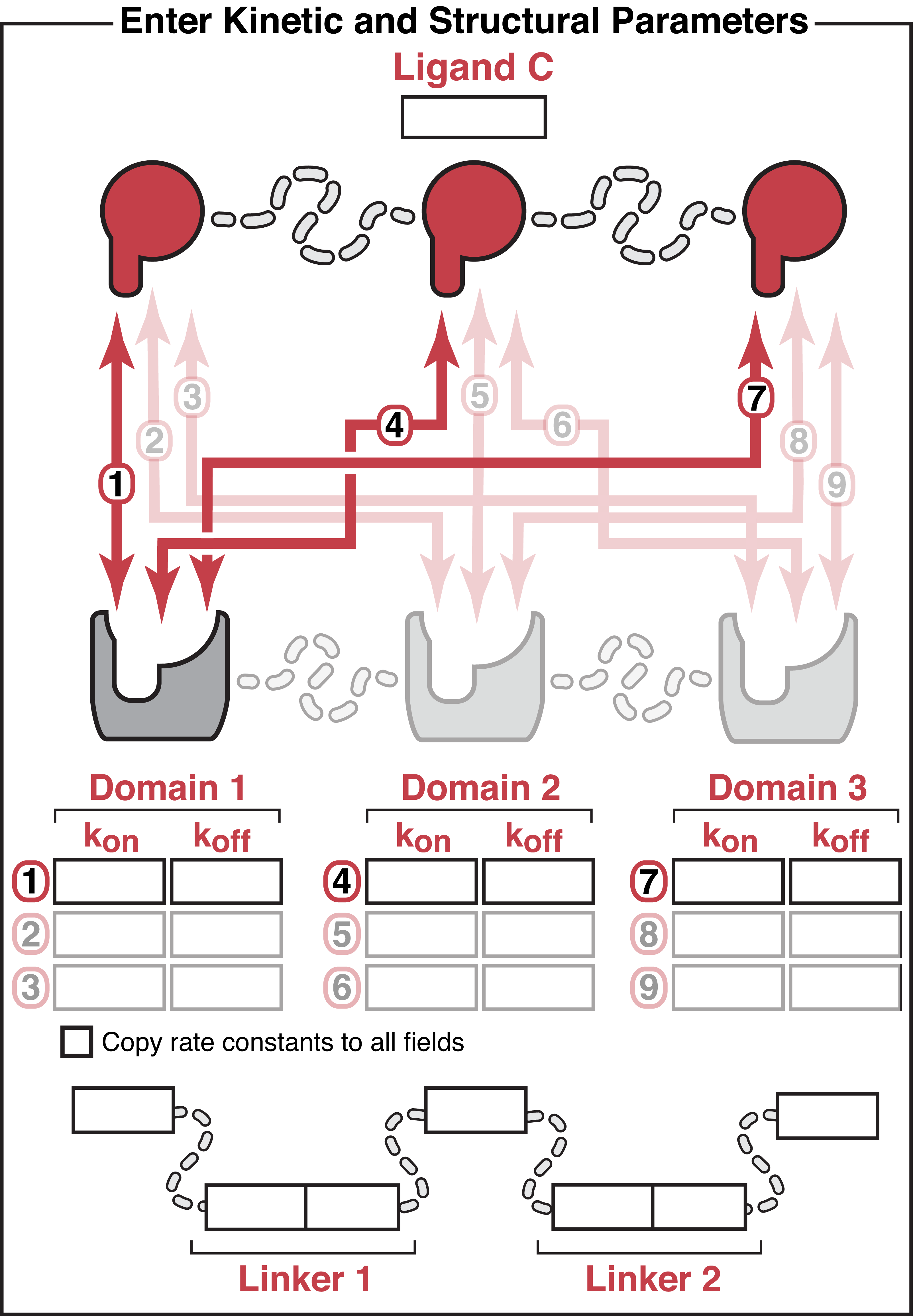

Supplement: Supplementary file 4 — Supplementary Software [file 41467_2022_32496_MOESM4_ESM.zip › Images/Connection_tab_images/MK_Tab2_C3x1.png]

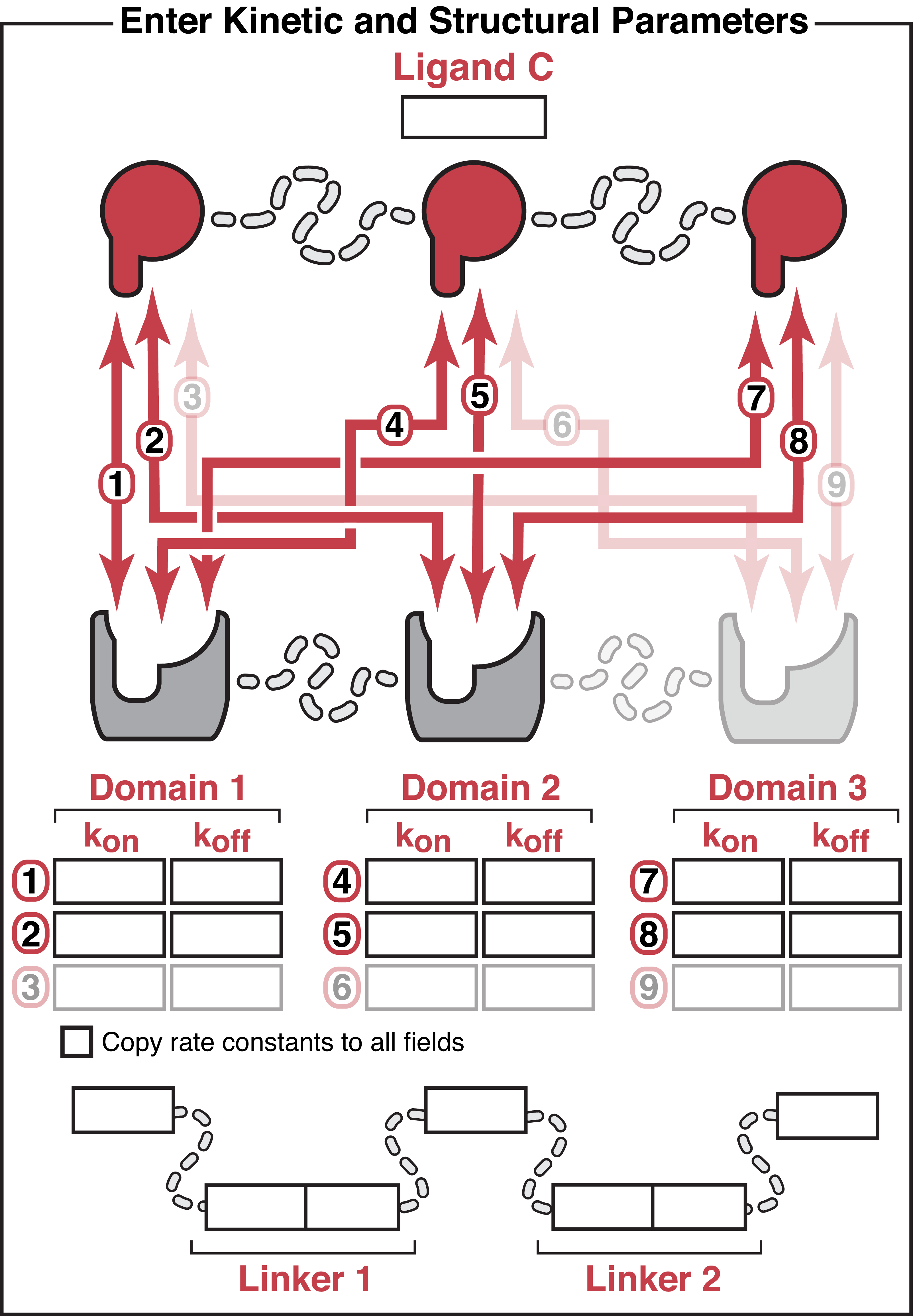

Supplement: Supplementary file 4 — Supplementary Software [file 41467_2022_32496_MOESM4_ESM.zip › Images/Connection_tab_images/MK_Tab2_C3x2.png]

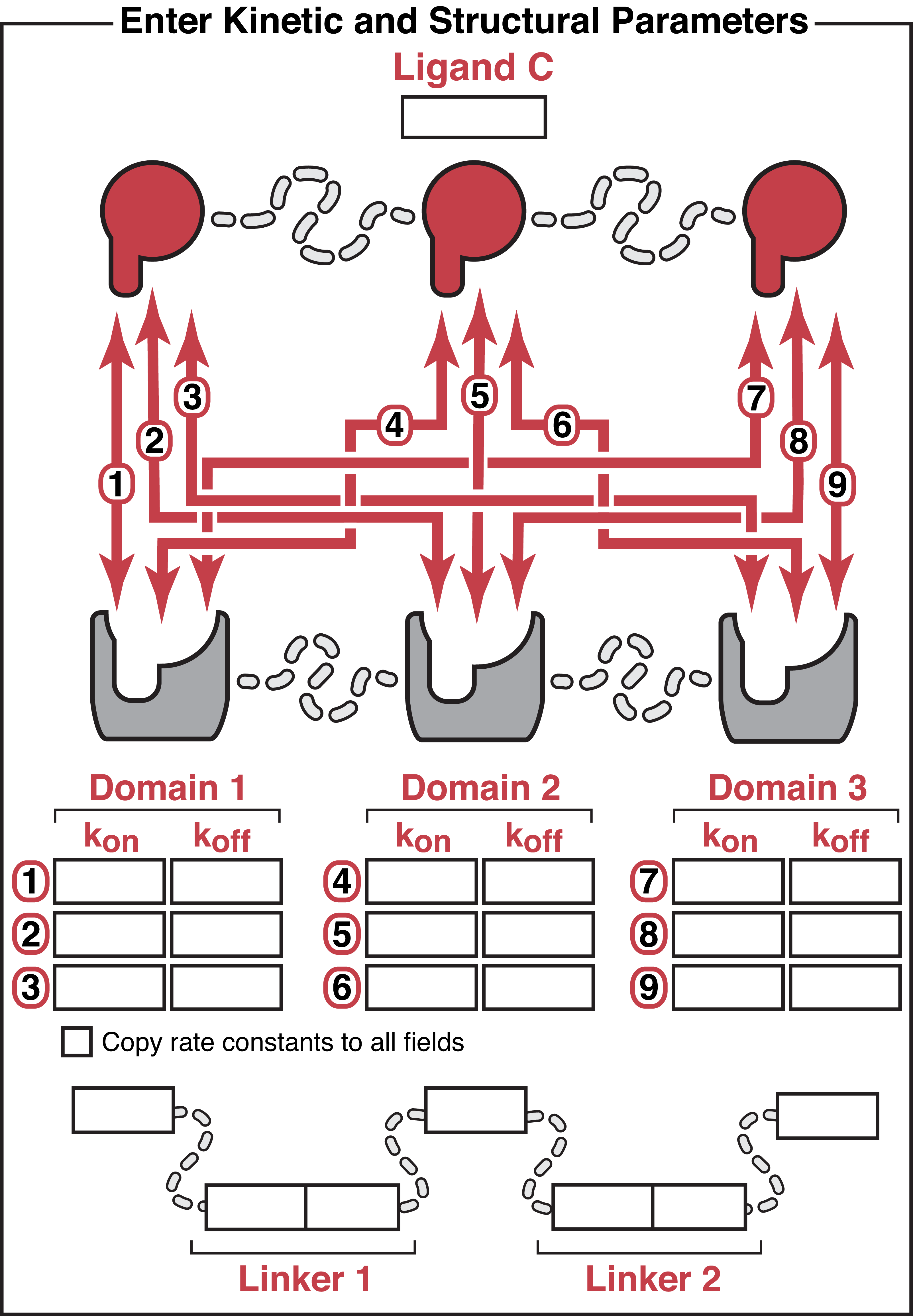

Supplement: Supplementary file 4 — Supplementary Software [file 41467_2022_32496_MOESM4_ESM.zip › Images/Connection_tab_images/MK_Tab2_C3x3.png]

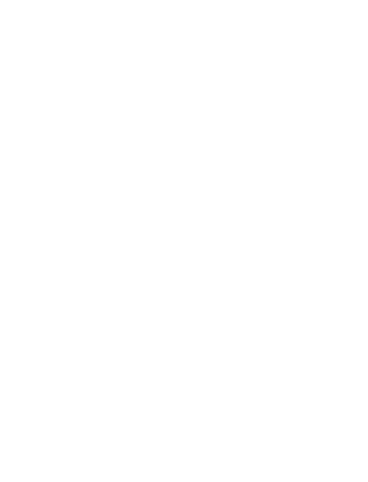

Supplement: Supplementary file 4 — Supplementary Software [file 41467_2022_32496_MOESM4_ESM.zip › Images/Empty.png]

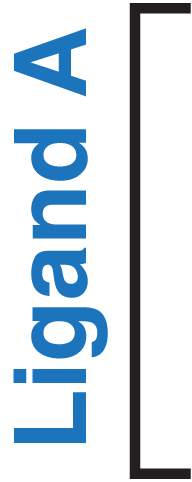

Supplement: Supplementary file 4 — Supplementary Software [file 41467_2022_32496_MOESM4_ESM.zip › Images/Ligand_A_0.png]

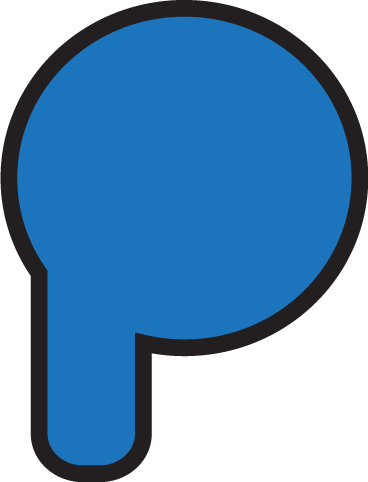

Supplement: Supplementary file 4 — Supplementary Software [file 41467_2022_32496_MOESM4_ESM.zip › Images/Ligand_A_1.png]

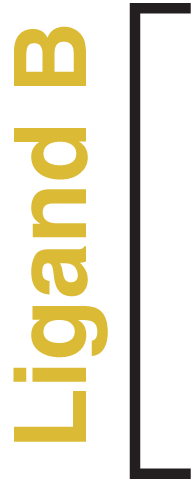

Supplement: Supplementary file 4 — Supplementary Software [file 41467_2022_32496_MOESM4_ESM.zip › Images/Ligand_B_0.png]

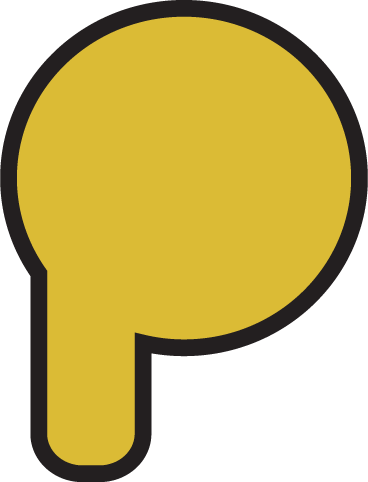

Supplement: Supplementary file 4 — Supplementary Software [file 41467_2022_32496_MOESM4_ESM.zip › Images/Ligand_B_1.png]

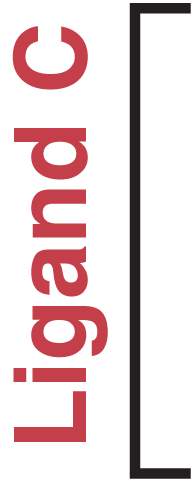

Supplement: Supplementary file 4 — Supplementary Software [file 41467_2022_32496_MOESM4_ESM.zip › Images/Ligand_C_0.png]

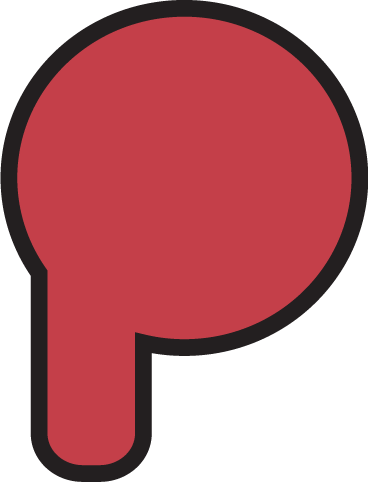

Supplement: Supplementary file 4 — Supplementary Software [file 41467_2022_32496_MOESM4_ESM.zip › Images/Ligand_C_1.png]

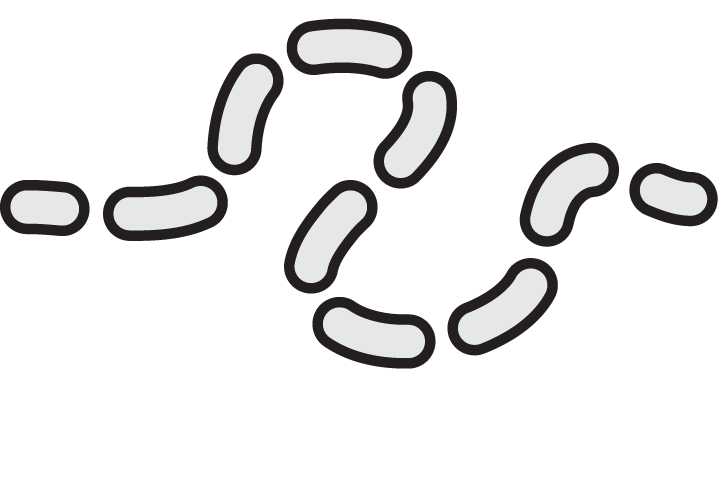

Supplement: Supplementary file 4 — Supplementary Software [file 41467_2022_32496_MOESM4_ESM.zip › Images/Linker_ligand.png]

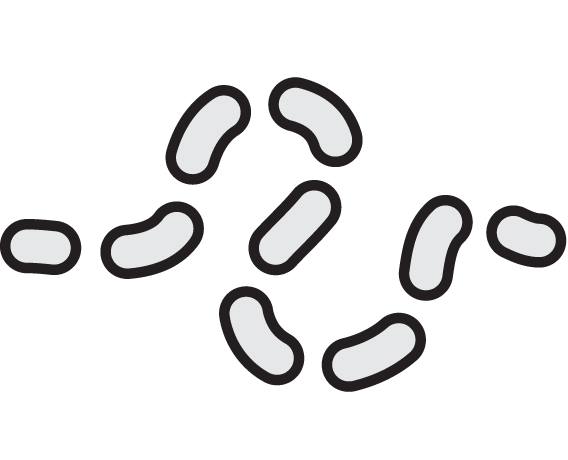

Supplement: Supplementary file 4 — Supplementary Software [file 41467_2022_32496_MOESM4_ESM.zip › Images/Linker_receptor.png]

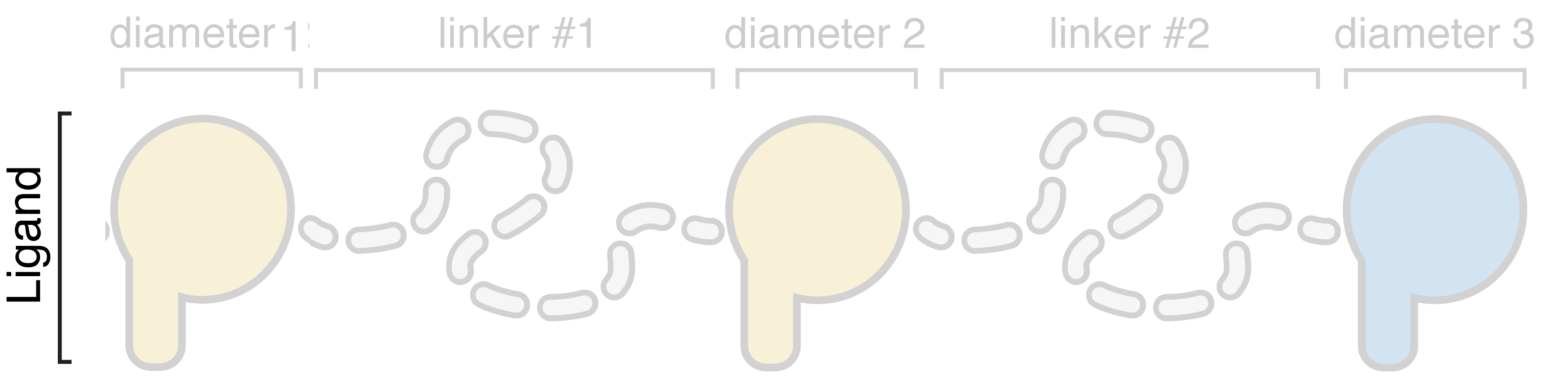

Supplement: Supplementary file 4 — Supplementary Software [file 41467_2022_32496_MOESM4_ESM.zip › Images/Pilot/MV_Lig0.png]

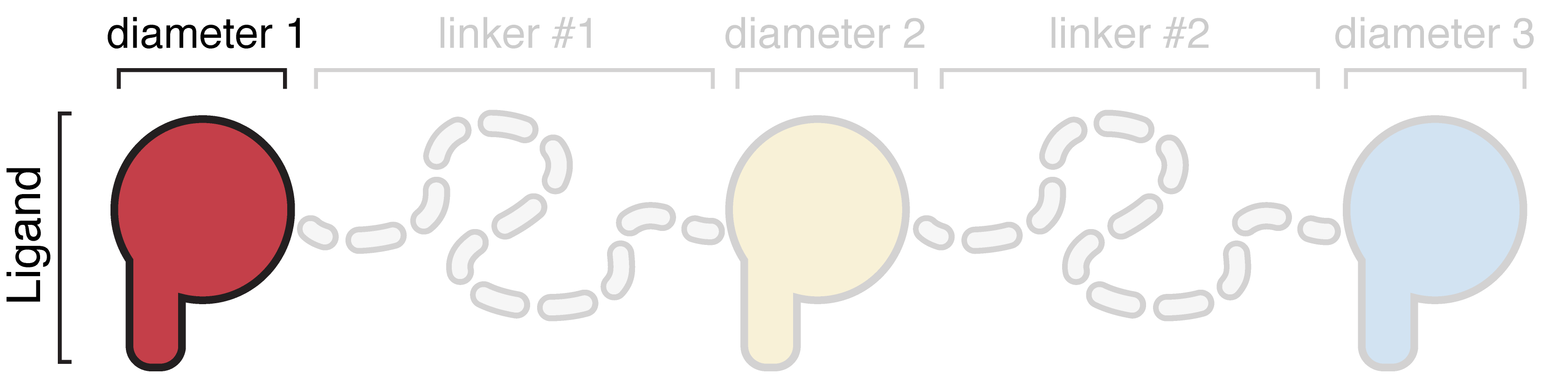

Supplement: Supplementary file 4 — Supplementary Software [file 41467_2022_32496_MOESM4_ESM.zip › Images/Pilot/MV_Lig1.png]

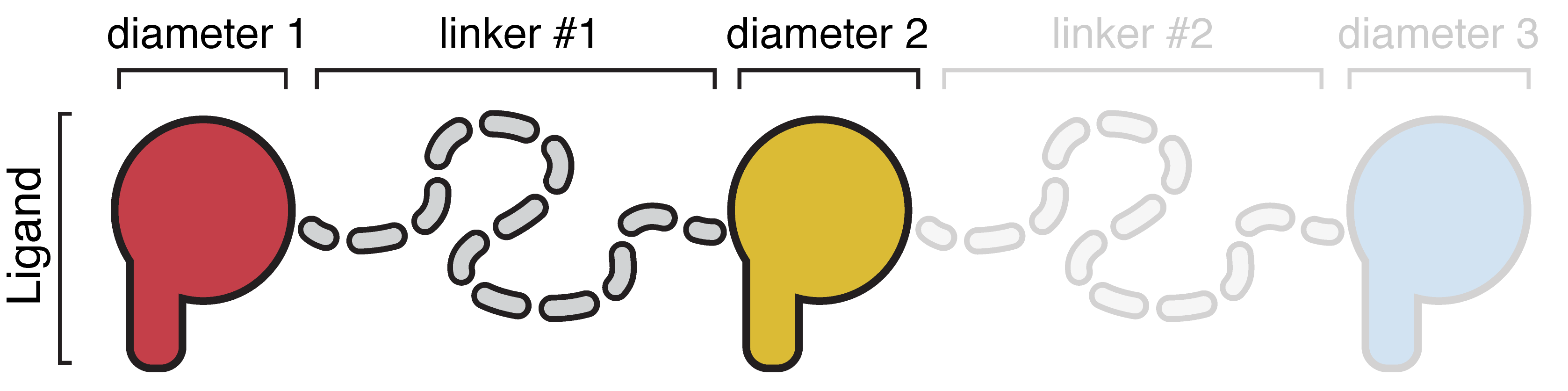

Supplement: Supplementary file 4 — Supplementary Software [file 41467_2022_32496_MOESM4_ESM.zip › Images/Pilot/MV_Lig2.png]

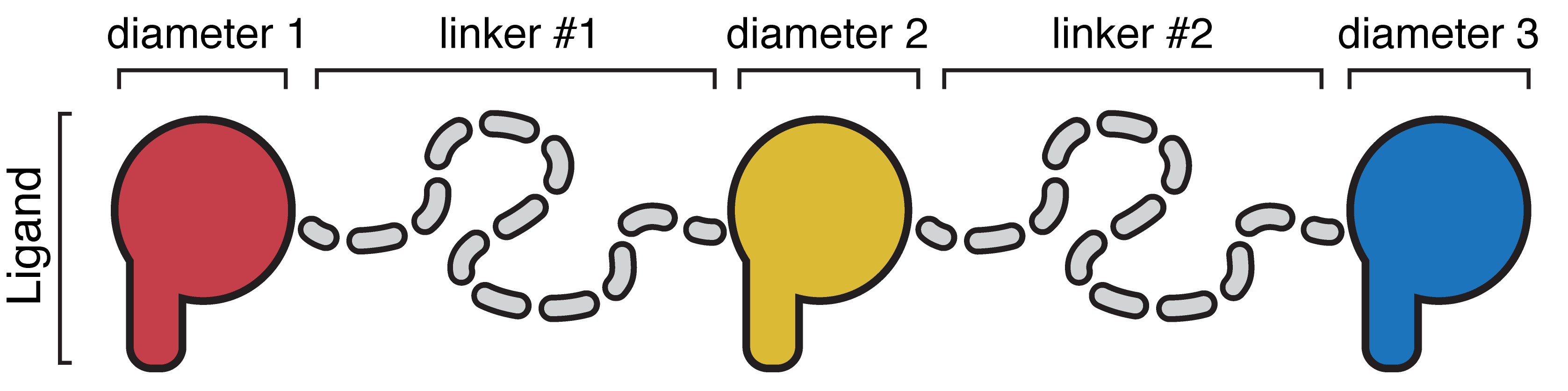

Supplement: Supplementary file 4 — Supplementary Software [file 41467_2022_32496_MOESM4_ESM.zip › Images/Pilot/MV_Lig3.png]

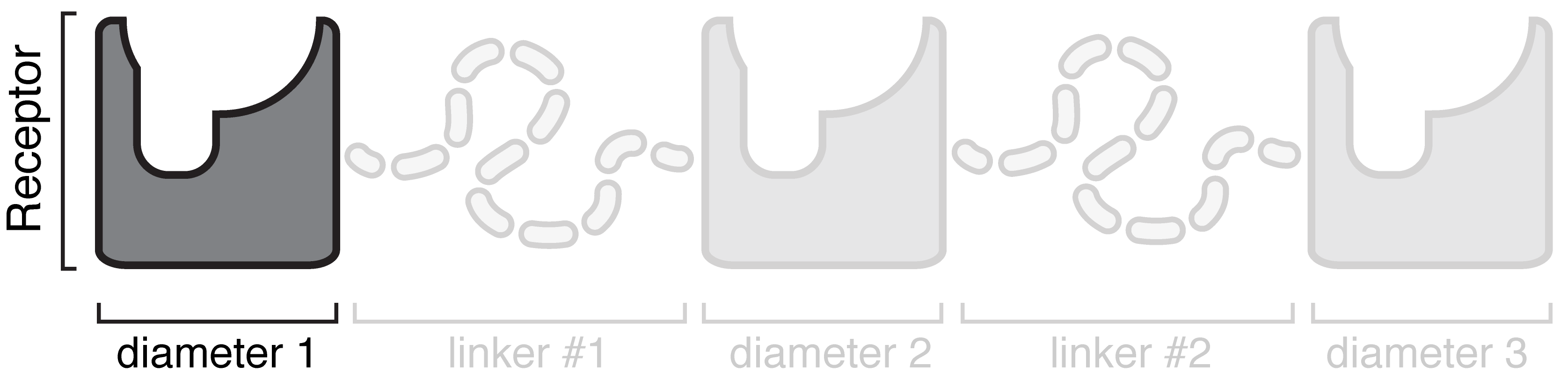

Supplement: Supplementary file 4 — Supplementary Software [file 41467_2022_32496_MOESM4_ESM.zip › Images/Pilot/MV_Rec1.png]

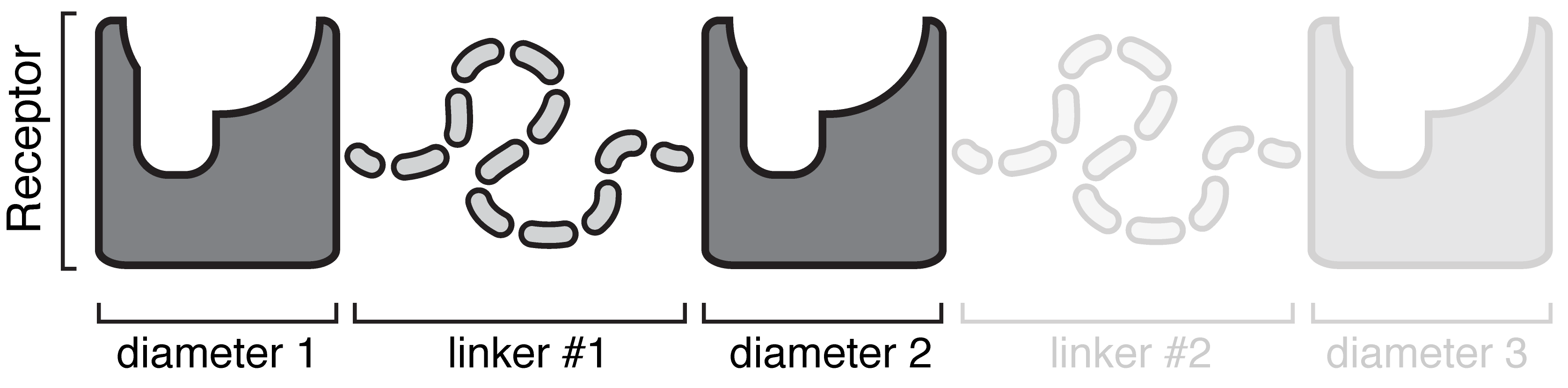

Supplement: Supplementary file 4 — Supplementary Software [file 41467_2022_32496_MOESM4_ESM.zip › Images/Pilot/MV_Rec2.png]

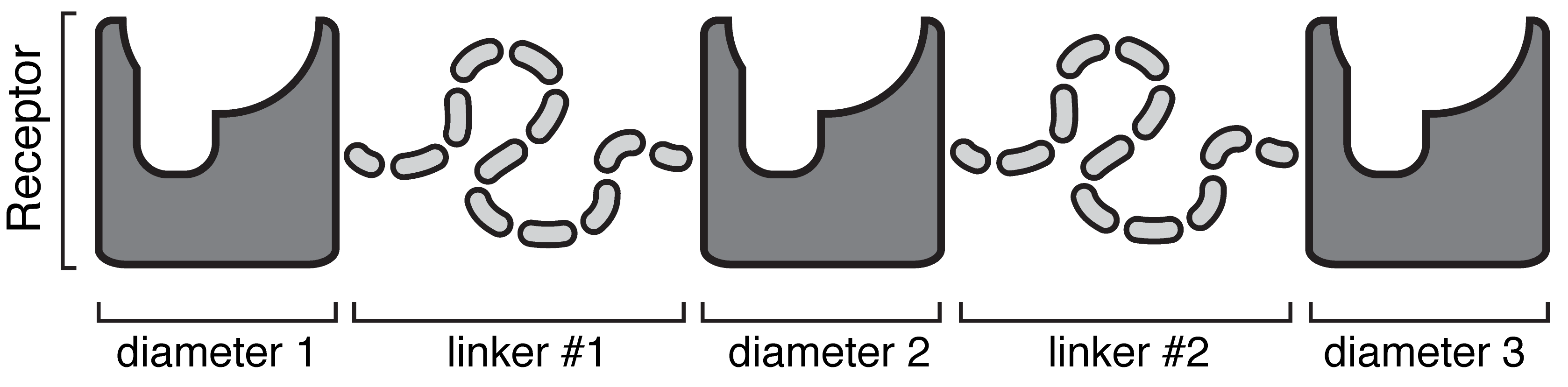

Supplement: Supplementary file 4 — Supplementary Software [file 41467_2022_32496_MOESM4_ESM.zip › Images/Pilot/MV_Rec3.png]

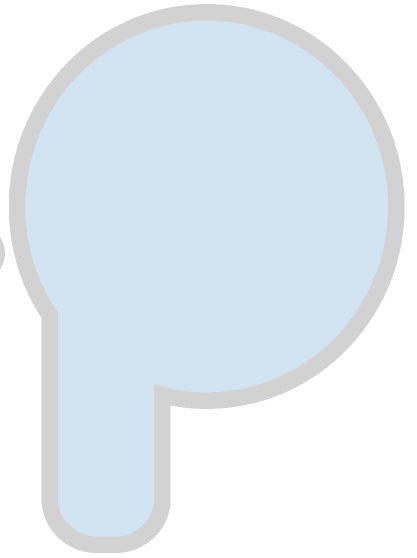

Supplement: Supplementary file 4 — Supplementary Software [file 41467_2022_32496_MOESM4_ESM.zip › Images/Pilot/Single_Ligand_empty.png]

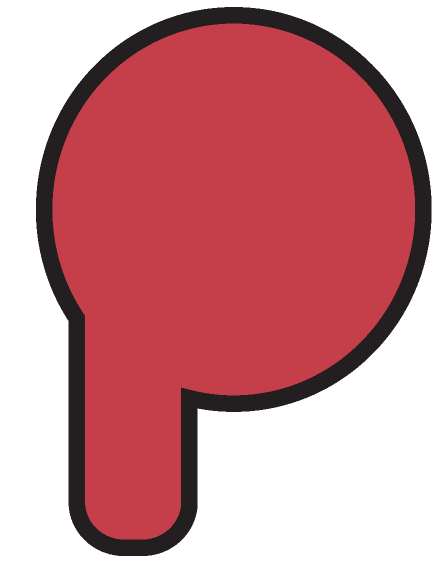

Supplement: Supplementary file 4 — Supplementary Software [file 41467_2022_32496_MOESM4_ESM.zip › Images/Pilot/Single_Ligand_filled.png]

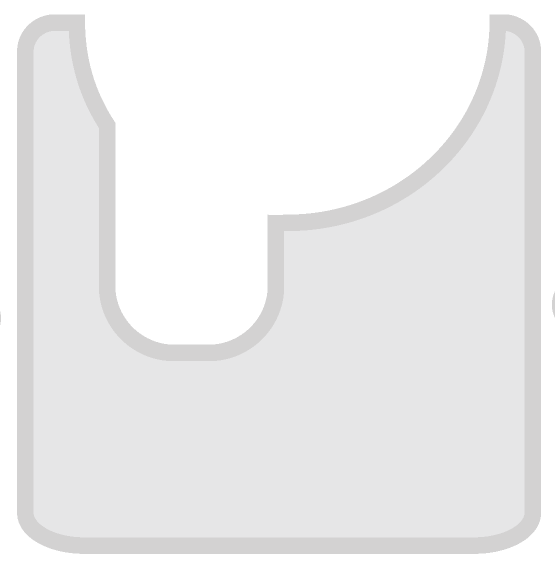

Supplement: Supplementary file 4 — Supplementary Software [file 41467_2022_32496_MOESM4_ESM.zip › Images/Pilot/Single_Receptor_empty.png]

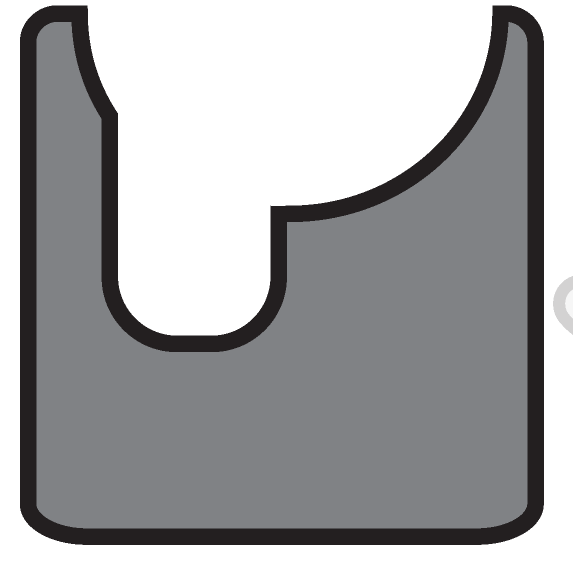

Supplement: Supplementary file 4 — Supplementary Software [file 41467_2022_32496_MOESM4_ESM.zip › Images/Pilot/Single_Receptor_filled.png]

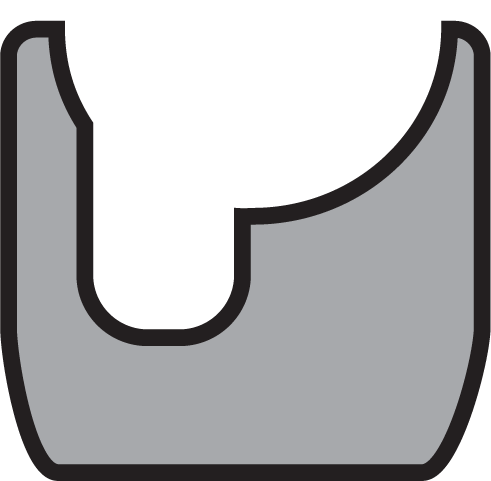

Supplement: Supplementary file 4 — Supplementary Software [file 41467_2022_32496_MOESM4_ESM.zip › Images/Receptor.png]

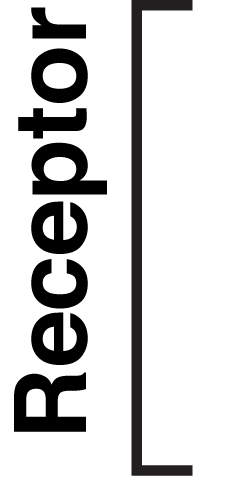

Supplement: Supplementary file 4 — Supplementary Software [file 41467_2022_32496_MOESM4_ESM.zip › Images/Receptor_0.png]

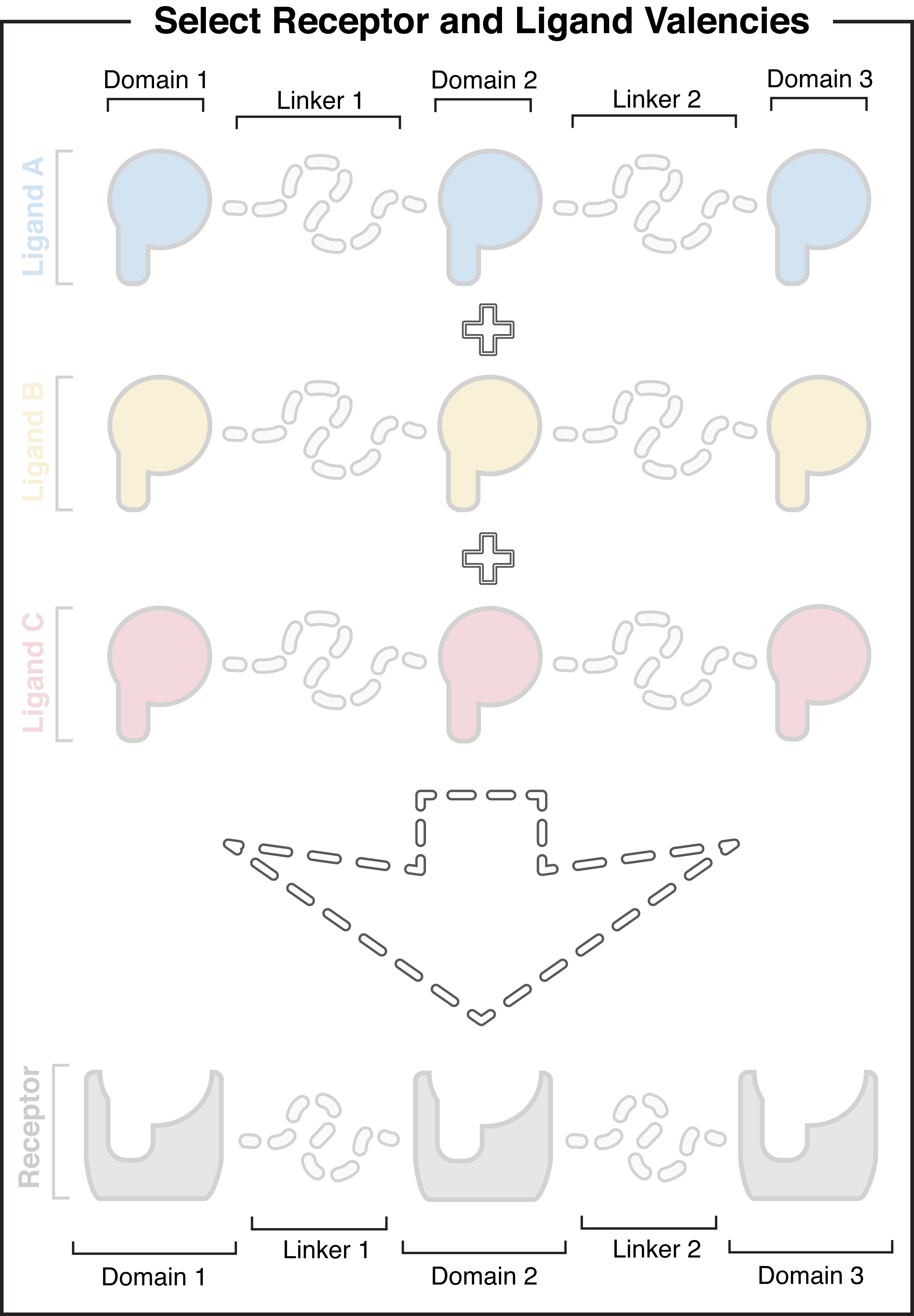

Supplement: Supplementary file 4 — Supplementary Software [file 41467_2022_32496_MOESM4_ESM.zip › Images/Tab1_background.png]
