# Supplementary material for: MVsim is a toolset for quantifying and designing multivalent interactions
Source: Nat Commun. 2022 Sep 6;13:5029. doi: 10.1038/s41467-022-32496-6 (PMC9448752; doi:10.1038/s41467-022-32496-6)

Simulating Multivalent Biomolecular Interactions

Tutorial 1: Interacting with the MVsim graphical user interface

Bruncsics et al. (2022)

1. Designing a multivalent interaction system in the Topology tab

a. MVsim initiates with a bivalent interaction demo preset

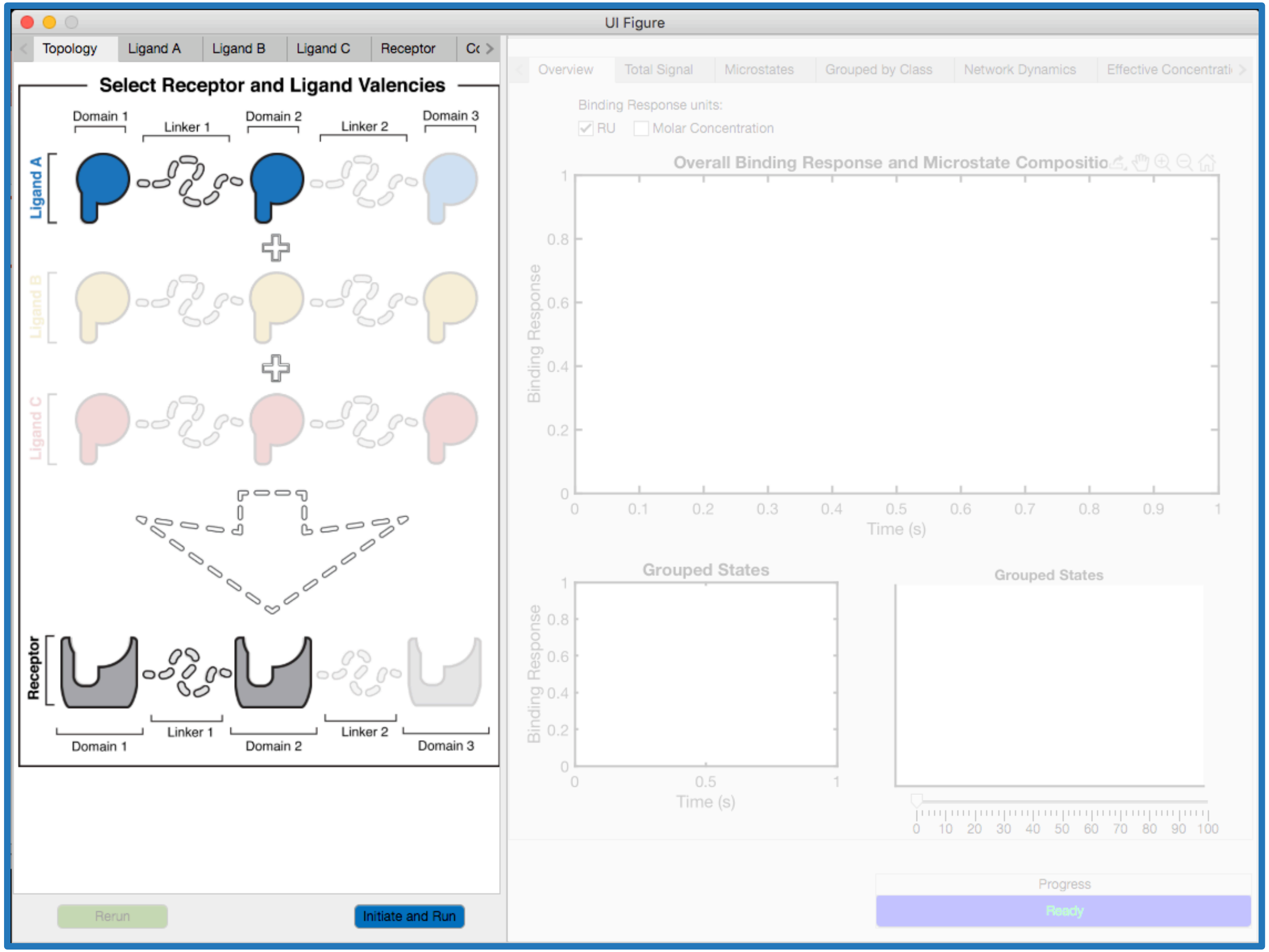

1. Designing a multivalent interaction system in the Topology tab

b. A bivalent Ligand A and bivalent Receptor are indicated in the Topology tab

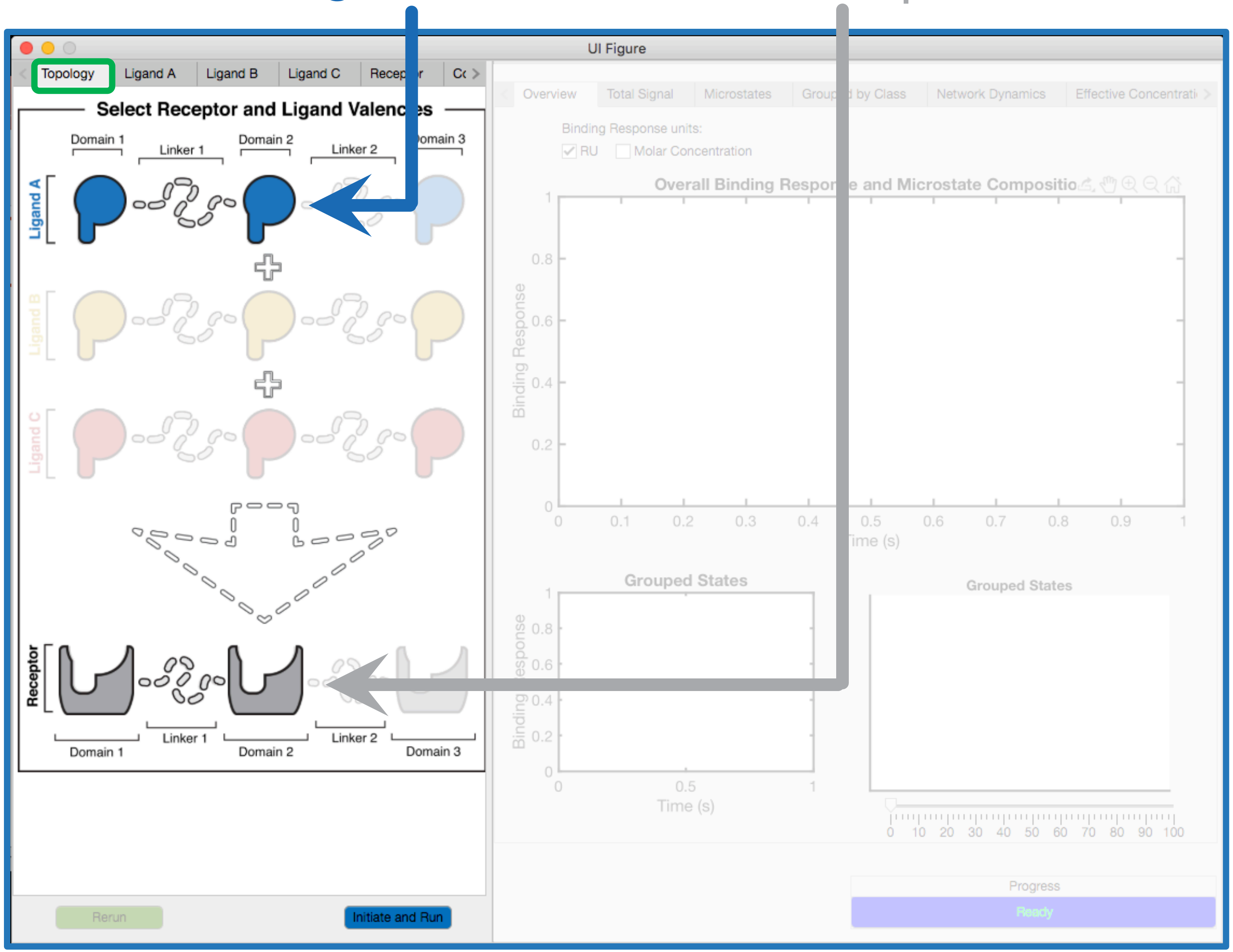

1. Designing a multivalent interaction system in the Topology tab

c. Clicking the binding domain icons will toggle between mono, bi, and trivalent

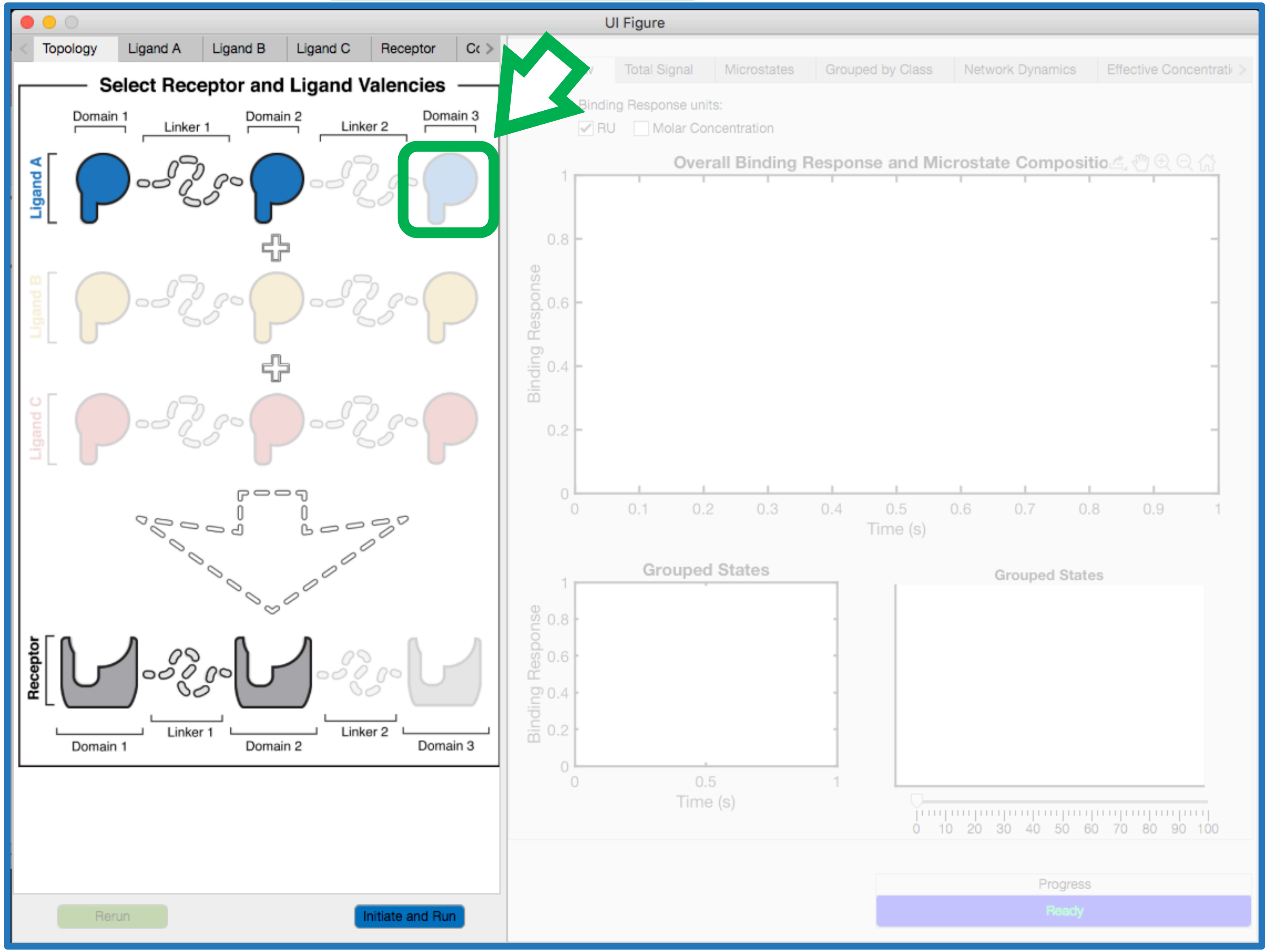

1. Designing a multivalent interaction system in the Topology tab

c. Clicking the binding domain icons will toggle between mono, bi, and trivalent

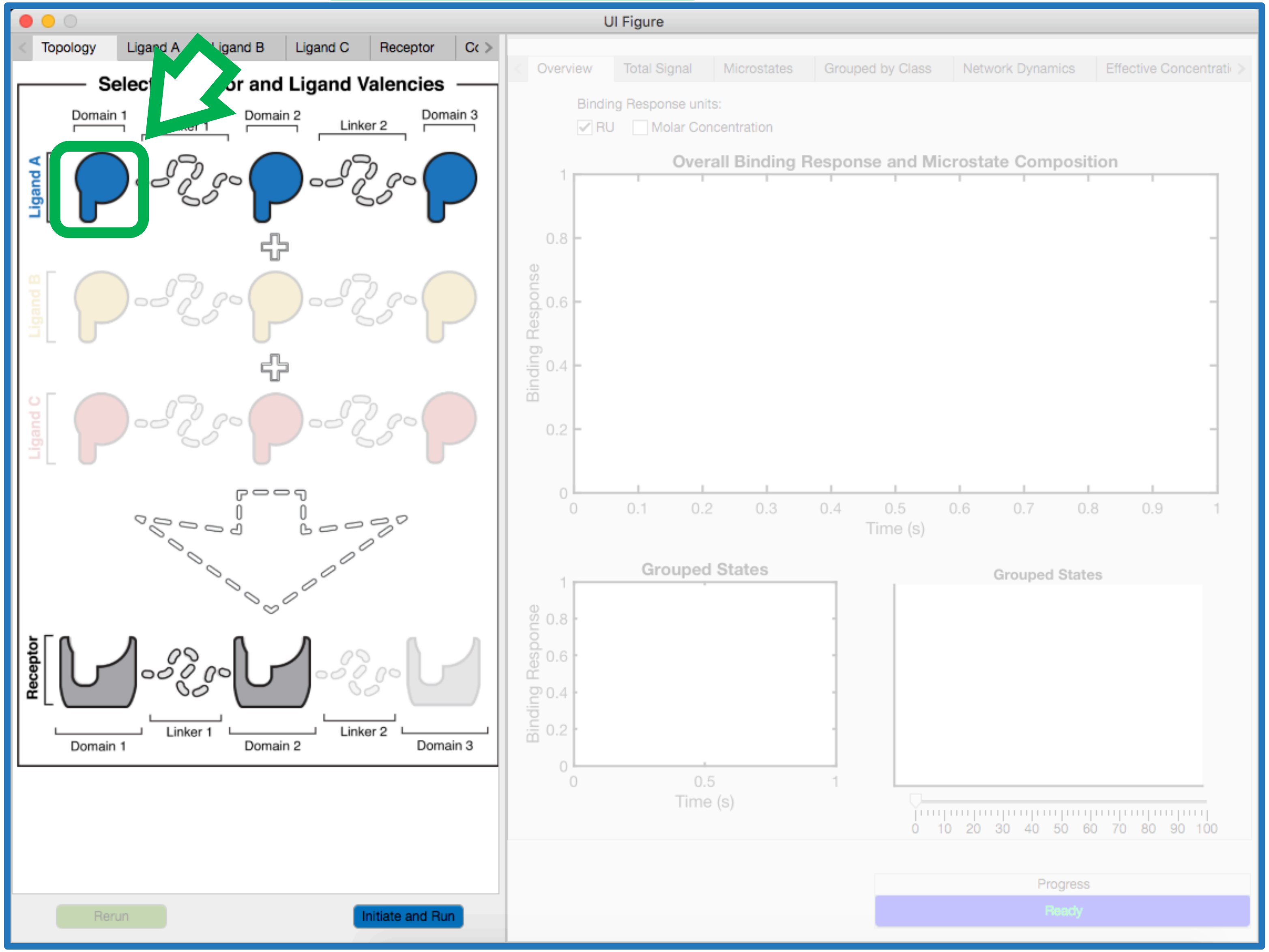

d. Clicking a monovalent ligand a second time will remove it from the simulation

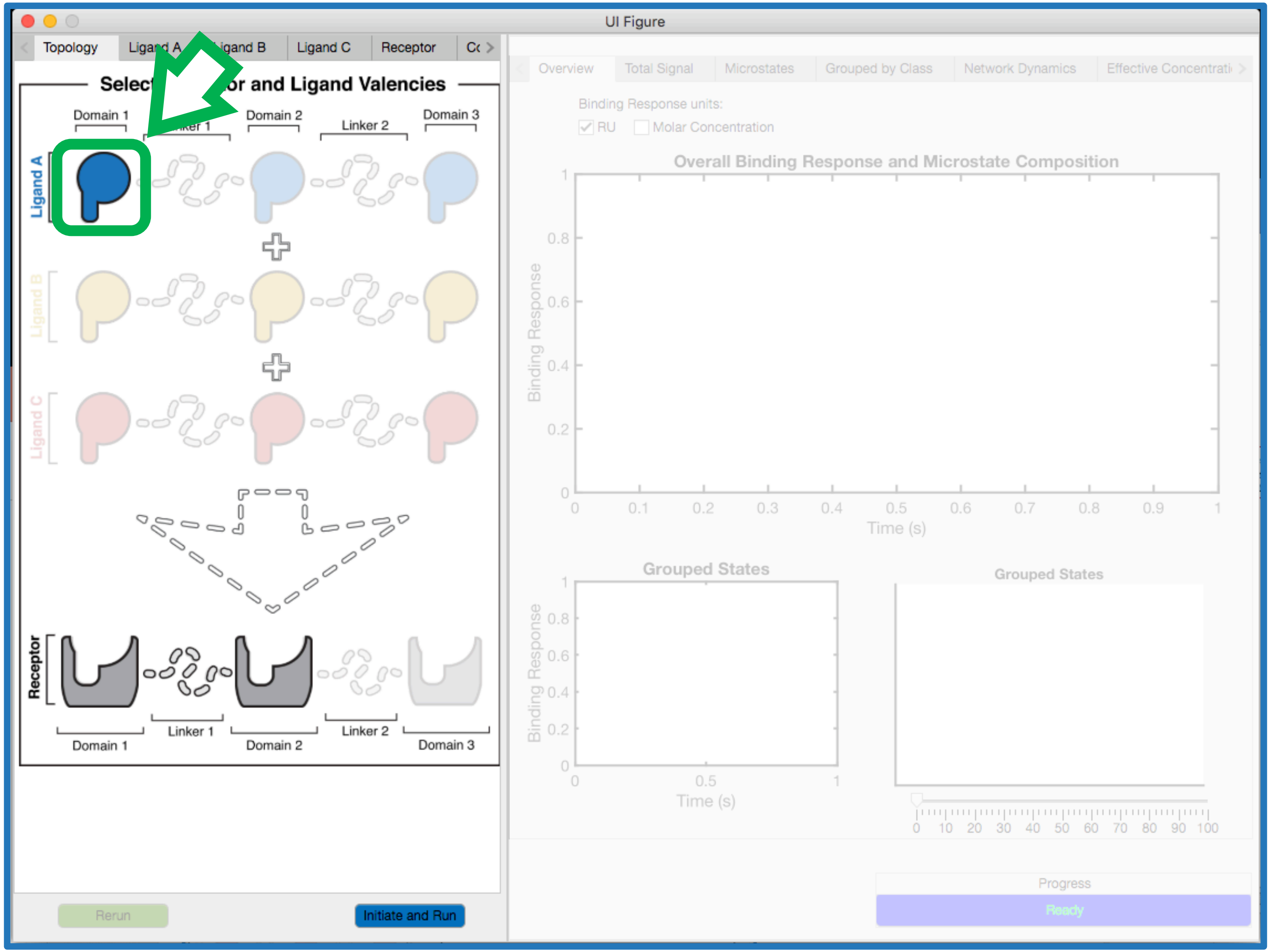

d. Clicking a monovalent ligand a second time will remove it from the simulation

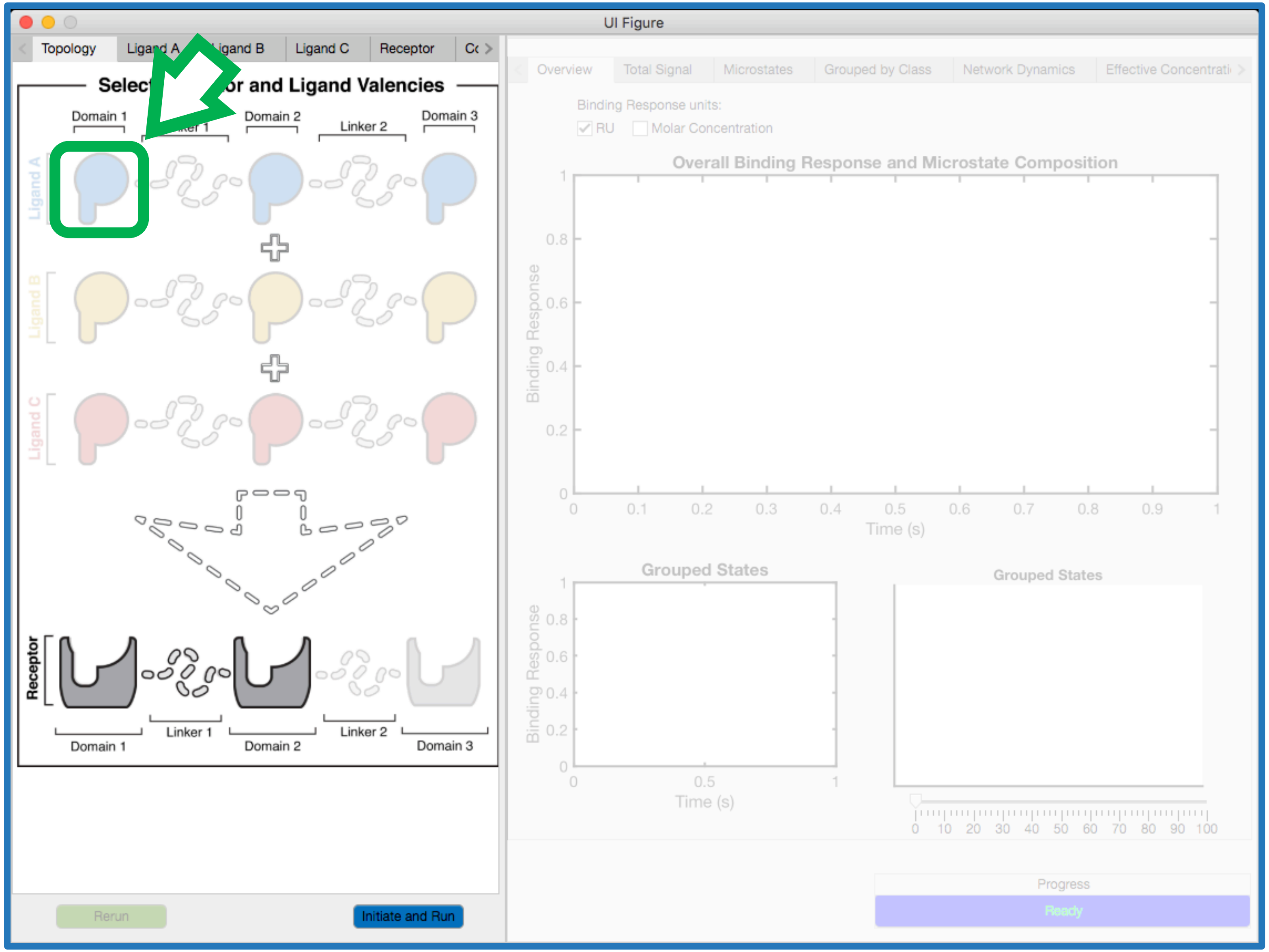

e. Ligand B and Ligand C are similarly added, removed, and their valencies selected

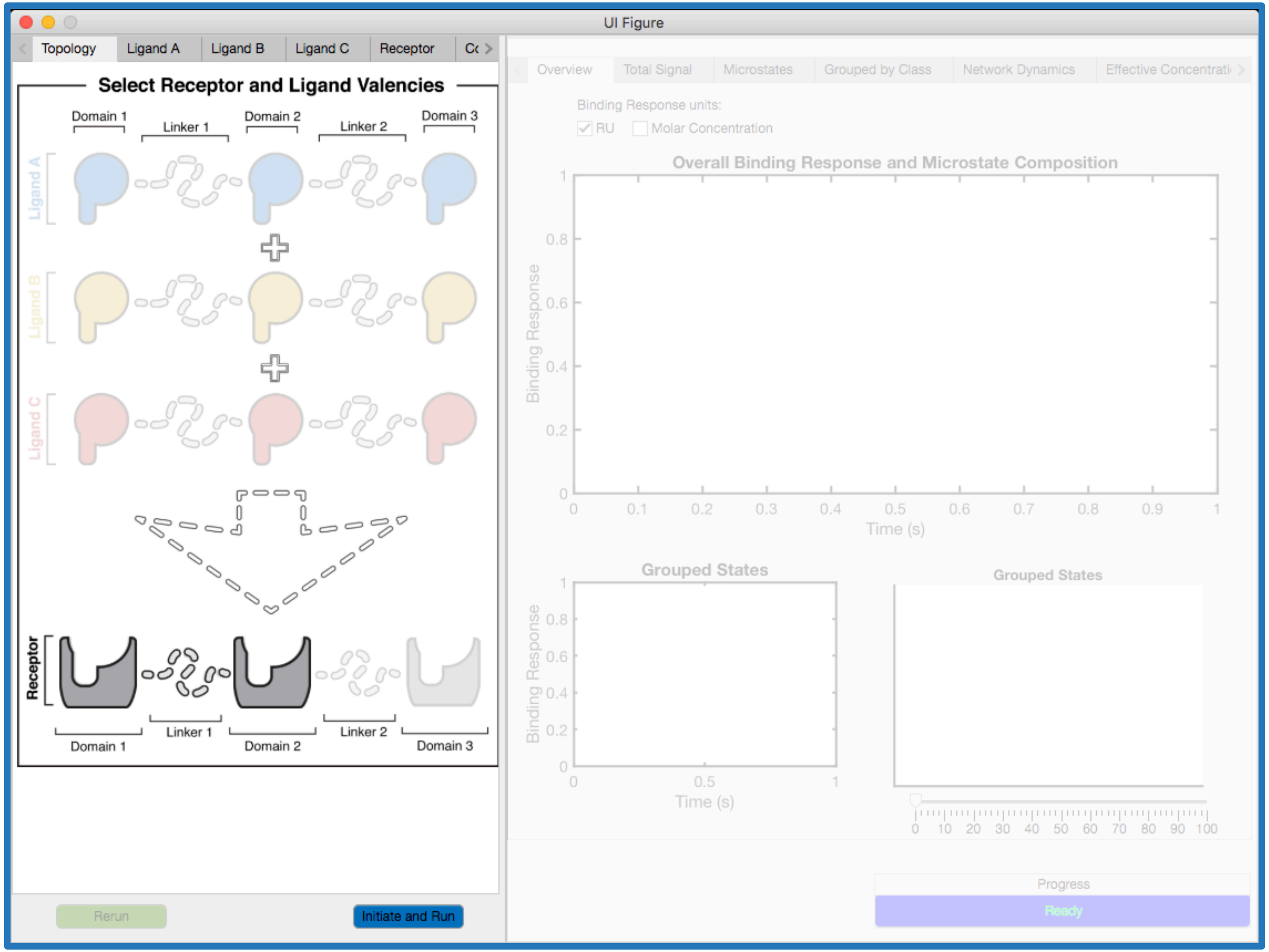

e. Ligand B and Ligand C are similarly added, removed, and their valencies selected

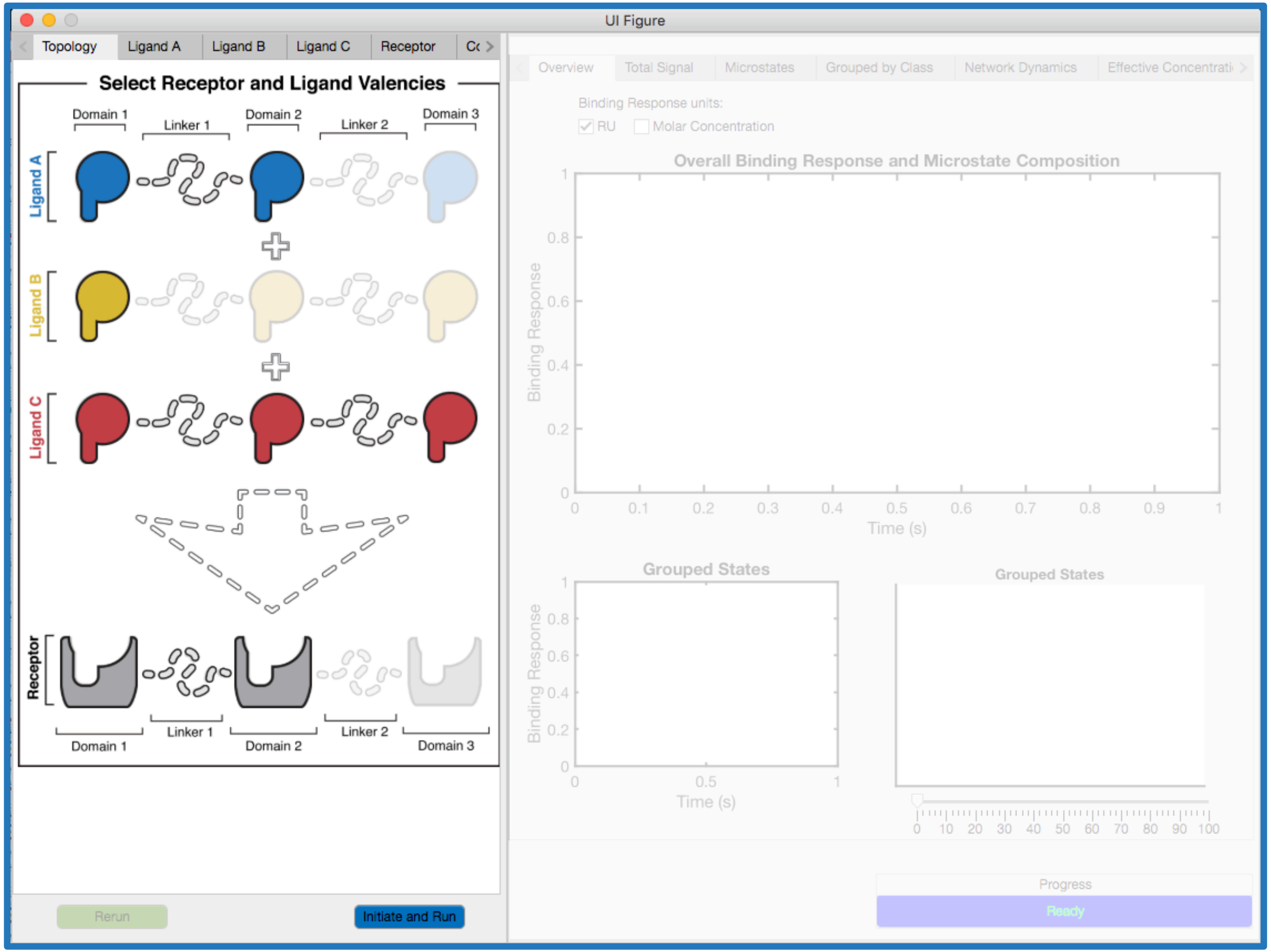

f. The Receptor is permanently active and toggled between mono, bi, and trivalency

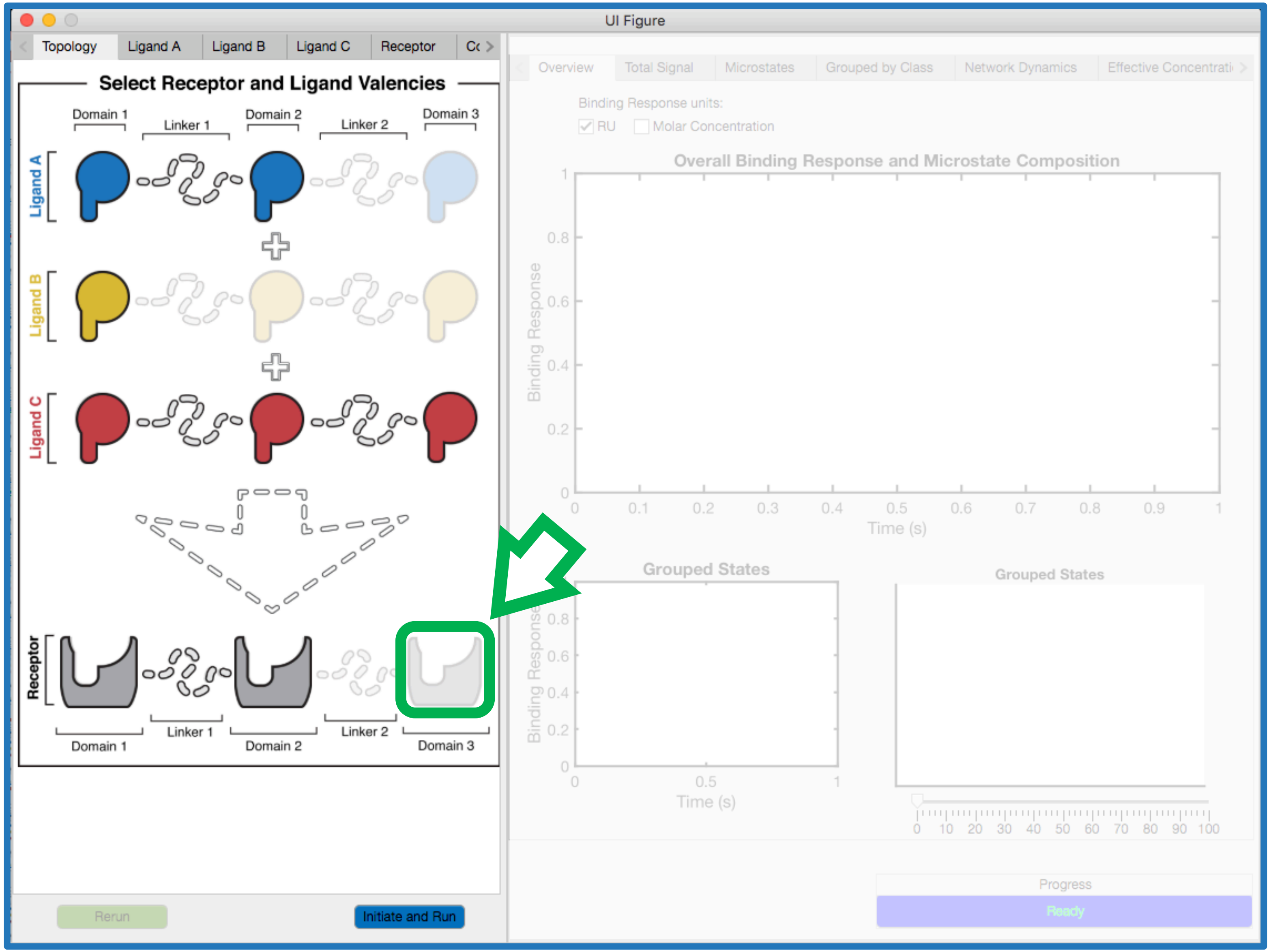

1. Designing a multivalent interaction system in the Topology tab

f. The Receptor is permanently active and toggled between mono, bi, and trivalency

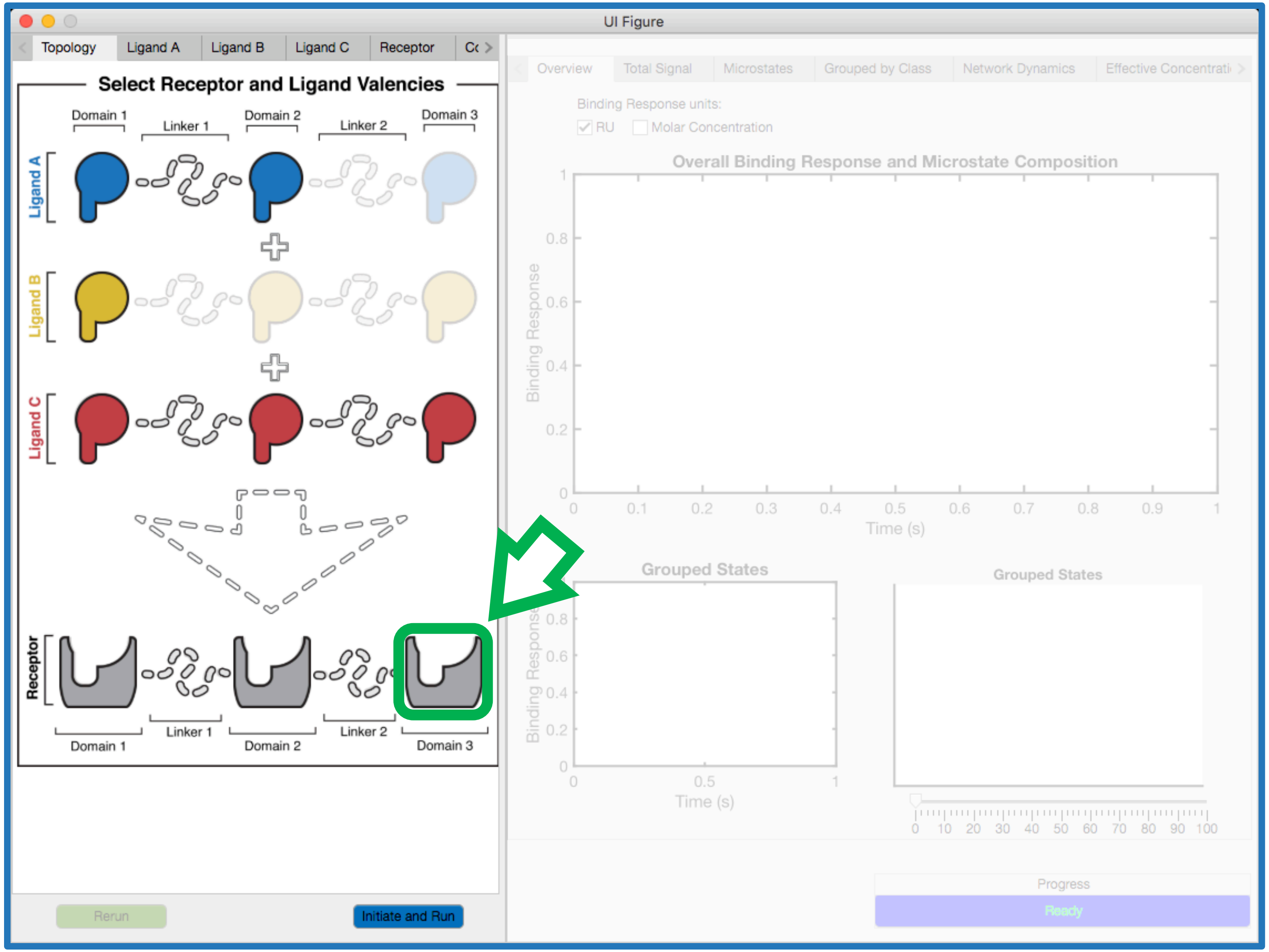

2. Parameterizing the multivalent system in the Ligand tabs

a. For a given topology the Ligand tabs enable input of kinetic and structural parameters

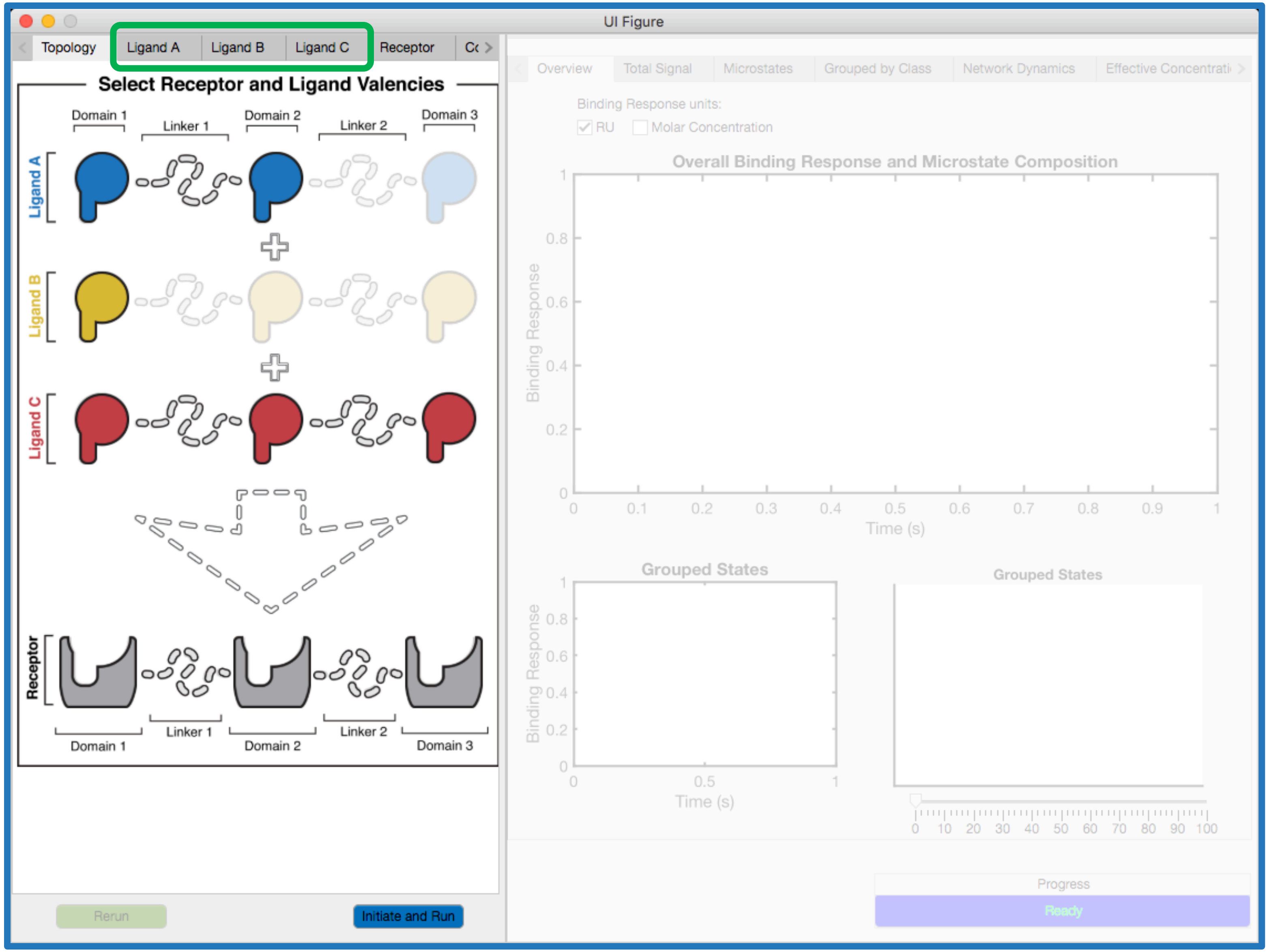

b. User input parameterization of Ligand A

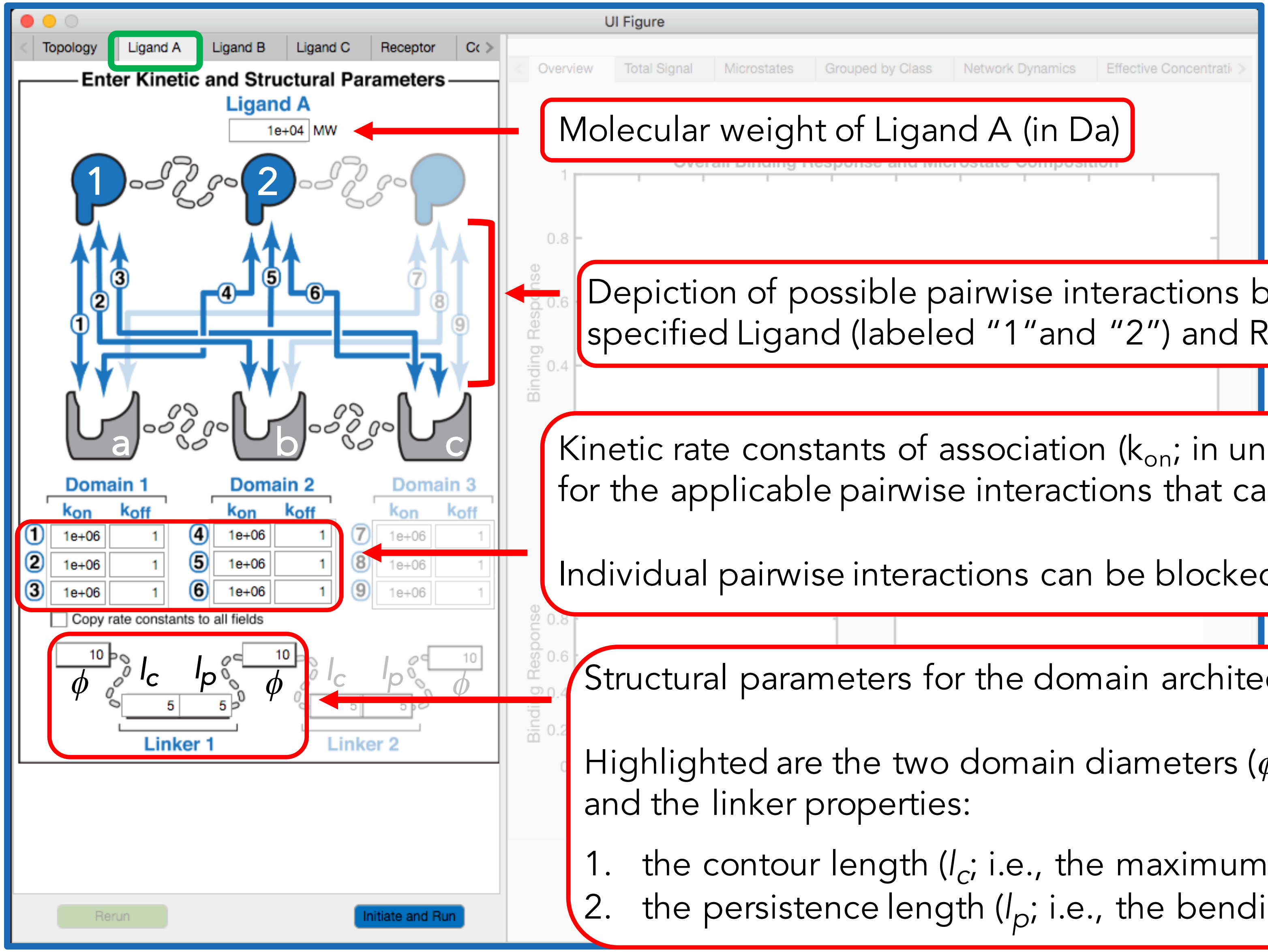

Molecular weight of Ligand A (in Da)

Depiction of possible pairwise interactions between the binding domains of the specified Ligand (labeled "1"and "2") and Receptor (labeled "a", "b", and "c")

Kinetic rate constants of association (k_{on}; in units of $M^{-1}s^{-1}$) and dissociation (k_{off}; in units of s^{-1}) for the applicable pairwise interactions that can occur between Ligand A and the Receptor

Individual pairwise interactions can be blocked by entering "0" for the respective k_{on} and k_{off}

Structural parameters for the domain architecture of a bivalent Ligand A.

Highlighted are the two domain diameters (ϕ, units of Angstrom) and the linker properties:

1. the contour length (l_c; i.e., the maximum end-to-end distance; units of Angstrom)
2. the persistence length (l_p; i.e., the bending stiffness; units of Angstrom)

c. User input parameterization of Ligand B

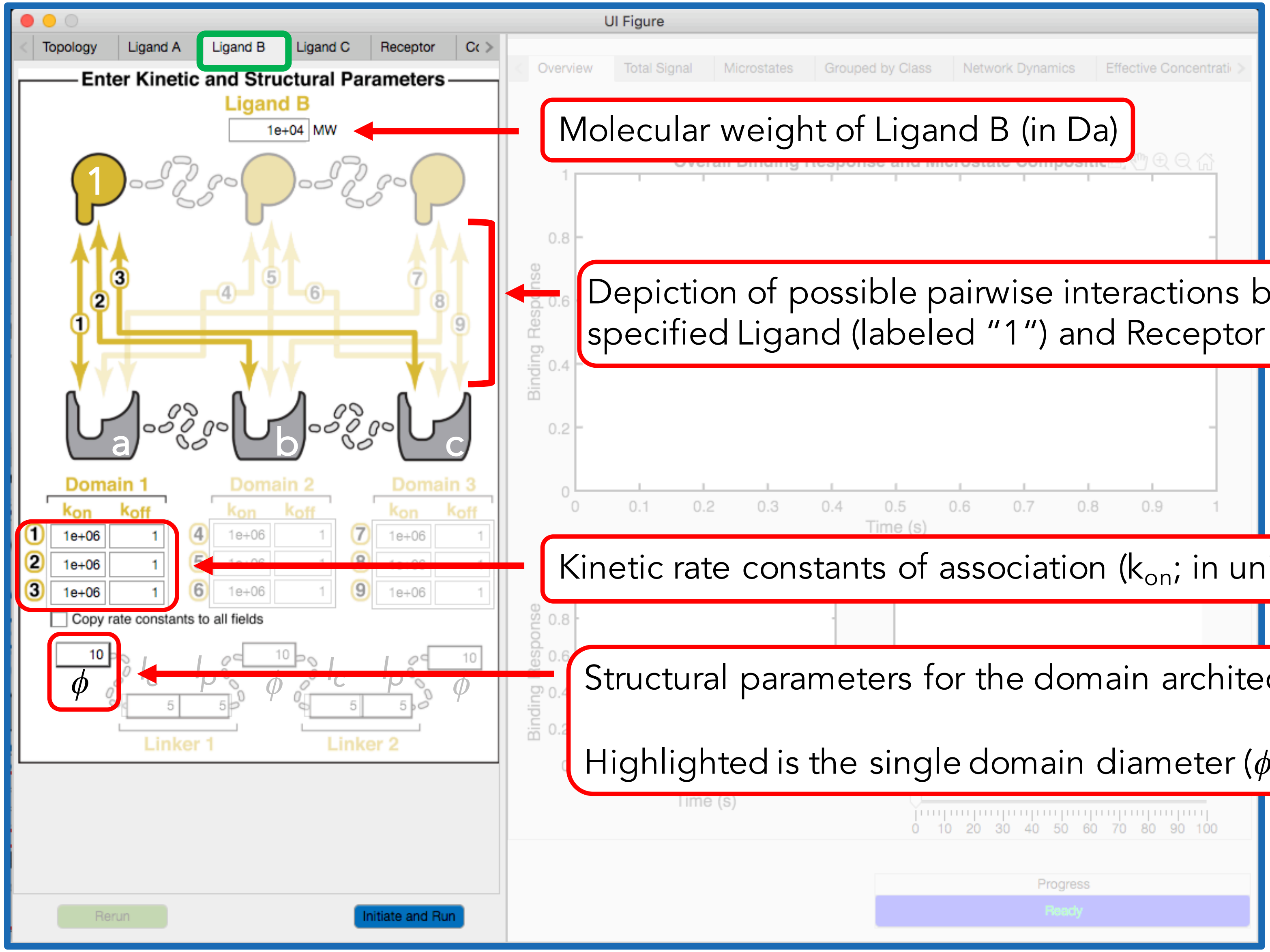

Molecular weight of Ligand B (in Da)

Depiction of possible pairwise interactions between the binding domains of the specified Ligand (labeled "1") and Receptor (labeled "a", "b", and "c")

Kinetic rate constants of association (k_{on}; in units of $M^{-1}s^{-1}$) and dissociation (k_{off}; in units of s^{-1})

Structural parameters for the domain architecture of a monovalent Ligand B

Highlighted is the single domain diameter (ϕ, units of Angstrom)

d. User input parameterization of Ligand C

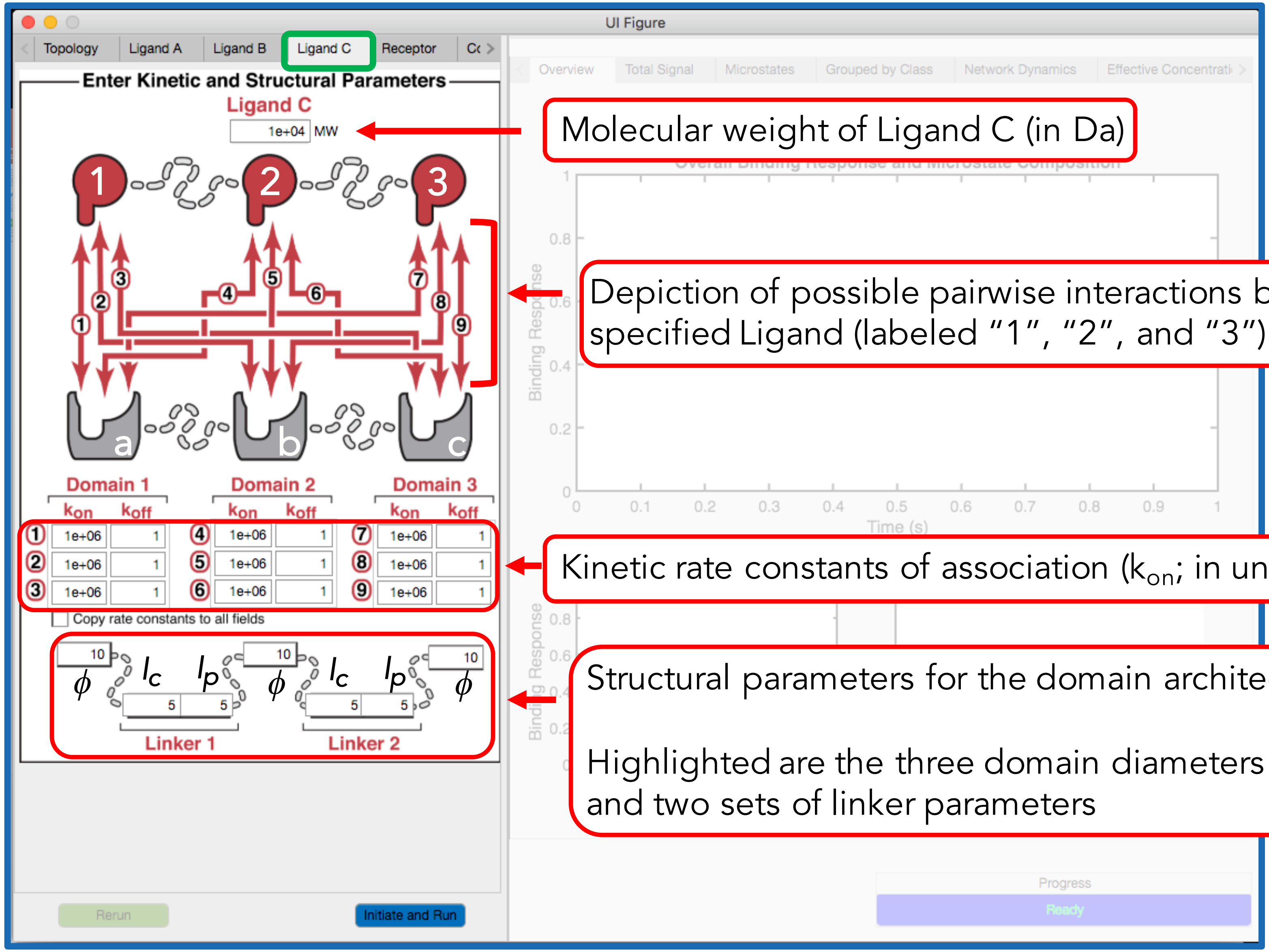

Molecular weight of Ligand C (in Da)

Depiction of possible pairwise interactions between the binding domains of the specified Ligand (labeled “1”, “2”, and “3”) and Receptor (labeled “a”, “b”, and “c”)

Kinetic rate constants of association (k_{on}; in units of $M^{-1}s^{-1}$) and dissociation (k_{off}; in units of s^{-1})

Structural parameters for the domain architecture of a trivalent Ligand C

Highlighted are the three domain diameters (ϕ, units of Angstrom) and two sets of linker parameters

3. Parameterizing the multivalent system in the Receptor tab

e. User input parameterization of the Receptor

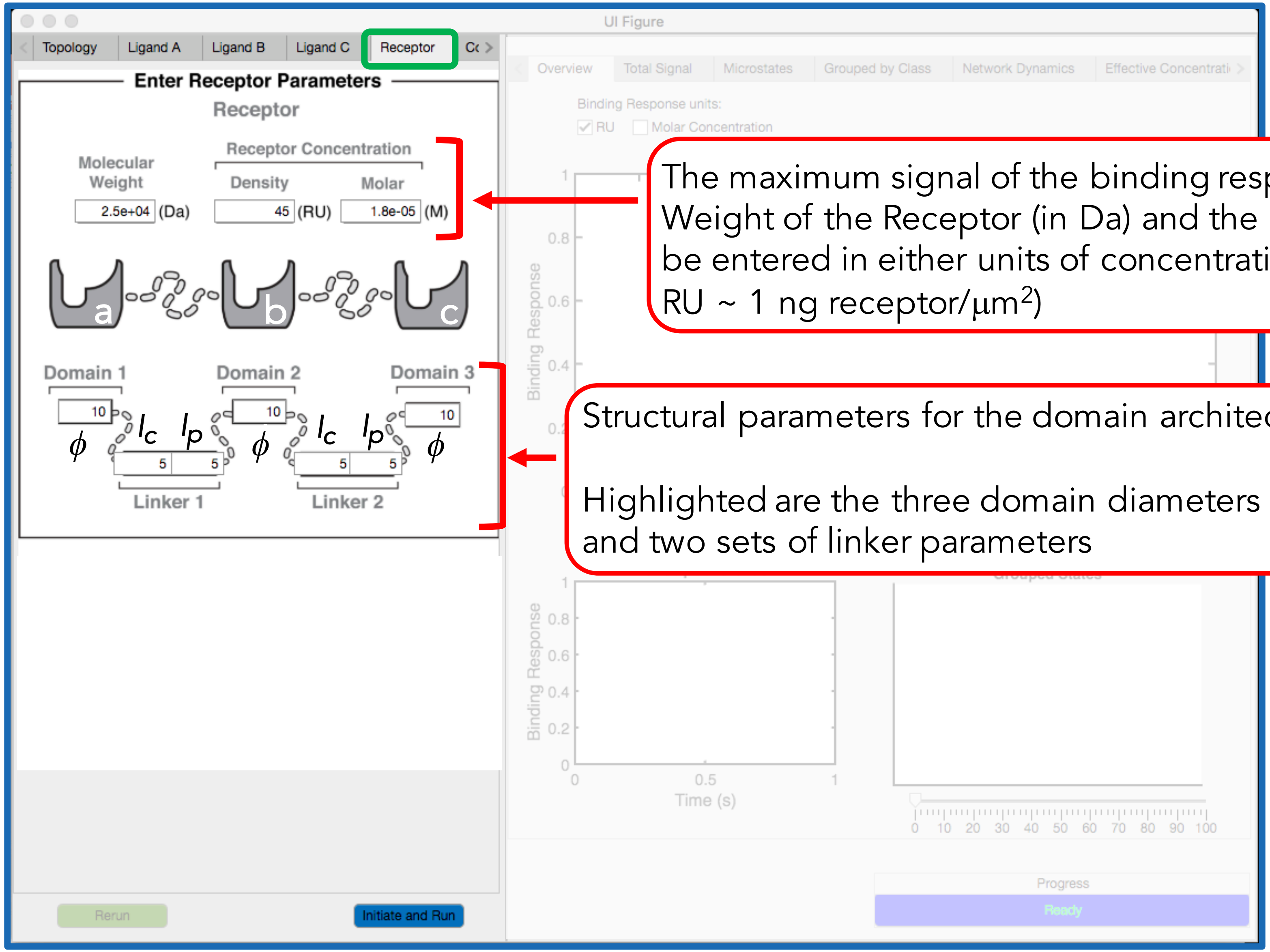

The maximum signal of the binding response is related to the Molecular Weight of the Receptor (in Da) and the Receptor Concentration, which can be entered in either units of concentration (M) or surface density (where 1 RU ~ 1 ng receptor/μm^2)

Structural parameters for the domain architecture of a trivalent Receptor

Highlighted are the three domain diameters (ϕ, units of Angstrom) and two sets of linker parameters

3. Parameterizing the multivalent system in the Concentrations tab

f. User input parameterization of the Ligand Concentrations

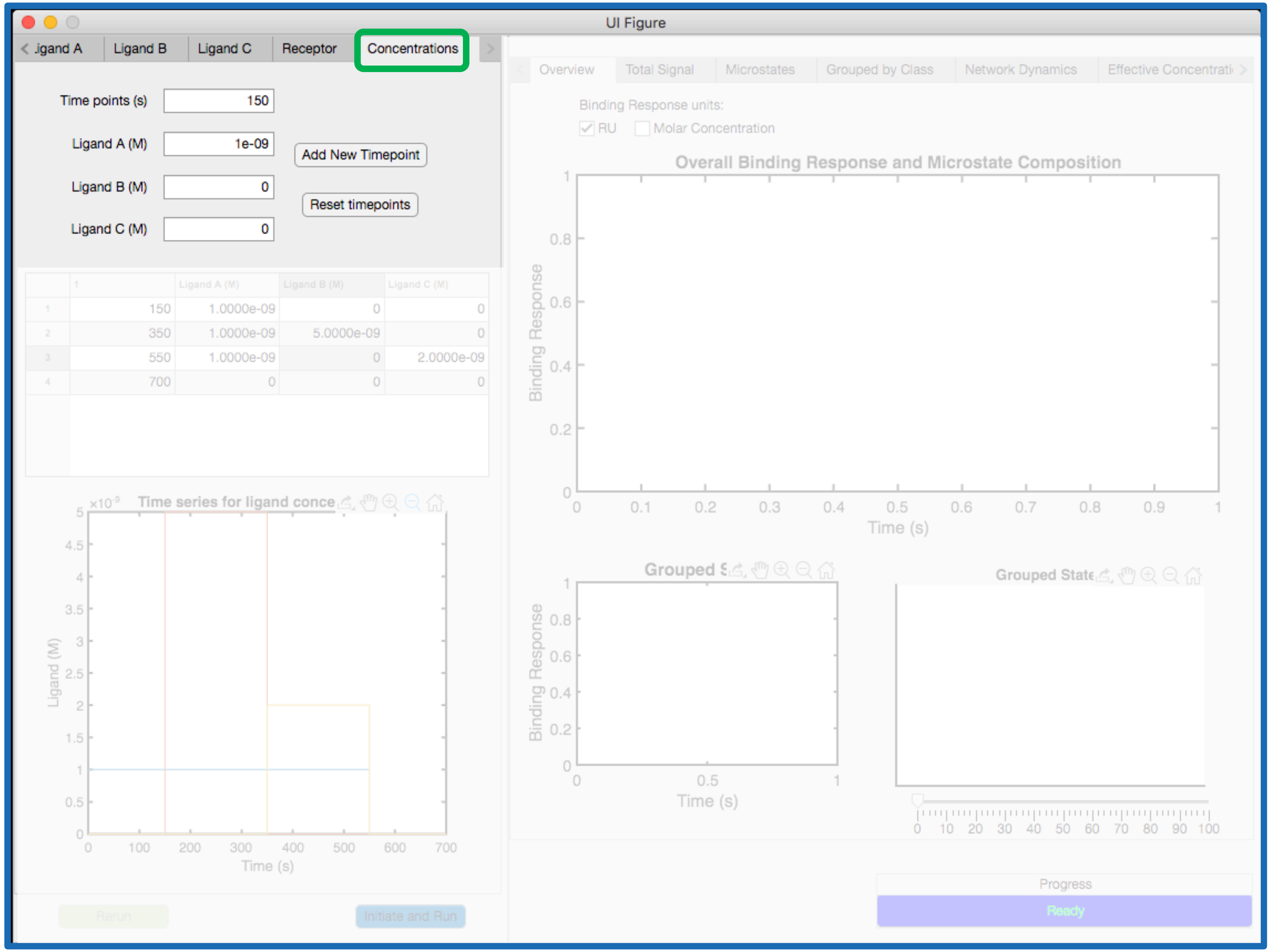

3. Parameterizing the multivalent system in the Concentrations tab

f. User input parameterization of the Ligand Concentrations

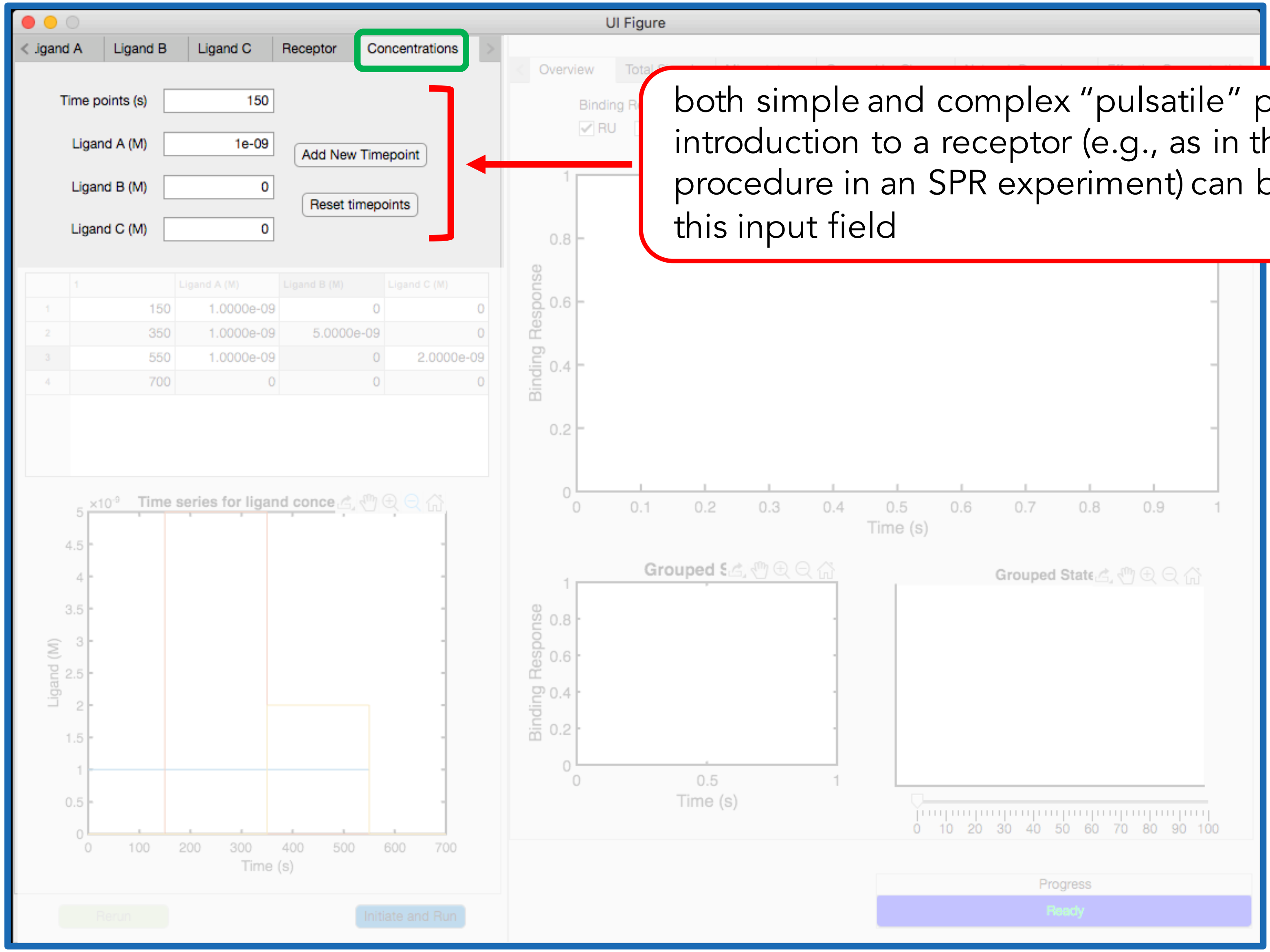

both simple and complex "pulsatile" patterns of ligand introduction to a receptor (e.g., as in the analyte injection procedure in an SPR experiment) can be specified through this input field

f. User input parameterization of the Ligand Concentrations

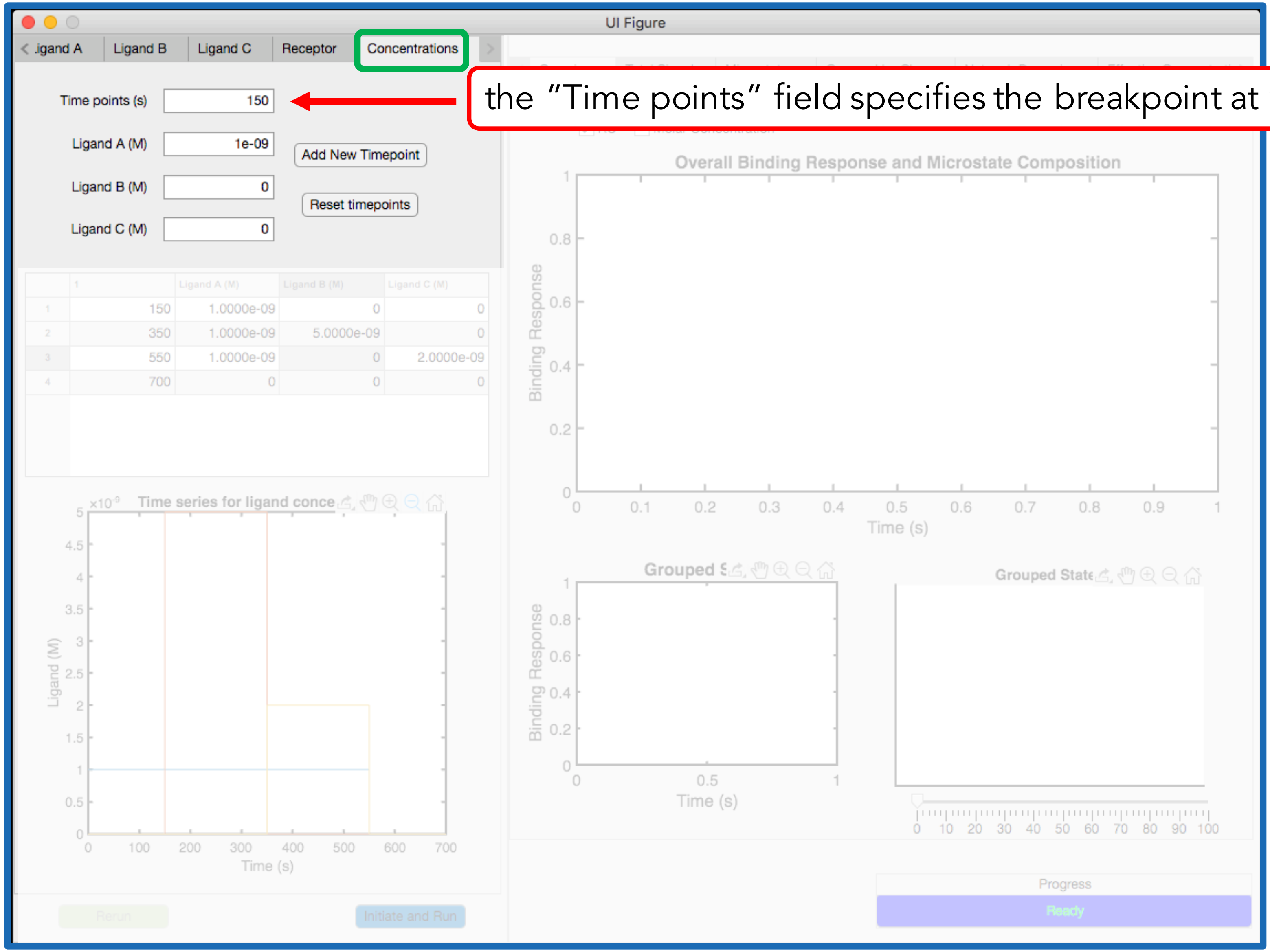

the "Time points" field specifies the breakpoint at which a specified ligand "injection" ends

3. Parameterizing the multivalent system in the Concentrations tab

f. User input parameterization of the Ligand Concentrations

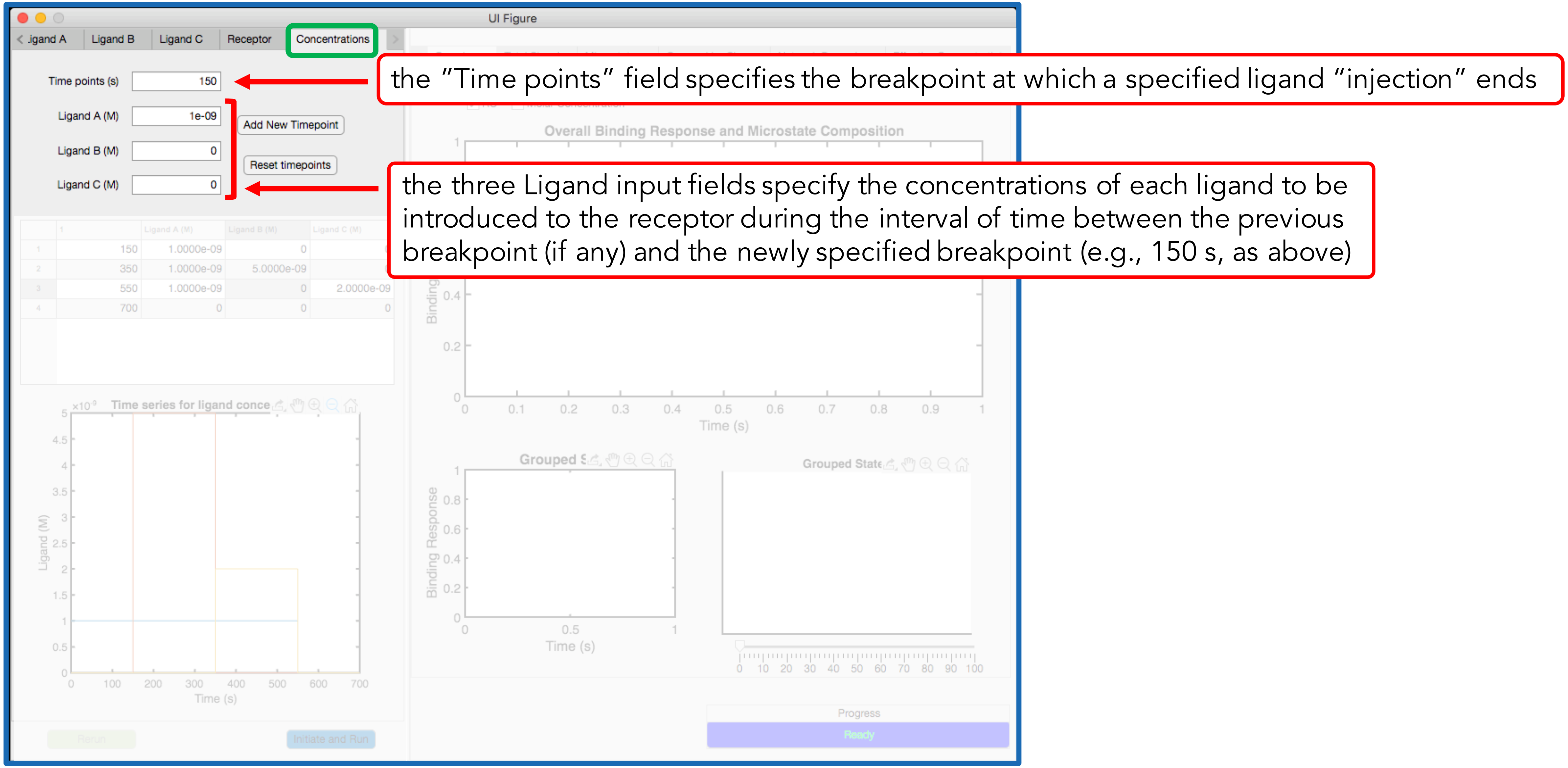

f. User input parameterization of the Ligand Concentrations

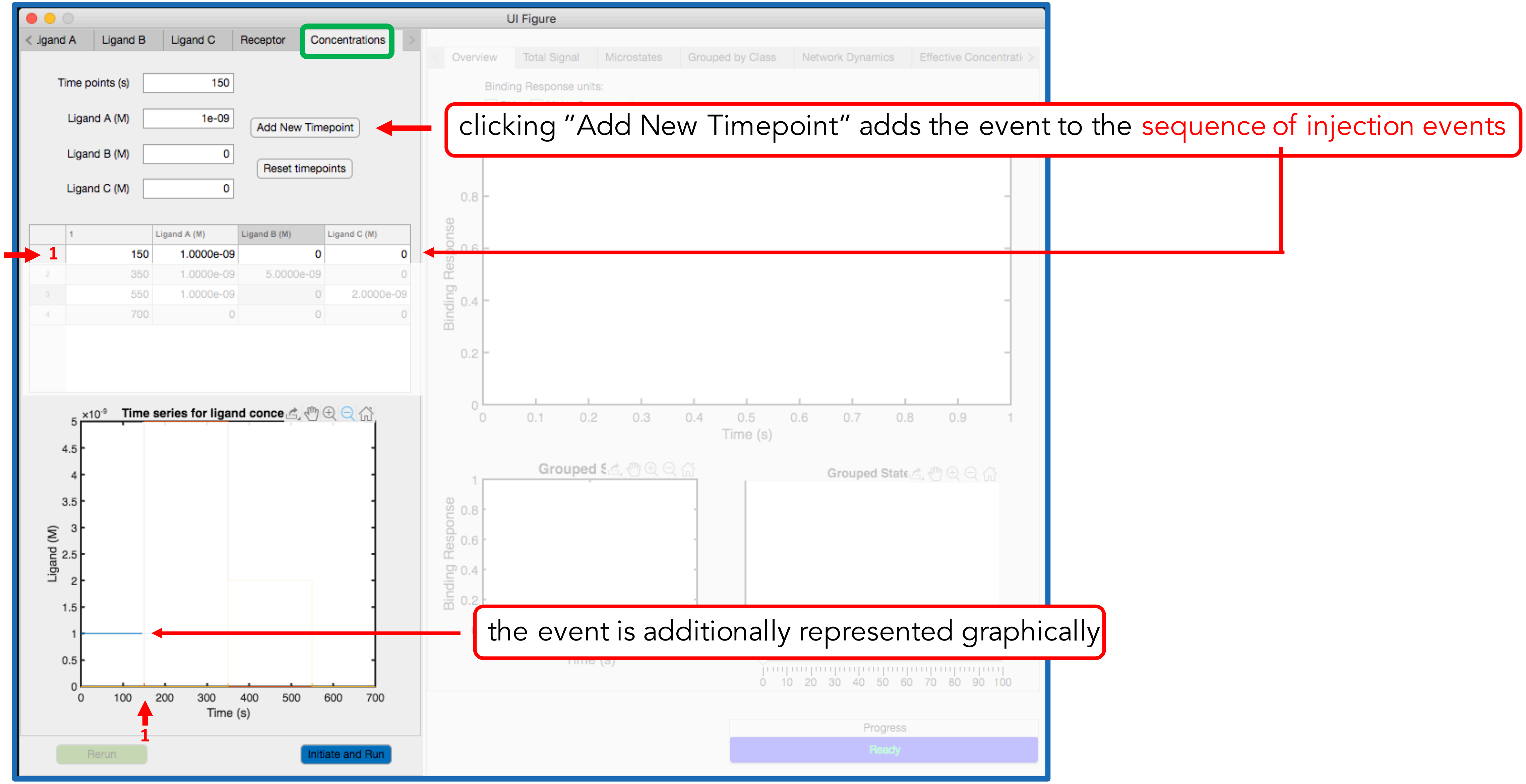

f. User input parameterization of the Ligand Concentrations

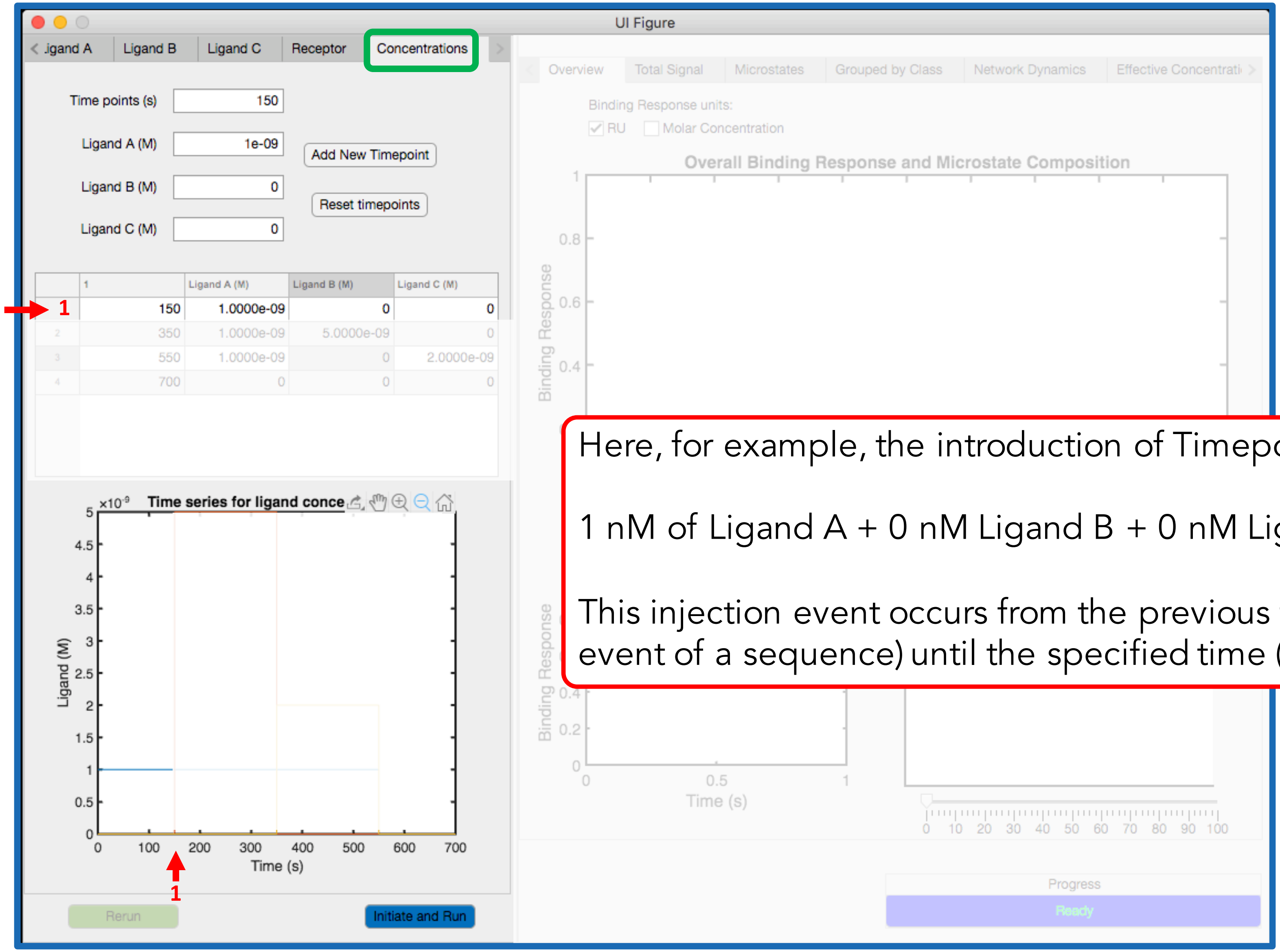

Here, for example, the introduction of Timepoint 1 specifies the following event:

1 nM of Ligand A + 0 nM Ligand B + 0 nM Ligand C

This injection event occurs from the previous timepoint (by default, 0 s for the first event of a sequence) until the specified time (here, 150 s)

f. User input parameterization of the Ligand Concentrations

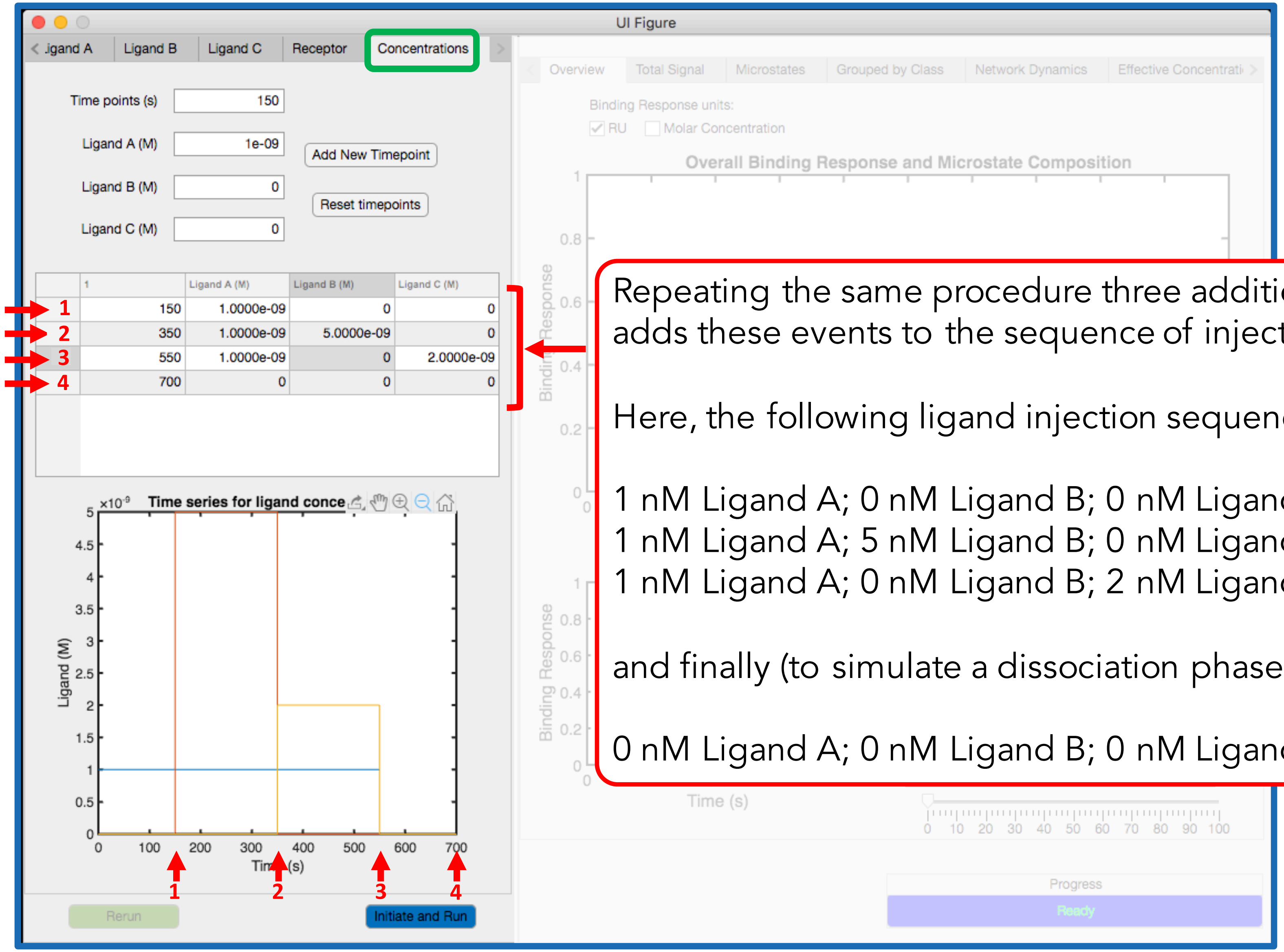

Repeating the same procedure three additional times (@ 350 s, 550 s, and 700 s) adds these events to the sequence of injections

Here, the following ligand injection sequence is programmed:

1 nM Ligand A; 0 nM Ligand B; 0 nM Ligand C; from 0 s – 150 s
1 nM Ligand A; 5 nM Ligand B; 0 nM Ligand C; from 150 s – 350 s
1 nM Ligand A; 0 nM Ligand B; 2 nM Ligand C from 350 s – 550 s

and finally (to simulate a dissociation phase),

0 nM Ligand A; 0 nM Ligand B; 0 nM Ligand C; from 550 s – 700 s

f. User input parameterization of the Ligand Concentrations

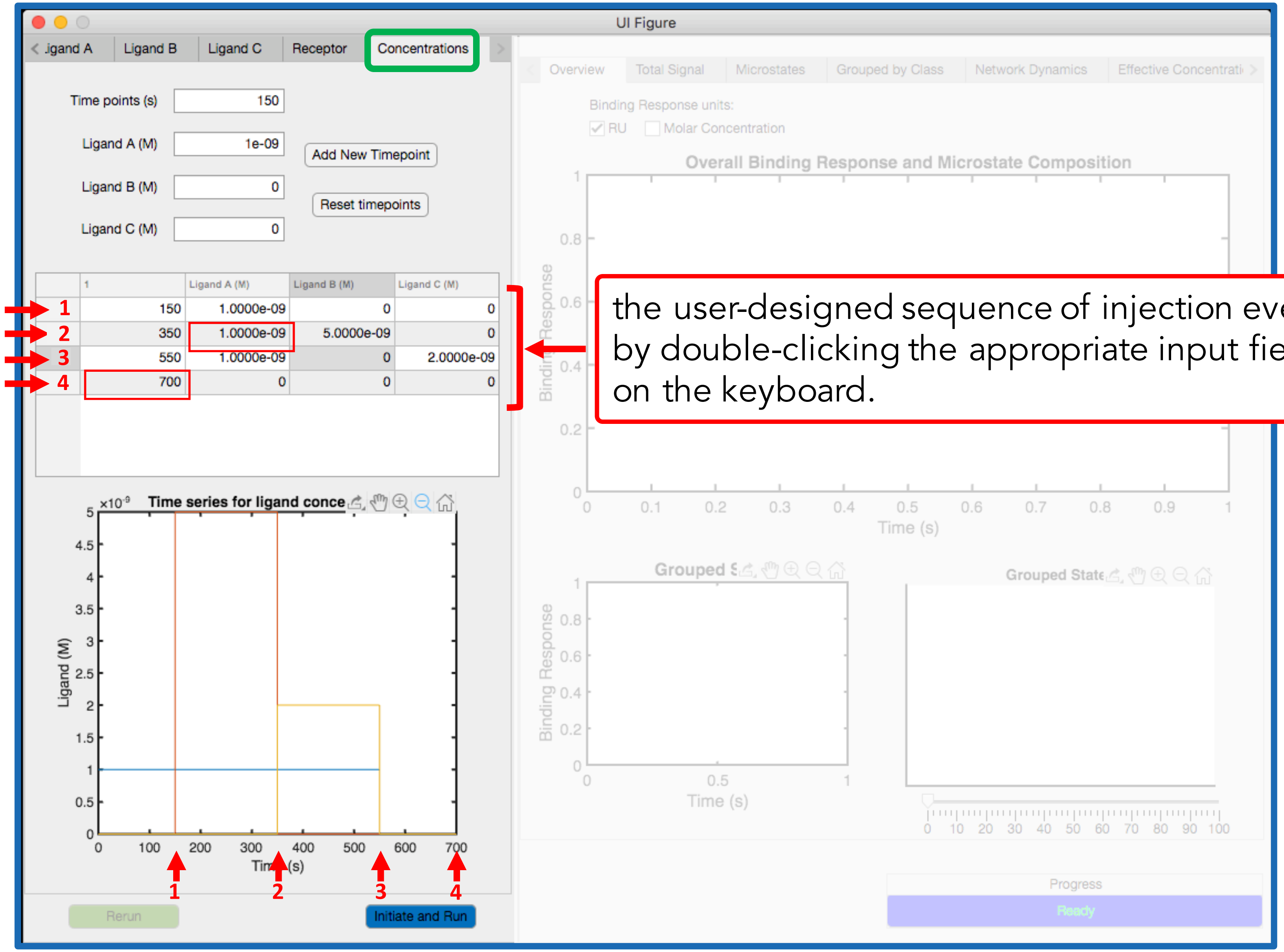

1		Ligand A (M)	Ligand B (M)	Ligand C (M)
1	150	1.0000e-09	0	0
2	350	1.0000e-09	5.0000e-09	0
3	550	1.0000e-09	0	2.0000e-09
4	700	0	0	0

the user-designed sequence of injection events can be manually edited (one-by-one) by double-clicking the appropriate input field, editing the value, and pressing “enter” on the keyboard.

f. User input parameterization of the Ligand Concentrations

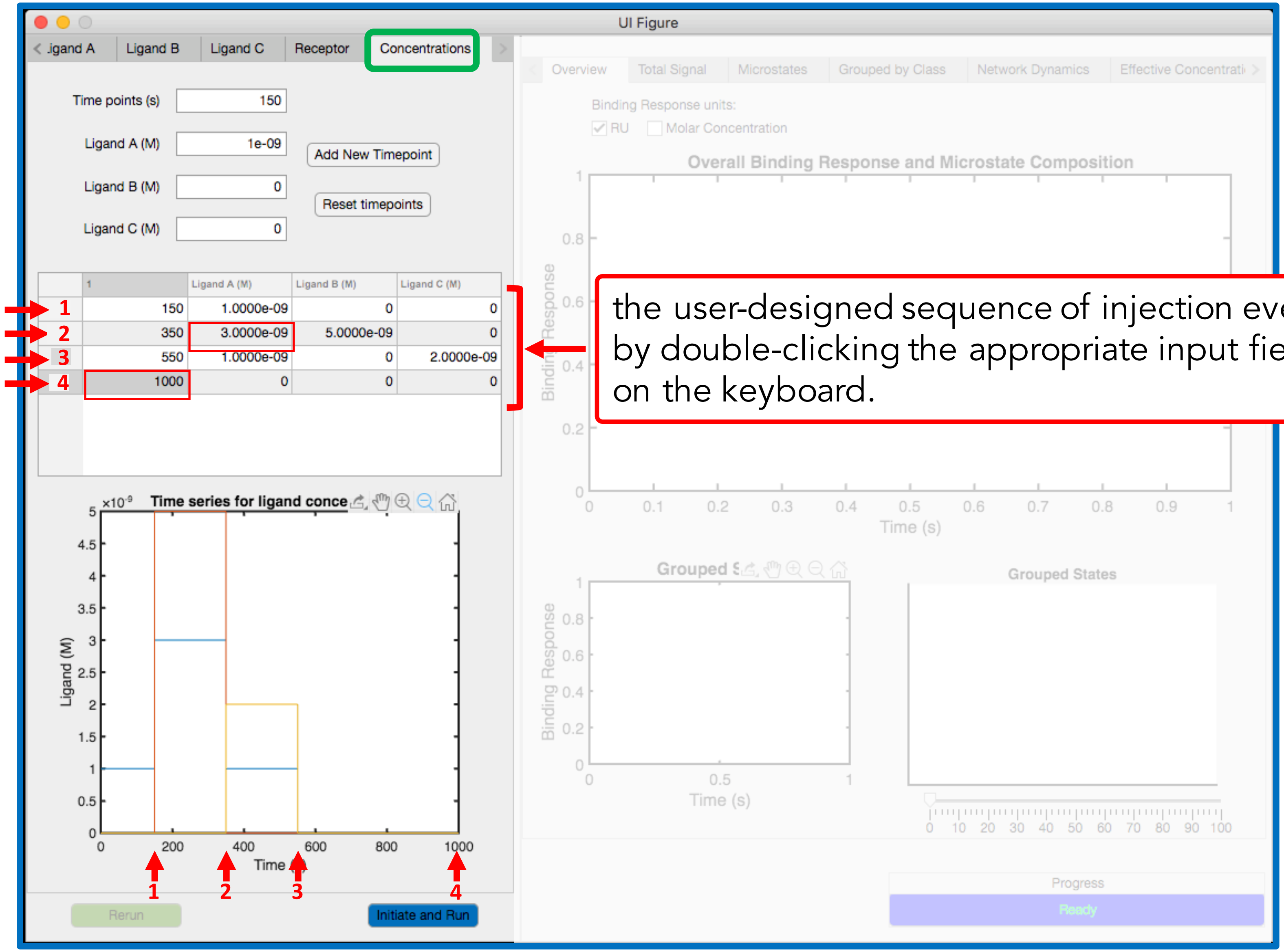

the user-designed sequence of injection events can be manually edited (one-by-one) by double-clicking the appropriate input field, editing the value, and pressing "enter" on the keyboard.

3. Parameterizing the multivalent system in the Concentrations tab

f. User input parameterization of the Ligand Concentrations

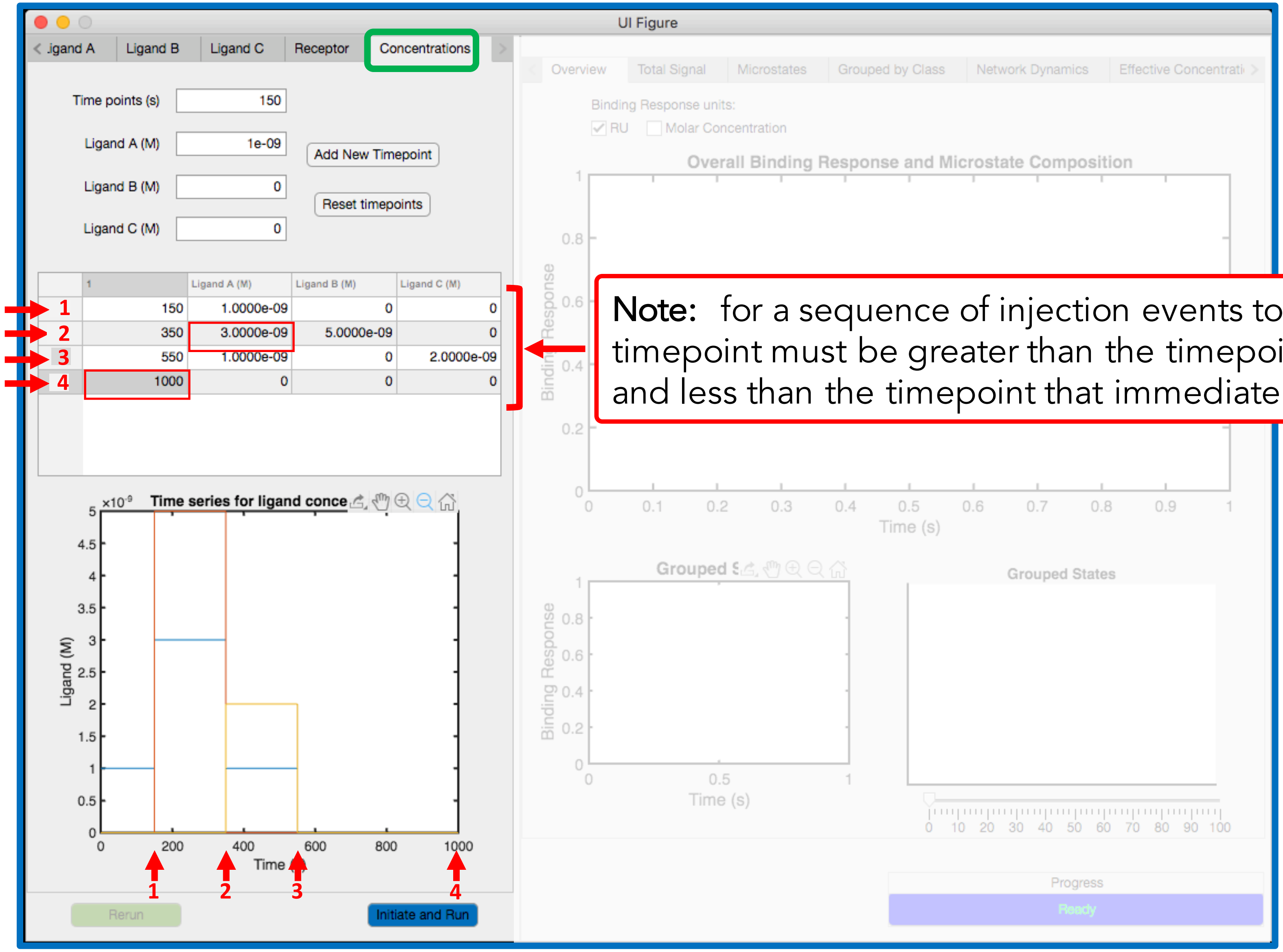

Note: for a sequence of injection events to be valid the designated time for each timepoint must be greater than the timepoint that precedes it (i.e., it must occur after) and less than the timepoint that immediately follows it (i.e., it must occur before)

f. User input parameterization of the Ligand Concentrations

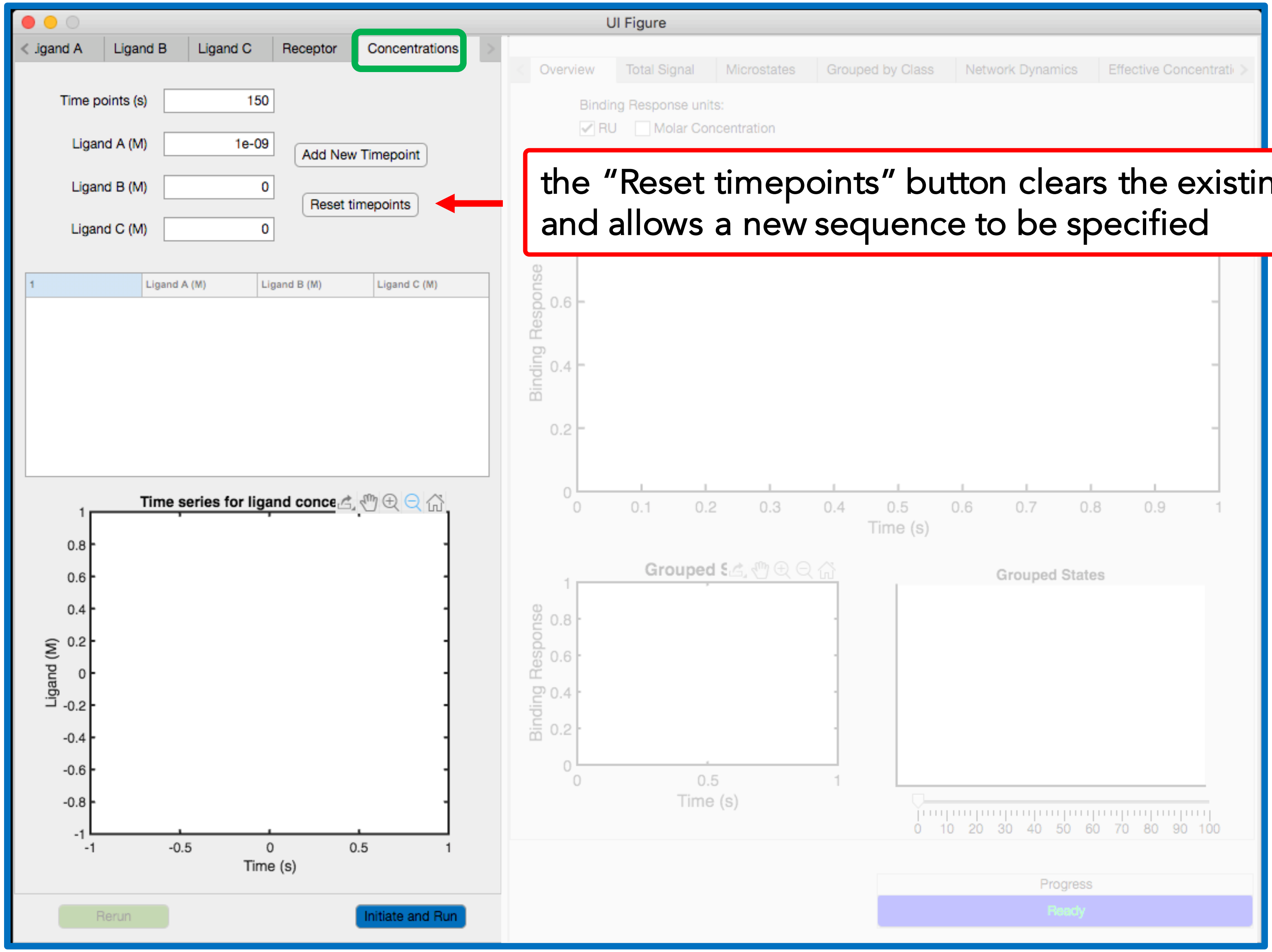

the "Reset timepoints" button clears the existing sequence of injection events and allows a new sequence to be specified

g. Initiate and Run *MVsim*

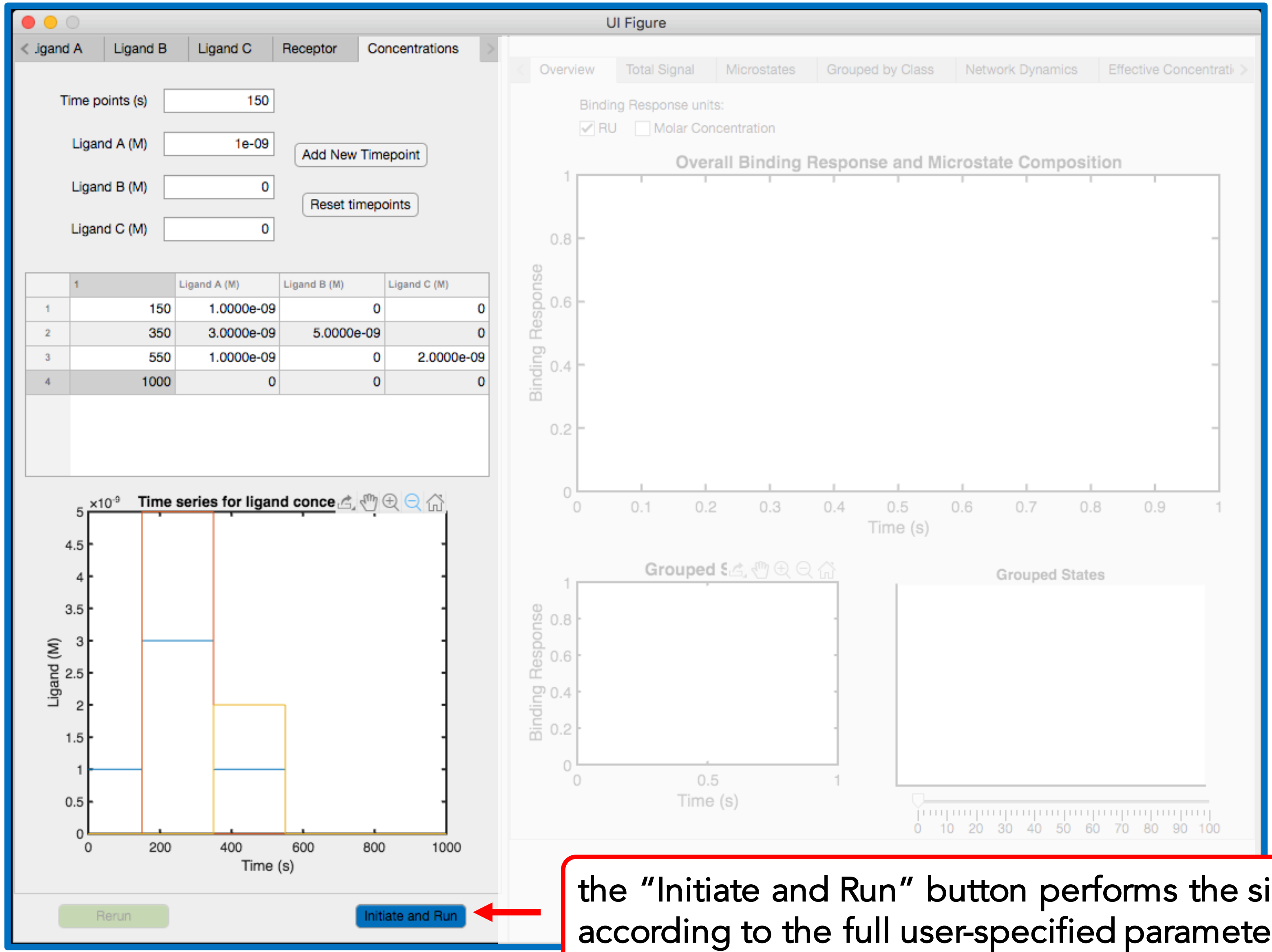

the "Initiate and Run" button performs the simulation according to the full user-specified parameterization

a. Output Overview

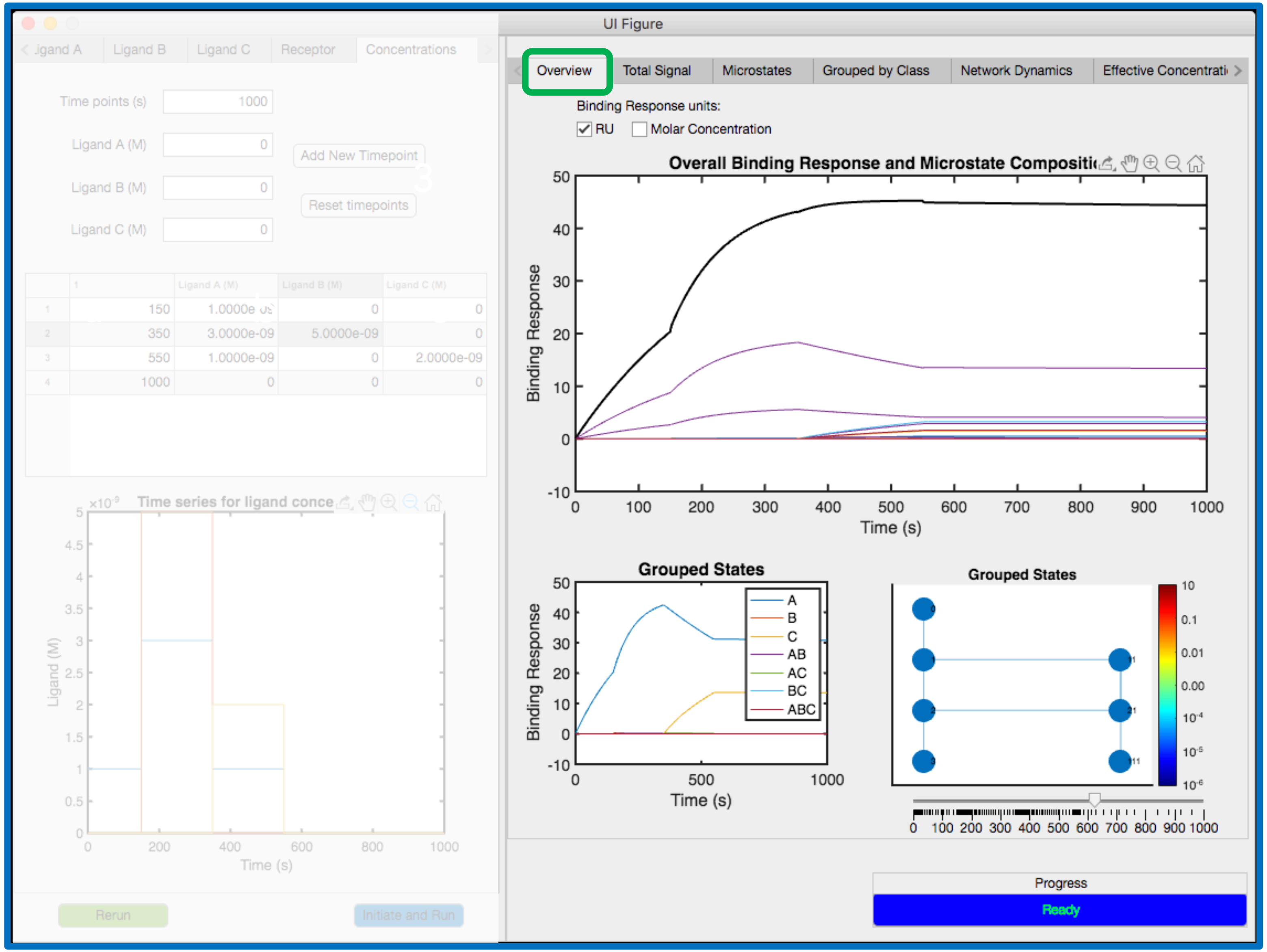

4. Navigating the *MVsim* output tabs

a. Output Overview

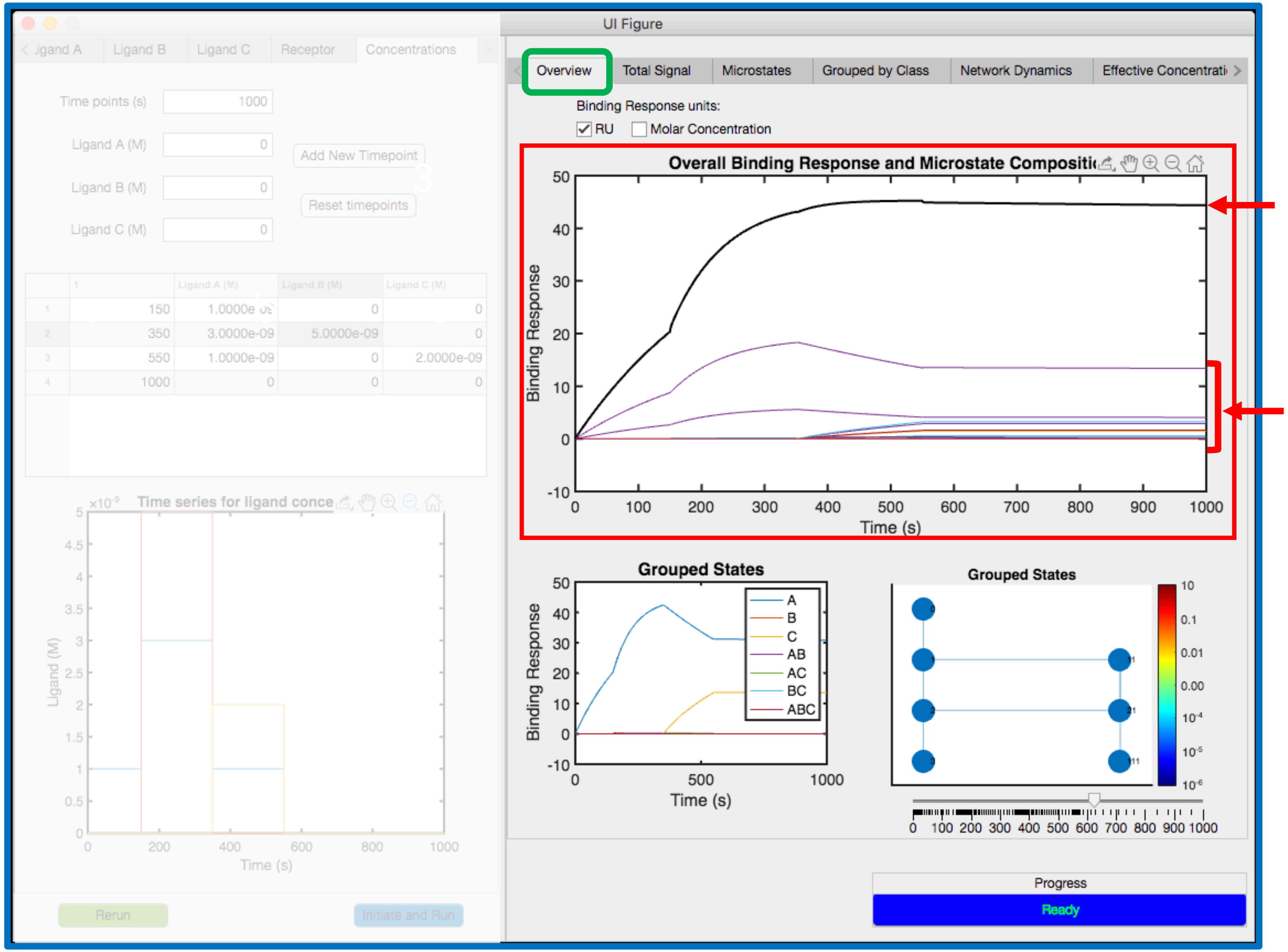

the total binding response dynamics (top black trace) represents the cumulative signal for all binding events that occur during the simulation (i.e., the measurable signal in an SPR experiment)

the conformational microstates (i.e., all monovalent, bivalent, and trivalent patterns of configuration that the multivalent and/or multi-ligand system populates)

4. Navigating the *MVsim* output tabs

a. Output Overview

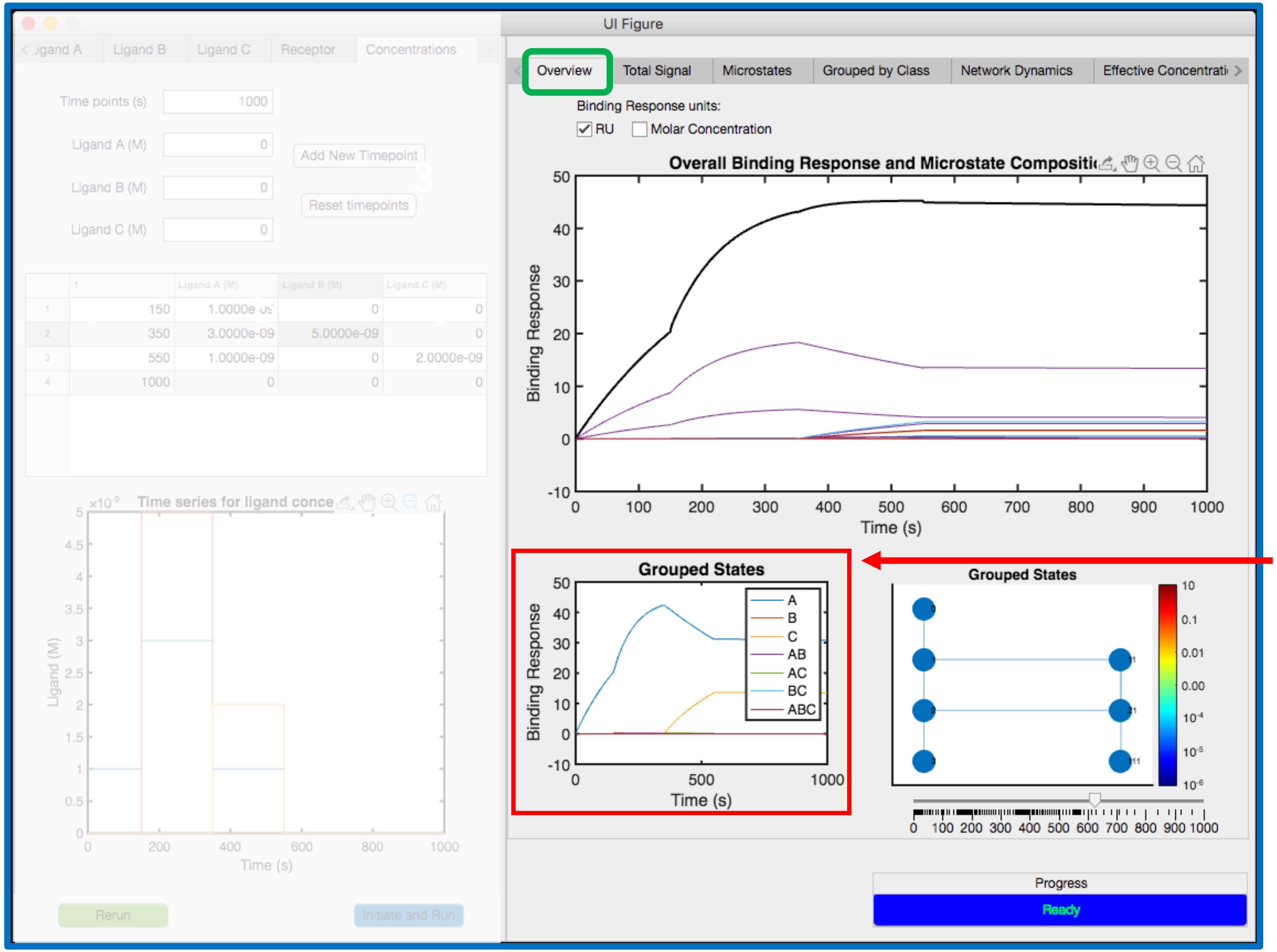

the "Grouped States" plots a simplified view of the overall binding response in which microstates are summed together based on their class. Here, the class represents microstates based on the ligand(s) from which they are composed

E.g., "ABC" represents the binding response from all microstates which entail a receptor bound simultaneously by Ligands A, B, and C

4. Navigating the *MVsim* output tabs

a. Output Overview

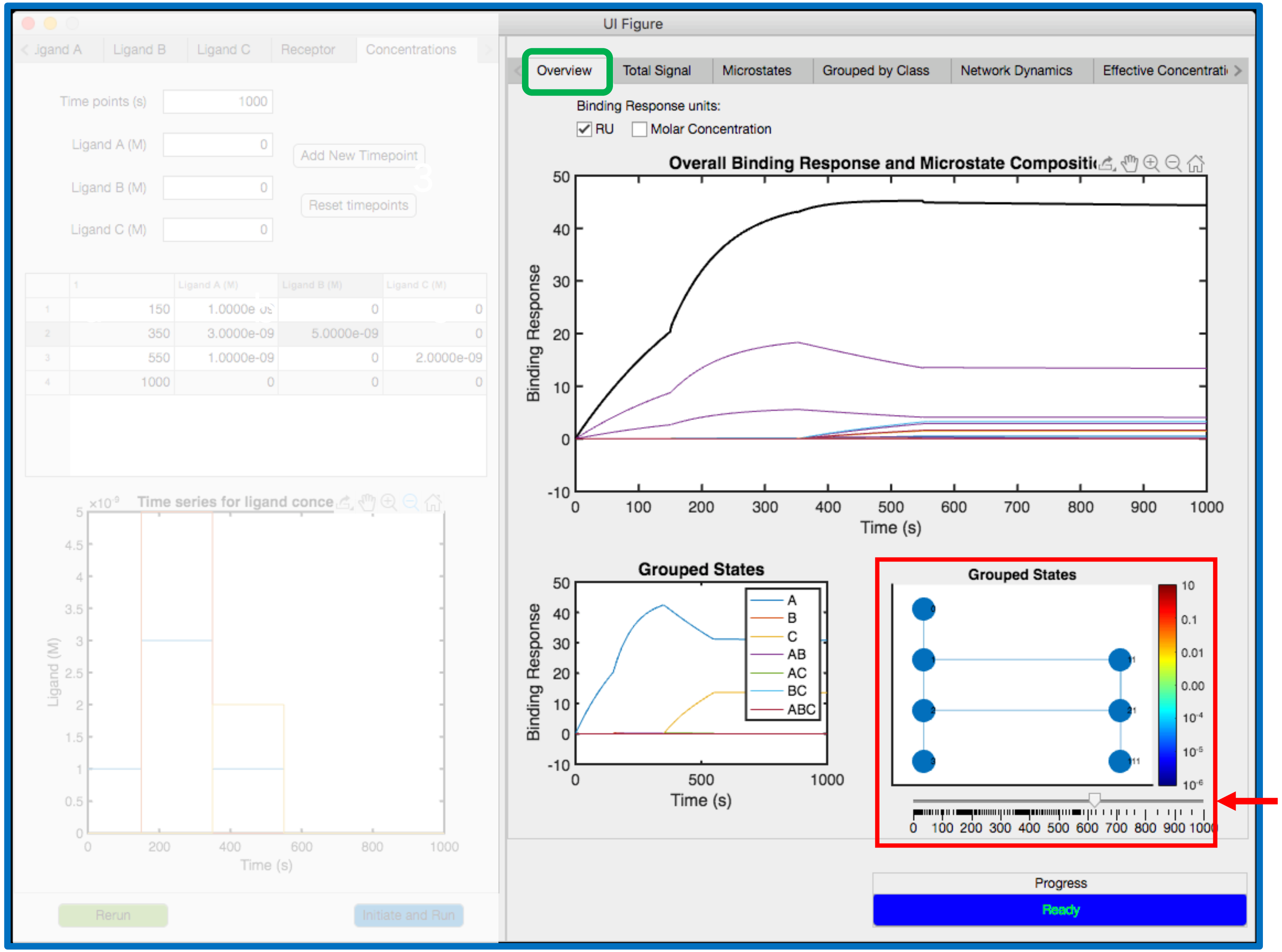

a "click-and-drag" slide bar enables the grouped states to be visualized at individual timepoints sampled during the entire length of the simulated interaction

b. Output Total Signal

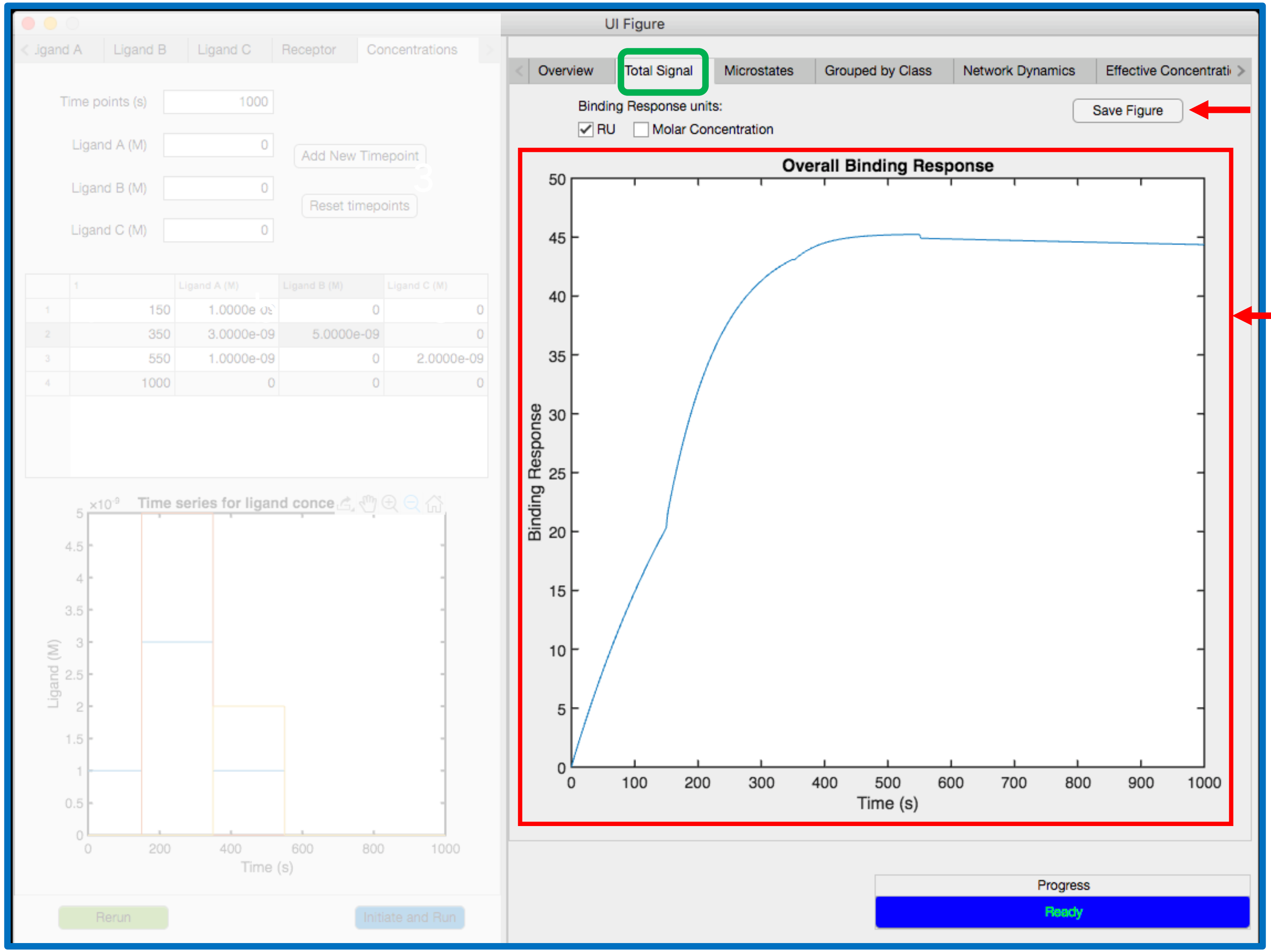

"Save Figure" exports the plot as a .fig file

single view of the overall binding response (i.e., the black trace from the Overview tab)

b. Output Total Signal

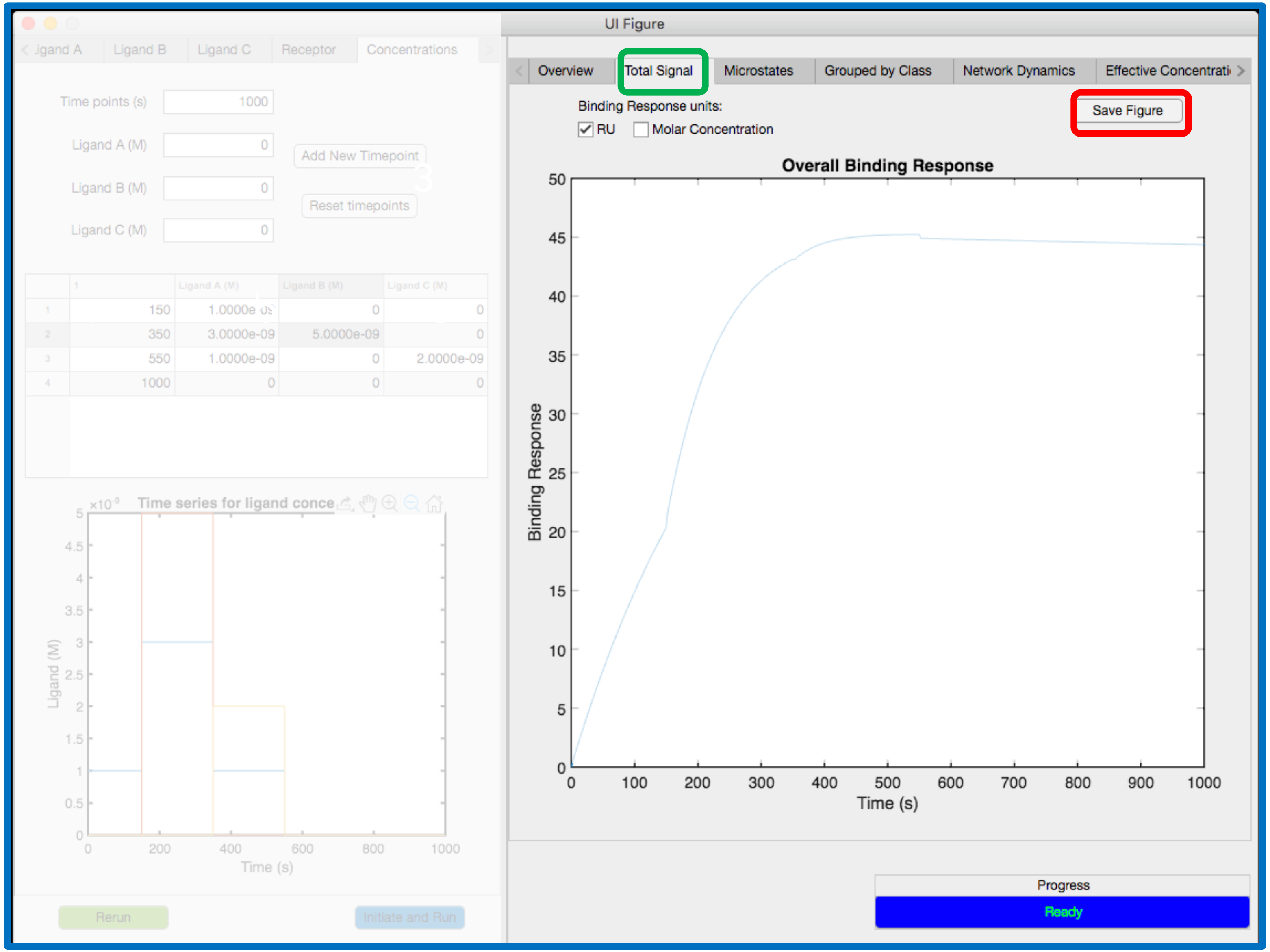

"Save Figure" exports the plot as a .fig file

b. Output Total Signal

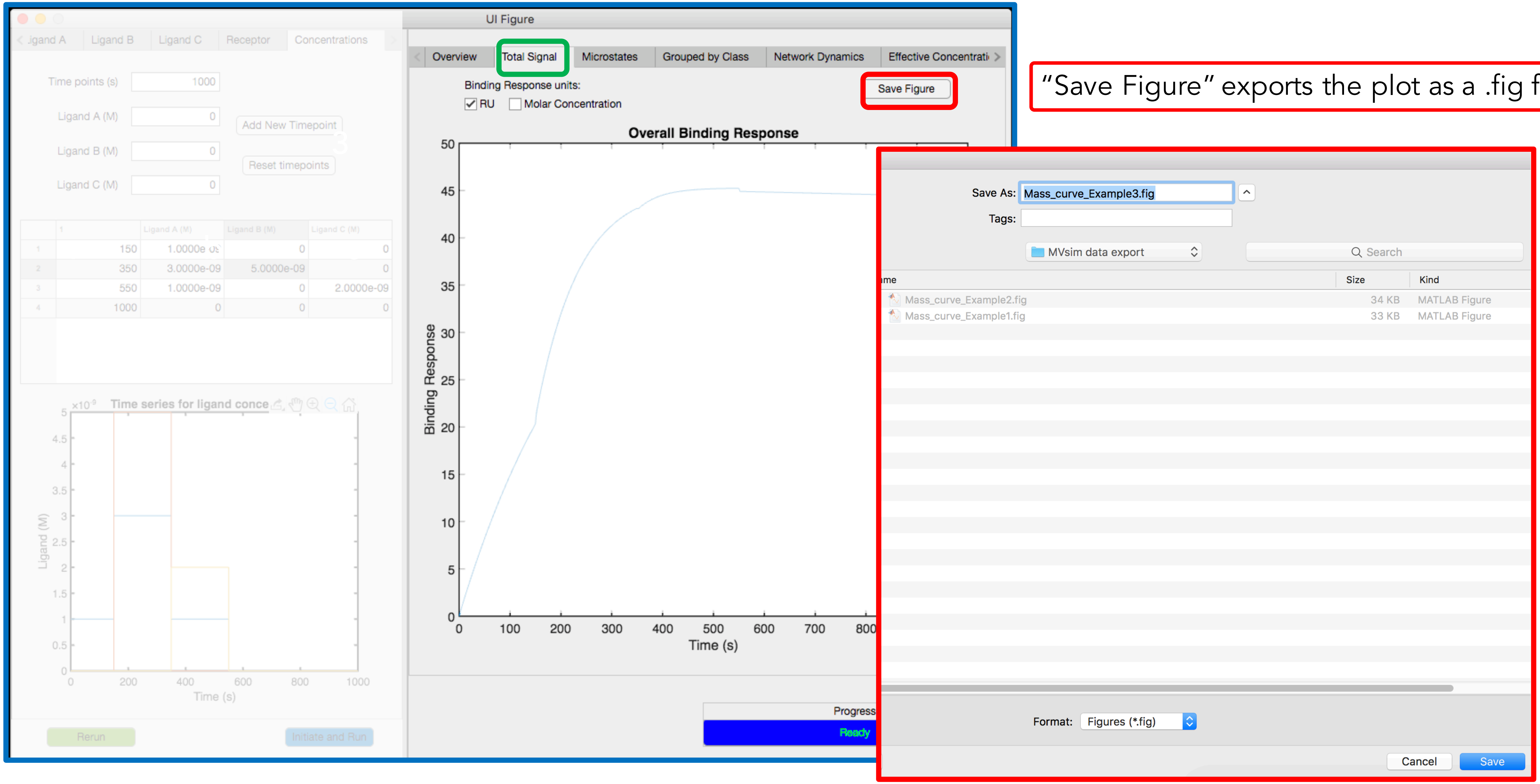

"Save Figure" exports the plot as a .fig file

b. Output Total Signal

Individual simulations saved to a folder

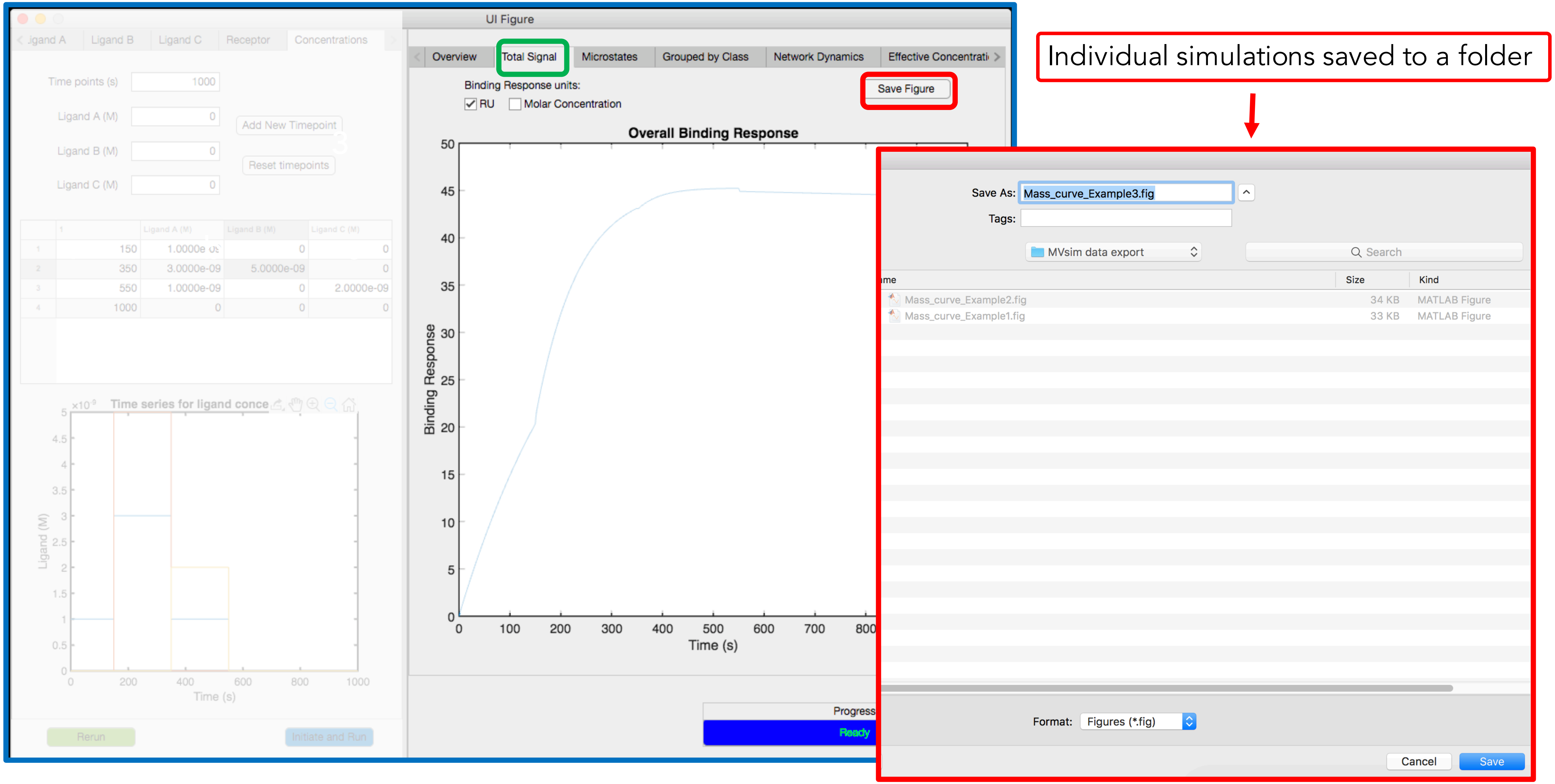

4. Navigating the *MVsim* output tabs

b. Output Total Signal

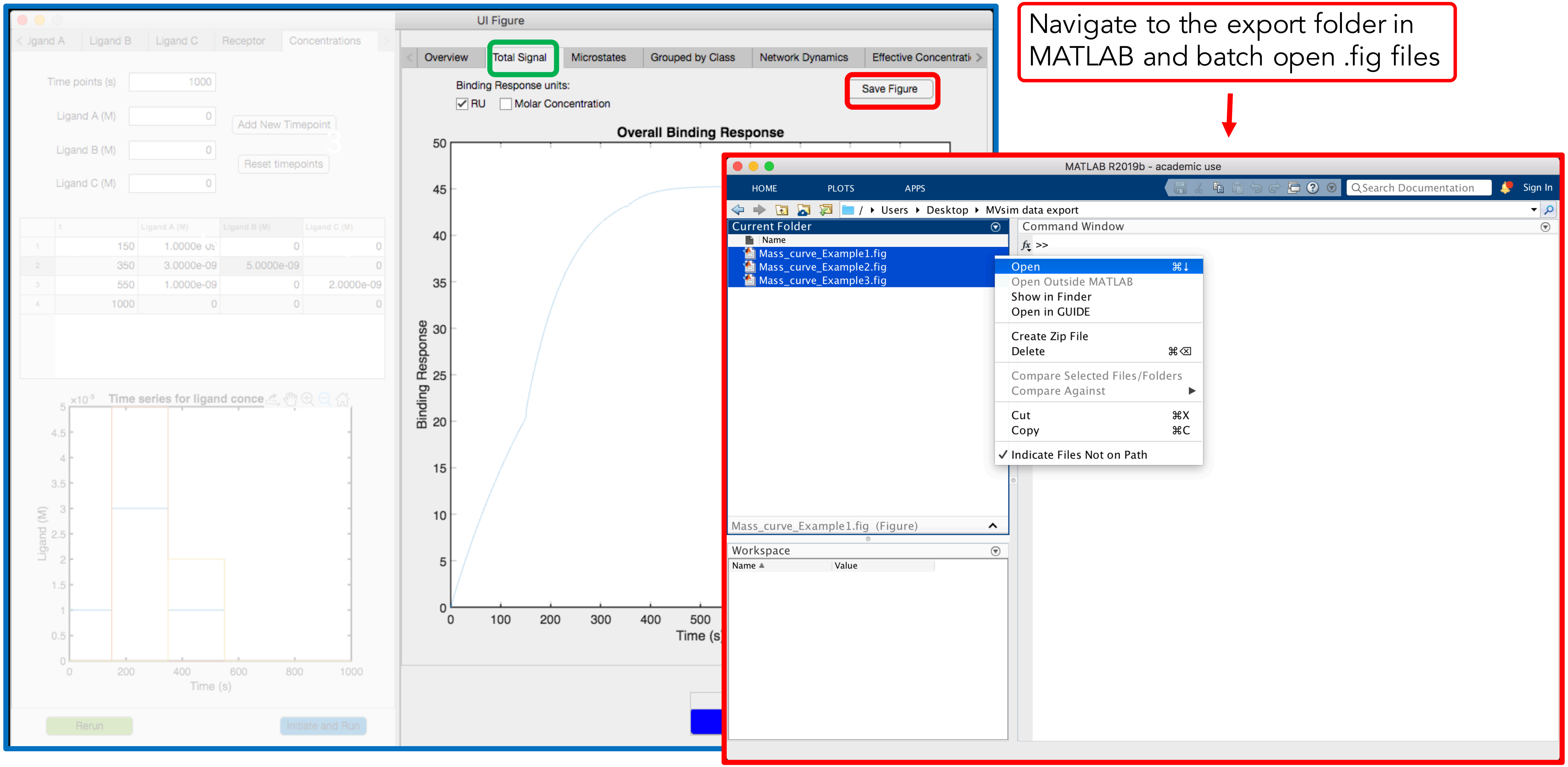

4. Navigating the *MVsim* output tabs

b. Output Total Signal

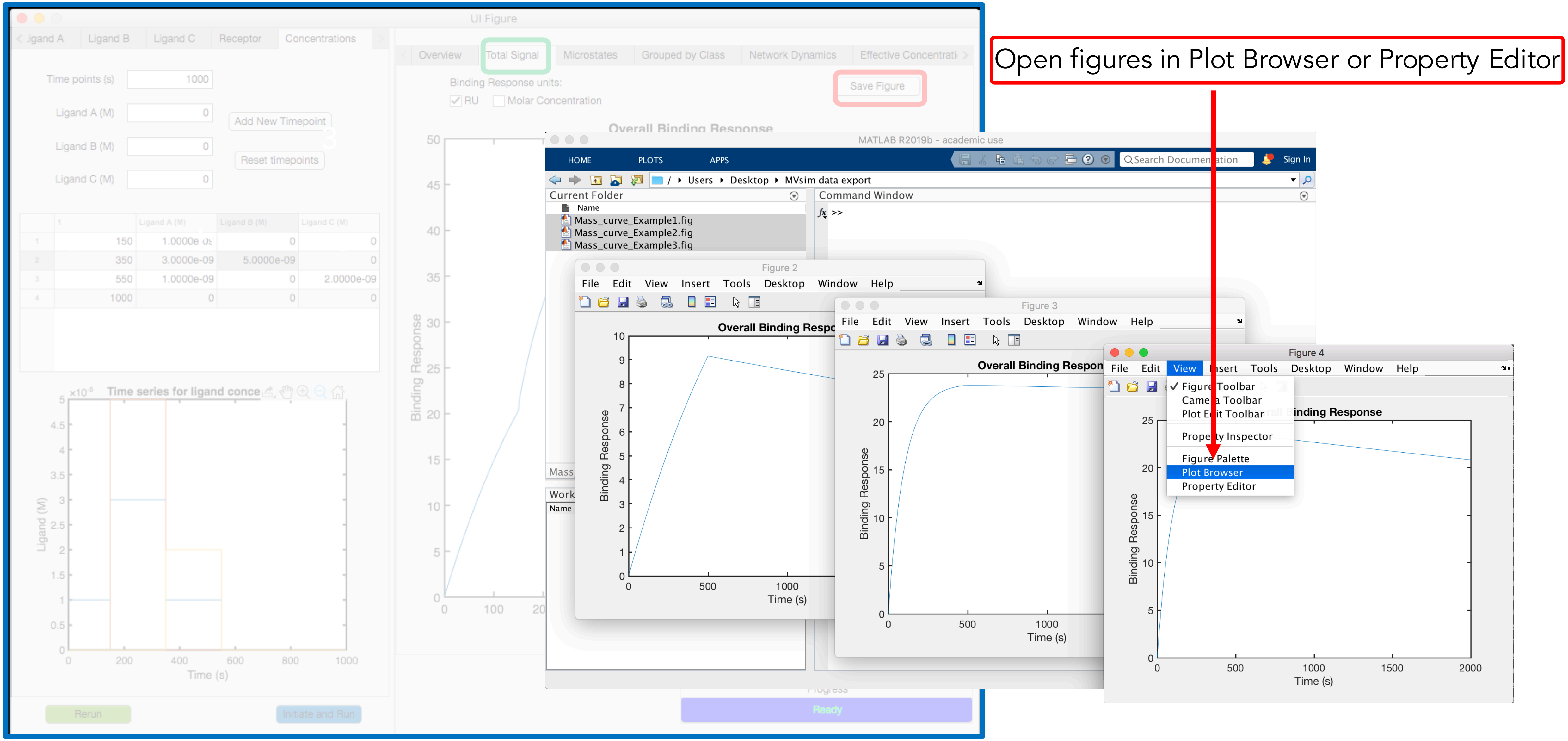

4. Navigating the *MVsim* output tabs

b. Output Total Signal

Copying and Pasting individual sensorgram curves into a common tab enables batch viewing of multiple superimposed simulations

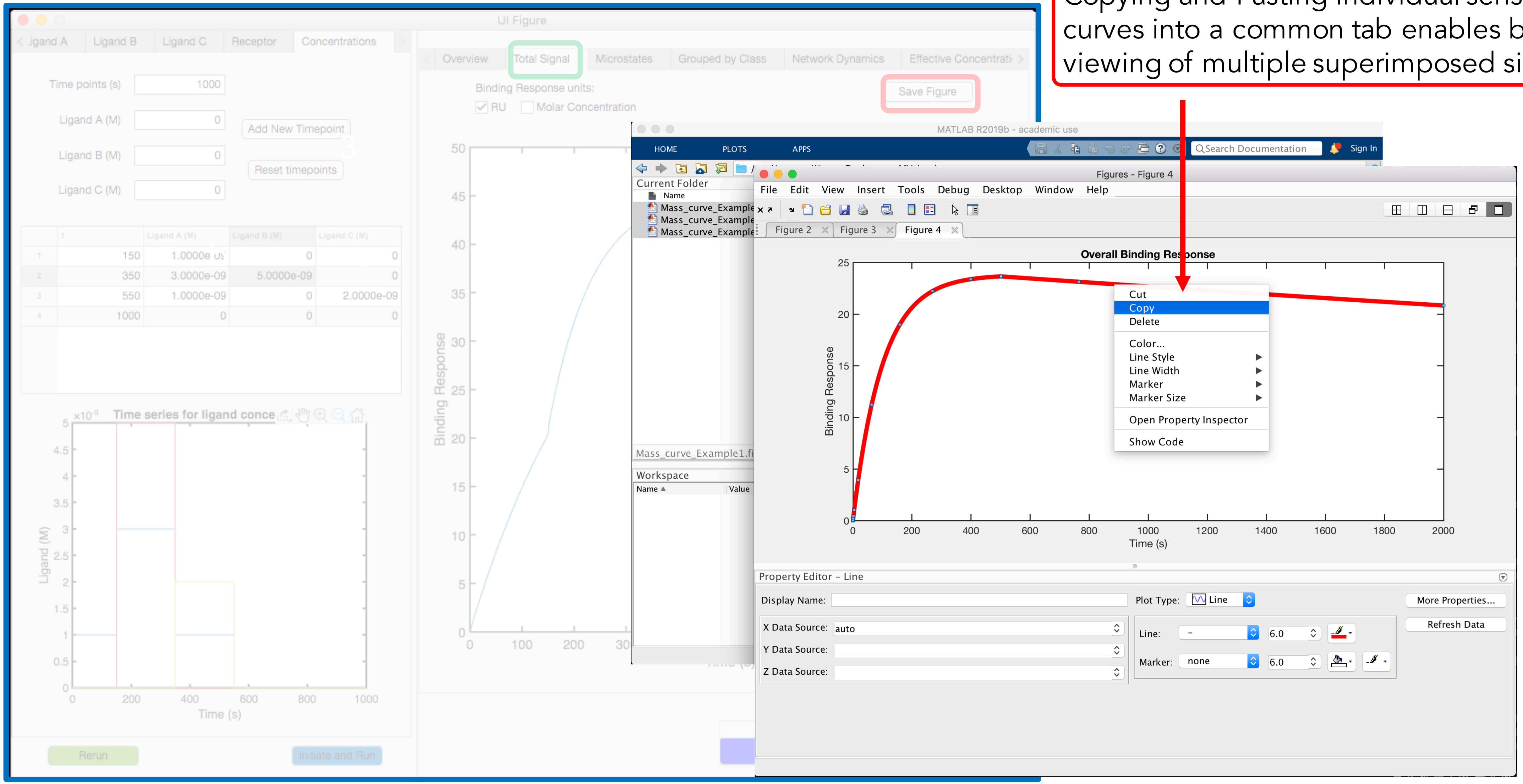

4. Navigating the *MVsim* output tabs

b. Output Total Signal

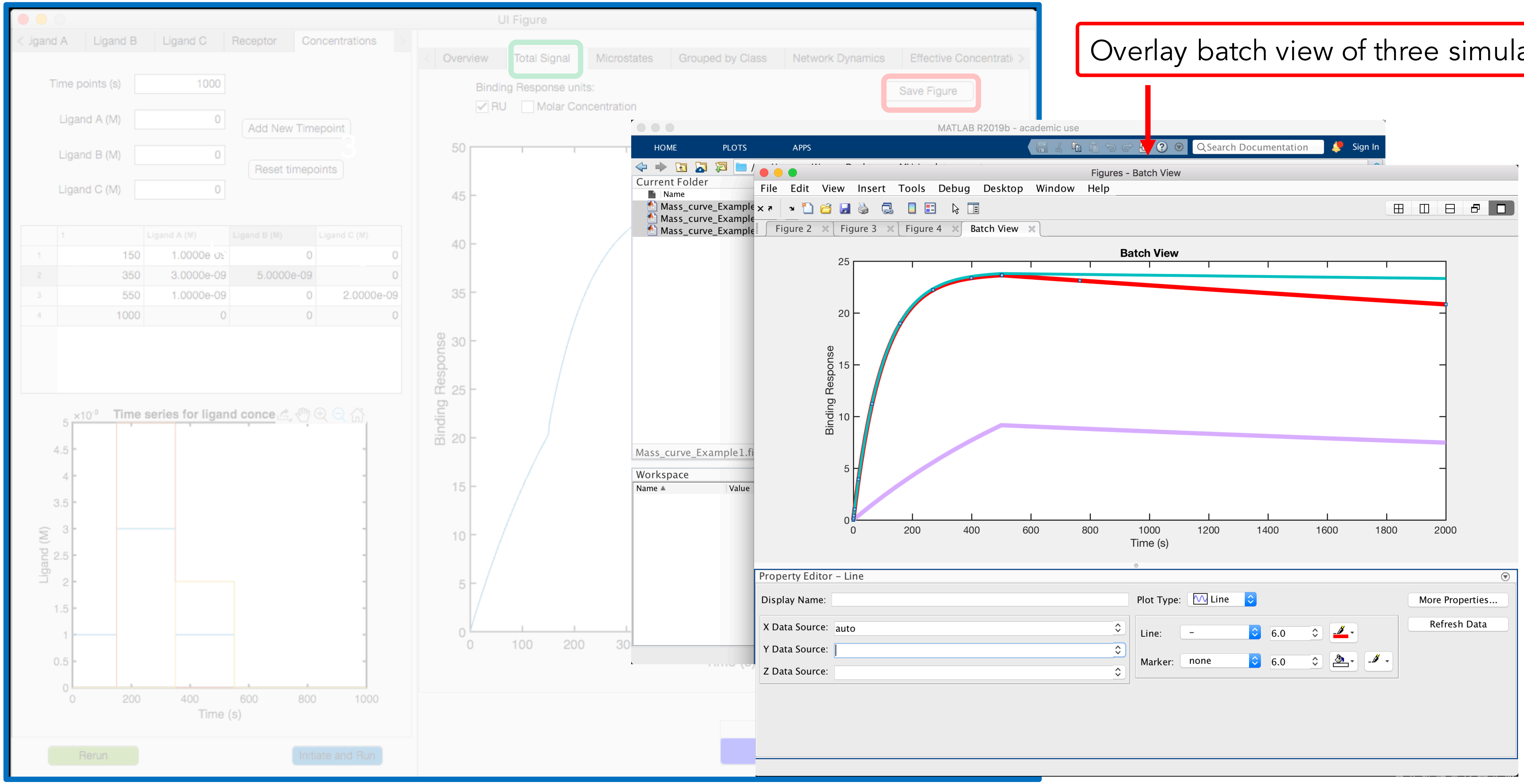

Overlay batch view of three simulations

b. Output Total Signal

Extraction of x,y datapoints for exported simulations enables further 'offline' analyses

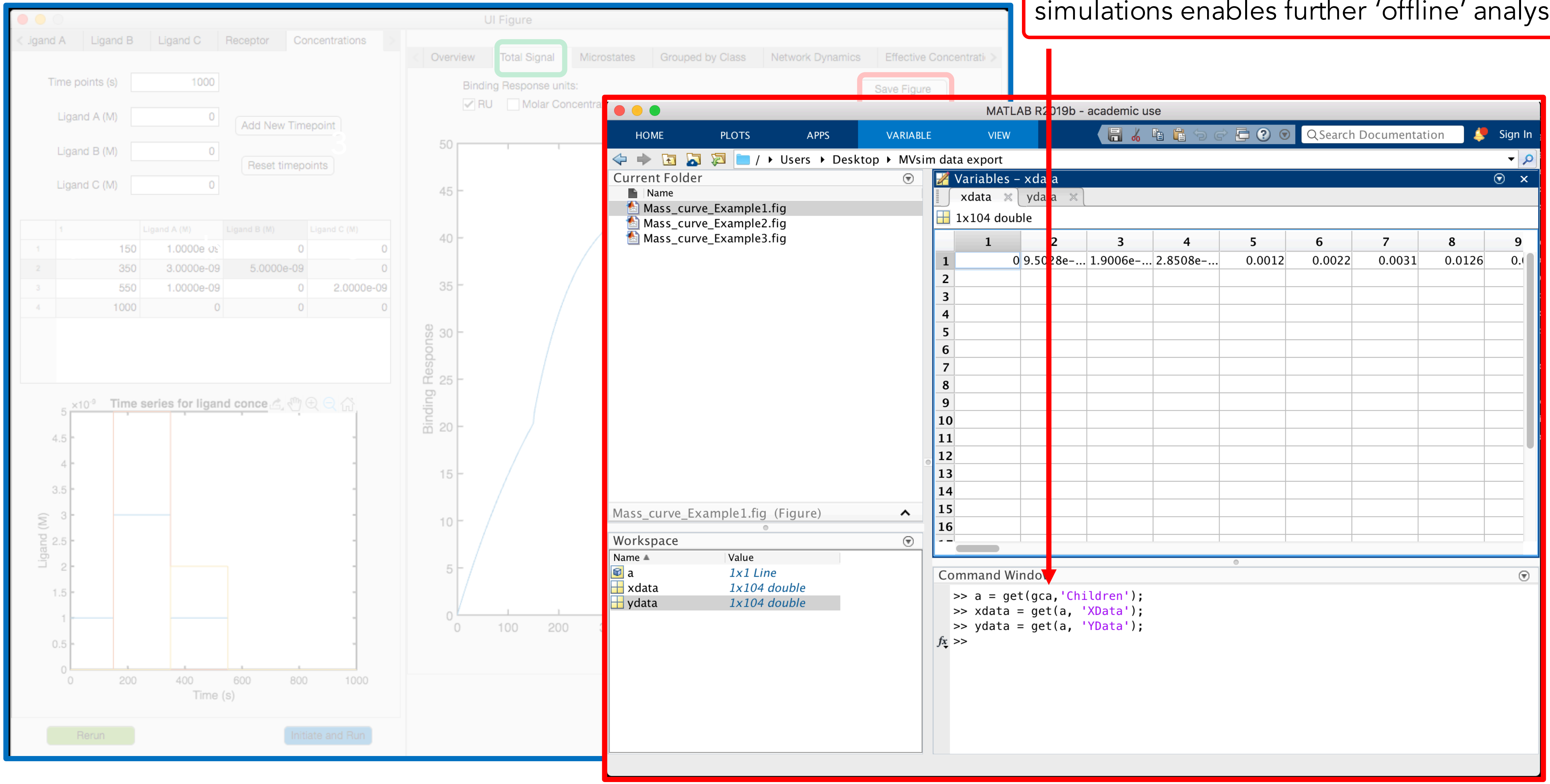

c. Output Microstates

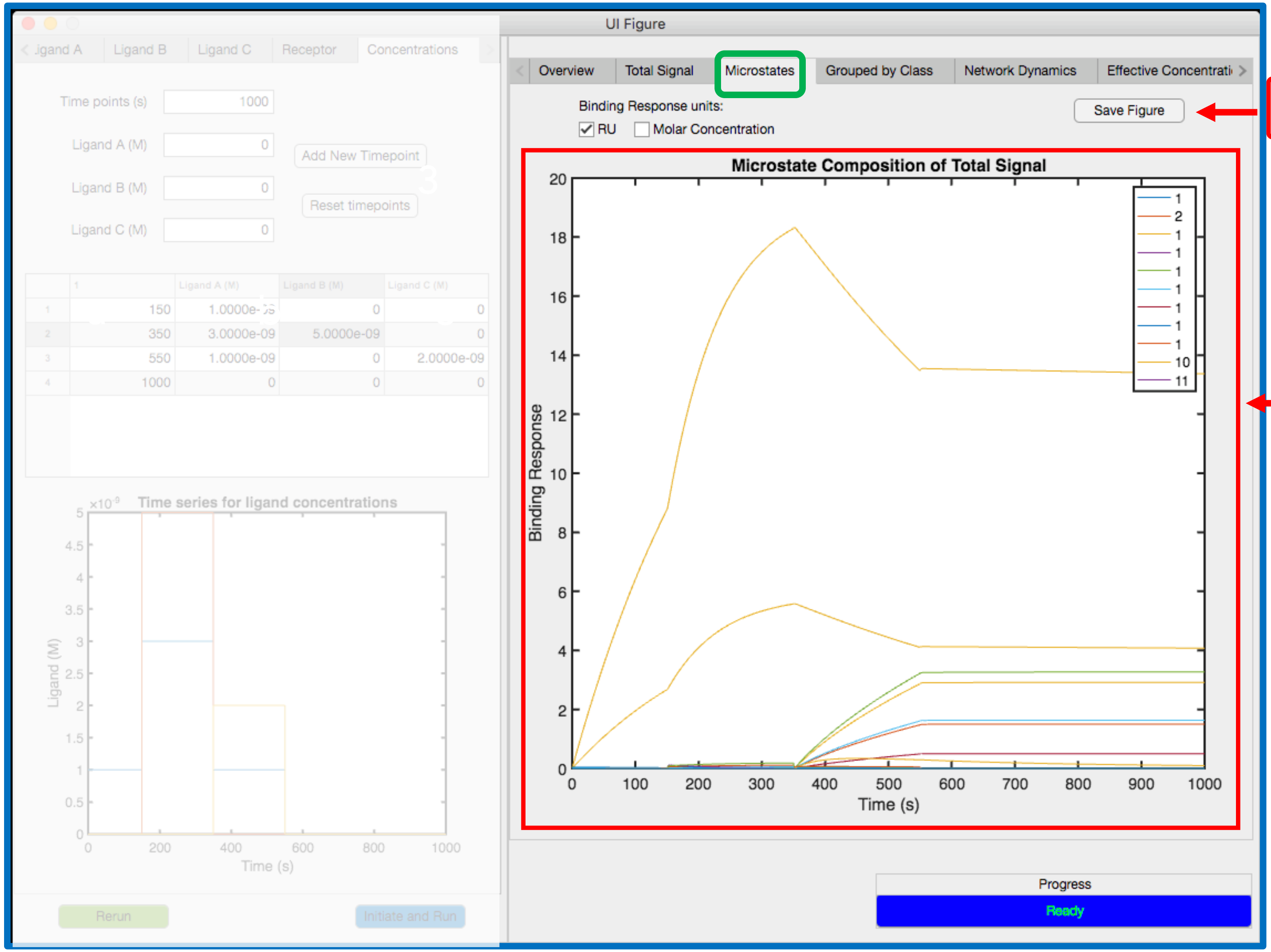

"Save Figure" exports the plot as a .fig file

plot showing all composite microstates that underlie the overall binding response

4. Navigating the *MVsim* output tabs

d. Output Grouped by Class

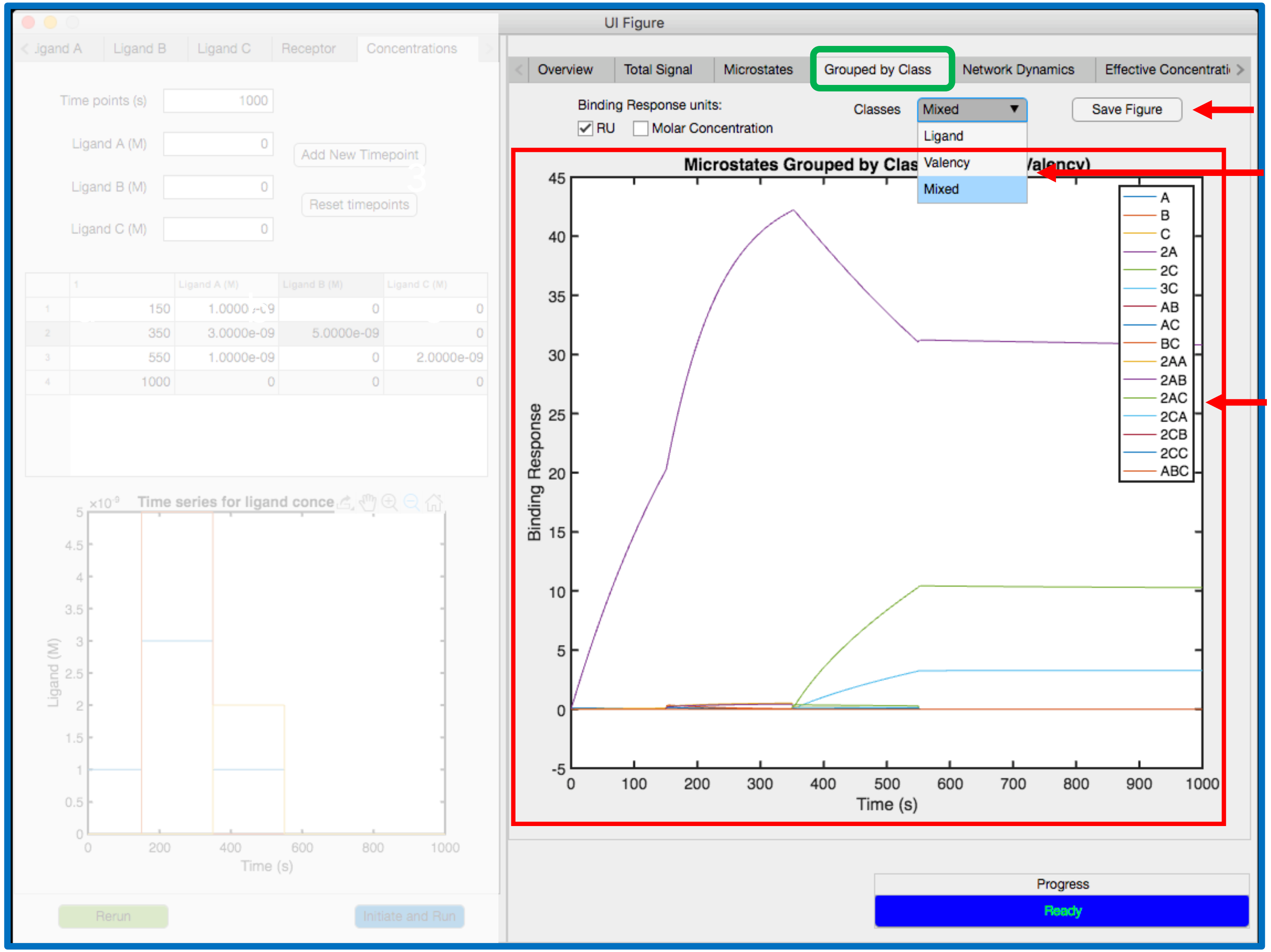

"Save Figure" exports the plot as a .fig file

three "Group by Class" options:
1. group by Ligand
2. group by Valency
3. group by Mixed (i.e., Ligand and Valency)

Plot Legend (for Group by "Mixed")

Here, the letters represent the Ligands composing the grouped microstates, and the numbers represent the valency

E.g., "C" = all microstates composed of one monovalently bound Ligand C

"3C" = all microstates composed of one trivalently bound Ligand C

"2CA" = all microstates composed of one bivalently bound Ligand C and one monovalently bound Ligand A

e. Output Network Dynamics

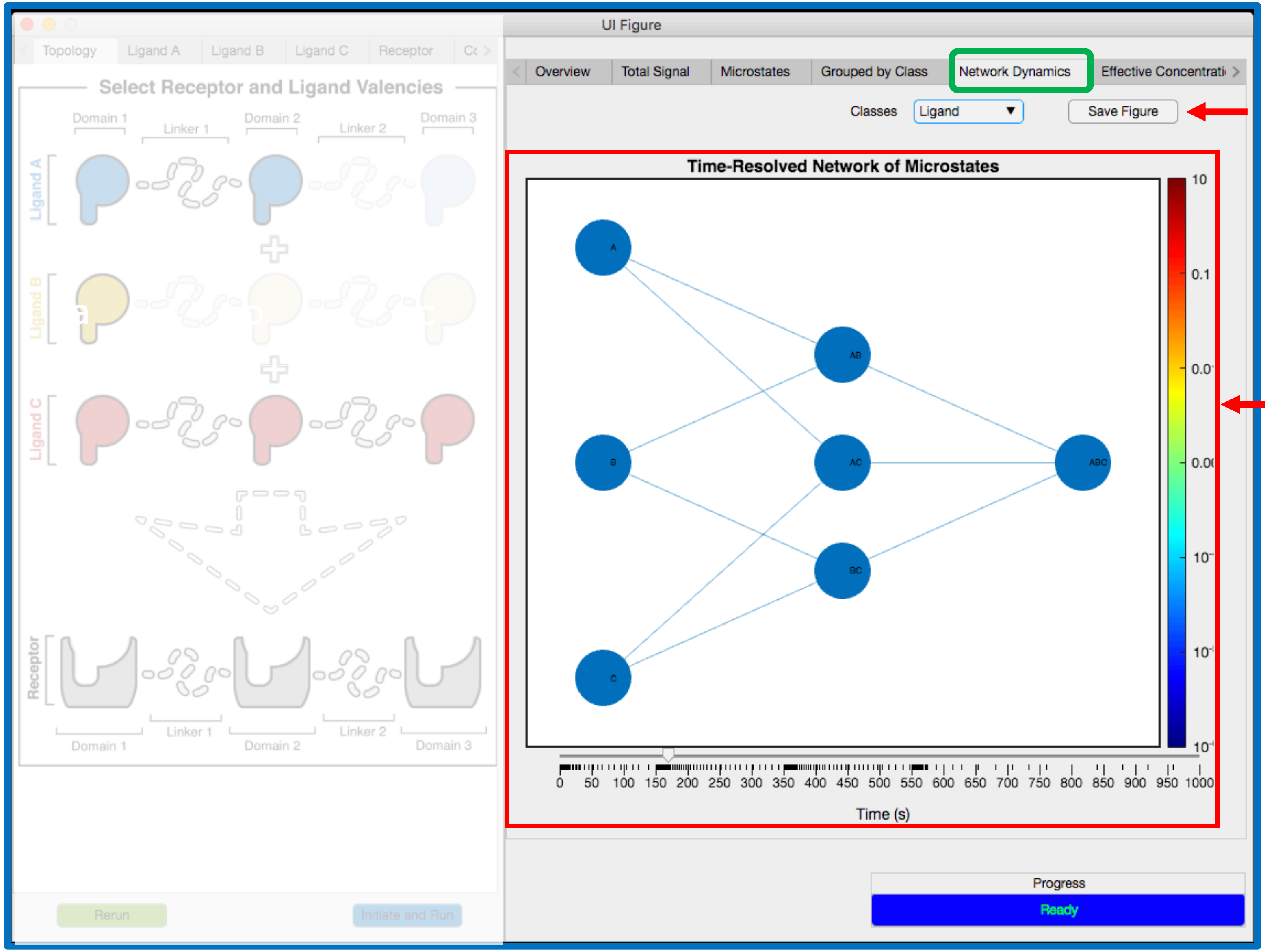

"Save Figure" exports the plot as a .fig file

the "Network Dynamics" tab displays a single view of the animated, "click-and-drag" representation of the microstate binding response dynamics presented in the Overview tab.

This visualization can be rendered according to two grouped classes of microstates: Ligand and Valency

f. Output Effective Concentration

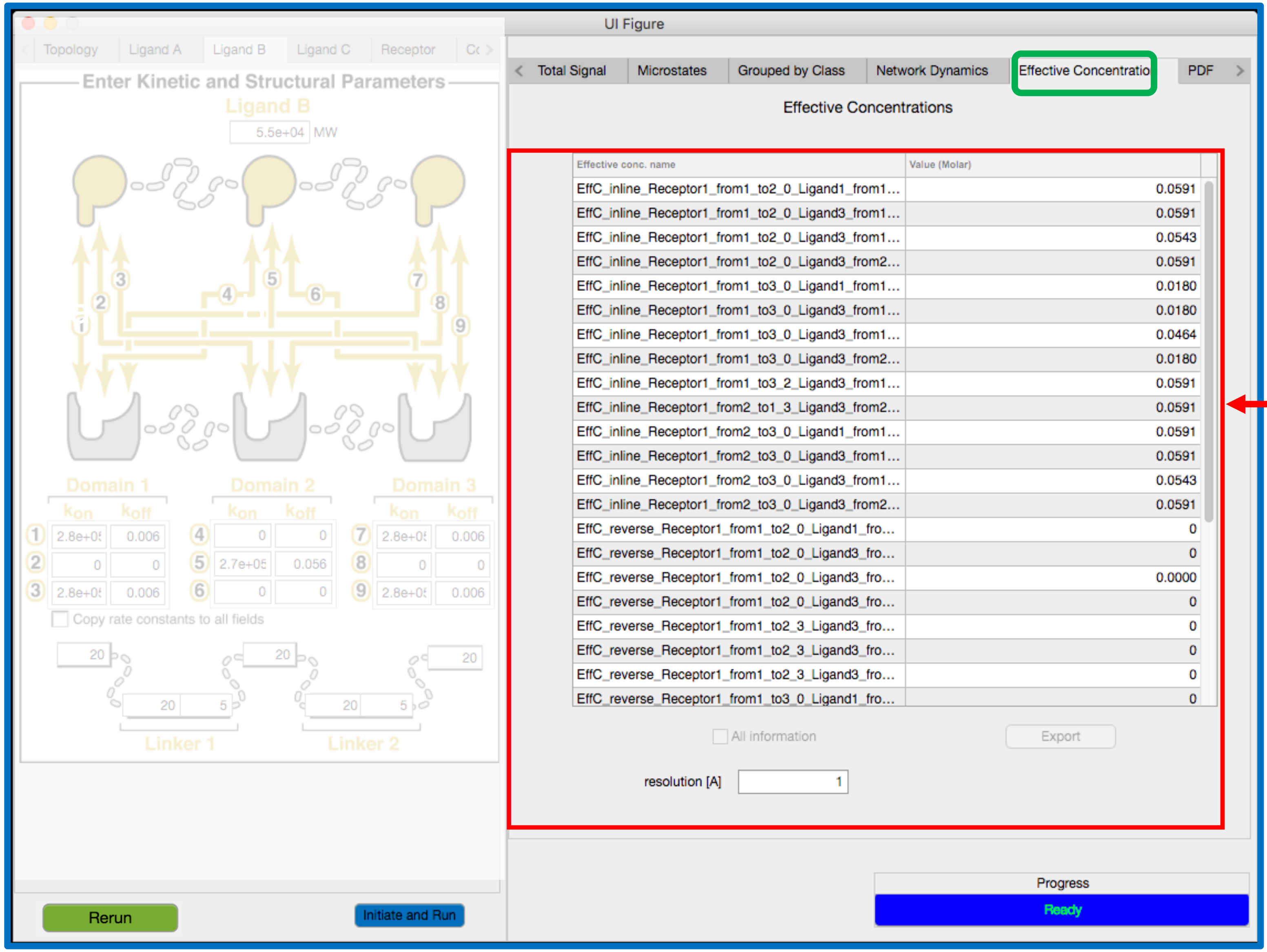

The Effective Concentration tab outputs the list of first-order rate constants of association that correlate with the permissibility of every possible "intra-complex" interaction (i.e., point of multivalent contact) that exists between the simulated ligands and receptor.

The left column lists each type of interaction.

The right column lists the effective concentration in molar units. These values can be manually edited: double click field, re-enter value, press "enter" on keyboard.

The simulation can be re-run with these user-specified values by clicking the "ReRun" button (bottom left). Note: the "ReRun" is highlighted in green when it can be used following an "Initiate and Run"

g. Output PDF (Probability Density Functions)

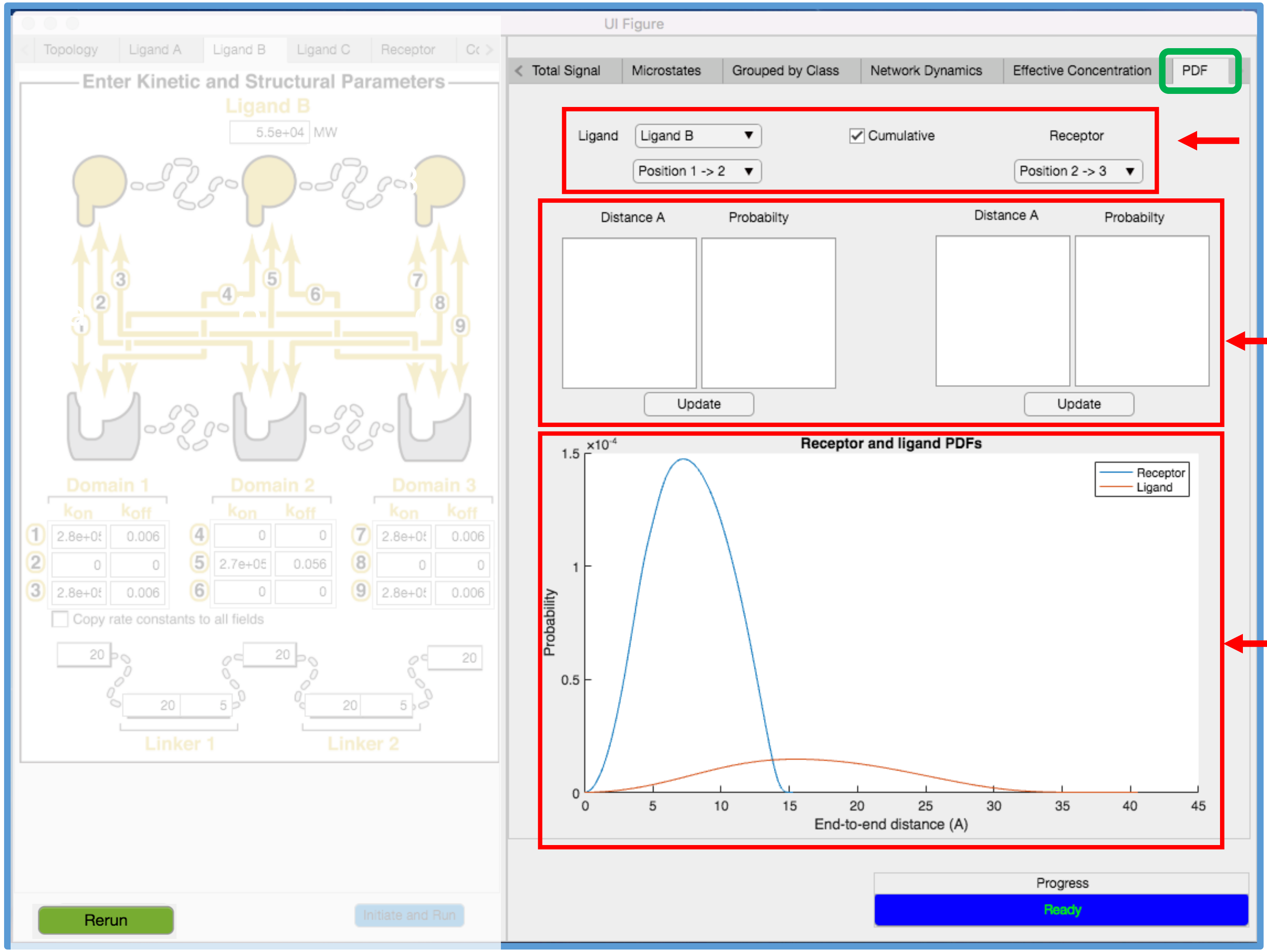

user selection of the PDF describing the probability of finding a domain *x* Å from an origin point. E.g., here, the PDF for Ligand B's domain 2 relative to domain 1; and the Receptor's domain 3 relative to domain 2 are selected from the drop down menus

input fields enabling the user to create their own PDF for the ligand and receptor domains selected above.

The "ReRun" button will re-run the simulation with the user-designed PDFs

graphical visualization of the two PDFs showing the overlap between the distributions of Ligand B domain 2 and Receptor domain 3

4. Navigating the *MVsim* output tabs

h. Batch Input/Output of a Ligand Concentration Series

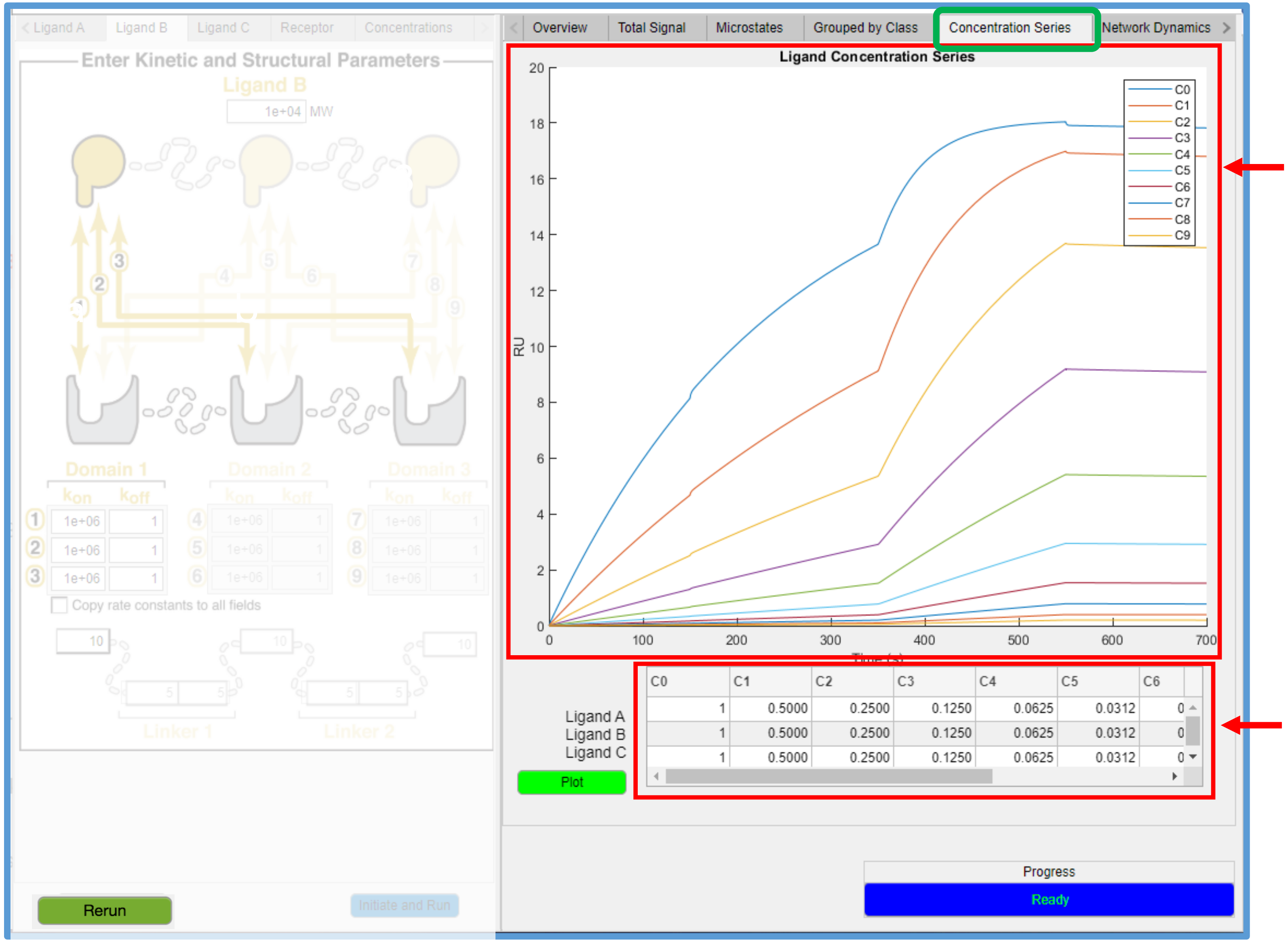

Plot showing the output for nine curves with varying ligand concentrations

The "Plot" button will output the curves for the given ligand concentrations.

The relative concentrations of the ligands compared to the original ligand concentration given in the concentration input tab.

Initially, a twofold dilution series is given for all the ligands. The concentration of the ligands can be kept at the original constant values by entering "1" for each cell in a row. Ligands can be excluded by entering "0" to any cells.

Any concentration can be specified, including relative values larger than 1.

Simulating Multivalent Biomolecular Interactions

Tutorial 2: Advanced Application of MVsim for the S protein

Bruncsics et al. (2022)

1. Parameterizing the competitive inhibition of the SARS-CoV-2 S protein - ACE2 interaction with a trivalent, RBD-binding inhibitor [example taken from Figure 5, Bruncsics et al., 2022]

a. Overview of the components of the multivalent interaction system in the Topology tab

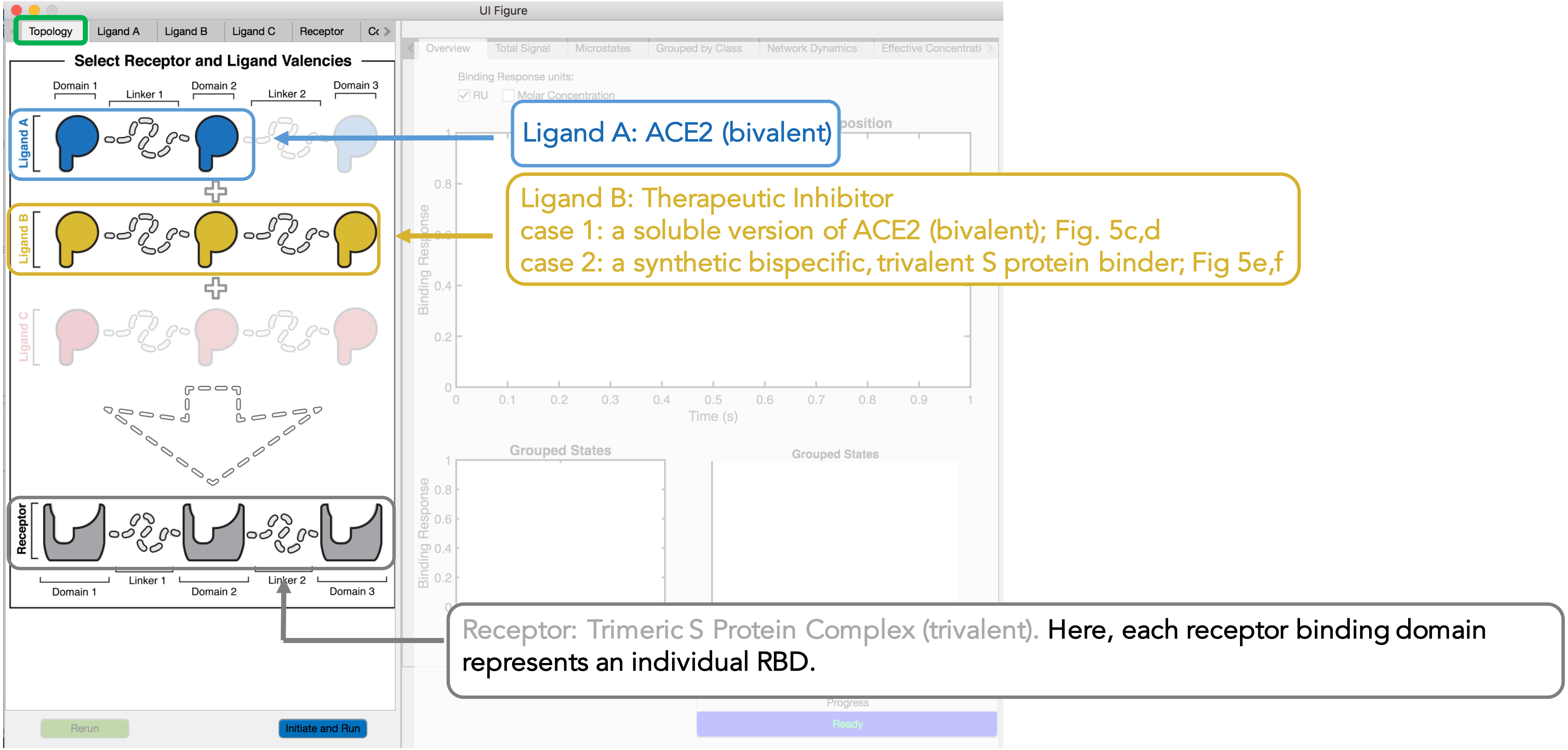

1. Parameterizing the competitive inhibition of the SARS-CoV-2 S protein - ACE2 interaction with a trivalent, RBD-binding inhibitor [example taken from Figure 5, Bruncsics et al., 2022]

b. Parameterizing ACE2 from kinetic and structural data derived from the literature

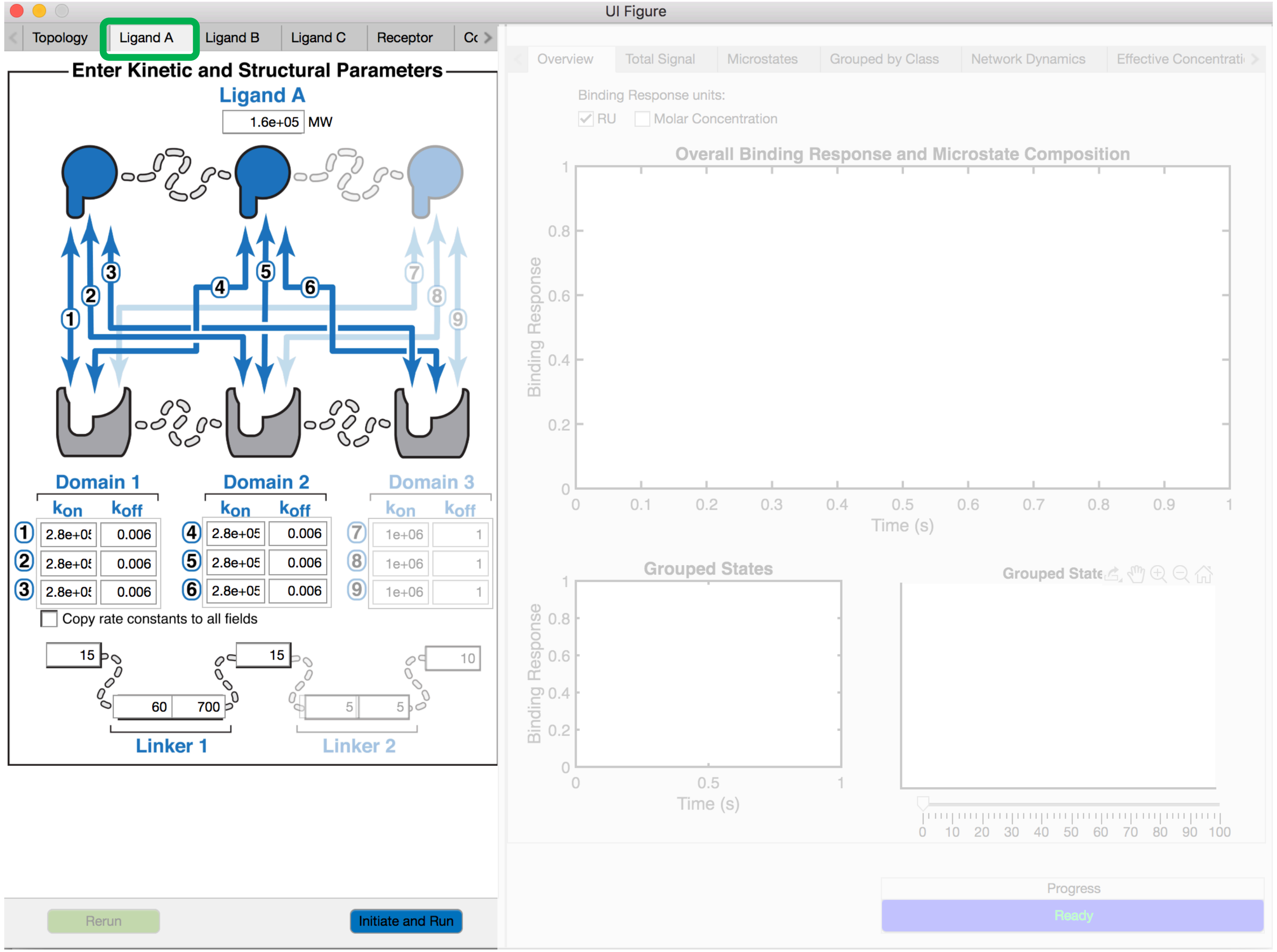

1. Parameterizing the competitive inhibition of the SARS-CoV-2 S protein - ACE2 interaction with a trivalent, RBD-binding inhibitor [example taken from Figure 5, Bruncsics et al., 2022]

c. Case 1: Parameterizing the bivalent, soluble inhibitory ACE2 parameterized from Chan et al. 2020

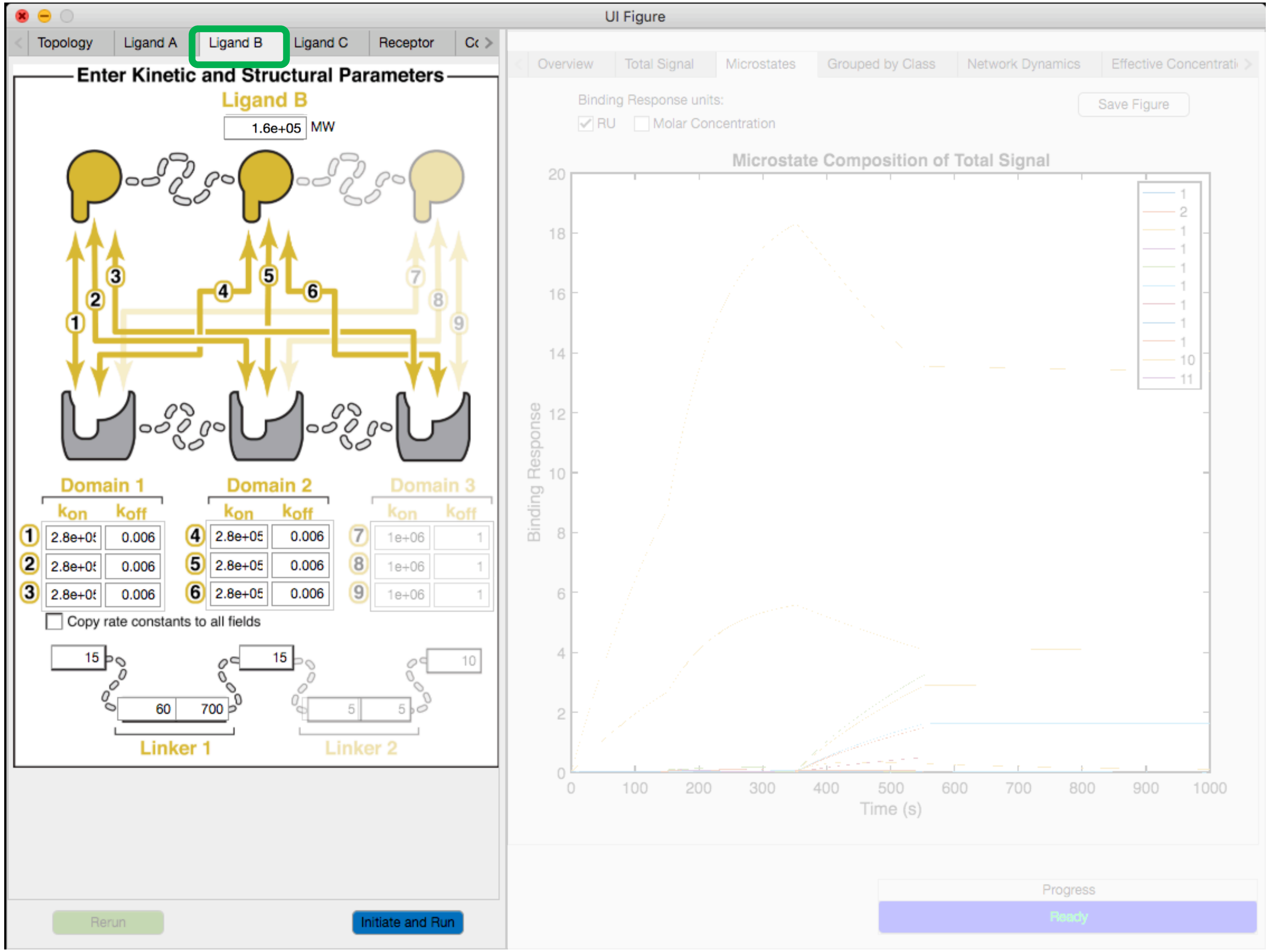

1. Parameterizing the competitive inhibition of the SARS-CoV-2 S protein - ACE2 interaction with a trivalent, RBD-binding inhibitor [example taken from Figure 5, Bruncsics et al., 2022]

d. Case 2: Parameterizing the computationally derived bispecific, trivalent S protein inhibitor

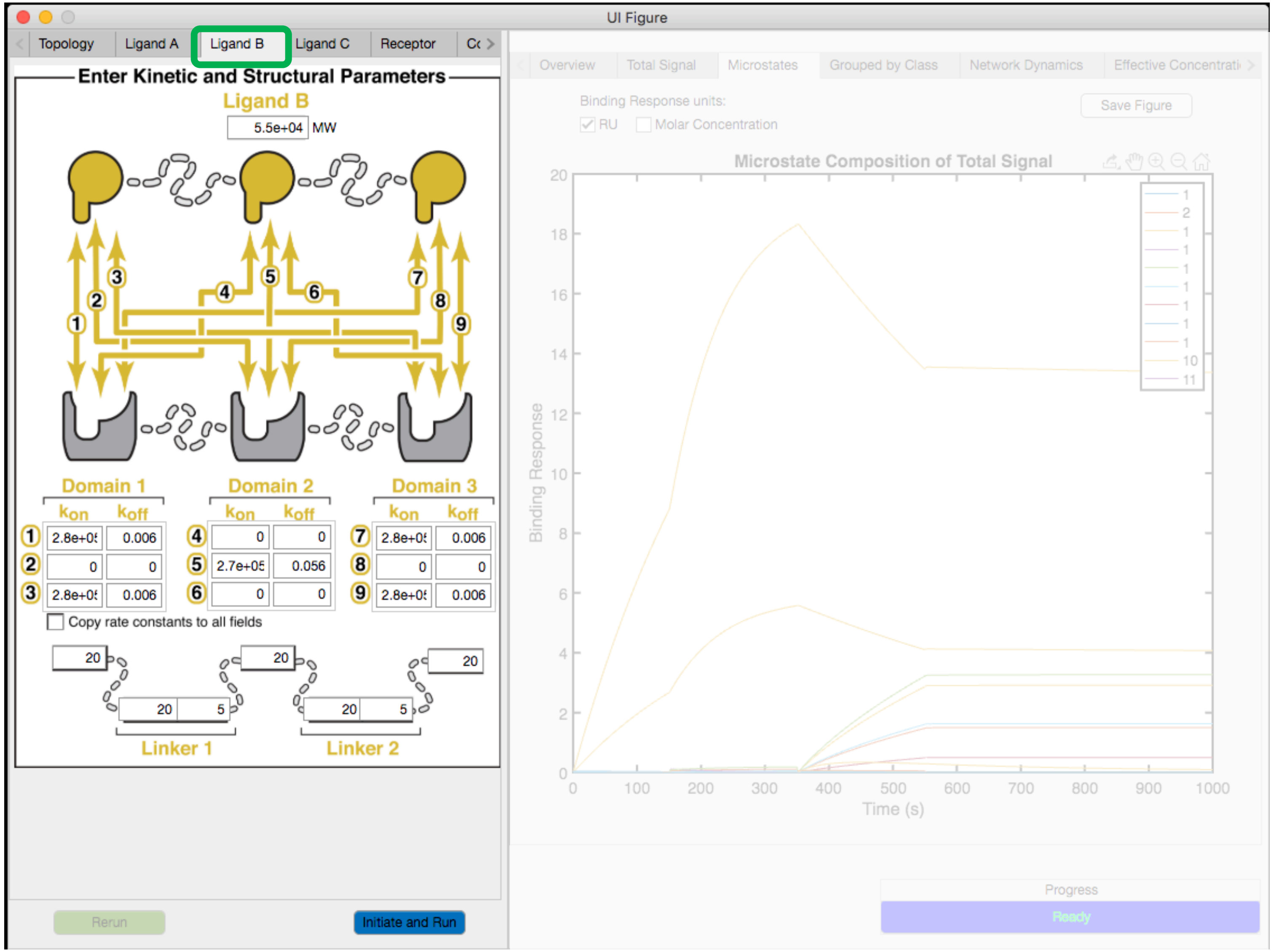

1. Parameterizing the competitive inhibition of the SARS-CoV-2 S protein - ACE2 interaction with a trivalent, RBD-binding inhibitor [example taken from Figure 5, Bruncsics et al., 2022]

e. Parameterizing the trimeric and trivalent S protein from a suite of structural data from the literature

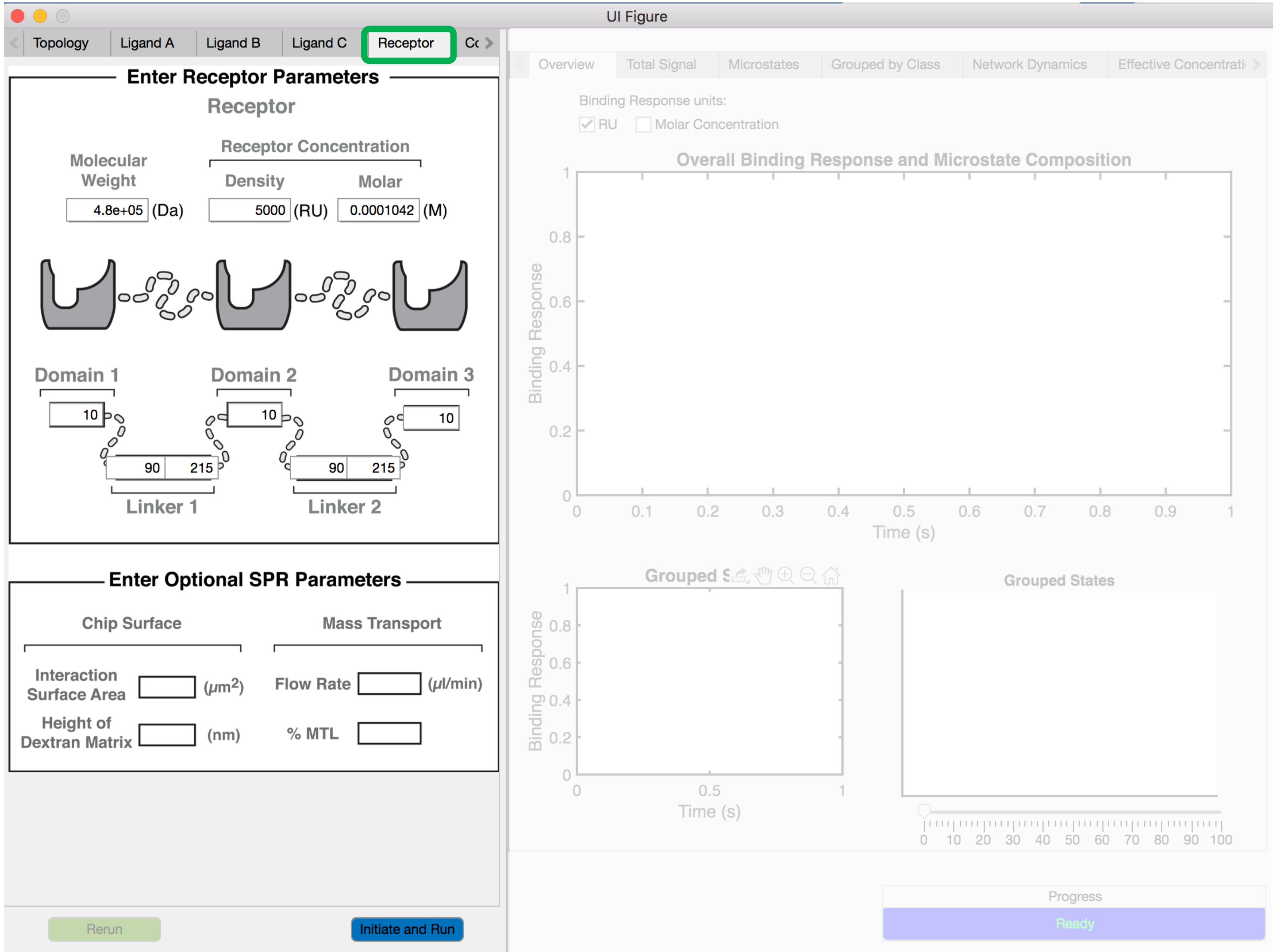

2. Parameterizing the competitive inhibition of the SARS-CoV-2 S protein - S protein interaction with a trivalent, RBD-down-specific inhibitor [example taken from Figure 6, Bruncsics et al., 2022]

a. Overview of the components of the multivalent interaction system in the Topology tab

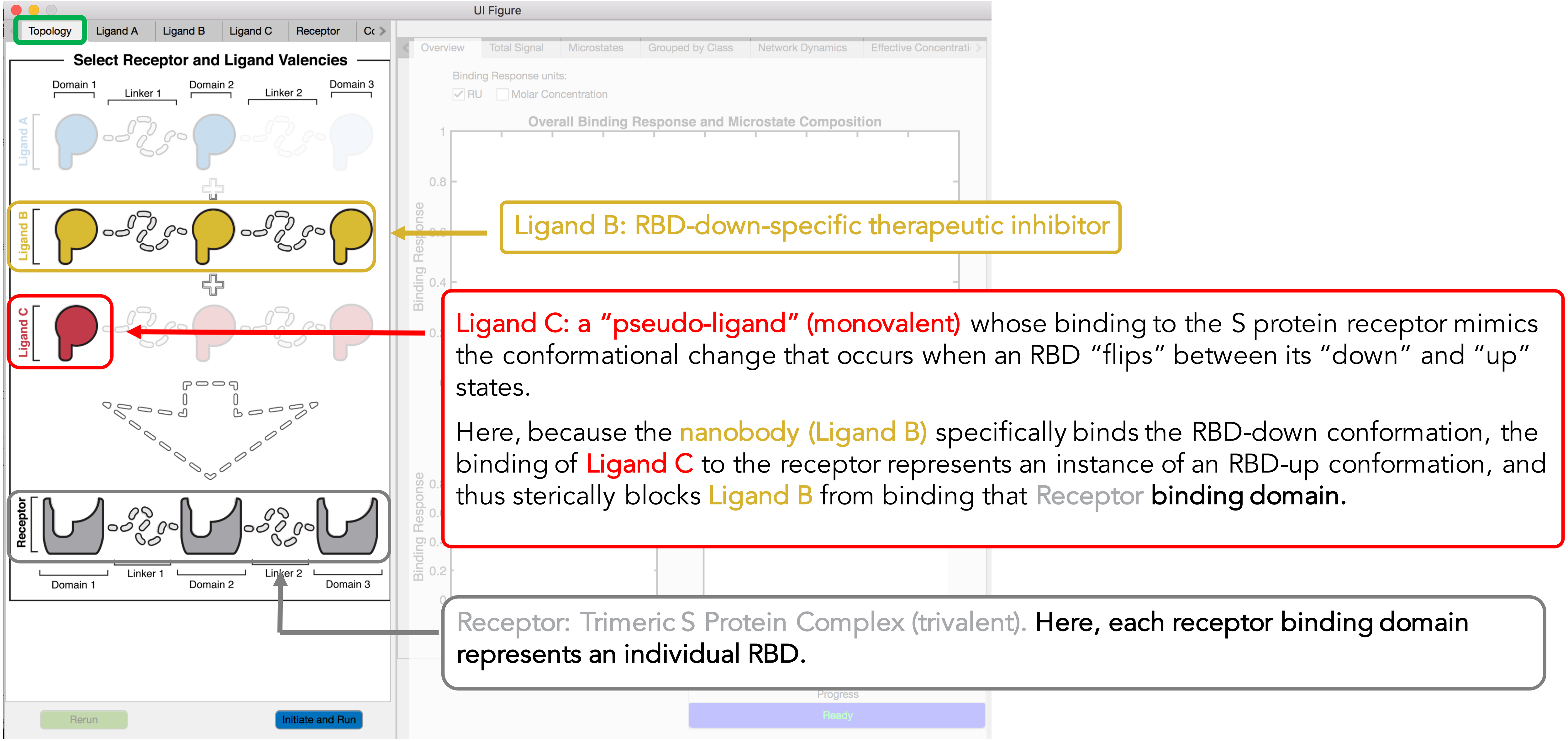

2. Parameterizing the competitive inhibition of the SARS-CoV-2 S protein - ACE2 interaction with a trivalent, RBD-binding inhibitor [example taken from Figure 6, Bruncsics et al., 2022]

b. Parameterizing the trivalent nanobody from kinetic and structural data derived from its primary report

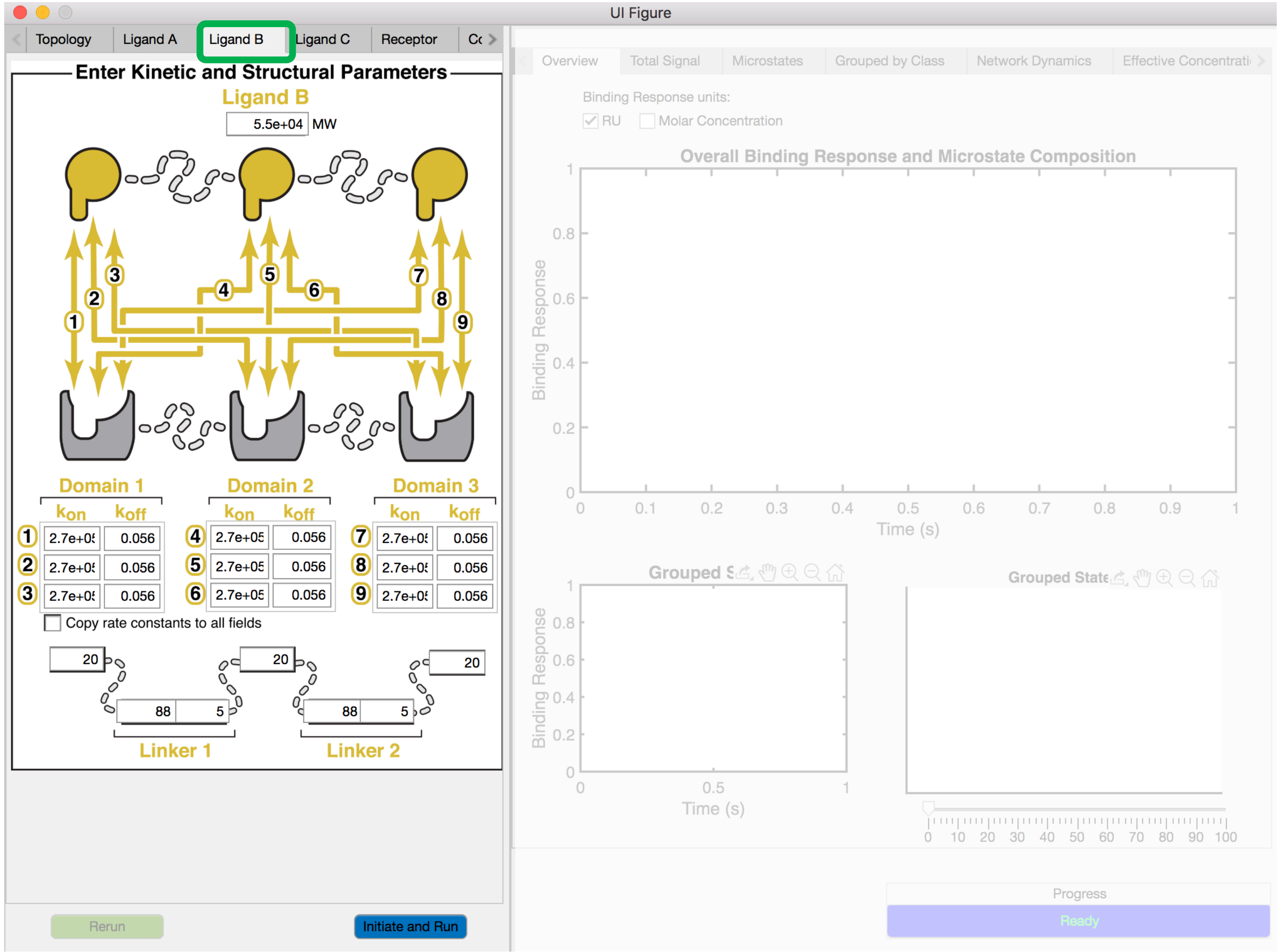

2. Parameterizing the competitive inhibition of the SARS-CoV-2 S protein - ACE2 interaction with a trivalent, RBD-binding inhibitor [example taken from Figure 6, Bruncsics et al., 2022]

c. Parameterizing the S protein RBD "up"/ "down" conformational change

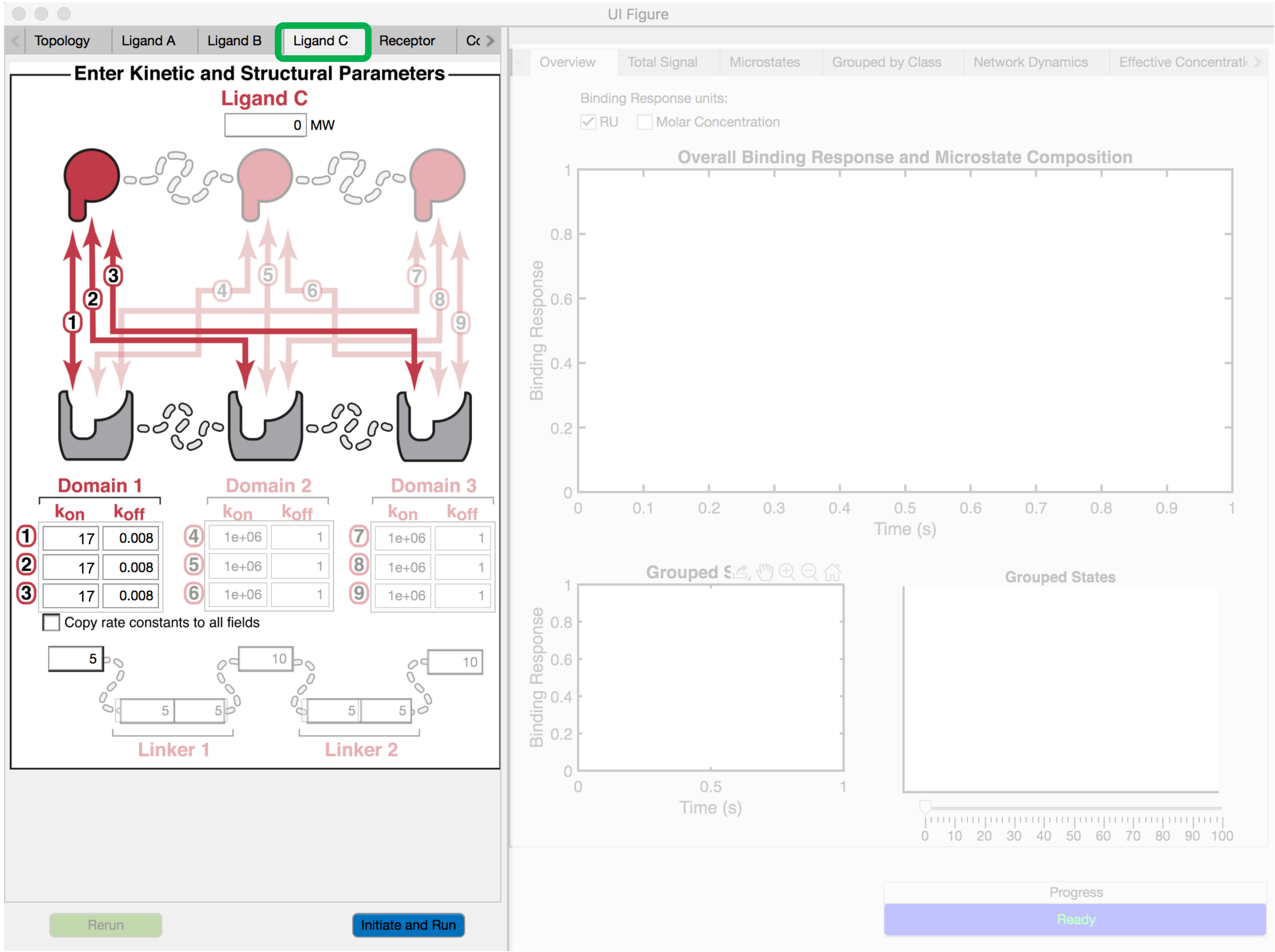

d. Parameterizing the ligand concentrations

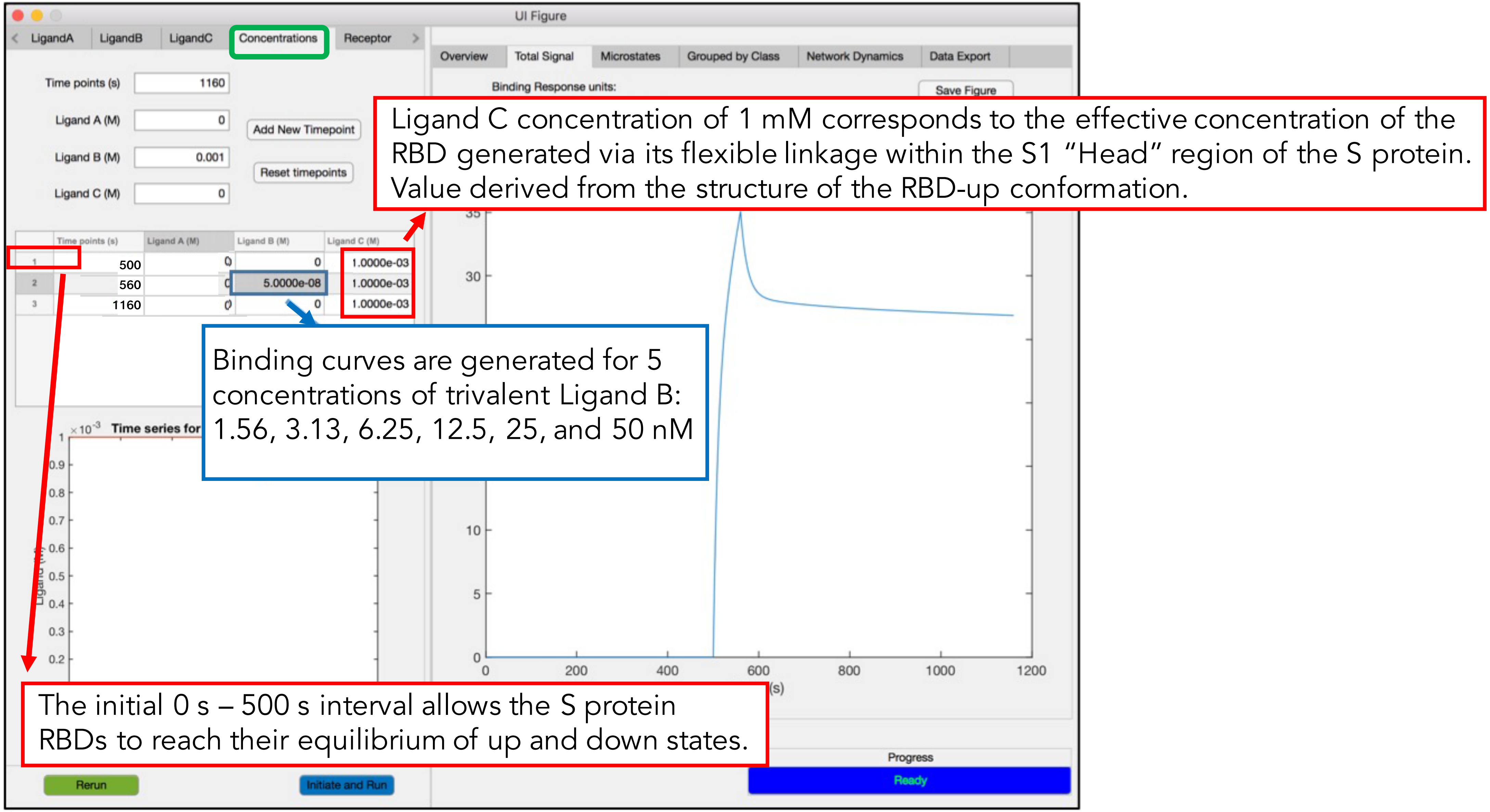

2. Parameterizing the competitive inhibition of the SARS-CoV-2 S protein - ACE2 interaction with a trivalent, RBD-binding inhibitor [example taken from Figure 6, Bruncsics et al., 2022]

e. Parameterizing the trimeric and trivalent S protein from a suite of structural data from the literature

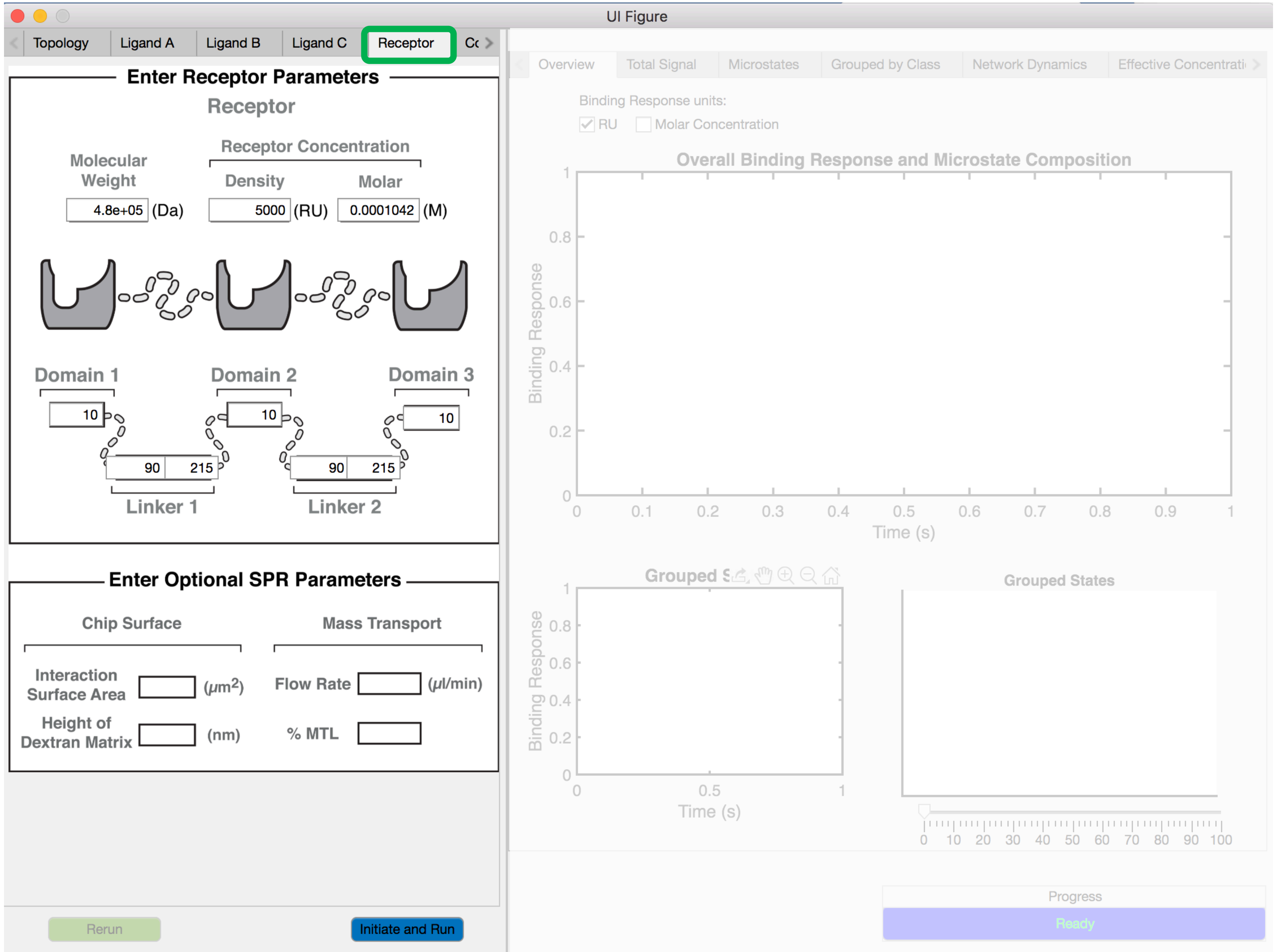

Supplement: Supplementary file 4 — Supplementary Software [file 41467_2022_32496_MOESM4_ESM.zip › MVsim_user_tutorial.pdf]
